# How many people is the COVID-19 pandemic pushing into poverty? A long-term forecast to 2050 with alternative scenarios

**DOI:** 10.1371/journal.pone.0270846

**Published:** 2022-07-08

**Authors:** Jonathan D. Moyer, Willem Verhagen, Brendan Mapes, David K. Bohl, Yutang Xiong, Vivian Yang, Kaylin McNeil, José Solórzano, Mohammod Irfan, Cade Carter, Barry B. Hughes

**Affiliations:** Frederick S. Pardee Center for International Futures, Josef Korbel School of International Studies, University of Denver, Denver, Colorado, United States of America; Cranfield University, UNITED KINGDOM

## Abstract

The COVID-19 pandemic has changed the course of human development. In this manuscript we analyze the long-term effect of COVID-19 on poverty at the country-level across various income thresholds to 2050. We do this by introducing eight quantitative scenarios that model the future of Sustainable Development Goal 1 (SDG1) achievement using alternative assumptions about COVID-19 effects on both economic growth and inequality in the International Futures model. Relative to a scenario without the pandemic (the *No COVID* scenario), the *COVID Base* scenario increases global extreme poverty by 73.9 million in 2020 (the range across all scenarios: 43.5 to 155.0 million), 63.6 million in 2030 (range: 9.8 to 167.2 million) and 57.1 million in 2050 (range: 3.1 to 163.0 million). The *COVID Base* results in seven more countries not meeting the SDG1 target by 2030 that would have achieved the target in a *No COVID* scenario. The most pessimistic scenario results in 17 more countries not achieving SDG1 compared with a *No COVID* scenario. The greatest pandemic driven increases in poverty occur in South Asia and sub-Saharan Africa.

## Introduction

The sustainable development goals (SDGs) include a broad set of human development outcomes, with the first target indicator aiming to, “end poverty in all its forms,” by 2030 [1]. Over the previous 20 years, much progress has been made to improve the livelihoods of millions around the world by reducing extreme poverty, alleviating malnutrition, and improving human development capabilities [2]. However, the spread of COVID-19 is expected to disrupt these development gains, making it more difficult for economies to function, for individuals to earn income, and for governments to raise revenues.

Previous research has analyzed long-term global poverty trajectories in the absence of COVID-19 [3–6] and explored the possibility of achieving SDG 1 across various socio-economic scenarios [7, 8], with estimates ranging from 1% to 18% of the global population living in extreme poverty by 2030. Recent analyses suggest that COVID-19 will increase poverty in the short-run but we have less insights about the potential longer-term effects of COVID-19 on extreme poverty projections in the SDG horizon or beyond [9–12]. An exception is a study by Lakner et al. (2022) that does incorporate COVID-19 into poverty projections to 2030, but does not quantify the effect relative to a world without COVID-19. This paper addresses this gap.

We introduce eight scenarios modeling different assumptions related to economic growth and income distribution that reflect two key dimensions of uncertainty associated with the COVID-19 global pandemic, operationalized within the International Futures (IFs) integrated assessment model. We begin by comparing a *No COVID Base* against a *COVID Base* scenario modeling the future of global poverty following the current country-level development trajectory with and without COVID-19 effects on GDP growth. We then create six additional uncertainty scenarios with alternative assumptions related to uncertainty associated with the effects of the global pandemic on GDP growth and income inequality for 186 countries from 2021–2050.

## COVID-19 and development: Dimensions of uncertainty

Household income and its distribution are two key determinants of poverty levels [4, 13, 14], as well as two major sources of uncertainty associated with the impact of COVID-19 on development. Here we review our current understanding of how COVID-19 is changing patterns of economic growth as well as the distribution of resources. Across the studies surveyed in this section, we identify greater consensus in the understanding of the effect of COVID-19 on economic growth, and greater uncertainty when analyzing its effect on resource distribution.

The macro-economic impacts of the virus have been broad and complex, effecting development patterns across changing patterns of labor participation [15], economic growth [16], changes in trade, remittances, foreign direct investment [17], education [18] and more [19]. COVID-19 has proven distinct from other economic crises by its constraining effects on both supply-side production and demand-side consumption [20]. These dual shocks have impacts with considerable variation across countries as governments implement a range of policy responses for both virus containment and economic stimulation.

Labor markets were affected in unprecedented ways in 2020 with working-hour losses approximately four times greater than in the 2009 global financial crisis [15]. The ILO estimates that 8.8 percent of global working hours were lost in 2020 relative to the fourth quarter of 2019–equivalent to 255 million jobs [15]. Although there was a larger rebound than anticipated in working hours in the second half of 2020, global working hours still declined by 4.6 percent in the fourth quarter of 2020 [15]. The labor market rebound seen in the second half of 2020 did not continue into 2021, as estimations for lost labor hours in quarters one, two, and three of 2021 represent a respective 4.5, 4.8, and 4.7 percent decrease from the fourth quarter of 2019 [21]. In the United States alone, the economic consequence of lost working hours amounts to an estimated $138 billion [22].

The World Bank’s Global Economic Prospects (GEP) presents anticipated economic recovery levels in 2022 and 2023. Following an economic rebound in global GDP growth to 5.5 percent in 2021, the GEP predicts a pullback in both 2022 and 2023, to 4.1 and 3.2 percent respectively [23]. However, variation in factors such as the speed and efficacy of vaccination program and economic policy implementation as well as structural characteristics such as reliance on tourism, trade, or remittances will lead to significant variation in how quickly economies recover. As such, emerging markets and developing economies may face more significant lasting effects while advanced economies are likely to recover more quickly [24].

While COVID-19 has direct and measurable effects upon the growth of overall economic output, its effect on the distribution of income is less clear and historical evidence from other pandemics is mixed. In prior pandemics, the working-population mortality rate is a key determinant in driving reductions in income inequality, with higher rates associated with labor shortage driving an increase in real wages [25–27]. The Black Death, for example, decreased income inequality in both England and Italy due to a large mortality-driven decrease in the labor supply [25, 26, 28, 29]. Pandemics may also create a production crisis as well as prompt a reduction in consumption due to an increase in savings, thus reducing the rate of return on capital and disproportionately affecting the wealthy [27, 30]. Some previous pandemics have increased inequality, for example in 1629–1630 [28]. COVID-19 is unlikely to increase the wage-rental ratio, as the death rate is not high enough nor are its consequences evenly distributed enough across low and high paying occupations to have lowered inequality on its own [31].

Another dimension of inequality relates to the distribution of job losses and reduced output across sectors. Job losses from COVID-19 have been disproportionately located within certain sectors including hotels and restaurants, agriculture, construction and commerce [32, 33] making certain workers, industries, countries and regions more vulnerable to pandemic driven downturns [24]. Generally, remote work has been more common in better educated and higher paid industries [34] while more labor-intensive industries and those with limited use of information and communications technology have proven less amenable to remote work [32]. Persistent job losses and the impact on consumer spending, savings and productivity will continue to prolong recovery, especially in countries with a large portion of labor-intensive industries and weak social safety nets. However, the effects of COVID-19 on inequality are not limited to the characteristics of the virus itself but include the reactions of the societies that it affects; herein lies the uncertainty as to the overall effect of the pandemic upon inequality.

The emerging evidence concerning the effects of COVID-19 on between-country inequality is mixed but seems to point towards an increase. On a between-country basis, international income inequality was found to have both decreased [35], increased [36], and increasing when countries are weighted by population [35]. This is due in part, to the influence of both China and India over population weighted studies, as rising incomes in China were not able to fully offset declining incomes in India [35]. Other studies have estimated an increase in the global Gini-index of 1.2–2% based on previous pandemics [37], whereas preliminary data suggest the between country Gini-index increased by 0.4 points throughout 2020 [23]. While this increase is lower than some predicted, it is still significant as it returned between-country inequality to levels last seen during the early 2010s [23].

Evidence for effects of COVID-19 on within-country inequality is highly heterogeneous and can be both positive and negative. Within-country inequality experienced an estimated increase, of 0.3 points in emerging market and developing economies and 0.4 points in low-income countries [23]. A working paper by Hill & Narayan [38] provided evidence for the high uncertainty and heterogeneity of Covid-19’s effects upon short-to-medium term within-country inequality; but also concluded that Covid-19 will likely generate negative consequences for within-country inequality in the long-term. Additional country-level research sheds light onto the differential impacts of COVID-19 on within-country inequality, including research examining Mexico, Brazil, Argentina, and Colombia. In Brazil, within-country inequality fell by an estimated 2.9 points below its pre-pandemic levels [39]. In Mexico, the Gini-index had an estimated increase of 1.3–3.7% for the 2020 fiscal year [40] while Columbia and Argentina both experienced an estimated increase of only 0.9 and 0.1 points during 2020 [39].

Overall, data and analysis with respect to the growth and inequality impacts of COVID-19 during the pandemic period continue to emerge, but the implications of those impacts through the 2030 horizon of the current SDGs and beyond remain highly uncertain. This reality affects the scenarios used in this study that will be elaborated below.

## Poverty projections and COVID-19

Projections of poverty often include quantitative simulations that model structural factors in isolation or interaction. These models frequently include assumptions or dynamics related to three sets of variables: a) growth in economic production/income/consumption [41–44]; b) the distribution of resources/income/consumption [13, 45]; and c) demographic change. Extensions of earlier approaches have innovated in their representation of income distribution [7], integrated poverty research with Shared Socio-economic Pathways (SSPs) long-term economic growth projections [7, 46], developed income distributions along the SSP projections [47], studied the relative importance the Gini-index for extreme poverty [10], or integrated these three systems dynamically in one framework [4, 5, 8, 48].

**Fig 1:**
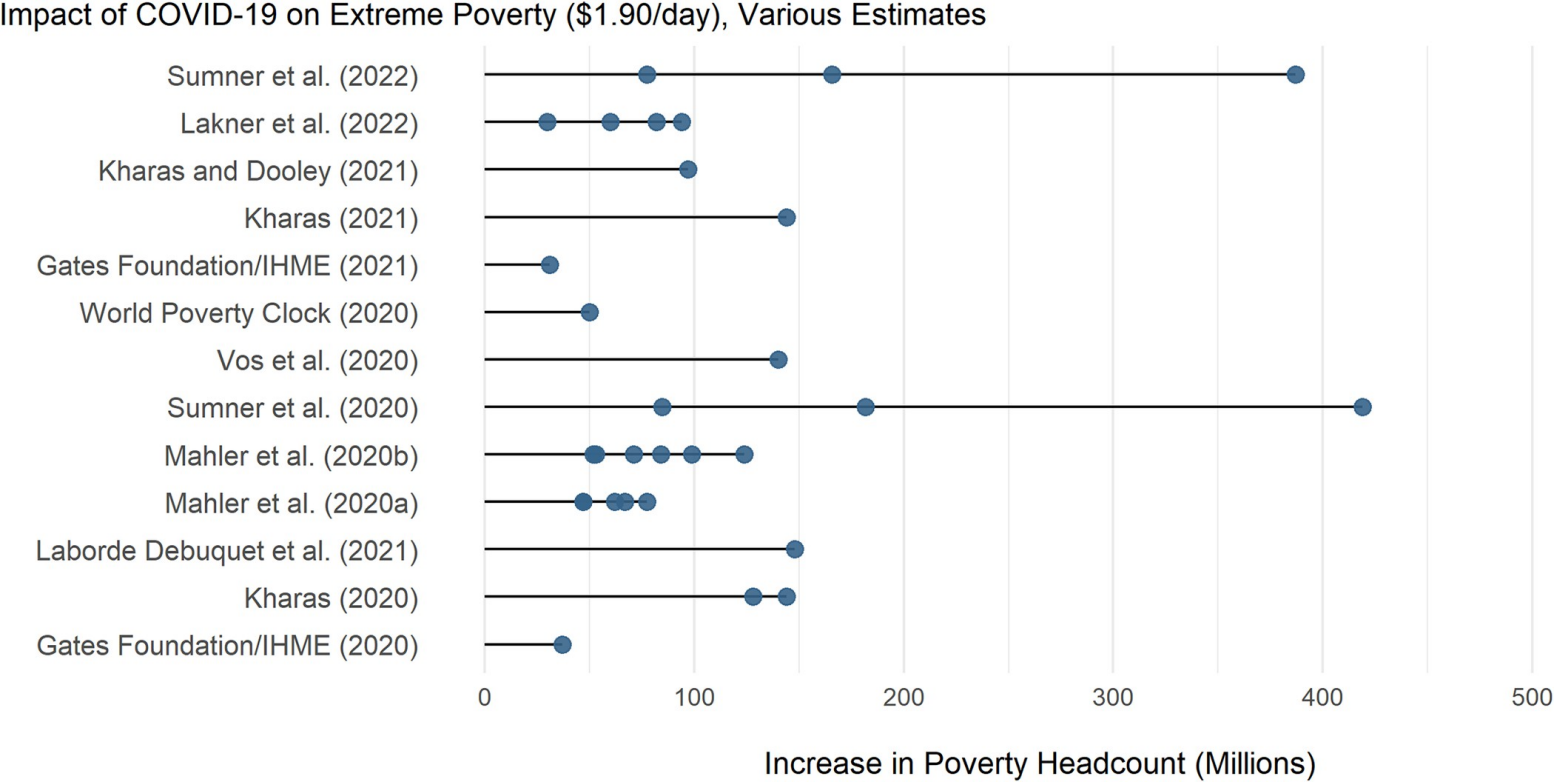
The effect of COVID-19 on poverty: Early estimates from grey literature in millions. If no impact year was specified in the original study, it was assumed that the year assessed for the impact of COVID-19 was the year of study publication.

COVID-19 caused many researchers to revise their estimates of global poverty often focused on pandemic-period impacts (Fig 1). Sumner et al. [12, 49] provide early and updated poverty estimates using an “augmented poverty line approach” to model the impact of income per capita contraction across various scenarios due to COVID-19. The updated study estimated that between 77 and 390 million people could fall into extreme poverty due to the pandemic, revised downward from 89 to 419 million people in the earlier version of the study.

Research using MIRAGRODEP (a dynamic recursive CGE model built on a microsimulation framework) projects that COVID-19 will have increased extreme poverty by almost 150 million in 2020, with the majority of increased poverty burden (~80 million) occurring in sub-Saharan Africa [50].

Other estimates of the short term impact of COVID-19 on poverty include: a) analysis from Kharas & Hamel [51] and Kharas & Dooley [9] showing that COVID-19 could increase global extreme poverty by 50 million people and 97.1 million people respectively in the short term; b) figures from Mahler et al. [11] showing that COVID could increase poverty between 40–60 million in 2020; and c) figures from the Bill & Melinda Gates Foundation and IHME [52] report an initial poverty increase of 37 million driven by the global pandemic, with a more recent report citing a 31 million increase. Lakner et al. [10] and provides a number of alternative scenarios that vary GDP and Gini-index assumptions by 1%. The analysis from Kharas & Hamel [51] and Kharas & Dooley [9] build on the methodology from Crespo-Cuaresma et al. [7] used for the World Poverty Clock to account for decreased global economic output. A summary of these alternative estimates is shown in Fig 1.

Many of the studies surveyed in Fig 1 do not include an assessment of the impact of COVID-19 beyond the short-term (2020–2021). Exceptions include work associated with the World Poverty Clock project, estimating that COVID-19 will increase poverty by 100 million in the short-run and 50 million by 2030 [9]. Reedy [53] also explores the effect of different GDP growth rates on 2030 poverty outcomes, with a range of 6%-11% under high and low growth scenarios, respectively, but shifts in within-country inequality are not explored in the study. There remain very few estimates of the impact of COVID-19 through 2030 on global poverty in academic literature. Lakner et al. [10] is an exception, which builds upon earlier work from the same authors to test the impact of a range of growth rate and inequality assumptions on the effects of the pandemic across the SDG horizon, estimating poverty ratio outcomes ranging from 5.3%-11.7% by 2030. This study however, does not seek to explicitly estimate the progress lost on poverty reduction due to the COVID-19 pandemic over the SDG horizon.

## Modeling methodology

IFs models economic growth and household consumption, income distribution at various thresholds, and demographics to forecast poverty headcounts under alternative scenarios for 186 countries through the year 2050. The poverty model is embedded within a larger system of agricultural, demography, education, economic, energy, environmental, health, infrastructure, governance, security, and technological models [54]. IFs has been used in a variety of research on poverty and other measures of human development [4, 5, 8, 48, 55–57], with a recent focus on the effects of COVID-19 on extreme poverty, and more generally human and economic development [17, 19, 58–60].

The full model, and individual sub-modules, have also been documented in several publications [4, 54, 61–64] and documentation and model are freely available for use (https://pardeewiki.du.edu/index.php?title=Understand_the_Model). The following sections outline how IFs forecasts changes in average income as a result of economic growth, how changes in average income are transformed to changes in average consumption, and the calculation of income distribution using the Gini-index. These components are the input into a log-normal distribution to calculate extreme poverty per country.

### Using economic growth, disposable income and a social accounting matrix (SAM) to forecast consumption

Existing poverty models base their projections on changes in average income or average consumption [3, 6, 46]. The IFs model also uses consumption to drive its poverty forecasts using three steps. First, IFs projects economic growth using a Cobb-Douglas production function that determines value added by economic sector from capital, labor and productivity. That economic activity drives gross household wages, a key source of income. Second, these earnings are considered along with other income and expenditure flows, for example taxes and transfers, in a social accounting matrix (SAM) that computes a measure of disposable income. Finally, disposable income is used in a multi-step process to calculate final consumption in balance with final savings.

To elaborate, economic growth projections are calculated in the IFs economic model, a dynamic recursive computable general equilibrium tool that uses a Cobb-Douglas [65] production function (broken down by six sectors) representing labor (broken down by skilled and unskilled) and capital with a Solow residual [66] representing productivity [54]. Changing patterns of labor supply are driven by the IFs demographic model (more below), while changing patterns of investment and depreciation drive growth in capital stocks. Changing productivity dynamics are driven by an integration with other IFs submodels and factors representing human capital, physical capital, social capital, and knowledge capital [67]. ${GDP}_{r}=\sum^{S}\left[{CDa}_{r,s,t=1}*{{TEFF}_{r,s}*CAPUT}_{r,s}*{{KS}_{r,s}}^{{AlphaS}_{r,s}}*{{LABS}_{r,s}}^{\left(1-{AlphaS}_{r,s}\right)}\right]$ where r is country/region, s is economic sector, TEFF, KS, LABS and CAPUT are sector specific values of total factor productivity, capital, labor and capacity utilization. CDA is a scaling factor computed in the base year to make model computations consistent with historical data.

Economic growth drives changes in gross income, the next step in the calculation of overall consumption. This process begins with an estimate of country-level household earnings measured as the share of value added that goes to labor. Next, to translate earned income into disposable income (the income that a household can choose to either consume or save), the model computes the other financial flows relying on a SAM structure. Data to initialize the production function and the broader SAM come from GTAP, the World Bank, and IMF [24, 68, 69] with priority attention to sources based on the extensiveness and recency of data, as well as on the differential focus of the sources on the variable. When data do not exist for specific country values, holes are generally filled using cross-sectionally functions of GDP per capita at purchasing power parity.

The SAM represents monetary flows within and across economies and includes the following elements: a) actors (households, governments, firms) and b) domestic and global financial flows among these actors (including input-output matrices for each country with related sectoral flows, governmental revenue and expenditure streams, trade, investment, remittances, aid, loans, etc.) [70].

Various processes augment or reduce earned income on its way to the calculation of final consumption. Tax policies reduce household earned incomes and are modeled using variables measuring value-add, income, and pension taxes. Government transfers augment household earned income and include cash transfer and pension payments (Initial data values come from the IMF, World Bank, and OECD Social Expenditure database). Returns on investment and dividend payments are also represented as augmenting factors to earned income and are computed from gross fixed capital formation (aka investment) and firm income. Firm income is computed from firm earning and firm taxes including indirect taxes. Earning is computed from value added and its capital share. Those are initialized from GTAP data, with some economic data coming from WDI and IMF. Data on corporate and indirect taxes come from the IMF. Finally, international financial flows can also augment or detract from earned income, depending on whether a country is a net sender of remittance payments with data initialized from the World Bank.

$${HHINCDIS}_{r,h=1}={HHINCEARN}_{r,h=1}+{GOVHHTRNWEL}_{r,h=1}+{GOVHHTRNPEN}_{r,h=1}-{HHTAX}_{r,h=1}-{HHGOVSS}_{r,h=1}+{HHDIVINT}_{r,h=1}+{XWORKREMIT}_{r}$$

where r is country/region, h is the type of household categorized by skill level, HHINCDIS is household disposable income, HHINCEARN is household earned income, GOVHHTRNWEL is government to household welfare transfers, GOVHHRTNPEN is government to household pension transfers, HHTAX is income tax, HHGOVSS is pension tax, and XWORKREMIT is net incoming and outgoing remittances flows.

At the end of this process, the model calculates country-level disposable household income which is then used in the final stage to calculate overall consumption. To calculate consumption, IFs relies on a multiple-step process that factors in a) global patterns of changing consumption/savings based on levels of development; b) the age structure of a society; and c) signals from changing patterns of price and interest rates. To connect changing patterns of consumption propensity with levels of development, IFs calculates a long-range consumption target as a share of price adjusted GDP potential, using the historical relationship of consumption with GDP per capita at PPP. A preliminary consumption value is then computed from a consumption ratio of income driven by level of development and responsive to the gap in household saving needs and converges to the target value over 150 years. Next, following insights from Modigliani [71], we track the life-cycle savings and consumption patterns reflecting an understanding that both young and old have higher levels of consumption relative to income compared with working age individuals.

Finally, IFs adjusts the relationship between consumption and savings based on signals sent from two price mechanisms: interest rates and sectoral prices. Both interest rates and prices are calculated across time in IFs using long-term equilibrating mechanisms that are connected to underlying inventory stocks. For instance, if inventory stocks fall as a portion of annual production, prices and interest rates rise and over time direct more of income to savings. The final product of this multi-step process is country-level consumption, which is an input to the next stage of modeling poverty.

### Calculating the distribution of consumption

There are multiple approaches and functional forms used to estimate the distribution of consumption [10, 45, 46]. IFs estimates poverty using the assumption of a log-normal distribution of per capita consumption [13]. The log-normal functional form can be specified using two parameters: mean consumption and its standard deviation. In contrast to income and consumption, we treat Gini-index as exogeneous, holding it constant in the *Base* scenario and adjusting it for alternative scenarios. Poverty rates are initialized using data from PovcalNet [72].

Projections of poverty are computed through changes in the log-normal distribution of consumption. The parameterization of the log-normal distribution involves two steps. The first step computes the mean and standard deviation of the projected distribution using consumption, population, and Gini-index. Later, these parameters are used in a cumulative distribution function to compute the share of people at any consumption threshold. When consumption follows a log-normal distribution, with parameters *LNMean* and *LnStdDev*, we can use the expression for the first moment to obtain [73]:

$${MeanConsumption}_{r}=Exp\left({LNMean}_{r}+\frac{1}{2}{LNStDev}_{r}^{2}\right)$$

where *MeanConsumption_r_* is the ratio of household consumption at PPP to total population, and the index r represents regions or countries.

Rearranging the above, we write:

$${LNMean}_{r}=Ln({MeanConsumption}_{r}*{AdjustmentFactor}_{r,t=1}*1000)-.05*{LNStDev}_{r}^{2}$$

where AdjustmentFactor is a scaling factor calculated during the first year to account for differences between average consumption from national accounts (data) and average consumption used in the poverty calculation to ensure that these two data sources align.

As illustrated in Chotikapanich et al. [74], the standard deviation of a log-normal distribution can be used to compute the Gini-index of inequality. Taking an inverse we compute the standard deviation of the normal distribution from the Gini-index. Data measuring the Gini-index come from the World Bank World Development Indicators [69, 75]. Data for 2017 is used and if absent existing data from previous years is used. Remaining missing data points (17 countries in total) are filled in with a regression function driven by GDP per capita.

$${LNStDev}_{r}=\sqrt{2}*\Phi^{-1}(\frac{{GINI}_{r}+1}{2})$$

*where LNStDev is the standard deviation of the log normal distribution, Φ is the standard normal integral at* $\frac{{LNStDev}_{r}}{\surd 2}$, *and GINI is the Gini-index for income inequality*

Once we have the parameters of the log-normal distribution, we can use them in the cumulative distribution function (CDF). The CDF is used to calculate the share of people living below the poverty threshold of consumption, i.e., $1.90 per person per day, or, $693.5 per person per year. The population fraction living below poverty is multiplied with total population to get the poverty headcount.

$${INCOMELT190LN}_{r}=LogNormalCDF(PovertyThreshold,{LNMean}_{r},{LNStDev}_{r})*{POP}_{r}$$

where INCOMELT190LN is the number of people living on less than $1.90 per day at PPP, LogNormalCDF is the CDF, PovertyThreshold is the annualized income for a particular level of poverty, LNMean is the mean of the log normal distribution, LNStDev is the standard deviation of the log normal distribution, and POP is the population size.

### Modeling demographic change

The IFs demographic model uses an age-sex cohort structure and forecasts demographic changes based on changing patterns of fertility, mortality and migration [54]. Population data are initialized using the UN population division [76]. Data and projections on migration come from WIC/IIASA projections [77] in work for the shared socioeconomic pathways project [78]. The drivers of change in fertility rates include contraception use, infant mortality, GDP per capita, and average levels of education. The drivers of mortality are broad and documented in other publications [62].

### Comparison with other approaches

Several models aim to project extreme poverty over long time horizons. There are some important differences between the IFs methodology and other existing poverty projections. A major difference between IFs and other approaches is that most approaches use exogeneous growth rates to project income and/or consumption, whereas our approach aims to endogenously represent many processes with details on economic activities, agent decisions and accounting flows, making poverty reduction pathways more transparent. The choice to represent the world as an interconnected system and aiming to represent many of these interactions in itself represent a different modelling philosophy with advantages to more broadly representing patterns of economic and human development but arguably makes it more challenging to succinctly describe the full model.

In addition to these broader philosophical distinctions in modeling approaches, more concrete differences are also important to mention. First, previous research has used changes in average income (GDP per capita) to estimate poverty [3, 46]. The use of average income provides clear computational advantage, increases data availability, and allows for integration of the work with existing scenario frameworks such as the SSPs, but the downside of this approach is that extreme poverty is generally defined in terms of consumption rather than income. Some poverty models, for example [3, 10] address this by computing an adjustment factor to convert income to consumption. Here we also calculate poverty based on consumption, with the main differences being the use of the SAM to convert income to disposable income, and then using a ratio of income to consumption couple with population dynamics to arrive at final consumption.

A second difference relates to the choice of the distributional function. IFs uses a log-normal distribution to estimate extreme poverty given its computational advantages and applicability across different countries [13, 45, 79]. Other distributional forms include Beta-Lorentz Curves using the Gini-index [7]. An alternative to these Gini-based approaches, is to rather use the entire distribution, either by using microdata or binned data, from household surveys. These distributions can then be fitted using multiple distribution types such as Beta-Lorentz Curve or a generalized quadratic Lorentz Curve depending on fit to the data, allowing for greater flexibility in the functional form [10]. Growth projections in these models are applied to each of the micro-data and poverty is recomputed for the same cut-off. Some of these bottom-up models simulate distributional change through non-parametric techniques like the growth-incidence curves used by [10].

**Fig 2:**
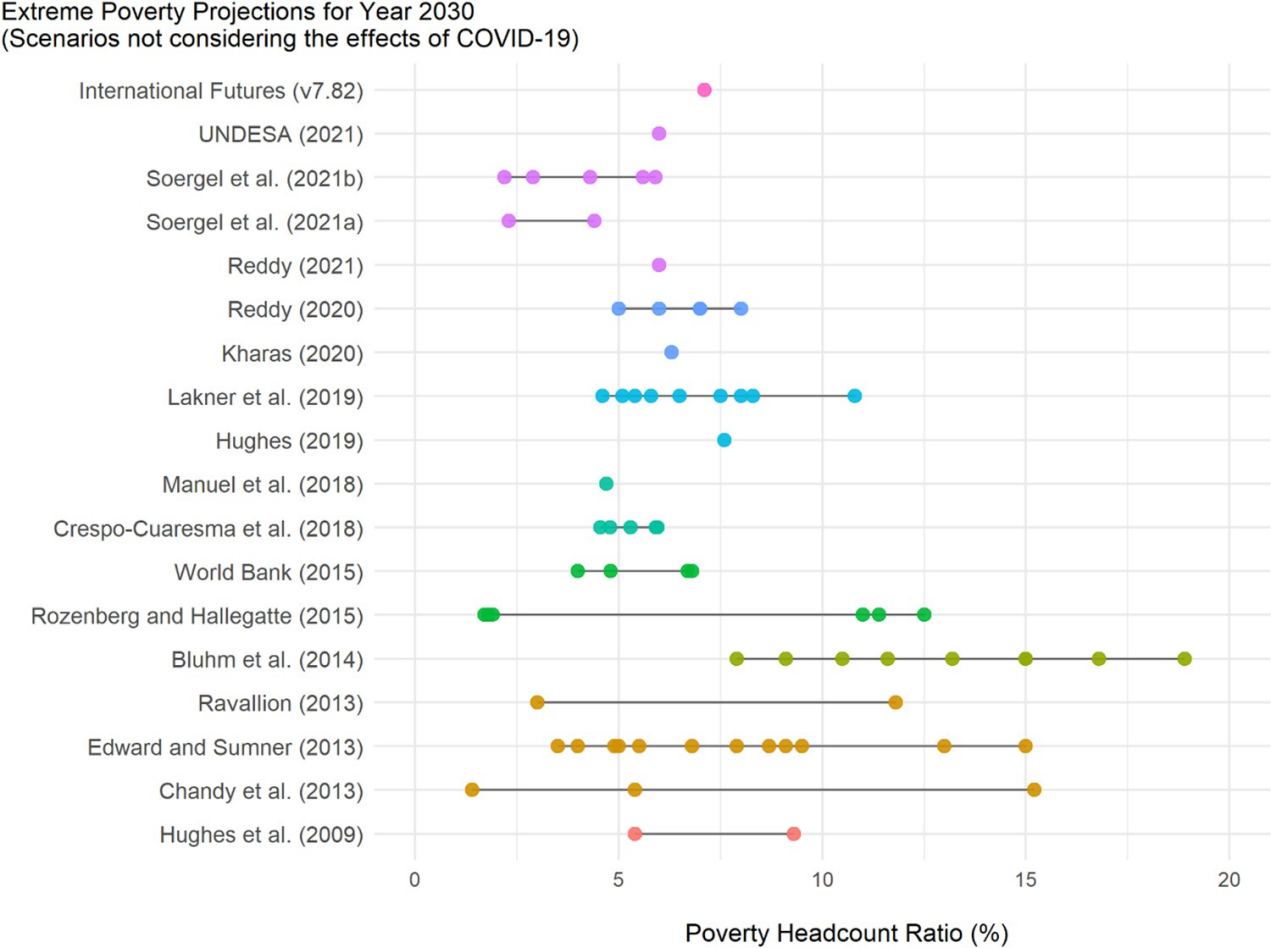
Previous projections of extreme poverty levels summary that do not account for COVID-19. Each point represents a scenario outcome value–point color reflects year published. Studies published in or before 2015 expressed using extreme poverty line of $1.25/day (USD2005). Hughes et al. [4] figures expressed using poverty line of $1.00/day (USD2005). Studies after 2015 use extreme poverty line of $1.90/day (USD2011). Different income thresholds are comparable because they are expressed using different real dollar thresholds that attempt to capture similar levels of PPP across time.

Fig 2 presents the IFs model projections and contextualizes them relative to other model results in the year 2030. As the model results presented here do not include COVID-19 impacts, we present the *No COVID Base* (International Futures version 7.82), which is described in a subsequent section. This scenario projects 7.1% of the global population to live in extreme poverty by the year 2030. This value is towards the middle of other published 2030 poverty projections (ranging from 1% to 19% poverty ratio across various methodologies and scenario assumptions) and towards the pessimistic side of more recently published studies [7, 53, 54, 80–83].

## Scenario assumptions

The analysis presented here begins with a *No COVID Base* scenario representing expected development patterns in a world without the pandemic. To simulate this we rely on economic growth rates produced just prior to the pandemic in the World Economic Outlook (WEO) [84]. We apply growth rates from this report for 2019–2025 and then use IFs endogenous growth projections through 2050. For this scenario we maintain 2017 country Gini-index values through 2050. As noted previously, this scenario produces similar results to other medium-variant forecasts prior to the outbreak (Fig 1). We compare this *No COVID Base* scenario with the *COVID Base* scenario. This scenario simulates the effect of COVID-19 by including WEO growth projections published for the years 2021–2023 [16]. From 2023–2050 we also rely on IFs endogenous growth projections for this scenario and keep country-level income inequality values flat across time.

We compare these two scenarios with six alternative scenarios that frame uncertainty by varying GDP growth and income inequality. We vary GDP growth by 1.5 percentage points for 2022 around the *COVID-Base* values and then converge these to the *COVID Base* growth trajectory by 2025. The 1.5 percentage points variation is a high-end assumption that falls within the standard variation across world GDP growth rates from the WEO during the COVID-19 period (~1.6%), and the mean difference across countries in GDP growth rates comparing the World Bank Global Economic Prospects [85] and the IMF WEO April 2021 release (~1.6%) [24].

After 2025 the alternative scenario growth calculations are a product of the IFs model described above. These scenarios also differ in their assumptions regarding inequality, which we vary by -2%, +2% and +5% relative to each country’s *COVID Base* value. This reflects literature described earlier that suggests that the pandemic will increase inequality but recognizes that this is highly uncertain. Thus, these additional six scenarios allow a sensitivity analysis of longer-term poverty futures around the *COVID Base* scenario. Their variation from that scenario therefore represent not just unknowns about the direct and indirect longer-term impacts of COVID, but also other uncertainties with respect to longer-term patterns of economic growth and change in distribution. Table 1 describes the scenario assumptions.

**Table 1:** Scenarios analyzed.

Scenario Name	Impact Area	Assumption
**No COVID Base**	**Growth**	World Bank GDP growth for 2017–2019 (WDI 2021), IMF Growth rate assumptions (IMF 2019) for 2020–23, IFs growth projections through 2050.
	**Inequality**	Gini-index set at 2019 values and kept constant.
**COVID Base**	**Growth**	World Bank GDP growth for 2017–2020 (WDI 2021), IMF Growth rate assumptions (IMF 2022) 2021–23, IFs growth projections through 2050.
	**Inequality**	Gini-index set at 2019 values and kept constant.
**High Growth, Very High Inequality**	**Growth**	World Bank GDP growth 2017–2020 (WDI 2021), IMF Growth rate assumptions (IMF 2022) 2021–23 plus 1.5 percentage points for 2022 that converge to IFs growth assumptions by 2025 growth projections through 2050.
	**Inequality**	An increase of 5% on the global Gini-index for household income in 2020 maintained through 2050.
**High Growth, High Inequality**	**Growth**	World Bank GDP growth 2017–2020 (WDI 2021), IMF Growth rate assumptions (IMF 2022) 2021–23 plus 1.5 percentage points for 2022 that converge to IFs growth assumptions by 2025 growth projections through 2050.
	**Inequality**	An increase of 2% on the global Gini-index for household income in 2020 maintained through 2050.
**High Growth, Low Inequality**	**Growth**	World Bank GDP growth 2017–2020 (WDI 2021), IMF Growth rate assumptions (IMF 2022) 2021–23 plus 1.5 percentage points for 2022 that converge to IFs growth assumptions by 2025 growth projections through 2050.
	**Inequality**	A decrease of 2% on the global Gini-index for household income in 2020 maintained through 2050.
**Low Growth, Very High Inequality**	**Growth**	World Bank GDP growth 2017–2020 (WDI 2021), IMF Growth rate assumptions (IMF 2022) 2021–23 minus 1.5 percentage points for 2022 that converge to IFs growth assumptions by 2025 growth projections through 2050.
	**Inequality**	An increase of 5% on the global Gini-index for household income in 2020 maintained through 2050.
**Low Growth, High Inequality**	**Growth**	World Bank GDP growth 2017–2020 (WDI 2021), IMF Growth rate assumptions (IMF 2022) 2021–23 minus 1.5 percentage points for 2022 that converge to IFs growth assumptions by 2025 growth projections through 2050.
	**Inequality**	An increase of 2% on the global Gini-index for household income in 2020 maintained through 2050.
**Low Growth, Low Inequality**	**Growth**	World Bank GDP growth 2017–2020 (WDI 2021), IMF Growth rate assumptions (IMF 2022) 2021–23 minus 1.5 percentage points for 2022 that converge to IFs growth assumptions by 2025 growth projections through 2050.
	**Inequality**	A decrease of 2% on the global Gini-index for household income in 2020 maintained through 2050.

## Results

COVID-19 has reduced global economic activity. Our simulation suggests that global GDP in the *COVID Base* is $5.7 trillion less than the *No COVID Base* in 2020 and $3.5 trillion less in 2021. Annually, the gap between these scenarios grows to $6.1 trillion in 2050, resulting in a 3.2% reduction in the *COVID Base* relative to the *No COVID Base*. Cumulatively through 2050 the pandemic is projected to have reduced economic output by $122.6 trillion dollars. Table 2 highlights the economic growth results across different scenarios.

**Table 2:** GDP (MER in $2011 USD) annual growth rate by decade and scenario.

	COVID Base	No COVID	Growth Low, Gini Low	Growth Low, Gini Very High	Growth Low, Gini High	Growth High, Gini Low	Growth High, Gini Very High	Growth High, Gini High
**2019**	2.43	2.43	2.43	2.43	2.43	2.43	2.43	2.43
**2020**	-3.43	2.71	-3.43	-3.43	-3.43	-3.43	-3.43	-3.43
**2021**	5.6	2.8	5.6	5.6	5.6	5.6	5.6	5.6
**2022**	4.10	2.78	2.60	2.60	2.60	5.60	5.60	5.60
**2030**	2.36	2.48	2.33	2.32	2.32	2.40	2.39	2.4
**2040**	2.27	2.32	2.26	2.26	2.26	2.29	2.28	2.29
**2050**	2.19	2.20	2.18	2.17	2.18	2.19	2.18	2.19

The scenarios that include high economic growth assumptions see global annual GDP levels return to the *No COVID Base* by 2023. While the volume of economic activity returns quickly, the reductions in economic output in 2020–2022 remain large, totaling $10.2 trillion. Scenarios with low growth assumptions result in reduced economic output relative to the *No COVID Base* by a cumulative $27.5 trillion by 2025, $57 trillion by 2030, and around $250 trillion by 2050. Across the low-growth scenarios GDP in 2050 is between 6.5% and 6.7% lower than in a world where the pandemic did not occur. By 2050, differences in population size are relatively minor across scenarios, with the largest difference (between the *No COVID Base* and the *Low Growth Very High Inequality* scenarios) totaling 15.0 million people, or 0.15% of the projected population in that year.

In 2019, IFs estimates that the global population living in extreme poverty to be 693.1 million with 1.79 billion people living on less than $3.20 per day. In the *No COVID Base*, the number of people living on less than $1.90 is projected to fall to 607.8 million by 2030 and 383.0 million by 2050 while those living on less than $3.20 is projected to fall to 1.5 billion by 2030 and 1.1 billion by 2050. In percentage terms, the share of the global population living on less than $1.90 per day was 9.0% in 2019 and projected to reach 7.1% in 2030 and 3.9% by 2050 while the share of the population living on less than $3.20 was 23.2% in 2019 and projected to decline to 18.0% by 2030 and 11.1% by 2050. Of the 186 countries analyzed here, 102 had already achieved the target value (of less than 3%) for SDG 1 by 2019 and an additional 14 countries were projected to achieve the goal by 2030 in a *No COVID* scenario.

The *COVID-19 Base* increases both the number of people and the share of the population living on less than $1.90 and $3.20 compared with the *No COVID Base*, from 2020–2050. In 2020 and 2021, an additional 73.9 and 86.8 million people are projected to live on less than $1.90 per day (0.95 and 1.1 percentage point increase in the share of the population) and 149.5 and 180.5 million people are projected to live on less than $3.20 per day (1.9 and 2.3 percentage point increase in the share of the population). Over the long-run the *COVID Base* increases the number of people living on less than $1.90 per day relative to the *No COVID Base* by 63.6 million in 2030 and 57.1 million in 2050 (0.7 and 0.6 percentage point increase in the share of the population), and the number of people living on less than $3.20 per day by 152.7 million in 2030 and 136.8 million in 2050 (1.8 and 1.4 percentage point increase in the share of the population).

The *Low Growth*, *High Inequality* scenario is the most pessimistic and projects an increase in extreme poverty of 163.0 million by 2050 relative to the *No COVID Base*. More optimistically, the *High Growth*, *Low Inequality* scenario estimates an increase of 56.5 million people in extreme poverty by 2021, 9.8 million by 2030, and 3.1 million by 2050, representing increases of 0.7 percentage points in 2021, 0.1 percentage points in 2030 and 0.03 percentage points by 2050 relative to the *No COVID Base*. See Table 3 for a summary of findings across scenarios for the global population living on less than $1.90 and $3.20 per day.

**Table 3:** Increase in global population living on less than $1.90 per day and $3.20 per day by scenario and time relative to the No COVID Base scenario, millions of people.

$1.90	COVID Base	Low Growth, Low Inequality	Low Growth, Very High Inequality	Low Growth, High Inequality	High Growth, Low Inequality	High Growth, Very High Inequality	High Growth, High Inequality
**2020**	73.9	43.5	155.0	105.5	43.5	155.0	105.5
**2021**	86.8	56.5	167.4	118.2	56.5	167.4	118.2
**2022**	90.7	75.3	186.1	136.9	45.2	154.2	105.7
**2030**	63.6	62.6	167.2	120.5	9.8	110.6	65.6
**2040**	62.1	64.9	173.4	125.1	2.8	106.7	60.4
**2050**	57.1	59.3	163.0	116.6	3.1	100.2	56.7
**$3.20**							
**2020**	149.5	112.5	245.3	187.2	112.5	245.3	187.2
**2021**	180.5	143.5	276.4	218.3	143.5	276.4	218.3
**2022**	200.0	188.6	322.7	264.1	133.6	268.0	209.3
**2030**	152.7	164.6	312.1	247.8	59.17	206.3	142.2
**2040**	155.6	173.6	339.1	267.1	45.84	210.4	138.8
**2050**	136.8	154.9	332.5	254.9	25.56	198.5	122.7

In the short-run, the largest increase in extreme poverty headcount due to COVID-19 occurs in South Asia and sub-Saharan Africa. In the long-run the region most likely to see the greatest COVID-related increases in extreme poverty in absolute terms is sub-Saharan Africa. Headcount increases in extreme poverty driven by COVID-19 in South Asia range from 29.8 to 73.3 million in 2021, 7.0 to 49.9 million in 2030, and 0.6 to 19.9 million by 2050. In Sub-Saharan Africa, increases in extreme poverty range from 17.4 to 49.3 million in 2021, -3.6 to 70.5 million in 2030, and -2.0 to 108.6 million in 2050 compared with a *No COVID Base*.

**Fig 3:**
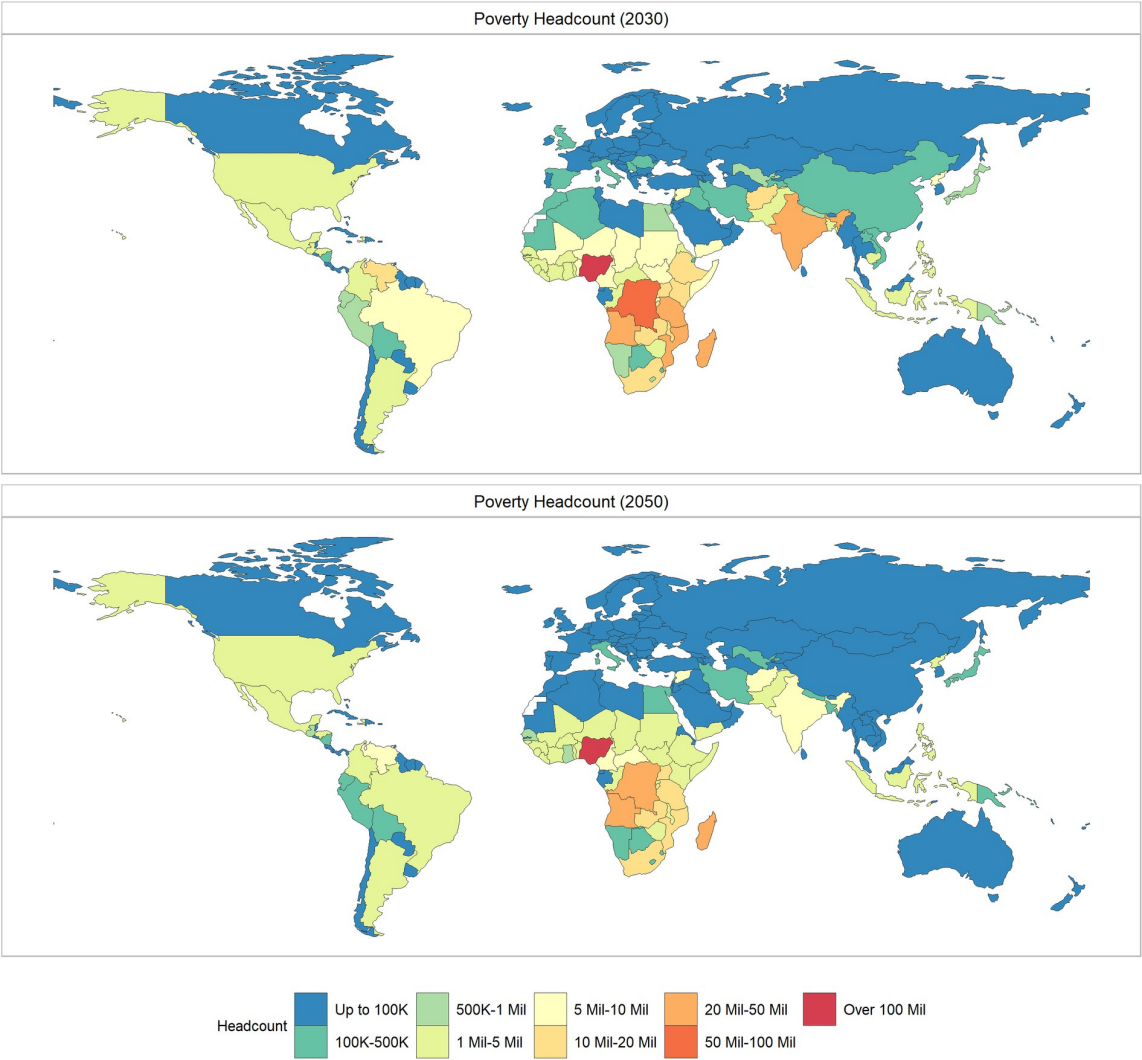
Absolute level of extreme poverty by country in a No COVID scenario. The top panel shows the results for 2030, the bottom panel shows 2050. Source: Author’s computation. Shapefiles for map sourced from the NaturalEarth project (naturalearthdata.com).

**Fig 4:**
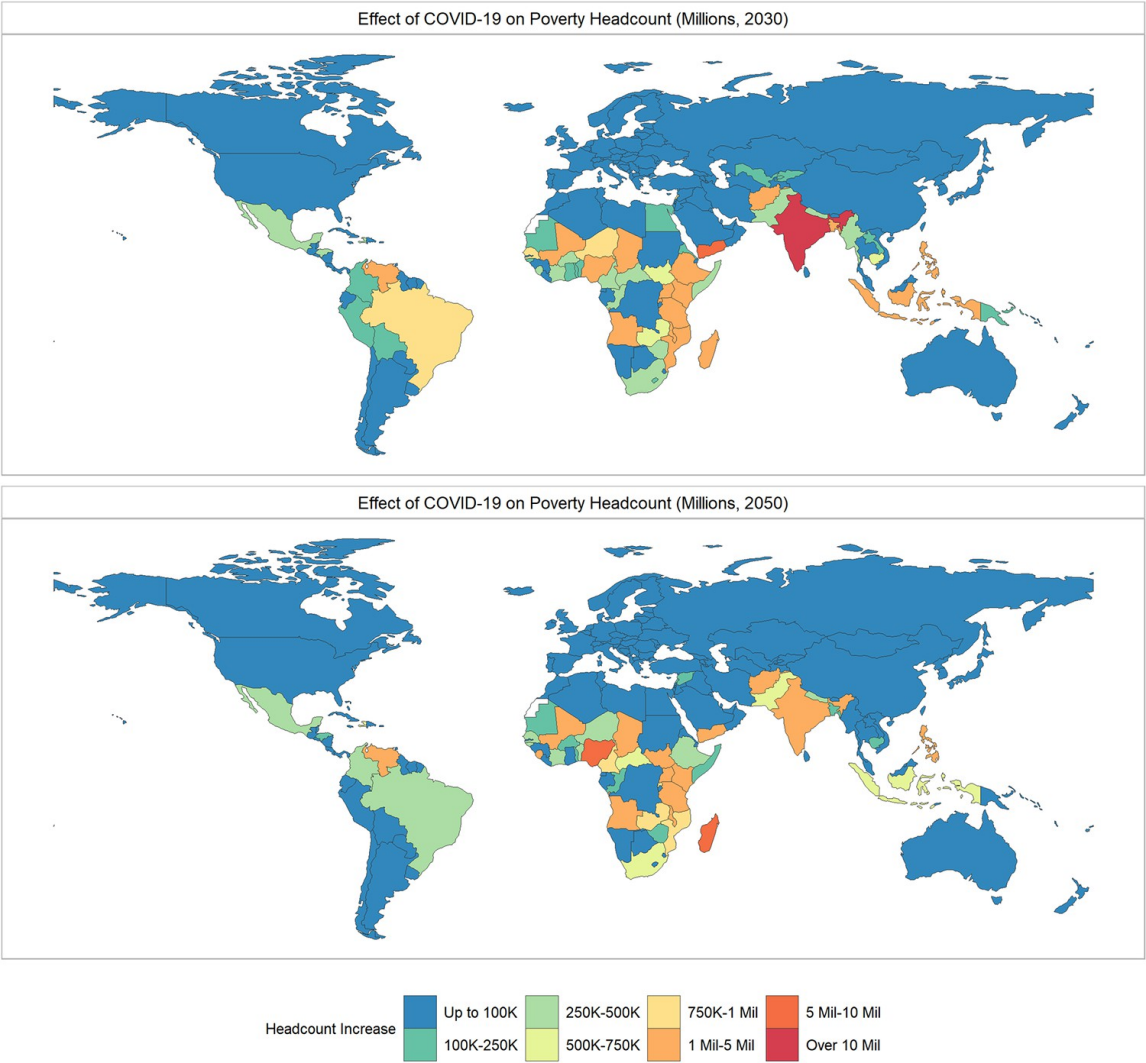
Absolute increate in extreme poverty by country due to COVID-19. The top panel shows the results for a comparison of the No COVID Base and the COVID-Base for 2030, the bottom panel shows the same comparison for 2050. Source: Author’s computation. Shapefiles for map sourced from the NaturalEarth project (naturalearthdata.com).

In 2021, the countries that experience the largest increase in the number of people living in extreme poverty are geographically diverse, with the greatest projected increase in India (an additional 36.6 million people), followed by Yemen (4.7 million), Ethiopia (3.3 million), Nigeria (3.1 million), and the Philippines (2.3 million). The countries with the largest share of the population projected to be pushed into $1.90 poverty in 2021 are Yemen (15.4%), Rwanda (7.9%), Lebanon (7.2%), Cambodia (6.5%) and Venezuela (6.1%). Fig 3 tracks country level changes in extreme poverty headcount in 2030 and 2050 comparing *COVID Base* and *No COVID Base* scenarios and Fig 4 shows the absolute increase in extreme poverty driven by the global pandemic.

We analyzed the impact of COVID-19 on the achievement of SDG1, which targets an elimination of extreme poverty (often operationalized as bringing rates below 3% of each country’s population by 2030). In the *No COVID Base* 70 out of 186 countries do not achieve the 3% threshold by 2030. In the *COVID Base* this number increases to 77 countries. However, in a worst-case scenario, the number of countries that do not achieve SDG1 increases to 87.

## Discussion

This paper has highlighted the high costs for core aspects of human material well-being that can be associated with changing patterns of economic production and its distribution. Any modelling exercise has methodological limitations, and this is even more true for an analysis of the impacts on human development of an evolving and continuously changing pandemic.

First, there are challenges in assessing the immediate impacts of the pandemic. Similar to other COVID impact assessments [9, 10, 50], this analysis estimated the COVID-19 impact on GDP as the difference between growth rates anticipated in 2020 and later years anticipated in 2019 prior to the pandemic and those that have subsequently been achieved. The resulting differences in GDP growth between these two series can be primarily attributed to COVID-19 but obviously also include other impacts on GDP growth.

A second limitation is the lack of sectoral differentiation of COVID’s economic growth impact. Here, largely for reasons of current data and analysis availability, we looked at overall GDP growth without accounting for how these differences play out across different economic sectors (but see [39]).

A third limitation is the large uncertainty associated with the future of the pandemic, the rise of new variants and consequential vastly different growth and inequality trajectories. The analysis presented here frames part of this uncertainty by using a total of seven COVID-scenarios. With the emergence of more data and better understanding of the economic and human development costs of COVID-19, future analysis could explore its poverty impacts in more detail, and even support scenarios that simulate various patterns of pandemic resurgence over time.

Looking forward, research should allow a more structurally sophisticated analysis of the implications of shocks like the COVID-19 pandemic on human development. For instance, inevitable future shocks will come from a variety of sources, including unexpected intra- and inter-state conflict [86], economic recessions, natural disasters, future health emergencies and other unanticipated change [87]. To better prepare for analysis of disruptions, models should be structured to better represent both short-term shocks as well as longer-term structural transformations. In addition to and supporting examination of differential impact across economic sectors, modeling should represent the broader socio-political and demographic dynamics of the unfolding shocks. Toward this end, progress is being made in model-based approaches that link impacts of conflict and of climate change to long-term human development indicators [88–91].

Furthering our ability to model such structural dynamics will allow for identification of resilient policies that improve humans’ ability to adapt and thrive. In addition to integrating shock analysis into our modeling, policy-analysis research must focus on trade-offs and synergies in exploring developmental policy, including pathways in the pursuit of sustainable development broadly [56, 92]. The impact of COVID-19 on human development isn’t limited to direct, proximate drivers of poverty, on GDP growth or inequality. It includes effects on education, government debt and finance, international financial flows and trade, undernourishment, child stunting, and broad SDG achievement [17–19, 60, 93, 94]. Combinations of impacts will jointly shape the future impact of COVID-19 on extreme poverty, and more broadly human development. Prior to the pandemic, the world was not on track to meeting SDG goals [7, 8], and COVID-19 further complicates reaching these [19]. Additional efforts to accelerate progress on human and environmental sustainability (including climate change) are partly synergistic, but will also need to navigate trade-offs, stemming from limited resources and budgets.

Quantitative tools can be useful resources to enhance how we think about these integrated, complex issues. Integrated assessment tools can be a useful resource to frame how sustainable development systems can unfold, and they should continue to be embedded into conversations about policy strategies. While these tools can be helpful, they are also not panaceas, and mixed research methods that combine qualitative and quantitative approaches cutting across levels of analysis as well as disciplinary boundaries should be the focus of future work.

## Conclusion

The pandemic has hurt our ability to achieve the SDGs, leading to increased suffering among the poor and most vulnerable around the world. While the COVID-19 pandemic will not prevent the world from making progress toward eliminating extreme poverty over the coming decades, it will both disrupt progress during the pandemic period and shift the trajectory of ongoing advancements. We show that the rise in poverty from COVID-19 is potentially long-lasting with, in the absence of policy changes, higher poverty levels out to 2030 and 2050 than we would otherwise have anticipated.

Prior to the pandemic, many countries were not on track to meet the SDGs within the 2030 horizon period [8]. Here we show that the ambition to eradicate poverty globally by 2030 is projected to have moved further out of reach for many countries, and that the negative effects of COVID-19 will primarily be felt in countries and world regions already struggling with high levels of poverty. Even more troublesome is that poverty is only one of the SDG indicators projected to be negatively affected by COVID-19 over the long-term, while losses in education, growth in food insecurity and increasing levels of child mortality in today’s most vulnerable regions and populations are also expected to occur [17–19, 50, 60, 95]. The real challenge for academics and policy makers will be to come up with integrated, innovative, and inclusive policy-based solutions to realize the potential of the SDGs and minimize the long-term setback of COVID-19 on poverty and other development indicators.

In the most optimistic scenario of this study, poverty projections do not differ significantly from a *No COVID Base* in the long-term, but this requires a two percent improvement in the distribution of income and strong economic growth in the recovery period. This will also likely require aggressive policy around environmental and other commons-related challenges (such as accelerated climatic change) that might be associated with increased economic growth; however, these issues fall outside the scope of this study. There is still room for optimism, but it requires prioritizing policies that both encourage economic growth as well as improve resource distribution. The aim of these policies should be to not only realize a rapid recovery coupled with equality in the near term, but to build resilient populations and resilient countries that can deal with future shocks, be they economic, health or environment related.

## Supporting information

S1 Appendix (DOCX)

S1 File (PDF)
