# Supplementary material for: How many people is the COVID-19 pandemic pushing into poverty? A long-term forecast to 2050 with alternative scenarios
Source: PLoS One. 2022 Jul 8;17(7):e0270846. doi: 10.1371/journal.pone.0270846 (PMC9269768; doi:10.1371/journal.pone.0270846)
Supplement: S1 Appendix — (DOCX) [file pone.0270846.s001.docx]

# **Appendix**

The tables below show model results across scenarios for economic growth, the number of people living on less than $1.90 per day in millions and the share of the population living on less than $1.90 per day for the years 2020, 2021, 2022, 2030 and 2050.

**Table 4. Model results for the real GDP growth rate by scenario and selected years.**

| **Country** | **Scenario** | **2020** | **2021** | **2022** | **2030** | **2050** |
| --- | --- | --- | --- | --- | --- | --- |
| **Afghanistan** | **GDPrGiniBase** | -2.351 | 4 | 4.524 | 5.037 | 6.362 |
| **Afghanistan** | **GDPrHGiniH** | -2.351 | 4 | 6.024 | 5.173 | 6.409 |
| **Afghanistan** | **GDPrHGiniHv** | -2.351 | 4 | 6.024 | 5.157 | 6.401 |
| **Afghanistan** | **GDPrHGiniLow** | -2.351 | 4 | 6.024 | 5.191 | 6.419 |
| **Afghanistan** | **GDPrLGiniH** | -2.351 | 4 | 3.024 | 4.89 | 6.278 |
| **Afghanistan** | **GDPrLGiniHv** | -2.351 | 4 | 3.024 | 4.875 | 6.269 |
| **Afghanistan** | **GDPrLGiniLow** | -2.351 | 4 | 3.024 | 4.91 | 6.292 |
| **Afghanistan** | **NoCOVIDginiB** | 3.478 | 4 | 4.5 | 5.433 | 6.395 |
| **Albania** | **GDPrGiniBase** | -3.955 | 5.3 | 4.5 | 2.733 | 3.621 |
| **Albania** | **GDPrHGiniH** | -3.955 | 5.3 | 6 | 2.767 | 3.635 |
| **Albania** | **GDPrHGiniHv** | -3.955 | 5.3 | 6 | 2.763 | 3.633 |
| **Albania** | **GDPrHGiniLow** | -3.955 | 5.3 | 6 | 2.771 | 3.636 |
| **Albania** | **GDPrLGiniH** | -3.955 | 5.3 | 3 | 2.696 | 3.601 |
| **Albania** | **GDPrLGiniHv** | -3.955 | 5.3 | 3 | 2.692 | 3.599 |
| **Albania** | **GDPrLGiniLow** | -3.955 | 5.3 | 3 | 2.699 | 3.602 |
| **Albania** | **NoCOVIDginiB** | 4.022 | 3.989 | 4.016 | 2.896 | 3.626 |
| **Algeria** | **GDPrGiniBase** | -5.1 | 3.41 | 1.947 | 2.183 | 2.329 |
| **Algeria** | **GDPrHGiniH** | -5.1 | 3.41 | 3.447 | 2.223 | 2.439 |
| **Algeria** | **GDPrHGiniHv** | -5.1 | 3.41 | 3.447 | 2.221 | 2.438 |
| **Algeria** | **GDPrHGiniLow** | -5.1 | 3.41 | 3.447 | 2.225 | 2.441 |
| **Algeria** | **GDPrLGiniH** | -5.1 | 3.41 | 0.447 | 2.155 | 2.252 |
| **Algeria** | **GDPrLGiniHv** | -5.1 | 3.41 | 0.447 | 2.153 | 2.25 |
| **Algeria** | **GDPrLGiniLow** | -5.1 | 3.41 | 0.447 | 2.158 | 2.254 |
| **Algeria** | **NoCOVIDginiB** | 2.406 | 1.754 | 1.447 | 2.254 | 2.451 |
| **Angola** | **GDPrGiniBase** | -5.4 | -0.655 | 2.364 | 4.279 | 5.495 |
| **Angola** | **GDPrHGiniH** | -5.4 | -0.655 | 3.864 | 4.351 | 5.446 |
| **Angola** | **GDPrHGiniHv** | -5.4 | -0.655 | 3.864 | 4.34 | 5.441 |
| **Angola** | **GDPrHGiniLow** | -5.4 | -0.655 | 3.864 | 4.366 | 5.454 |
| **Angola** | **GDPrLGiniH** | -5.4 | -0.655 | 0.864 | 4.182 | 5.487 |
| **Angola** | **GDPrLGiniHv** | -5.4 | -0.655 | 0.864 | 4.171 | 5.479 |
| **Angola** | **GDPrLGiniLow** | -5.4 | -0.655 | 0.864 | 4.196 | 5.498 |
| **Angola** | **NoCOVIDginiB** | 1.153 | 2.874 | 3.328 | 4.927 | 5.394 |
| **Argentina** | **GDPrGiniBase** | -9.895 | 10 | 3 | 1.703 | 1.493 |
| **Argentina** | **GDPrHGiniH** | -9.895 | 10 | 4.5 | 1.73 | 1.495 |
| **Argentina** | **GDPrHGiniHv** | -9.895 | 10 | 4.5 | 1.725 | 1.495 |
| **Argentina** | **GDPrHGiniLow** | -9.895 | 10 | 4.5 | 1.735 | 1.495 |
| **Argentina** | **GDPrLGiniH** | -9.895 | 10 | 1.5 | 1.675 | 1.492 |
| **Argentina** | **GDPrLGiniHv** | -9.895 | 10 | 1.5 | 1.67 | 1.492 |
| **Argentina** | **GDPrLGiniLow** | -9.895 | 10 | 1.5 | 1.68 | 1.492 |
| **Argentina** | **NoCOVIDginiB** | -1.281 | 1.433 | 2.283 | 1.742 | 1.485 |
| **Armenia** | **GDPrGiniBase** | -7.4 | 6.461 | 4.543 | 3.242 | 2.562 |
| **Armenia** | **GDPrHGiniH** | -7.4 | 6.461 | 6.043 | 3.289 | 2.561 |
| **Armenia** | **GDPrHGiniHv** | -7.4 | 6.461 | 6.043 | 3.284 | 2.558 |
| **Armenia** | **GDPrHGiniLow** | -7.4 | 6.461 | 6.043 | 3.294 | 2.564 |
| **Armenia** | **GDPrLGiniH** | -7.4 | 6.461 | 3.043 | 3.186 | 2.552 |
| **Armenia** | **GDPrLGiniHv** | -7.4 | 6.461 | 3.043 | 3.181 | 2.549 |
| **Armenia** | **GDPrLGiniLow** | -7.4 | 6.461 | 3.043 | 3.192 | 2.555 |
| **Armenia** | **NoCOVIDginiB** | 4.8 | 4.5 | 4.5 | 3.475 | 2.571 |
| **Australia** | **GDPrGiniBase** | -0.004 | 4.2 | 4.1 | 2.117 | 1.552 |
| **Australia** | **GDPrHGiniH** | -0.004 | 4.2 | 5.6 | 2.113 | 1.549 |
| **Australia** | **GDPrHGiniHv** | -0.004 | 4.2 | 5.6 | 2.111 | 1.551 |
| **Australia** | **GDPrHGiniLow** | -0.004 | 4.2 | 5.6 | 2.115 | 1.547 |
| **Australia** | **GDPrLGiniH** | -0.004 | 4.2 | 2.6 | 2.101 | 1.564 |
| **Australia** | **GDPrLGiniHv** | -0.004 | 4.2 | 2.6 | 2.099 | 1.564 |
| **Australia** | **GDPrLGiniLow** | -0.004 | 4.2 | 2.6 | 2.104 | 1.561 |
| **Australia** | **NoCOVIDginiB** | 2.263 | 2.582 | 2.699 | 2.097 | 1.548 |
| **Austria** | **GDPrGiniBase** | -6.735 | 3.918 | 4.5 | 0.098 | 0.481 |
| **Austria** | **GDPrHGiniH** | -6.735 | 3.918 | 6 | 0.114 | 0.535 |
| **Austria** | **GDPrHGiniHv** | -6.735 | 3.918 | 6 | 0.112 | 0.535 |
| **Austria** | **GDPrHGiniLow** | -6.735 | 3.918 | 6 | 0.116 | 0.536 |
| **Austria** | **GDPrLGiniH** | -6.735 | 3.918 | 3 | 0.08 | 0.426 |
| **Austria** | **GDPrLGiniHv** | -6.735 | 3.918 | 3 | 0.078 | 0.425 |
| **Austria** | **GDPrLGiniLow** | -6.735 | 3.918 | 3 | 0.081 | 0.426 |
| **Austria** | **NoCOVIDginiB** | 1.7 | 1.55 | 1.55 | 0.128 | 0.475 |
| **Azerbaijan** | **GDPrGiniBase** | -4.3 | 2.959 | 2.349 | 3.614 | 2.29 |
| **Azerbaijan** | **GDPrHGiniH** | -4.3 | 2.959 | 3.849 | 3.588 | 2.313 |
| **Azerbaijan** | **GDPrHGiniHv** | -4.3 | 2.959 | 3.849 | 3.582 | 2.261 |
| **Azerbaijan** | **GDPrHGiniLow** | -4.3 | 2.959 | 3.849 | 3.596 | 2.331 |
| **Azerbaijan** | **GDPrLGiniH** | -4.3 | 2.959 | 0.849 | 3.639 | 2.276 |
| **Azerbaijan** | **GDPrLGiniHv** | -4.3 | 2.959 | 0.849 | 3.634 | 2.264 |
| **Azerbaijan** | **GDPrLGiniLow** | -4.3 | 2.959 | 0.849 | 3.648 | 2.294 |
| **Azerbaijan** | **NoCOVIDginiB** | 2.11 | 2.145 | 2.214 | 3.779 | 2.33 |
| **Bahamas** | **GDPrGiniBase** | -14.51 | 2 | 7.996 | 0.52 | 0.582 |
| **Bahamas** | **GDPrHGiniH** | -14.51 | 2 | 9.496 | 0.583 | 0.605 |
| **Bahamas** | **GDPrHGiniHv** | -14.51 | 2 | 9.496 | 0.573 | 0.602 |
| **Bahamas** | **GDPrHGiniLow** | -14.51 | 2 | 9.496 | 0.596 | 0.607 |
| **Bahamas** | **GDPrLGiniH** | -14.51 | 2 | 6.496 | 0.449 | 0.554 |
| **Bahamas** | **GDPrLGiniHv** | -14.51 | 2 | 6.496 | 0.437 | 0.552 |
| **Bahamas** | **GDPrLGiniLow** | -14.51 | 2 | 6.496 | 0.462 | 0.559 |
| **Bahamas** | **NoCOVIDginiB** | -0.557 | 2.101 | 1.732 | 0.663 | 0.637 |
| **Bahrain** | **GDPrGiniBase** | -5.085 | 2.441 | 3.097 | 3.188 | 0.836 |
| **Bahrain** | **GDPrHGiniH** | -5.085 | 2.441 | 4.597 | 3.228 | 0.862 |
| **Bahrain** | **GDPrHGiniHv** | -5.085 | 2.441 | 4.597 | 3.226 | 0.862 |
| **Bahrain** | **GDPrHGiniLow** | -5.085 | 2.441 | 4.597 | 3.232 | 0.862 |
| **Bahrain** | **GDPrLGiniH** | -5.085 | 2.441 | 1.597 | 3.152 | 0.821 |
| **Bahrain** | **GDPrLGiniHv** | -5.085 | 2.441 | 1.597 | 3.15 | 0.82 |
| **Bahrain** | **GDPrLGiniLow** | -5.085 | 2.441 | 1.597 | 3.156 | 0.821 |
| **Bahrain** | **NoCOVIDginiB** | 2.096 | 2.352 | 2.445 | 3.305 | 0.828 |
| **Bangladesh** | **GDPrGiniBase** | 3.509 | 4.599 | 6.544 | 5.275 | 4.759 |
| **Bangladesh** | **GDPrHGiniH** | 3.509 | 4.599 | 8.044 | 5.354 | 4.765 |
| **Bangladesh** | **GDPrHGiniHv** | 3.509 | 4.599 | 8.044 | 5.35 | 4.766 |
| **Bangladesh** | **GDPrHGiniLow** | 3.509 | 4.599 | 8.044 | 5.36 | 4.764 |
| **Bangladesh** | **GDPrLGiniH** | 3.509 | 4.599 | 5.044 | 5.184 | 4.754 |
| **Bangladesh** | **GDPrLGiniHv** | 3.509 | 4.599 | 5.044 | 5.173 | 4.754 |
| **Bangladesh** | **GDPrLGiniLow** | 3.509 | 4.599 | 5.044 | 5.201 | 4.753 |
| **Bangladesh** | **NoCOVIDginiB** | 7.445 | 7.3 | 7.3 | 5.548 | 4.775 |
| **Barbados** | **GDPrGiniBase** | -18.98 | 3.3 | 8.5 | 1.349 | 2.185 |
| **Barbados** | **GDPrHGiniH** | -18.98 | 3.3 | 10 | 1.368 | 2.18 |
| **Barbados** | **GDPrHGiniHv** | -18.98 | 3.3 | 10 | 1.36 | 2.181 |
| **Barbados** | **GDPrHGiniLow** | -18.98 | 3.3 | 10 | 1.376 | 2.179 |
| **Barbados** | **GDPrLGiniH** | -18.98 | 3.3 | 7 | 1.325 | 2.202 |
| **Barbados** | **GDPrLGiniHv** | -18.98 | 3.3 | 7 | 1.319 | 2.203 |
| **Barbados** | **GDPrLGiniLow** | -18.98 | 3.3 | 7 | 1.332 | 2.201 |
| **Barbados** | **NoCOVIDginiB** | 0.6 | 1.5 | 1.841 | 1.471 | 2.35 |
| **Belarus** | **GDPrGiniBase** | -0.9 | 2.071 | 0.512 | 1.729 | 1.74 |
| **Belarus** | **GDPrHGiniH** | -0.9 | 2.071 | 2.012 | 1.782 | 1.758 |
| **Belarus** | **GDPrHGiniHv** | -0.9 | 2.071 | 2.012 | 1.776 | 1.754 |
| **Belarus** | **GDPrHGiniLow** | -0.9 | 2.071 | 2.012 | 1.789 | 1.763 |
| **Belarus** | **GDPrLGiniH** | -0.9 | 2.071 | -0.988 | 1.673 | 1.709 |
| **Belarus** | **GDPrLGiniHv** | -0.9 | 2.071 | -0.988 | 1.668 | 1.705 |
| **Belarus** | **GDPrLGiniLow** | -0.9 | 2.071 | -0.988 | 1.68 | 1.714 |
| **Belarus** | **NoCOVIDginiB** | 0.305 | 0.105 | 0.061 | 1.714 | 1.713 |
| **Belgium** | **GDPrGiniBase** | -5.66 | 5.636 | 3.107 | 1.194 | 1.168 |
| **Belgium** | **GDPrHGiniH** | -5.66 | 5.636 | 4.607 | 1.246 | 1.156 |
| **Belgium** | **GDPrHGiniHv** | -5.66 | 5.636 | 4.607 | 1.244 | 1.156 |
| **Belgium** | **GDPrHGiniLow** | -5.66 | 5.636 | 4.607 | 1.248 | 1.154 |
| **Belgium** | **GDPrLGiniH** | -5.66 | 5.636 | 1.607 | 1.154 | 1.179 |
| **Belgium** | **GDPrLGiniHv** | -5.66 | 5.636 | 1.607 | 1.152 | 1.179 |
| **Belgium** | **GDPrLGiniLow** | -5.66 | 5.636 | 1.607 | 1.156 | 1.18 |
| **Belgium** | **NoCOVIDginiB** | 1.3 | 1.305 | 1.319 | 1.157 | 1.15 |
| **Belize** | **GDPrGiniBase** | -14.01 | 8.5 | 5.4 | 3.67 | 3.81 |
| **Belize** | **GDPrHGiniH** | -14.01 | 8.5 | 6.9 | 3.729 | 3.714 |
| **Belize** | **GDPrHGiniHv** | -14.01 | 8.5 | 6.9 | 3.715 | 3.711 |
| **Belize** | **GDPrHGiniLow** | -14.01 | 8.5 | 6.9 | 3.748 | 3.717 |
| **Belize** | **GDPrLGiniH** | -14.01 | 8.5 | 3.9 | 3.595 | 3.843 |
| **Belize** | **GDPrLGiniHv** | -14.01 | 8.5 | 3.9 | 3.579 | 3.842 |
| **Belize** | **GDPrLGiniLow** | -14.01 | 8.5 | 3.9 | 3.613 | 3.845 |
| **Belize** | **NoCOVIDginiB** | 2.094 | 1.789 | 1.695 | 3.873 | 3.856 |
| **Benin** | **GDPrGiniBase** | 3.849 | 5.511 | 6.531 | 6.153 | 8.232 |
| **Benin** | **GDPrHGiniH** | 3.849 | 5.511 | 8.031 | 6.255 | 8.212 |
| **Benin** | **GDPrHGiniHv** | 3.849 | 5.511 | 8.031 | 6.23 | 8.196 |
| **Benin** | **GDPrHGiniLow** | 3.849 | 5.511 | 8.031 | 6.288 | 8.231 |
| **Benin** | **GDPrLGiniH** | 3.849 | 5.511 | 5.031 | 6.019 | 8.22 |
| **Benin** | **GDPrLGiniHv** | 3.849 | 5.511 | 5.031 | 5.997 | 8.198 |
| **Benin** | **GDPrLGiniLow** | 3.849 | 5.511 | 5.031 | 6.049 | 8.247 |
| **Benin** | **NoCOVIDginiB** | 6.701 | 6.742 | 6.744 | 6.388 | 8.198 |
| **Bhutan** | **GDPrGiniBase** | -10.08 | -1.916 | 4.198 | 2.664 | 2.722 |
| **Bhutan** | **GDPrHGiniH** | -10.08 | -1.916 | 5.698 | 2.773 | 2.726 |
| **Bhutan** | **GDPrHGiniHv** | -10.08 | -1.916 | 5.698 | 2.768 | 2.718 |
| **Bhutan** | **GDPrHGiniLow** | -10.08 | -1.916 | 5.698 | 2.78 | 2.736 |
| **Bhutan** | **GDPrLGiniH** | -10.08 | -1.916 | 2.698 | 2.537 | 2.689 |
| **Bhutan** | **GDPrLGiniHv** | -10.08 | -1.916 | 2.698 | 2.532 | 2.681 |
| **Bhutan** | **GDPrLGiniLow** | -10.08 | -1.916 | 2.698 | 2.545 | 2.699 |
| **Bhutan** | **NoCOVIDginiB** | 7.211 | 5.913 | 6.905 | 3.413 | 2.644 |
| **Bolivia** | **GDPrGiniBase** | -8.83 | 4.95 | 4 | 3.77 | 4.777 |
| **Bolivia** | **GDPrHGiniH** | -8.83 | 4.95 | 5.5 | 3.91 | 4.749 |
| **Bolivia** | **GDPrHGiniHv** | -8.83 | 4.95 | 5.5 | 3.903 | 4.748 |
| **Bolivia** | **GDPrHGiniLow** | -8.83 | 4.95 | 5.5 | 3.919 | 4.75 |
| **Bolivia** | **GDPrLGiniH** | -8.83 | 4.95 | 2.5 | 3.711 | 4.799 |
| **Bolivia** | **GDPrLGiniHv** | -8.83 | 4.95 | 2.5 | 3.705 | 4.797 |
| **Bolivia** | **GDPrLGiniLow** | -8.83 | 4.95 | 2.5 | 3.72 | 4.8 |
| **Bolivia** | **NoCOVIDginiB** | 3.8 | 3.7 | 3.7 | 4.123 | 4.697 |
| **Bosnia and Herzegovina** | **GDPrGiniBase** | -3.197 | 2.8 | 3.25 | 2.614 | 2.487 |
| **Bosnia and Herzegovina** | **GDPrHGiniH** | -3.197 | 2.8 | 4.75 | 2.633 | 2.472 |
| **Bosnia and Herzegovina** | **GDPrHGiniHv** | -3.197 | 2.8 | 4.75 | 2.63 | 2.472 |
| **Bosnia and Herzegovina** | **GDPrHGiniLow** | -3.197 | 2.8 | 4.75 | 2.637 | 2.473 |
| **Bosnia and Herzegovina** | **GDPrLGiniH** | -3.197 | 2.8 | 1.75 | 2.572 | 2.48 |
| **Bosnia and Herzegovina** | **GDPrLGiniHv** | -3.197 | 2.8 | 1.75 | 2.568 | 2.48 |
| **Bosnia and Herzegovina** | **GDPrLGiniLow** | -3.197 | 2.8 | 1.75 | 2.576 | 2.481 |
| **Bosnia and Herzegovina** | **NoCOVIDginiB** | 2.6 | 2.6 | 2.8 | 2.782 | 2.468 |
| **Botswana** | **GDPrGiniBase** | -8.493 | 9.162 | 4.68 | 4.32 | 4.108 |
| **Botswana** | **GDPrHGiniH** | -8.493 | 9.162 | 6.18 | 4.382 | 4.099 |
| **Botswana** | **GDPrHGiniHv** | -8.493 | 9.162 | 6.18 | 4.371 | 4.097 |
| **Botswana** | **GDPrHGiniLow** | -8.493 | 9.162 | 6.18 | 4.397 | 4.1 |
| **Botswana** | **GDPrLGiniH** | -8.493 | 9.162 | 3.18 | 4.247 | 4.115 |
| **Botswana** | **GDPrLGiniHv** | -8.493 | 9.162 | 3.18 | 4.236 | 4.113 |
| **Botswana** | **GDPrLGiniLow** | -8.493 | 9.162 | 3.18 | 4.262 | 4.116 |
| **Botswana** | **NoCOVIDginiB** | 4.339 | 5.906 | 3.851 | 4.55 | 4.082 |
| **Brazil** | **GDPrGiniBase** | -4.059 | 4.7 | 0.3 | 1.597 | 1.094 |
| **Brazil** | **GDPrHGiniH** | -4.059 | 4.7 | 1.8 | 1.625 | 1.083 |
| **Brazil** | **GDPrHGiniHv** | -4.059 | 4.7 | 1.8 | 1.609 | 1.083 |
| **Brazil** | **GDPrHGiniLow** | -4.059 | 4.7 | 1.8 | 1.644 | 1.083 |
| **Brazil** | **GDPrLGiniH** | -4.059 | 4.7 | -1.2 | 1.553 | 1.107 |
| **Brazil** | **GDPrLGiniHv** | -4.059 | 4.7 | -1.2 | 1.538 | 1.107 |
| **Brazil** | **GDPrLGiniLow** | -4.059 | 4.7 | -1.2 | 1.572 | 1.107 |
| **Brazil** | **NoCOVIDginiB** | 2.039 | 2.371 | 2.358 | 1.758 | 1.072 |
| **Brunei Darussalam** | **GDPrGiniBase** | 1.134 | 1.974 | 2.578 | 1.134 | 0.144 |
| **Brunei Darussalam** | **GDPrHGiniH** | 1.134 | 1.974 | 4.078 | 1.122 | 0.215 |
| **Brunei Darussalam** | **GDPrHGiniHv** | 1.134 | 1.974 | 4.078 | 1.12 | 0.215 |
| **Brunei Darussalam** | **GDPrHGiniLow** | 1.134 | 1.974 | 4.078 | 1.125 | 0.215 |
| **Brunei Darussalam** | **GDPrLGiniH** | 1.134 | 1.974 | 1.078 | 1.147 | 0.072 |
| **Brunei Darussalam** | **GDPrLGiniHv** | 1.134 | 1.974 | 1.078 | 1.144 | 0.071 |
| **Brunei Darussalam** | **GDPrLGiniLow** | 1.134 | 1.974 | 1.078 | 1.15 | 0.072 |
| **Brunei Darussalam** | **NoCOVIDginiB** | 4.702 | 3.595 | 3.539 | 1.142 | 0.156 |
| **Bulgaria** | **GDPrGiniBase** | -4.387 | 4.54 | 4.4 | 1.444 | 0.469 |
| **Bulgaria** | **GDPrHGiniH** | -4.387 | 4.54 | 5.9 | 1.468 | 0.472 |
| **Bulgaria** | **GDPrHGiniHv** | -4.387 | 4.54 | 5.9 | 1.464 | 0.472 |
| **Bulgaria** | **GDPrHGiniLow** | -4.387 | 4.54 | 5.9 | 1.472 | 0.47 |
| **Bulgaria** | **GDPrLGiniH** | -4.387 | 4.54 | 2.9 | 1.424 | 0.449 |
| **Bulgaria** | **GDPrLGiniHv** | -4.387 | 4.54 | 2.9 | 1.421 | 0.449 |
| **Bulgaria** | **GDPrLGiniLow** | -4.387 | 4.54 | 2.9 | 1.428 | 0.448 |
| **Bulgaria** | **NoCOVIDginiB** | 3.2 | 3 | 2.8 | 1.613 | 0.447 |
| **Burkina Faso** | **GDPrGiniBase** | 1.93 | 6.667 | 5.613 | 6.034 | 7.453 |
| **Burkina Faso** | **GDPrHGiniH** | 1.93 | 6.667 | 7.113 | 6.136 | 7.456 |
| **Burkina Faso** | **GDPrHGiniHv** | 1.93 | 6.667 | 7.113 | 6.112 | 7.415 |
| **Burkina Faso** | **GDPrHGiniLow** | 1.93 | 6.667 | 7.113 | 6.171 | 7.504 |
| **Burkina Faso** | **GDPrLGiniH** | 1.93 | 6.667 | 4.113 | 5.897 | 7.388 |
| **Burkina Faso** | **GDPrLGiniHv** | 1.93 | 6.667 | 4.113 | 5.877 | 7.348 |
| **Burkina Faso** | **GDPrLGiniLow** | 1.93 | 6.667 | 4.113 | 5.927 | 7.444 |
| **Burkina Faso** | **NoCOVIDginiB** | 5.967 | 6.013 | 6.018 | 6.259 | 7.465 |
| **Burundi** | **GDPrGiniBase** | 0.298 | 1.634 | 4.191 | 3.984 | 6.74 |
| **Burundi** | **GDPrHGiniH** | 0.298 | 1.634 | 5.691 | 4.312 | 6.79 |
| **Burundi** | **GDPrHGiniHv** | 0.298 | 1.634 | 5.691 | 4.298 | 6.773 |
| **Burundi** | **GDPrHGiniLow** | 0.298 | 1.634 | 5.691 | 4.331 | 6.813 |
| **Burundi** | **GDPrLGiniH** | 0.298 | 1.634 | 2.691 | 3.619 | 6.668 |
| **Burundi** | **GDPrLGiniHv** | 0.298 | 1.634 | 2.691 | 3.606 | 6.643 |
| **Burundi** | **GDPrLGiniLow** | 0.298 | 1.634 | 2.691 | 3.638 | 6.701 |
| **Burundi** | **NoCOVIDginiB** | 0.501 | 0.498 | 0.499 | 3.232 | 6.386 |
| **Cabo Verde** | **GDPrGiniBase** | -14.78 | 3.995 | 6.504 | 2.758 | 2.749 |
| **Cabo Verde** | **GDPrHGiniH** | -14.78 | 3.995 | 8.004 | 2.845 | 2.712 |
| **Cabo Verde** | **GDPrHGiniHv** | -14.78 | 3.995 | 8.004 | 2.825 | 2.689 |
| **Cabo Verde** | **GDPrHGiniLow** | -14.78 | 3.995 | 8.004 | 2.872 | 2.742 |
| **Cabo Verde** | **GDPrLGiniH** | -14.78 | 3.995 | 5.004 | 2.664 | 2.749 |
| **Cabo Verde** | **GDPrLGiniHv** | -14.78 | 3.995 | 5.004 | 2.643 | 2.723 |
| **Cabo Verde** | **GDPrLGiniLow** | -14.78 | 3.995 | 5.004 | 2.689 | 2.78 |
| **Cabo Verde** | **NoCOVIDginiB** | 4.954 | 4.983 | 5.001 | 3.408 | 2.663 |
| **Cambodia** | **GDPrGiniBase** | -3.148 | 1.932 | 5.662 | 5.472 | 5.724 |
| **Cambodia** | **GDPrHGiniH** | -3.148 | 1.932 | 7.162 | 5.567 | 5.7 |
| **Cambodia** | **GDPrHGiniHv** | -3.148 | 1.932 | 7.162 | 5.556 | 5.7 |
| **Cambodia** | **GDPrHGiniLow** | -3.148 | 1.932 | 7.162 | 5.579 | 5.7 |
| **Cambodia** | **GDPrLGiniH** | -3.148 | 1.932 | 4.162 | 5.362 | 5.745 |
| **Cambodia** | **GDPrLGiniHv** | -3.148 | 1.932 | 4.162 | 5.352 | 5.745 |
| **Cambodia** | **GDPrLGiniLow** | -3.148 | 1.932 | 4.162 | 5.375 | 5.745 |
| **Cambodia** | **NoCOVIDginiB** | 6.772 | 6.704 | 6.639 | 6.153 | 5.648 |
| **Cameroon** | **GDPrGiniBase** | 0.492 | 3.625 | 4.559 | 3.877 | 4.726 |
| **Cameroon** | **GDPrHGiniH** | 0.492 | 3.625 | 6.059 | 3.961 | 4.786 |
| **Cameroon** | **GDPrHGiniHv** | 0.492 | 3.625 | 6.059 | 3.947 | 4.77 |
| **Cameroon** | **GDPrHGiniLow** | 0.492 | 3.625 | 6.059 | 3.98 | 4.803 |
| **Cameroon** | **GDPrLGiniH** | 0.492 | 3.625 | 3.059 | 3.772 | 4.637 |
| **Cameroon** | **GDPrLGiniHv** | 0.492 | 3.625 | 3.059 | 3.757 | 4.625 |
| **Cameroon** | **GDPrLGiniLow** | 0.492 | 3.625 | 3.059 | 3.791 | 4.654 |
| **Cameroon** | **NoCOVIDginiB** | 4.167 | 4.511 | 4.962 | 4.073 | 4.85 |
| **Canada** | **GDPrGiniBase** | -5.313 | 4.7 | 4.1 | 1.291 | 1.292 |
| **Canada** | **GDPrHGiniH** | -5.313 | 4.7 | 5.6 | 1.289 | 1.288 |
| **Canada** | **GDPrHGiniHv** | -5.313 | 4.7 | 5.6 | 1.287 | 1.288 |
| **Canada** | **GDPrHGiniLow** | -5.313 | 4.7 | 5.6 | 1.291 | 1.287 |
| **Canada** | **GDPrLGiniH** | -5.313 | 4.7 | 2.6 | 1.295 | 1.295 |
| **Canada** | **GDPrLGiniHv** | -5.313 | 4.7 | 2.6 | 1.293 | 1.296 |
| **Canada** | **GDPrLGiniLow** | -5.313 | 4.7 | 2.6 | 1.297 | 1.294 |
| **Canada** | **NoCOVIDginiB** | 1.761 | 1.756 | 1.719 | 1.299 | 1.263 |
| **Central African Republic** | **GDPrGiniBase** | 0.829 | -0.998 | 4.031 | 2.777 | 5.121 |
| **Central African Republic** | **GDPrHGiniH** | 0.829 | -0.998 | 5.531 | 2.889 | 5.224 |
| **Central African Republic** | **GDPrHGiniHv** | 0.829 | -0.998 | 5.531 | 2.872 | 5.198 |
| **Central African Republic** | **GDPrHGiniLow** | 0.829 | -0.998 | 5.531 | 2.911 | 5.26 |
| **Central African Republic** | **GDPrLGiniH** | 0.829 | -0.998 | 2.531 | 2.579 | 4.97 |
| **Central African Republic** | **GDPrLGiniHv** | 0.829 | -0.998 | 2.531 | 2.563 | 4.947 |
| **Central African Republic** | **GDPrLGiniLow** | 0.829 | -0.998 | 2.531 | 2.602 | 4.986 |
| **Central African Republic** | **NoCOVIDginiB** | 4.968 | 4.963 | 4.988 | 3.221 | 5.694 |
| **Chad** | **GDPrGiniBase** | -0.947 | 0.939 | 2.371 | 4.659 | 7.848 |
| **Chad** | **GDPrHGiniH** | -0.947 | 0.939 | 3.871 | 4.809 | 7.932 |
| **Chad** | **GDPrHGiniHv** | -0.947 | 0.939 | 3.871 | 4.79 | 7.909 |
| **Chad** | **GDPrHGiniLow** | -0.947 | 0.939 | 3.871 | 4.834 | 7.958 |
| **Chad** | **GDPrLGiniH** | -0.947 | 0.939 | 0.871 | 4.492 | 7.717 |
| **Chad** | **GDPrLGiniHv** | -0.947 | 0.939 | 0.871 | 4.472 | 7.693 |
| **Chad** | **GDPrLGiniLow** | -0.947 | 0.939 | 0.871 | 4.519 | 7.75 |
| **Chad** | **NoCOVIDginiB** | 5.442 | 4.814 | 5.35 | 5.619 | 8.277 |
| **Chile** | **GDPrGiniBase** | -5.772 | 11 | 2.471 | 1.987 | 1.497 |
| **Chile** | **GDPrHGiniH** | -5.772 | 11 | 3.971 | 2.014 | 1.507 |
| **Chile** | **GDPrHGiniHv** | -5.772 | 11 | 3.971 | 2.01 | 1.507 |
| **Chile** | **GDPrHGiniLow** | -5.772 | 11 | 3.971 | 2.019 | 1.508 |
| **Chile** | **GDPrLGiniH** | -5.772 | 11 | 0.971 | 1.952 | 1.579 |
| **Chile** | **GDPrLGiniHv** | -5.772 | 11 | 0.971 | 1.948 | 1.579 |
| **Chile** | **GDPrLGiniLow** | -5.772 | 11 | 0.971 | 1.957 | 1.579 |
| **Chile** | **NoCOVIDginiB** | 3.017 | 3.229 | 3.265 | 2.045 | 1.467 |
| **China** | **GDPrGiniBase** | 2.348 | 8.1 | 4.8 | 4.75 | 2.652 |
| **China** | **GDPrHGiniH** | 2.348 | 8.1 | 6.3 | 4.803 | 2.637 |
| **China** | **GDPrHGiniHv** | 2.348 | 8.1 | 6.3 | 4.801 | 2.637 |
| **China** | **GDPrHGiniLow** | 2.348 | 8.1 | 6.3 | 4.805 | 2.637 |
| **China** | **GDPrLGiniH** | 2.348 | 8.1 | 3.3 | 4.698 | 2.662 |
| **China** | **GDPrLGiniHv** | 2.348 | 8.1 | 3.3 | 4.696 | 2.662 |
| **China** | **GDPrLGiniLow** | 2.348 | 8.1 | 3.3 | 4.7 | 2.662 |
| **China** | **NoCOVIDginiB** | 5.819 | 5.9 | 5.7 | 4.833 | 2.596 |
| **Colombia** | **GDPrGiniBase** | -6.796 | 7.608 | 3.834 | 1.694 | 0.886 |
| **Colombia** | **GDPrHGiniH** | -6.796 | 7.608 | 5.334 | 1.647 | 0.877 |
| **Colombia** | **GDPrHGiniHv** | -6.796 | 7.608 | 5.334 | 1.631 | 0.876 |
| **Colombia** | **GDPrHGiniLow** | -6.796 | 7.608 | 5.334 | 1.666 | 0.879 |
| **Colombia** | **GDPrLGiniH** | -6.796 | 7.608 | 2.334 | 1.614 | 0.881 |
| **Colombia** | **GDPrLGiniHv** | -6.796 | 7.608 | 2.334 | 1.598 | 0.88 |
| **Colombia** | **GDPrLGiniLow** | -6.796 | 7.608 | 2.334 | 1.634 | 0.881 |
| **Colombia** | **NoCOVIDginiB** | 3.646 | 3.749 | 3.75 | 1.782 | 0.906 |
| **Comoros** | **GDPrGiniBase** | -0.129 | 1.6 | 3.829 | 3.517 | 5.531 |
| **Comoros** | **GDPrHGiniH** | -0.129 | 1.6 | 5.33 | 3.63 | 5.593 |
| **Comoros** | **GDPrHGiniHv** | -0.129 | 1.6 | 5.329 | 3.618 | 5.581 |
| **Comoros** | **GDPrHGiniLow** | -0.129 | 1.6 | 5.33 | 3.646 | 5.606 |
| **Comoros** | **GDPrLGiniH** | -0.129 | 1.6 | 2.33 | 3.383 | 5.439 |
| **Comoros** | **GDPrLGiniHv** | -0.129 | 1.6 | 2.329 | 3.371 | 5.429 |
| **Comoros** | **GDPrLGiniLow** | -0.129 | 1.6 | 2.33 | 3.399 | 5.454 |
| **Comoros** | **NoCOVIDginiB** | 4.253 | 3.606 | 3.509 | 3.856 | 5.722 |
| **Congo** | **GDPrGiniBase** | -7.944 | -0.164 | 2.303 | 5.797 | 7.52 |
| **Congo** | **GDPrHGiniH** | -7.944 | -0.164 | 3.803 | 5.969 | 7.35 |
| **Congo** | **GDPrHGiniHv** | -7.944 | -0.164 | 3.803 | 5.96 | 7.348 |
| **Congo** | **GDPrHGiniLow** | -7.944 | -0.164 | 3.803 | 5.987 | 7.351 |
| **Congo** | **GDPrLGiniH** | -7.944 | -0.164 | 0.803 | 5.65 | 7.376 |
| **Congo** | **GDPrLGiniHv** | -7.944 | -0.164 | 0.803 | 5.639 | 7.374 |
| **Congo** | **GDPrLGiniLow** | -7.944 | -0.164 | 0.803 | 5.665 | 7.378 |
| **Congo** | **NoCOVIDginiB** | 2.781 | 1.92 | 0.049 | 6.333 | 7.278 |
| **Congo, Dem. Republic of the** | **GDPrGiniBase** | 1.735 | 4.862 | 5.594 | 7.178 | 9.638 |
| **Congo, Dem. Republic of the** | **GDPrHGiniH** | 1.735 | 4.862 | 7.094 | 7.343 | 9.648 |
| **Congo, Dem. Republic of the** | **GDPrHGiniHv** | 1.735 | 4.862 | 7.094 | 7.329 | 9.635 |
| **Congo, Dem. Republic of the** | **GDPrHGiniLow** | 1.735 | 4.862 | 7.094 | 7.363 | 9.666 |
| **Congo, Dem. Republic of the** | **GDPrLGiniH** | 1.735 | 4.862 | 4.094 | 7.003 | 9.588 |
| **Congo, Dem. Republic of the** | **GDPrLGiniHv** | 1.735 | 4.862 | 4.094 | 6.988 | 9.574 |
| **Congo, Dem. Republic of the** | **GDPrLGiniLow** | 1.735 | 4.862 | 4.094 | 7.023 | 9.608 |
| **Congo, Dem. Republic of the** | **NoCOVIDginiB** | 3.891 | 3.393 | 4.507 | 7.053 | 9.555 |
| **Costa Rica** | **GDPrGiniBase** | -4.064 | 3.9 | 3.499 | 2.98 | 2.763 |
| **Costa Rica** | **GDPrHGiniH** | -4.064 | 3.9 | 4.999 | 3.001 | 2.765 |
| **Costa Rica** | **GDPrHGiniHv** | -4.064 | 3.9 | 4.999 | 2.996 | 2.764 |
| **Costa Rica** | **GDPrHGiniLow** | -4.064 | 3.9 | 4.999 | 3.008 | 2.766 |
| **Costa Rica** | **GDPrLGiniH** | -4.064 | 3.9 | 1.999 | 2.955 | 2.758 |
| **Costa Rica** | **GDPrLGiniHv** | -4.064 | 3.9 | 1.999 | 2.95 | 2.757 |
| **Costa Rica** | **GDPrLGiniLow** | -4.064 | 3.9 | 1.999 | 2.962 | 2.759 |
| **Costa Rica** | **NoCOVIDginiB** | 2.528 | 2.799 | 2.961 | 3.085 | 2.745 |
| **Cote D'Ivoire** | **GDPrGiniBase** | 1.958 | 5.956 | 6.47 | 6.167 | 6.741 |
| **Cote D'Ivoire** | **GDPrHGiniH** | 1.958 | 5.956 | 7.97 | 6.228 | 6.705 |
| **Cote D'Ivoire** | **GDPrHGiniHv** | 1.958 | 5.956 | 7.97 | 6.218 | 6.703 |
| **Cote D'Ivoire** | **GDPrHGiniLow** | 1.958 | 5.956 | 7.97 | 6.242 | 6.708 |
| **Cote D'Ivoire** | **GDPrLGiniH** | 1.958 | 5.956 | 4.97 | 6.09 | 6.765 |
| **Cote D'Ivoire** | **GDPrLGiniHv** | 1.958 | 5.956 | 4.97 | 6.079 | 6.761 |
| **Cote D'Ivoire** | **GDPrLGiniLow** | 1.958 | 5.956 | 4.97 | 6.104 | 6.77 |
| **Cote D'Ivoire** | **NoCOVIDginiB** | 7.294 | 7.006 | 6.724 | 6.408 | 6.655 |
| **Croatia** | **GDPrGiniBase** | -8.1 | 6.3 | 5.8 | 2.009 | 1.176 |
| **Croatia** | **GDPrHGiniH** | -8.1 | 6.3 | 7.3 | 2.058 | 1.169 |
| **Croatia** | **GDPrHGiniHv** | -8.1 | 6.3 | 7.3 | 2.056 | 1.169 |
| **Croatia** | **GDPrHGiniLow** | -8.1 | 6.3 | 7.3 | 2.06 | 1.17 |
| **Croatia** | **GDPrLGiniH** | -8.1 | 6.3 | 4.3 | 1.951 | 1.186 |
| **Croatia** | **GDPrLGiniHv** | -8.1 | 6.3 | 4.3 | 1.949 | 1.185 |
| **Croatia** | **GDPrLGiniLow** | -8.1 | 6.3 | 4.3 | 1.953 | 1.186 |
| **Croatia** | **NoCOVIDginiB** | 2.704 | 2.518 | 2.271 | 2.041 | 1.136 |
| **Cuba** | **GDPrGiniBase** | -10.95 | 0 | 0 | 1.356 | 1.867 |
| **Cuba** | **GDPrHGiniH** | -10.95 | 0 | 1.5 | 1.426 | 1.904 |
| **Cuba** | **GDPrHGiniHv** | -10.95 | 0 | 1.5 | 1.422 | 1.904 |
| **Cuba** | **GDPrHGiniLow** | -10.95 | 0 | 1.5 | 1.43 | 1.905 |
| **Cuba** | **GDPrLGiniH** | -10.95 | 0 | -1.5 | 1.303 | 1.837 |
| **Cuba** | **GDPrLGiniHv** | -10.95 | 0 | -1.5 | 1.299 | 1.837 |
| **Cuba** | **GDPrLGiniLow** | -10.95 | 0 | -1.5 | 1.307 | 1.838 |
| **Cuba** | **NoCOVIDginiB** | 0 | 0 | 0 | 1.585 | 1.986 |
| **Cyprus** | **GDPrGiniBase** | -5.229 | 4.766 | 3.552 | 2.072 | 1.653 |
| **Cyprus** | **GDPrHGiniH** | -5.229 | 4.766 | 5.052 | 2.114 | 1.643 |
| **Cyprus** | **GDPrHGiniHv** | -5.229 | 4.766 | 5.052 | 2.112 | 1.643 |
| **Cyprus** | **GDPrHGiniLow** | -5.229 | 4.766 | 5.052 | 2.118 | 1.643 |
| **Cyprus** | **GDPrLGiniH** | -5.229 | 4.766 | 2.052 | 2.02 | 1.661 |
| **Cyprus** | **GDPrLGiniHv** | -5.229 | 4.766 | 2.052 | 2.018 | 1.661 |
| **Cyprus** | **GDPrLGiniLow** | -5.229 | 4.766 | 2.052 | 2.024 | 1.661 |
| **Cyprus** | **NoCOVIDginiB** | 2.885 | 2.712 | 2.605 | 2.09 | 1.588 |
| **Czech Republic** | **GDPrGiniBase** | -5.794 | 3.787 | 4.489 | 1.292 | 0.898 |
| **Czech Republic** | **GDPrHGiniH** | -5.794 | 3.787 | 5.989 | 1.337 | 0.919 |
| **Czech Republic** | **GDPrHGiniHv** | -5.794 | 3.787 | 5.989 | 1.335 | 0.919 |
| **Czech Republic** | **GDPrHGiniLow** | -5.794 | 3.787 | 5.989 | 1.339 | 0.919 |
| **Czech Republic** | **GDPrLGiniH** | -5.794 | 3.787 | 2.989 | 1.228 | 0.871 |
| **Czech Republic** | **GDPrLGiniHv** | -5.794 | 3.787 | 2.989 | 1.226 | 0.87 |
| **Czech Republic** | **GDPrLGiniLow** | -5.794 | 3.787 | 2.989 | 1.231 | 0.872 |
| **Czech Republic** | **NoCOVIDginiB** | 2.617 | 2.646 | 2.547 | 1.39 | 0.873 |
| **Denmark** | **GDPrGiniBase** | -2.062 | 3.8 | 3 | 1.319 | 1.709 |
| **Denmark** | **GDPrHGiniH** | -2.062 | 3.8 | 4.5 | 1.307 | 1.701 |
| **Denmark** | **GDPrHGiniHv** | -2.062 | 3.8 | 4.5 | 1.309 | 1.701 |
| **Denmark** | **GDPrHGiniLow** | -2.062 | 3.8 | 4.5 | 1.306 | 1.702 |
| **Denmark** | **GDPrLGiniH** | -2.062 | 3.8 | 1.5 | 1.331 | 1.717 |
| **Denmark** | **GDPrLGiniHv** | -2.062 | 3.8 | 1.5 | 1.33 | 1.717 |
| **Denmark** | **GDPrLGiniLow** | -2.062 | 3.8 | 1.5 | 1.331 | 1.718 |
| **Denmark** | **NoCOVIDginiB** | 1.905 | 1.723 | 1.645 | 1.314 | 1.691 |
| **Djibouti** | **GDPrGiniBase** | 0.5 | 5 | 5.5 | 4.342 | 3.388 |
| **Djibouti** | **GDPrHGiniH** | 0.5 | 5 | 7 | 4.407 | 3.341 |
| **Djibouti** | **GDPrHGiniHv** | 0.5 | 5 | 7 | 4.396 | 3.338 |
| **Djibouti** | **GDPrHGiniLow** | 0.5 | 5 | 7 | 4.422 | 3.346 |
| **Djibouti** | **GDPrLGiniH** | 0.5 | 5 | 4 | 4.273 | 3.408 |
| **Djibouti** | **GDPrLGiniHv** | 0.5 | 5 | 4 | 4.262 | 3.405 |
| **Djibouti** | **GDPrLGiniLow** | 0.5 | 5 | 4 | 4.29 | 3.413 |
| **Djibouti** | **NoCOVIDginiB** | 6 | 5.999 | 5.997 | 4.578 | 3.319 |
| **Dominican Republic** | **GDPrGiniBase** | -6.72 | 9.521 | 5.502 | 4.133 | 3.65 |
| **Dominican Republic** | **GDPrHGiniH** | -6.72 | 9.521 | 7.002 | 4.163 | 3.65 |
| **Dominican Republic** | **GDPrHGiniHv** | -6.72 | 9.521 | 7.002 | 4.155 | 3.652 |
| **Dominican Republic** | **GDPrHGiniLow** | -6.72 | 9.521 | 7.002 | 4.174 | 3.65 |
| **Dominican Republic** | **GDPrLGiniH** | -6.72 | 9.521 | 4.002 | 4.088 | 3.637 |
| **Dominican Republic** | **GDPrLGiniHv** | -6.72 | 9.521 | 4.002 | 4.08 | 3.637 |
| **Dominican Republic** | **GDPrLGiniLow** | -6.72 | 9.521 | 4.002 | 4.099 | 3.637 |
| **Dominican Republic** | **NoCOVIDginiB** | 5.231 | 4.952 | 5.018 | 4.32 | 3.61 |
| **Ecuador** | **GDPrGiniBase** | -7.75 | 2.796 | 3.506 | 2.201 | 1.83 |
| **Ecuador** | **GDPrHGiniH** | -7.75 | 2.796 | 5.006 | 2.206 | 1.828 |
| **Ecuador** | **GDPrHGiniHv** | -7.75 | 2.796 | 5.006 | 2.198 | 1.826 |
| **Ecuador** | **GDPrHGiniLow** | -7.75 | 2.796 | 5.006 | 2.216 | 1.831 |
| **Ecuador** | **GDPrLGiniH** | -7.75 | 2.796 | 2.006 | 2.164 | 1.852 |
| **Ecuador** | **GDPrLGiniHv** | -7.75 | 2.796 | 2.006 | 2.155 | 1.859 |
| **Ecuador** | **GDPrLGiniLow** | -7.75 | 2.796 | 2.006 | 2.175 | 1.844 |
| **Ecuador** | **NoCOVIDginiB** | 0.478 | 1.636 | 2.716 | 2.241 | 1.822 |
| **Egypt** | **GDPrGiniBase** | 3.57 | 3.3 | 5.6 | 4.236 | 4.075 |
| **Egypt** | **GDPrHGiniH** | 3.57 | 3.3 | 7.1 | 4.264 | 4.058 |
| **Egypt** | **GDPrHGiniHv** | 3.57 | 3.3 | 7.1 | 4.262 | 4.058 |
| **Egypt** | **GDPrHGiniLow** | 3.57 | 3.3 | 7.1 | 4.266 | 4.058 |
| **Egypt** | **GDPrLGiniH** | 3.57 | 3.3 | 4.1 | 4.215 | 4.092 |
| **Egypt** | **GDPrLGiniHv** | 3.57 | 3.3 | 4.1 | 4.213 | 4.093 |
| **Egypt** | **GDPrLGiniLow** | 3.57 | 3.3 | 4.1 | 4.217 | 4.092 |
| **Egypt** | **NoCOVIDginiB** | 5.868 | 5.955 | 5.967 | 4.393 | 4.026 |
| **El Salvador** | **GDPrGiniBase** | -8.581 | 9.025 | 3.54 | 3.457 | 2.686 |
| **El Salvador** | **GDPrHGiniH** | -8.581 | 9.025 | 5.04 | 3.459 | 2.657 |
| **El Salvador** | **GDPrHGiniHv** | -8.581 | 9.025 | 5.04 | 3.432 | 2.66 |
| **El Salvador** | **GDPrHGiniLow** | -8.581 | 9.025 | 5.04 | 3.494 | 2.652 |
| **El Salvador** | **GDPrLGiniH** | -8.581 | 9.025 | 2.04 | 3.423 | 2.719 |
| **El Salvador** | **GDPrLGiniHv** | -8.581 | 9.025 | 2.04 | 3.396 | 2.721 |
| **El Salvador** | **GDPrLGiniLow** | -8.581 | 9.025 | 2.04 | 3.457 | 2.714 |
| **El Salvador** | **NoCOVIDginiB** | 2.3 | 2.2 | 2.2 | 3.522 | 2.638 |
| **Equatorial Guinea** | **GDPrGiniBase** | -4.893 | 4.146 | -5.626 | 5.033 | 4.245 |
| **Equatorial Guinea** | **GDPrHGiniH** | -4.893 | 4.146 | -4.126 | 5.096 | 4.237 |
| **Equatorial Guinea** | **GDPrHGiniHv** | -4.893 | 4.146 | -4.126 | 5.091 | 4.237 |
| **Equatorial Guinea** | **GDPrHGiniLow** | -4.893 | 4.146 | -4.126 | 5.103 | 4.236 |
| **Equatorial Guinea** | **GDPrLGiniH** | -4.893 | 4.146 | -7.126 | 4.966 | 4.244 |
| **Equatorial Guinea** | **GDPrLGiniHv** | -4.893 | 4.146 | -7.126 | 4.96 | 4.244 |
| **Equatorial Guinea** | **GDPrLGiniLow** | -4.893 | 4.146 | -7.126 | 4.974 | 4.244 |
| **Equatorial Guinea** | **NoCOVIDginiB** | -4.992 | -5.261 | -5.637 | 4.849 | 4.265 |
| **Eritrea** | **GDPrGiniBase** | -0.627 | 2.911 | 4.827 | 8.357 | 10.36 |
| **Eritrea** | **GDPrHGiniH** | -0.627 | 2.911 | 6.327 | 8.563 | 10.26 |
| **Eritrea** | **GDPrHGiniHv** | -0.627 | 2.911 | 6.327 | 8.547 | 10.25 |
| **Eritrea** | **GDPrHGiniLow** | -0.627 | 2.911 | 6.327 | 8.592 | 10.28 |
| **Eritrea** | **GDPrLGiniH** | -0.627 | 2.911 | 3.327 | 8.136 | 10.4 |
| **Eritrea** | **GDPrLGiniHv** | -0.627 | 2.911 | 3.327 | 8.112 | 10.38 |
| **Eritrea** | **GDPrLGiniLow** | -0.627 | 2.911 | 3.327 | 8.169 | 10.43 |
| **Eritrea** | **NoCOVIDginiB** | 3.859 | 4.028 | 7.16 | 9.224 | 10.12 |
| **Estonia** | **GDPrGiniBase** | -2.951 | 8.5 | 4.2 | 0.976 | 1.926 |
| **Estonia** | **GDPrHGiniH** | -2.951 | 8.5 | 5.7 | 1.065 | 1.895 |
| **Estonia** | **GDPrHGiniHv** | -2.951 | 8.5 | 5.7 | 1.061 | 1.895 |
| **Estonia** | **GDPrHGiniLow** | -2.951 | 8.5 | 5.7 | 1.07 | 1.896 |
| **Estonia** | **GDPrLGiniH** | -2.951 | 8.5 | 2.7 | 0.779 | 2.012 |
| **Estonia** | **GDPrLGiniHv** | -2.951 | 8.5 | 2.7 | 0.775 | 2.009 |
| **Estonia** | **GDPrLGiniLow** | -2.951 | 8.5 | 2.7 | 0.784 | 2.021 |
| **Estonia** | **NoCOVIDginiB** | 2.9 | 2.8 | 2.8 | 0.809 | 1.87 |
| **Eswatini** | **GDPrGiniBase** | -1.85 | 1.529 | 1.669 | 4.97 | 5.398 |
| **Eswatini** | **GDPrHGiniH** | -1.85 | 1.529 | 3.169 | 5.016 | 5.402 |
| **Eswatini** | **GDPrHGiniHv** | -1.85 | 1.529 | 3.169 | 4.997 | 5.4 |
| **Eswatini** | **GDPrHGiniLow** | -1.85 | 1.529 | 3.169 | 5.041 | 5.404 |
| **Eswatini** | **GDPrLGiniH** | -1.85 | 1.529 | 0.169 | 4.892 | 5.386 |
| **Eswatini** | **GDPrLGiniHv** | -1.85 | 1.529 | 0.169 | 4.873 | 5.383 |
| **Eswatini** | **GDPrLGiniLow** | -1.85 | 1.529 | 0.169 | 4.917 | 5.391 |
| **Eswatini** | **NoCOVIDginiB** | 0.509 | 0.638 | 0.543 | 5.061 | 5.366 |
| **Ethiopia** | **GDPrGiniBase** | 6.057 | 1.989 | 8.675 | 7.691 | 8.216 |
| **Ethiopia** | **GDPrHGiniH** | 6.057 | 1.989 | 10.18 | 7.774 | 8.221 |
| **Ethiopia** | **GDPrHGiniHv** | 6.057 | 1.989 | 10.18 | 7.747 | 8.193 |
| **Ethiopia** | **GDPrHGiniLow** | 6.057 | 1.989 | 10.18 | 7.809 | 8.259 |
| **Ethiopia** | **GDPrLGiniH** | 6.057 | 1.989 | 7.175 | 7.49 | 8.177 |
| **Ethiopia** | **GDPrLGiniHv** | 6.057 | 1.989 | 7.175 | 7.468 | 8.149 |
| **Ethiopia** | **GDPrLGiniLow** | 6.057 | 1.989 | 7.175 | 7.524 | 8.22 |
| **Ethiopia** | **NoCOVIDginiB** | 7.198 | 7.126 | 7.023 | 7.805 | 8.267 |
| **Fiji** | **GDPrGiniBase** | -15.71 | -3.997 | 6.195 | 2.203 | 3.27 |
| **Fiji** | **GDPrHGiniH** | -15.71 | -3.997 | 7.695 | 2.236 | 3.233 |
| **Fiji** | **GDPrHGiniHv** | -15.71 | -3.997 | 7.695 | 2.229 | 3.233 |
| **Fiji** | **GDPrHGiniLow** | -15.71 | -3.997 | 7.695 | 2.244 | 3.234 |
| **Fiji** | **GDPrLGiniH** | -15.71 | -3.997 | 4.695 | 2.145 | 3.314 |
| **Fiji** | **GDPrLGiniHv** | -15.71 | -3.997 | 4.695 | 2.138 | 3.313 |
| **Fiji** | **GDPrLGiniLow** | -15.71 | -3.997 | 4.695 | 2.154 | 3.314 |
| **Fiji** | **NoCOVIDginiB** | 3 | 3.199 | 3.2 | 2.753 | 3.056 |
| **Finland** | **GDPrGiniBase** | -2.871 | 2.973 | 2.963 | 0.529 | 1.007 |
| **Finland** | **GDPrHGiniH** | -2.871 | 2.973 | 4.463 | 0.539 | 1.021 |
| **Finland** | **GDPrHGiniHv** | -2.871 | 2.973 | 4.463 | 0.537 | 1.021 |
| **Finland** | **GDPrHGiniLow** | -2.871 | 2.973 | 4.463 | 0.541 | 1.021 |
| **Finland** | **GDPrLGiniH** | -2.871 | 2.973 | 1.463 | 0.51 | 0.988 |
| **Finland** | **GDPrLGiniHv** | -2.871 | 2.973 | 1.463 | 0.508 | 0.988 |
| **Finland** | **GDPrLGiniLow** | -2.871 | 2.973 | 1.463 | 0.513 | 0.988 |
| **Finland** | **NoCOVIDginiB** | 1.465 | 1.474 | 1.391 | 0.55 | 0.982 |
| **France** | **GDPrGiniBase** | -7.855 | 6.7 | 3.5 | 0.623 | 0.969 |
| **France** | **GDPrHGiniH** | -7.855 | 6.7 | 5 | 0.638 | 1.014 |
| **France** | **GDPrHGiniHv** | -7.855 | 6.7 | 5 | 0.637 | 1.014 |
| **France** | **GDPrHGiniLow** | -7.855 | 6.7 | 5 | 0.64 | 1.015 |
| **France** | **GDPrLGiniH** | -7.855 | 6.7 | 2 | 0.606 | 0.959 |
| **France** | **GDPrLGiniHv** | -7.855 | 6.7 | 2 | 0.604 | 0.959 |
| **France** | **GDPrLGiniLow** | -7.855 | 6.7 | 2 | 0.608 | 0.96 |
| **France** | **NoCOVIDginiB** | 1.263 | 1.338 | 1.391 | 0.633 | 0.956 |
| **Gabon** | **GDPrGiniBase** | -1.838 | 1.481 | 3.905 | 2.774 | 3.05 |
| **Gabon** | **GDPrHGiniH** | -1.838 | 1.481 | 5.405 | 2.635 | 3.067 |
| **Gabon** | **GDPrHGiniHv** | -1.838 | 1.481 | 5.405 | 2.63 | 3.065 |
| **Gabon** | **GDPrHGiniLow** | -1.838 | 1.481 | 5.405 | 2.64 | 3.068 |
| **Gabon** | **GDPrLGiniH** | -1.838 | 1.481 | 2.405 | 2.758 | 3.01 |
| **Gabon** | **GDPrLGiniHv** | -1.838 | 1.481 | 2.405 | 2.754 | 3.007 |
| **Gabon** | **GDPrLGiniLow** | -1.838 | 1.481 | 2.405 | 2.763 | 3.01 |
| **Gabon** | **NoCOVIDginiB** | 3.419 | 3.723 | 4.475 | 2.909 | 3.125 |
| **Gambia** | **GDPrGiniBase** | -0.215 | 4.86 | 6 | 6.506 | 7.524 |
| **Gambia** | **GDPrHGiniH** | -0.215 | 4.86 | 7.5 | 6.589 | 7.501 |
| **Gambia** | **GDPrHGiniHv** | -0.215 | 4.86 | 7.5 | 6.573 | 7.479 |
| **Gambia** | **GDPrHGiniLow** | -0.215 | 4.86 | 7.5 | 6.612 | 7.531 |
| **Gambia** | **GDPrLGiniH** | -0.215 | 4.86 | 4.5 | 6.353 | 7.484 |
| **Gambia** | **GDPrLGiniHv** | -0.215 | 4.86 | 4.5 | 6.334 | 7.46 |
| **Gambia** | **GDPrLGiniLow** | -0.215 | 4.86 | 4.5 | 6.379 | 7.518 |
| **Gambia** | **NoCOVIDginiB** | 6.404 | 5.596 | 5.403 | 6.677 | 7.489 |
| **Georgia** | **GDPrGiniBase** | -6.76 | 7.69 | 5.764 | 2.763 | 2.314 |
| **Georgia** | **GDPrHGiniH** | -6.76 | 7.69 | 7.264 | 2.728 | 2.295 |
| **Georgia** | **GDPrHGiniHv** | -6.76 | 7.69 | 7.264 | 2.722 | 2.29 |
| **Georgia** | **GDPrHGiniLow** | -6.76 | 7.69 | 7.264 | 2.735 | 2.299 |
| **Georgia** | **GDPrLGiniH** | -6.76 | 7.69 | 4.264 | 2.761 | 2.302 |
| **Georgia** | **GDPrLGiniHv** | -6.76 | 7.69 | 4.264 | 2.756 | 2.298 |
| **Georgia** | **GDPrLGiniLow** | -6.76 | 7.69 | 4.264 | 2.769 | 2.307 |
| **Georgia** | **NoCOVIDginiB** | 4.801 | 5 | 5.2 | 2.813 | 2.24 |
| **Germany** | **GDPrGiniBase** | -4.57 | 2.7 | 3.8 | -0.006 | 0.632 |
| **Germany** | **GDPrHGiniH** | -4.57 | 2.7 | 5.3 | 0.006 | 0.654 |
| **Germany** | **GDPrHGiniHv** | -4.57 | 2.7 | 5.3 | 0.004 | 0.655 |
| **Germany** | **GDPrHGiniLow** | -4.57 | 2.7 | 5.3 | 0.008 | 0.653 |
| **Germany** | **GDPrLGiniH** | -4.57 | 2.7 | 2.3 | -0.02 | 0.587 |
| **Germany** | **GDPrLGiniHv** | -4.57 | 2.7 | 2.3 | -0.022 | 0.589 |
| **Germany** | **GDPrLGiniLow** | -4.57 | 2.7 | 2.3 | -0.018 | 0.585 |
| **Germany** | **NoCOVIDginiB** | 1.247 | 1.439 | 1.336 | 0.031 | 0.611 |
| **Ghana** | **GDPrGiniBase** | 0.414 | 4.712 | 6.172 | 5.986 | 7.257 |
| **Ghana** | **GDPrHGiniH** | 0.414 | 4.712 | 7.672 | 6.074 | 7.225 |
| **Ghana** | **GDPrHGiniHv** | 0.414 | 4.712 | 7.672 | 6.055 | 7.214 |
| **Ghana** | **GDPrHGiniLow** | 0.414 | 4.712 | 7.672 | 6.098 | 7.236 |
| **Ghana** | **GDPrLGiniH** | 0.414 | 4.712 | 4.672 | 5.872 | 7.255 |
| **Ghana** | **GDPrLGiniHv** | 0.414 | 4.712 | 4.672 | 5.854 | 7.241 |
| **Ghana** | **GDPrLGiniLow** | 0.414 | 4.712 | 4.672 | 5.897 | 7.272 |
| **Ghana** | **NoCOVIDginiB** | 5.607 | 4.222 | 4.616 | 6.147 | 7.227 |
| **Greece** | **GDPrGiniBase** | -9.019 | 6.494 | 4.607 | 0.581 | 0.705 |
| **Greece** | **GDPrHGiniH** | -9.019 | 6.494 | 6.107 | 0.627 | 0.695 |
| **Greece** | **GDPrHGiniHv** | -9.019 | 6.494 | 6.107 | 0.625 | 0.695 |
| **Greece** | **GDPrHGiniLow** | -9.019 | 6.494 | 6.107 | 0.629 | 0.695 |
| **Greece** | **GDPrLGiniH** | -9.019 | 6.494 | 3.107 | 0.538 | 0.717 |
| **Greece** | **GDPrLGiniHv** | -9.019 | 6.494 | 3.107 | 0.536 | 0.717 |
| **Greece** | **GDPrLGiniLow** | -9.019 | 6.494 | 3.107 | 0.54 | 0.717 |
| **Greece** | **NoCOVIDginiB** | 2.22 | 1.707 | 1.345 | 0.644 | 0.686 |
| **Grenada** | **GDPrGiniBase** | -13.71 | 2.679 | 6.168 | 2.648 | 2.3 |
| **Grenada** | **GDPrHGiniH** | -13.71 | 2.679 | 7.668 | 2.668 | 2.276 |
| **Grenada** | **GDPrHGiniHv** | -13.71 | 2.679 | 7.668 | 2.659 | 2.27 |
| **Grenada** | **GDPrHGiniLow** | -13.71 | 2.679 | 7.668 | 2.679 | 2.282 |
| **Grenada** | **GDPrLGiniH** | -13.71 | 2.679 | 4.668 | 2.614 | 2.277 |
| **Grenada** | **GDPrLGiniHv** | -13.71 | 2.679 | 4.668 | 2.605 | 2.273 |
| **Grenada** | **GDPrLGiniLow** | -13.71 | 2.679 | 4.668 | 2.625 | 2.287 |
| **Grenada** | **NoCOVIDginiB** | 2.731 | 2.658 | 3.178 | 2.719 | 2.162 |
| **Guatemala** | **GDPrGiniBase** | -1.519 | 5.5 | 4.501 | 4.016 | 3.641 |
| **Guatemala** | **GDPrHGiniH** | -1.519 | 5.5 | 6.001 | 4.04 | 3.623 |
| **Guatemala** | **GDPrHGiniHv** | -1.519 | 5.5 | 6.001 | 4.015 | 3.619 |
| **Guatemala** | **GDPrHGiniLow** | -1.519 | 5.5 | 6.001 | 4.071 | 3.626 |
| **Guatemala** | **GDPrLGiniH** | -1.519 | 5.5 | 3.001 | 3.956 | 3.654 |
| **Guatemala** | **GDPrLGiniHv** | -1.519 | 5.5 | 3.001 | 3.932 | 3.65 |
| **Guatemala** | **GDPrLGiniLow** | -1.519 | 5.5 | 3.001 | 3.987 | 3.658 |
| **Guatemala** | **NoCOVIDginiB** | 3.541 | 3.72 | 3.596 | 4.082 | 3.631 |
| **Guinea** | **GDPrGiniBase** | 6.988 | 5.173 | 6.289 | 4.902 | 6.073 |
| **Guinea** | **GDPrHGiniH** | 6.988 | 5.173 | 7.789 | 4.97 | 6.093 |
| **Guinea** | **GDPrHGiniHv** | 6.988 | 5.173 | 7.789 | 4.957 | 6.082 |
| **Guinea** | **GDPrHGiniLow** | 6.988 | 5.173 | 7.789 | 4.986 | 6.108 |
| **Guinea** | **GDPrLGiniH** | 6.988 | 5.173 | 4.789 | 4.793 | 6.043 |
| **Guinea** | **GDPrLGiniHv** | 6.988 | 5.173 | 4.789 | 4.78 | 6.033 |
| **Guinea** | **GDPrLGiniLow** | 6.988 | 5.173 | 4.789 | 4.811 | 6.057 |
| **Guinea** | **NoCOVIDginiB** | 5.963 | 5.99 | 5.281 | 4.89 | 6.02 |
| **Guinea Bissau** | **GDPrGiniBase** | -2.4 | 3.301 | 4 | 3.979 | 6.647 |
| **Guinea Bissau** | **GDPrHGiniH** | -2.4 | 3.301 | 5.5 | 4.157 | 6.801 |
| **Guinea Bissau** | **GDPrHGiniHv** | -2.4 | 3.301 | 5.5 | 4.139 | 6.773 |
| **Guinea Bissau** | **GDPrHGiniLow** | -2.4 | 3.301 | 5.5 | 4.181 | 6.84 |
| **Guinea Bissau** | **GDPrLGiniH** | -2.4 | 3.301 | 2.5 | 3.769 | 6.405 |
| **Guinea Bissau** | **GDPrLGiniHv** | -2.4 | 3.301 | 2.5 | 3.751 | 6.376 |
| **Guinea Bissau** | **GDPrLGiniLow** | -2.4 | 3.301 | 2.5 | 3.794 | 6.446 |
| **Guinea Bissau** | **NoCOVIDginiB** | 4.905 | 4.996 | 5.1 | 4.767 | 7.2 |
| **Guyana** | **GDPrGiniBase** | 43.48 | 20.41 | 48.71 | 7.743 | 2.087 |
| **Guyana** | **GDPrHGiniH** | 43.48 | 20.41 | 50.21 | 7.789 | 2.071 |
| **Guyana** | **GDPrHGiniHv** | 43.48 | 20.41 | 50.21 | 7.778 | 2.071 |
| **Guyana** | **GDPrHGiniLow** | 43.48 | 20.41 | 50.21 | 7.803 | 2.069 |
| **Guyana** | **GDPrLGiniH** | 43.48 | 20.41 | 47.21 | 7.683 | 2.088 |
| **Guyana** | **GDPrLGiniHv** | 43.48 | 20.41 | 47.21 | 7.672 | 2.089 |
| **Guyana** | **GDPrLGiniLow** | 43.48 | 20.41 | 47.21 | 7.697 | 2.086 |
| **Guyana** | **NoCOVIDginiB** | 85.62 | 4.766 | 20.63 | 7.436 | 2.37 |
| **Haiti** | **GDPrGiniBase** | -3.343 | -0.7 | 1.3 | 2.295 | 3.473 |
| **Haiti** | **GDPrHGiniH** | -3.343 | -0.7 | 2.8 | 2.397 | 3.52 |
| **Haiti** | **GDPrHGiniHv** | -3.343 | -0.7 | 2.8 | 2.384 | 3.512 |
| **Haiti** | **GDPrHGiniLow** | -3.343 | -0.7 | 2.8 | 2.413 | 3.53 |
| **Haiti** | **GDPrLGiniH** | -3.343 | -0.7 | -0.2 | 2.17 | 3.404 |
| **Haiti** | **GDPrLGiniHv** | -3.343 | -0.7 | -0.2 | 2.159 | 3.395 |
| **Haiti** | **GDPrLGiniLow** | -3.343 | -0.7 | -0.2 | 2.186 | 3.416 |
| **Haiti** | **NoCOVIDginiB** | 1.199 | 1.495 | 1.495 | 2.607 | 3.566 |
| **Honduras** | **GDPrGiniBase** | -8.965 | 4.9 | 4.35 | 3.341 | 4.164 |
| **Honduras** | **GDPrHGiniH** | -8.965 | 4.9 | 5.85 | 3.389 | 4.147 |
| **Honduras** | **GDPrHGiniHv** | -8.965 | 4.9 | 5.85 | 3.369 | 4.142 |
| **Honduras** | **GDPrHGiniLow** | -8.965 | 4.9 | 5.85 | 3.422 | 4.152 |
| **Honduras** | **GDPrLGiniH** | -8.965 | 4.9 | 2.85 | 3.259 | 4.171 |
| **Honduras** | **GDPrLGiniHv** | -8.965 | 4.9 | 2.85 | 3.239 | 4.164 |
| **Honduras** | **GDPrLGiniLow** | -8.965 | 4.9 | 2.85 | 3.292 | 4.178 |
| **Honduras** | **NoCOVIDginiB** | 3.528 | 3.634 | 3.743 | 3.649 | 4.109 |
| **Hong Kong** | **GDPrGiniBase** | -6.081 | 6.435 | 3.518 | 2.107 | 0.642 |
| **Hong Kong** | **GDPrHGiniH** | -6.081 | 6.435 | 5.018 | 2.068 | 0.595 |
| **Hong Kong** | **GDPrHGiniHv** | -6.081 | 6.435 | 5.018 | 2.065 | 0.595 |
| **Hong Kong** | **GDPrHGiniLow** | -6.081 | 6.435 | 5.018 | 2.071 | 0.595 |
| **Hong Kong** | **GDPrLGiniH** | -6.081 | 6.435 | 2.018 | 2.154 | 0.67 |
| **Hong Kong** | **GDPrLGiniHv** | -6.081 | 6.435 | 2.018 | 2.152 | 0.67 |
| **Hong Kong** | **GDPrLGiniLow** | -6.081 | 6.435 | 2.018 | 2.157 | 0.671 |
| **Hong Kong** | **NoCOVIDginiB** | 1.464 | 2.461 | 2.656 | 2.044 | 0.54 |
| **Hungary** | **GDPrGiniBase** | -4.676 | 7.6 | 5.1 | 2.099 | 1.108 |
| **Hungary** | **GDPrHGiniH** | -4.676 | 7.6 | 6.6 | 2.15 | 1.103 |
| **Hungary** | **GDPrHGiniHv** | -4.676 | 7.6 | 6.6 | 2.148 | 1.103 |
| **Hungary** | **GDPrHGiniLow** | -4.676 | 7.6 | 6.6 | 2.153 | 1.104 |
| **Hungary** | **GDPrLGiniH** | -4.676 | 7.6 | 3.6 | 2.052 | 1.108 |
| **Hungary** | **GDPrLGiniHv** | -4.676 | 7.6 | 3.6 | 2.049 | 1.108 |
| **Hungary** | **GDPrLGiniLow** | -4.676 | 7.6 | 3.6 | 2.055 | 1.108 |
| **Hungary** | **NoCOVIDginiB** | 3.3 | 2.9 | 2.6 | 2.081 | 1.085 |
| **Iceland** | **GDPrGiniBase** | -6.504 | 3.696 | 4.07 | 2.207 | 1.723 |
| **Iceland** | **GDPrHGiniH** | -6.504 | 3.696 | 5.57 | 2.256 | 1.709 |
| **Iceland** | **GDPrHGiniHv** | -6.504 | 3.696 | 5.57 | 2.255 | 1.709 |
| **Iceland** | **GDPrHGiniLow** | -6.504 | 3.696 | 5.57 | 2.257 | 1.709 |
| **Iceland** | **GDPrLGiniH** | -6.504 | 3.696 | 2.57 | 2.161 | 1.733 |
| **Iceland** | **GDPrLGiniHv** | -6.504 | 3.696 | 2.57 | 2.16 | 1.733 |
| **Iceland** | **GDPrLGiniLow** | -6.504 | 3.696 | 2.57 | 2.163 | 1.733 |
| **Iceland** | **NoCOVIDginiB** | 1.631 | 2.048 | 2.035 | 2.192 | 1.653 |
| **India** | **GDPrGiniBase** | -7.252 | 9 | 9 | 4.999 | 4.408 |
| **India** | **GDPrHGiniH** | -7.252 | 9 | 10.5 | 5.099 | 4.381 |
| **India** | **GDPrHGiniHv** | -7.252 | 9 | 10.5 | 5.092 | 4.382 |
| **India** | **GDPrHGiniLow** | -7.252 | 9 | 10.5 | 5.11 | 4.373 |
| **India** | **GDPrLGiniH** | -7.252 | 9 | 7.5 | 4.983 | 4.423 |
| **India** | **GDPrLGiniHv** | -7.252 | 9 | 7.5 | 4.976 | 4.423 |
| **India** | **GDPrLGiniLow** | -7.252 | 9 | 7.5 | 4.993 | 4.421 |
| **India** | **NoCOVIDginiB** | 7.033 | 7.442 | 7.436 | 5.421 | 4.198 |
| **Indonesia** | **GDPrGiniBase** | -2.07 | 3.3 | 5.6 | 2.379 | 1.926 |
| **Indonesia** | **GDPrHGiniH** | -2.07 | 3.3 | 7.1 | 2.514 | 1.939 |
| **Indonesia** | **GDPrHGiniHv** | -2.07 | 3.3 | 7.1 | 2.512 | 1.94 |
| **Indonesia** | **GDPrHGiniLow** | -2.07 | 3.3 | 7.1 | 2.514 | 1.936 |
| **Indonesia** | **GDPrLGiniH** | -2.07 | 3.3 | 4.1 | 2.351 | 1.927 |
| **Indonesia** | **GDPrLGiniHv** | -2.07 | 3.3 | 4.1 | 2.347 | 1.928 |
| **Indonesia** | **GDPrLGiniLow** | -2.07 | 3.3 | 4.1 | 2.353 | 1.923 |
| **Indonesia** | **NoCOVIDginiB** | 5.072 | 5.226 | 5.307 | 2.673 | 1.956 |
| **Iran** | **GDPrGiniBase** | 3.388 | 2.5 | 2 | 1.876 | 0.57 |
| **Iran** | **GDPrHGiniH** | 3.388 | 2.5 | 3.5 | 1.897 | 0.584 |
| **Iran** | **GDPrHGiniHv** | 3.388 | 2.5 | 3.5 | 1.895 | 0.584 |
| **Iran** | **GDPrHGiniLow** | 3.388 | 2.5 | 3.5 | 1.9 | 0.584 |
| **Iran** | **GDPrLGiniH** | 3.388 | 2.5 | 0.5 | 1.853 | 0.562 |
| **Iran** | **GDPrLGiniHv** | 3.388 | 2.5 | 0.5 | 1.851 | 0.562 |
| **Iran** | **GDPrLGiniLow** | 3.388 | 2.5 | 0.5 | 1.856 | 0.562 |
| **Iran** | **NoCOVIDginiB** | 0.046 | 0.988 | 0.975 | 1.824 | 0.551 |
| **Iraq** | **GDPrGiniBase** | -15.67 | 3.647 | 10.5 | 4.91 | 5.073 |
| **Iraq** | **GDPrHGiniH** | -15.67 | 3.647 | 12 | 4.93 | 4.936 |
| **Iraq** | **GDPrHGiniHv** | -15.67 | 3.647 | 12 | 4.927 | 4.934 |
| **Iraq** | **GDPrHGiniLow** | -15.67 | 3.647 | 12 | 4.935 | 4.94 |
| **Iraq** | **GDPrLGiniH** | -15.67 | 3.647 | 9.004 | 4.978 | 4.89 |
| **Iraq** | **GDPrLGiniHv** | -15.67 | 3.647 | 9.004 | 4.973 | 4.886 |
| **Iraq** | **GDPrLGiniLow** | -15.67 | 3.647 | 9.004 | 4.984 | 4.895 |
| **Iraq** | **NoCOVIDginiB** | 4.676 | 3.727 | 2.323 | 5.182 | 4.939 |
| **Ireland** | **GDPrGiniBase** | 5.867 | 13.04 | 3.538 | 2.705 | 1.103 |
| **Ireland** | **GDPrHGiniH** | 5.867 | 13.04 | 5.038 | 2.636 | 1.099 |
| **Ireland** | **GDPrHGiniHv** | 5.867 | 13.04 | 5.038 | 2.635 | 1.098 |
| **Ireland** | **GDPrHGiniLow** | 5.867 | 13.04 | 5.038 | 2.638 | 1.1 |
| **Ireland** | **GDPrLGiniH** | 5.867 | 13.04 | 2.038 | 2.773 | 1.107 |
| **Ireland** | **GDPrLGiniHv** | 5.867 | 13.04 | 2.038 | 2.772 | 1.107 |
| **Ireland** | **GDPrLGiniLow** | 5.867 | 13.04 | 2.038 | 2.775 | 1.107 |
| **Ireland** | **NoCOVIDginiB** | 3.501 | 3.15 | 2.886 | 3.205 | 1.204 |
| **Israel** | **GDPrGiniBase** | -2.153 | 7.06 | 4.071 | 7.02 | 3.94 |
| **Israel** | **GDPrHGiniH** | -2.153 | 7.06 | 5.571 | 7.058 | 3.927 |
| **Israel** | **GDPrHGiniHv** | -2.153 | 7.06 | 5.571 | 7.056 | 3.927 |
| **Israel** | **GDPrHGiniLow** | -2.153 | 7.06 | 5.571 | 7.062 | 3.927 |
| **Israel** | **GDPrLGiniH** | -2.153 | 7.06 | 2.571 | 6.978 | 3.945 |
| **Israel** | **GDPrLGiniHv** | -2.153 | 7.06 | 2.571 | 6.975 | 3.944 |
| **Israel** | **GDPrLGiniLow** | -2.153 | 7.06 | 2.571 | 6.981 | 3.945 |
| **Israel** | **NoCOVIDginiB** | 3.069 | 3.16 | 3.093 | 7.064 | 3.908 |
| **Italy** | **GDPrGiniBase** | -8.939 | 6.2 | 3.8 | 0.478 | 0.638 |
| **Italy** | **GDPrHGiniH** | -8.939 | 6.2 | 5.3 | 0.501 | 0.629 |
| **Italy** | **GDPrHGiniHv** | -8.939 | 6.2 | 5.3 | 0.499 | 0.629 |
| **Italy** | **GDPrHGiniLow** | -8.939 | 6.2 | 5.3 | 0.503 | 0.629 |
| **Italy** | **GDPrLGiniH** | -8.939 | 6.2 | 2.3 | 0.472 | 0.644 |
| **Italy** | **GDPrLGiniHv** | -8.939 | 6.2 | 2.3 | 0.47 | 0.644 |
| **Italy** | **GDPrLGiniLow** | -8.939 | 6.2 | 2.3 | 0.474 | 0.644 |
| **Italy** | **NoCOVIDginiB** | 0.537 | 0.774 | 0.687 | 0.515 | 0.624 |
| **Jamaica** | **GDPrGiniBase** | -10 | 4.576 | 2.686 | 1.376 | 1.896 |
| **Jamaica** | **GDPrHGiniH** | -10 | 4.576 | 4.186 | 1.388 | 1.871 |
| **Jamaica** | **GDPrHGiniHv** | -10 | 4.576 | 4.186 | 1.374 | 1.869 |
| **Jamaica** | **GDPrHGiniLow** | -10 | 4.576 | 4.186 | 1.405 | 1.873 |
| **Jamaica** | **GDPrLGiniH** | -10 | 4.576 | 1.186 | 1.348 | 1.913 |
| **Jamaica** | **GDPrLGiniHv** | -10 | 4.576 | 1.186 | 1.334 | 1.911 |
| **Jamaica** | **GDPrLGiniLow** | -10 | 4.576 | 1.186 | 1.365 | 1.916 |
| **Jamaica** | **NoCOVIDginiB** | 1.021 | 1.7 | 1.975 | 1.488 | 1.825 |
| **Japan** | **GDPrGiniBase** | -4.586 | 1.6 | 3.3 | 0.058 | 0.038 |
| **Japan** | **GDPrHGiniH** | -4.586 | 1.6 | 4.8 | 0.062 | 0.038 |
| **Japan** | **GDPrHGiniHv** | -4.586 | 1.6 | 4.8 | 0.06 | 0.038 |
| **Japan** | **GDPrHGiniLow** | -4.586 | 1.6 | 4.8 | 0.064 | 0.039 |
| **Japan** | **GDPrLGiniH** | -4.586 | 1.6 | 1.8 | 0.052 | 0.037 |
| **Japan** | **GDPrLGiniHv** | -4.586 | 1.6 | 1.8 | 0.05 | 0.037 |
| **Japan** | **GDPrLGiniLow** | -4.586 | 1.6 | 1.8 | 0.054 | 0.038 |
| **Japan** | **NoCOVIDginiB** | 0.466 | 0.453 | 0.522 | 0.109 | 0.026 |
| **Jordan** | **GDPrGiniBase** | -1.551 | 2.039 | 2.7 | 2.744 | 3.134 |
| **Jordan** | **GDPrHGiniH** | -1.551 | 2.039 | 4.2 | 2.772 | 3.092 |
| **Jordan** | **GDPrHGiniHv** | -1.551 | 2.039 | 4.2 | 2.767 | 3.086 |
| **Jordan** | **GDPrHGiniLow** | -1.551 | 2.039 | 4.2 | 2.779 | 3.099 |
| **Jordan** | **GDPrLGiniH** | -1.551 | 2.039 | 1.2 | 2.691 | 3.163 |
| **Jordan** | **GDPrLGiniHv** | -1.551 | 2.039 | 1.2 | 2.685 | 3.159 |
| **Jordan** | **GDPrLGiniLow** | -1.551 | 2.039 | 1.2 | 2.699 | 3.169 |
| **Jordan** | **NoCOVIDginiB** | 2.4 | 2.6 | 2.8 | 2.869 | 3.073 |
| **Kazakhstan** | **GDPrGiniBase** | -2.5 | 3.7 | 3.8 | 3.548 | 2.777 |
| **Kazakhstan** | **GDPrHGiniH** | -2.5 | 3.7 | 5.3 | 3.557 | 2.786 |
| **Kazakhstan** | **GDPrHGiniHv** | -2.5 | 3.7 | 5.3 | 3.55 | 2.783 |
| **Kazakhstan** | **GDPrHGiniLow** | -2.5 | 3.7 | 5.3 | 3.566 | 2.79 |
| **Kazakhstan** | **GDPrLGiniH** | -2.5 | 3.7 | 2.3 | 3.536 | 2.759 |
| **Kazakhstan** | **GDPrLGiniHv** | -2.5 | 3.7 | 2.3 | 3.528 | 2.756 |
| **Kazakhstan** | **GDPrLGiniLow** | -2.5 | 3.7 | 2.3 | 3.545 | 2.763 |
| **Kazakhstan** | **NoCOVIDginiB** | 3.917 | 3.708 | 3.32 | 3.751 | 2.652 |
| **Kenya** | **GDPrGiniBase** | -0.316 | 5.599 | 6.045 | 5.305 | 6.848 |
| **Kenya** | **GDPrHGiniH** | -0.316 | 5.599 | 7.545 | 5.448 | 6.861 |
| **Kenya** | **GDPrHGiniHv** | -0.316 | 5.599 | 7.545 | 5.432 | 6.854 |
| **Kenya** | **GDPrHGiniLow** | -0.316 | 5.599 | 7.545 | 5.468 | 6.868 |
| **Kenya** | **GDPrLGiniH** | -0.316 | 5.599 | 4.545 | 5.143 | 6.817 |
| **Kenya** | **GDPrLGiniHv** | -0.316 | 5.599 | 4.545 | 5.127 | 6.809 |
| **Kenya** | **GDPrLGiniLow** | -0.316 | 5.599 | 4.545 | 5.161 | 6.825 |
| **Kenya** | **NoCOVIDginiB** | 6.046 | 5.815 | 5.841 | 5.591 | 6.916 |
| **Korea, Dem. People's Republic** | **GDPrGiniBase** | 0 | 0 | 0 | 0.97 | 1.709 |
| **Korea, Dem. People's Republic** | **GDPrHGiniH** | 0 | 0 | 1.5 | 0.902 | 1.855 |
| **Korea, Dem. People's Republic** | **GDPrHGiniHv** | 0 | 0 | 1.5 | 0.895 | 1.696 |
| **Korea, Dem. People's Republic** | **GDPrHGiniLow** | 0 | 0 | 1.5 | 0.911 | 1.862 |
| **Korea, Dem. People's Republic** | **GDPrLGiniH** | 0 | 0 | -1.5 | 0.994 | 1.656 |
| **Korea, Dem. People's Republic** | **GDPrLGiniHv** | 0 | 0 | -1.5 | 0.987 | 1.65 |
| **Korea, Dem. People's Republic** | **GDPrLGiniLow** | 0 | 0 | -1.5 | 1.003 | 1.665 |
| **Korea, Dem. People's Republic** | **NoCOVIDginiB** | 0 | 0 | 0 | 0.965 | 1.631 |
| **Korea, Republic of** | **GDPrGiniBase** | -0.852 | 4 | 3 | 1.514 | 1.094 |
| **Korea, Republic of** | **GDPrHGiniH** | -0.852 | 4 | 4.5 | 1.54 | 1.108 |
| **Korea, Republic of** | **GDPrHGiniHv** | -0.852 | 4 | 4.5 | 1.539 | 1.107 |
| **Korea, Republic of** | **GDPrHGiniLow** | -0.852 | 4 | 4.5 | 1.542 | 1.108 |
| **Korea, Republic of** | **GDPrLGiniH** | -0.852 | 4 | 1.5 | 1.485 | 1.079 |
| **Korea, Republic of** | **GDPrLGiniHv** | -0.852 | 4 | 1.5 | 1.484 | 1.079 |
| **Korea, Republic of** | **GDPrLGiniLow** | -0.852 | 4 | 1.5 | 1.487 | 1.08 |
| **Korea, Republic of** | **NoCOVIDginiB** | 2.218 | 2.748 | 2.894 | 1.712 | 1.037 |
| **Kosovo** | **GDPrGiniBase** | -5.34 | 6 | 4.5 | 2.996 | 3.21 |
| **Kosovo** | **GDPrHGiniH** | -5.34 | 6 | 6 | 3.046 | 3.199 |
| **Kosovo** | **GDPrHGiniHv** | -5.34 | 6 | 6 | 3.044 | 3.198 |
| **Kosovo** | **GDPrHGiniLow** | -5.34 | 6 | 6 | 3.05 | 3.201 |
| **Kosovo** | **GDPrLGiniH** | -5.34 | 6 | 3 | 2.935 | 3.217 |
| **Kosovo** | **GDPrLGiniHv** | -5.34 | 6 | 3 | 2.932 | 3.215 |
| **Kosovo** | **GDPrLGiniLow** | -5.34 | 6 | 3 | 2.939 | 3.218 |
| **Kosovo** | **NoCOVIDginiB** | 3.999 | 4 | 4 | 3.175 | 3.136 |
| **Kuwait** | **GDPrGiniBase** | -8.685 | 0.948 | 4.327 | 0.615 | -1.071 |
| **Kuwait** | **GDPrHGiniH** | -8.685 | 0.948 | 5.827 | 0.549 | -0.976 |
| **Kuwait** | **GDPrHGiniHv** | -8.685 | 0.948 | 5.827 | 0.547 | -0.976 |
| **Kuwait** | **GDPrHGiniLow** | -8.685 | 0.948 | 5.827 | 0.552 | -0.975 |
| **Kuwait** | **GDPrLGiniH** | -8.685 | 0.948 | 2.827 | 0.711 | -0.977 |
| **Kuwait** | **GDPrLGiniHv** | -8.685 | 0.948 | 2.827 | 0.709 | -0.975 |
| **Kuwait** | **GDPrLGiniLow** | -8.685 | 0.948 | 2.827 | 0.714 | -0.979 |
| **Kuwait** | **NoCOVIDginiB** | 3.071 | 2.629 | 2.259 | 0.673 | -1.011 |
| **Kyrgyzstan** | **GDPrGiniBase** | -8.617 | 2.098 | 5.633 | 2.331 | 2.95 |
| **Kyrgyzstan** | **GDPrHGiniH** | -8.617 | 2.098 | 7.133 | 2.381 | 2.964 |
| **Kyrgyzstan** | **GDPrHGiniHv** | -8.617 | 2.098 | 7.133 | 2.375 | 2.957 |
| **Kyrgyzstan** | **GDPrHGiniLow** | -8.617 | 2.098 | 7.133 | 2.389 | 2.972 |
| **Kyrgyzstan** | **GDPrLGiniH** | -8.617 | 2.098 | 4.133 | 2.249 | 2.939 |
| **Kyrgyzstan** | **GDPrLGiniHv** | -8.617 | 2.098 | 4.133 | 2.242 | 2.935 |
| **Kyrgyzstan** | **GDPrLGiniLow** | -8.617 | 2.098 | 4.133 | 2.258 | 2.944 |
| **Kyrgyzstan** | **NoCOVIDginiB** | 3.387 | 3.779 | 4.58 | 2.696 | 3.022 |
| **Lao People's Dem. Republic** | **GDPrGiniBase** | 0.503 | 2.061 | 4.233 | 6.509 | 6.258 |
| **Lao People's Dem. Republic** | **GDPrHGiniH** | 0.503 | 2.061 | 5.733 | 6.62 | 6.25 |
| **Lao People's Dem. Republic** | **GDPrHGiniHv** | 0.503 | 2.061 | 5.733 | 6.616 | 6.249 |
| **Lao People's Dem. Republic** | **GDPrHGiniLow** | 0.503 | 2.061 | 5.733 | 6.625 | 6.25 |
| **Lao People's Dem. Republic** | **GDPrLGiniH** | 0.503 | 2.061 | 2.733 | 6.391 | 6.264 |
| **Lao People's Dem. Republic** | **GDPrLGiniHv** | 0.503 | 2.061 | 2.733 | 6.386 | 6.264 |
| **Lao People's Dem. Republic** | **GDPrLGiniLow** | 0.503 | 2.061 | 2.733 | 6.396 | 6.264 |
| **Lao People's Dem. Republic** | **NoCOVIDginiB** | 6.507 | 6.736 | 6.771 | 7.129 | 6.154 |
| **Latvia** | **GDPrGiniBase** | -3.619 | 4.54 | 5.2 | 2.217 | 2.401 |
| **Latvia** | **GDPrHGiniH** | -3.619 | 4.54 | 6.7 | 2.258 | 2.425 |
| **Latvia** | **GDPrHGiniHv** | -3.619 | 4.54 | 6.7 | 2.253 | 2.426 |
| **Latvia** | **GDPrHGiniLow** | -3.619 | 4.54 | 6.7 | 2.264 | 2.426 |
| **Latvia** | **GDPrLGiniH** | -3.619 | 4.54 | 3.7 | 2.168 | 2.364 |
| **Latvia** | **GDPrLGiniHv** | -3.619 | 4.54 | 3.7 | 2.163 | 2.363 |
| **Latvia** | **GDPrLGiniLow** | -3.619 | 4.54 | 3.7 | 2.174 | 2.364 |
| **Latvia** | **NoCOVIDginiB** | 2.794 | 2.895 | 3.023 | 2.242 | 2.379 |
| **Lebanon** | **GDPrGiniBase** | -21.46 | 0 | 0 | 1.771 | 2.106 |
| **Lebanon** | **GDPrHGiniH** | -21.46 | 0 | 1.5 | 1.725 | 2.056 |
| **Lebanon** | **GDPrHGiniHv** | -21.46 | 0 | 1.5 | 1.718 | 2.057 |
| **Lebanon** | **GDPrHGiniLow** | -21.46 | 0 | 1.5 | 1.732 | 2.055 |
| **Lebanon** | **GDPrLGiniH** | -21.46 | 0 | -1.5 | 1.785 | 2.125 |
| **Lebanon** | **GDPrLGiniHv** | -21.46 | 0 | -1.5 | 1.778 | 2.126 |
| **Lebanon** | **GDPrLGiniLow** | -21.46 | 0 | -1.5 | 1.796 | 2.13 |
| **Lebanon** | **NoCOVIDginiB** | 0.863 | 2.336 | 2.57 | 1.737 | 1.649 |
| **Lesotho** | **GDPrGiniBase** | -9.614 | 2.811 | 1.621 | 2.834 | 4.458 |
| **Lesotho** | **GDPrHGiniH** | -9.614 | 2.811 | 3.121 | 2.896 | 4.445 |
| **Lesotho** | **GDPrHGiniHv** | -9.614 | 2.811 | 3.121 | 2.866 | 4.419 |
| **Lesotho** | **GDPrHGiniLow** | -9.614 | 2.811 | 3.121 | 2.937 | 4.478 |
| **Lesotho** | **GDPrLGiniH** | -9.614 | 2.811 | 0.121 | 2.727 | 4.435 |
| **Lesotho** | **GDPrLGiniHv** | -9.614 | 2.811 | 0.121 | 2.699 | 4.411 |
| **Lesotho** | **GDPrLGiniLow** | -9.614 | 2.811 | 0.121 | 2.766 | 4.469 |
| **Lesotho** | **NoCOVIDginiB** | -0.167 | 3.679 | 4.453 | 3.337 | 4.523 |
| **Liberia** | **GDPrGiniBase** | -2.983 | 3.561 | 4.724 | 3.42 | 7.659 |
| **Liberia** | **GDPrHGiniH** | -2.983 | 3.561 | 6.224 | 3.549 | 7.691 |
| **Liberia** | **GDPrHGiniHv** | -2.983 | 3.561 | 6.224 | 3.521 | 7.622 |
| **Liberia** | **GDPrHGiniLow** | -2.983 | 3.561 | 6.224 | 3.588 | 7.787 |
| **Liberia** | **GDPrLGiniH** | -2.983 | 3.561 | 3.224 | 3.182 | 7.474 |
| **Liberia** | **GDPrLGiniHv** | -2.983 | 3.561 | 3.224 | 3.155 | 7.402 |
| **Liberia** | **GDPrLGiniLow** | -2.983 | 3.561 | 3.224 | 3.221 | 7.576 |
| **Liberia** | **NoCOVIDginiB** | 1.553 | 1.254 | -1.266 | 2.911 | 7.409 |
| **Libya** | **GDPrGiniBase** | -31.3 | 123.2 | 5.252 | 5.54 | 2.95 |
| **Libya** | **GDPrHGiniH** | -31.3 | 123.2 | 6.752 | 5.592 | 2.954 |
| **Libya** | **GDPrHGiniHv** | -31.3 | 123.2 | 6.752 | 5.59 | 2.954 |
| **Libya** | **GDPrHGiniLow** | -31.3 | 123.2 | 6.752 | 5.594 | 2.954 |
| **Libya** | **GDPrLGiniH** | -31.3 | 123.2 | 3.752 | 5.629 | 2.963 |
| **Libya** | **GDPrLGiniHv** | -31.3 | 123.2 | 3.752 | 5.628 | 2.963 |
| **Libya** | **GDPrLGiniLow** | -31.3 | 123.2 | 3.752 | 5.63 | 2.963 |
| **Libya** | **NoCOVIDginiB** | -0.036 | 0 | 0 | 5.553 | 3.903 |
| **Lithuania** | **GDPrGiniBase** | -0.132 | 4.663 | 4.072 | 2.513 | 2.078 |
| **Lithuania** | **GDPrHGiniH** | -0.132 | 4.663 | 5.572 | 2.643 | 2.055 |
| **Lithuania** | **GDPrHGiniHv** | -0.132 | 4.663 | 5.572 | 2.639 | 2.055 |
| **Lithuania** | **GDPrHGiniLow** | -0.132 | 4.663 | 5.572 | 2.648 | 2.054 |
| **Lithuania** | **GDPrLGiniH** | -0.132 | 4.663 | 2.572 | 2.561 | 2.101 |
| **Lithuania** | **GDPrLGiniHv** | -0.132 | 4.663 | 2.572 | 2.558 | 2.102 |
| **Lithuania** | **GDPrLGiniLow** | -0.132 | 4.663 | 2.572 | 2.566 | 2.101 |
| **Lithuania** | **NoCOVIDginiB** | 2.718 | 2.462 | 2.387 | 2.644 | 2.111 |
| **Luxembourg** | **GDPrGiniBase** | -1.776 | 5.513 | 3.845 | 0.477 | 0.364 |
| **Luxembourg** | **GDPrHGiniH** | -1.776 | 5.513 | 5.345 | 0.426 | 0.387 |
| **Luxembourg** | **GDPrHGiniHv** | -1.776 | 5.513 | 5.345 | 0.425 | 0.387 |
| **Luxembourg** | **GDPrHGiniLow** | -1.776 | 5.513 | 5.345 | 0.427 | 0.388 |
| **Luxembourg** | **GDPrLGiniH** | -1.776 | 5.513 | 2.345 | 0.52 | 0.34 |
| **Luxembourg** | **GDPrLGiniHv** | -1.776 | 5.513 | 2.345 | 0.518 | 0.34 |
| **Luxembourg** | **GDPrLGiniLow** | -1.776 | 5.513 | 2.345 | 0.522 | 0.341 |
| **Luxembourg** | **NoCOVIDginiB** | 2.774 | 2.66 | 2.633 | 0.552 | 0.36 |
| **Macedonia, North** | **GDPrGiniBase** | -5.208 | 4 | 4.2 | 2.389 | 2.176 |
| **Macedonia, North** | **GDPrHGiniH** | -5.208 | 4 | 5.7 | 2.426 | 2.185 |
| **Macedonia, North** | **GDPrHGiniHv** | -5.208 | 4 | 5.7 | 2.422 | 2.182 |
| **Macedonia, North** | **GDPrHGiniLow** | -5.208 | 4 | 5.7 | 2.43 | 2.187 |
| **Macedonia, North** | **GDPrLGiniH** | -5.208 | 4 | 2.7 | 2.349 | 2.158 |
| **Macedonia, North** | **GDPrLGiniHv** | -5.208 | 4 | 2.7 | 2.345 | 2.156 |
| **Macedonia, North** | **GDPrLGiniLow** | -5.208 | 4 | 2.7 | 2.353 | 2.159 |
| **Macedonia, North** | **NoCOVIDginiB** | 3.355 | 3.2 | 3.301 | 2.557 | 2.167 |
| **Madagascar** | **GDPrGiniBase** | -7.141 | 2.913 | 4.755 | 3.469 | 4.744 |
| **Madagascar** | **GDPrHGiniH** | -7.141 | 2.913 | 6.255 | 3.657 | 4.76 |
| **Madagascar** | **GDPrHGiniHv** | -7.141 | 2.913 | 6.255 | 3.641 | 4.71 |
| **Madagascar** | **GDPrHGiniLow** | -7.141 | 2.913 | 6.255 | 3.683 | 4.832 |
| **Madagascar** | **GDPrLGiniH** | -7.141 | 2.913 | 3.255 | 3.306 | 4.614 |
| **Madagascar** | **GDPrLGiniHv** | -7.141 | 2.913 | 3.255 | 3.287 | 4.558 |
| **Madagascar** | **GDPrLGiniLow** | -7.141 | 2.913 | 3.255 | 3.334 | 4.684 |
| **Madagascar** | **NoCOVIDginiB** | 5.309 | 5.139 | 4.854 | 4.714 | 5.372 |
| **Malawi** | **GDPrGiniBase** | 0.8 | 2.2 | 3 | 6.033 | 8.514 |
| **Malawi** | **GDPrHGiniH** | 0.8 | 2.2 | 4.5 | 6.152 | 8.542 |
| **Malawi** | **GDPrHGiniHv** | 0.8 | 2.2 | 4.5 | 6.125 | 8.489 |
| **Malawi** | **GDPrHGiniLow** | 0.8 | 2.2 | 4.5 | 6.191 | 8.613 |
| **Malawi** | **GDPrLGiniH** | 0.8 | 2.2 | 1.5 | 5.871 | 8.456 |
| **Malawi** | **GDPrLGiniHv** | 0.8 | 2.2 | 1.5 | 5.841 | 8.387 |
| **Malawi** | **GDPrLGiniLow** | 0.8 | 2.2 | 1.5 | 5.913 | 8.549 |
| **Malawi** | **NoCOVIDginiB** | 5.101 | 5.5 | 6 | 7.055 | 8.737 |
| **Malaysia** | **GDPrGiniBase** | -5.647 | 3.5 | 5.7 | 3.062 | 2.217 |
| **Malaysia** | **GDPrHGiniH** | -5.647 | 3.5 | 7.2 | 3.121 | 2.259 |
| **Malaysia** | **GDPrHGiniHv** | -5.647 | 3.5 | 7.2 | 3.118 | 2.259 |
| **Malaysia** | **GDPrHGiniLow** | -5.647 | 3.5 | 7.2 | 3.124 | 2.26 |
| **Malaysia** | **GDPrLGiniH** | -5.647 | 3.5 | 4.2 | 2.998 | 2.178 |
| **Malaysia** | **GDPrLGiniHv** | -5.647 | 3.5 | 4.2 | 2.996 | 2.178 |
| **Malaysia** | **GDPrLGiniLow** | -5.647 | 3.5 | 4.2 | 3.002 | 2.179 |
| **Malaysia** | **NoCOVIDginiB** | 4.4 | 4.9 | 4.84 | 3.314 | 2.242 |
| **Maldives** | **GDPrGiniBase** | -33.5 | 18.87 | 13.21 | 2.016 | -0.124 |
| **Maldives** | **GDPrHGiniH** | -33.5 | 18.87 | 14.71 | 2.095 | -0.14 |
| **Maldives** | **GDPrHGiniHv** | -33.5 | 18.87 | 14.71 | 2.091 | -0.141 |
| **Maldives** | **GDPrHGiniLow** | -33.5 | 18.87 | 14.71 | 2.098 | -0.141 |
| **Maldives** | **GDPrLGiniH** | -33.5 | 18.87 | 11.71 | 1.979 | -0.1 |
| **Maldives** | **GDPrLGiniHv** | -33.5 | 18.87 | 11.71 | 1.976 | -0.099 |
| **Maldives** | **GDPrLGiniLow** | -33.5 | 18.87 | 11.71 | 1.982 | -0.101 |
| **Maldives** | **NoCOVIDginiB** | 6.046 | 5.508 | 5.468 | 2.685 | -0.131 |
| **Mali** | **GDPrGiniBase** | -1.235 | 4.025 | 5.34 | 4.698 | 6.817 |
| **Mali** | **GDPrHGiniH** | -1.235 | 4.025 | 6.84 | 4.842 | 6.918 |
| **Mali** | **GDPrHGiniHv** | -1.235 | 4.025 | 6.84 | 4.823 | 6.873 |
| **Mali** | **GDPrHGiniLow** | -1.235 | 4.025 | 6.84 | 4.869 | 6.972 |
| **Mali** | **GDPrLGiniH** | -1.235 | 4.025 | 3.84 | 4.519 | 6.665 |
| **Mali** | **GDPrLGiniHv** | -1.235 | 4.025 | 3.84 | 4.501 | 6.618 |
| **Mali** | **GDPrLGiniLow** | -1.235 | 4.025 | 3.84 | 4.544 | 6.735 |
| **Mali** | **NoCOVIDginiB** | 5.001 | 4.9 | 4.9 | 5.156 | 7.029 |
| **Malta** | **GDPrGiniBase** | -7.001 | 5.711 | 6.004 | 2.645 | 1.03 |
| **Malta** | **GDPrHGiniH** | -7.001 | 5.711 | 7.504 | 2.622 | 1.004 |
| **Malta** | **GDPrHGiniHv** | -7.001 | 5.711 | 7.504 | 2.621 | 1.001 |
| **Malta** | **GDPrHGiniLow** | -7.001 | 5.711 | 7.504 | 2.625 | 1.004 |
| **Malta** | **GDPrLGiniH** | -7.001 | 5.711 | 4.504 | 2.67 | 1.054 |
| **Malta** | **GDPrLGiniHv** | -7.001 | 5.711 | 4.504 | 2.666 | 1.054 |
| **Malta** | **GDPrLGiniLow** | -7.001 | 5.711 | 4.504 | 2.673 | 1.054 |
| **Malta** | **NoCOVIDginiB** | 4.316 | 3.744 | 3.5 | 2.53 | 0.9 |
| **Mauritania** | **GDPrGiniBase** | -1.763 | 2.729 | 4.98 | 3.737 | 4.812 |
| **Mauritania** | **GDPrHGiniH** | -1.763 | 2.729 | 6.48 | 3.861 | 4.825 |
| **Mauritania** | **GDPrHGiniHv** | -1.763 | 2.729 | 6.48 | 3.85 | 4.817 |
| **Mauritania** | **GDPrHGiniLow** | -1.763 | 2.729 | 6.48 | 3.876 | 4.835 |
| **Mauritania** | **GDPrLGiniH** | -1.763 | 2.729 | 3.48 | 3.611 | 4.785 |
| **Mauritania** | **GDPrLGiniHv** | -1.763 | 2.729 | 3.48 | 3.6 | 4.777 |
| **Mauritania** | **GDPrLGiniLow** | -1.763 | 2.729 | 3.48 | 3.625 | 4.796 |
| **Mauritania** | **NoCOVIDginiB** | 5.879 | 5.851 | 9.165 | 4.359 | 4.907 |
| **Mauritius** | **GDPrGiniBase** | -14.89 | 5 | 6.7 | 1.039 | 1.888 |
| **Mauritius** | **GDPrHGiniH** | -14.89 | 5 | 8.2 | 1.118 | 1.92 |
| **Mauritius** | **GDPrHGiniHv** | -14.89 | 5 | 8.2 | 1.116 | 1.92 |
| **Mauritius** | **GDPrHGiniLow** | -14.89 | 5 | 8.2 | 1.121 | 1.919 |
| **Mauritius** | **GDPrLGiniH** | -14.89 | 5 | 5.2 | 0.957 | 1.834 |
| **Mauritius** | **GDPrLGiniHv** | -14.89 | 5 | 5.2 | 0.954 | 1.834 |
| **Mauritius** | **GDPrLGiniLow** | -14.89 | 5 | 5.2 | 0.96 | 1.833 |
| **Mauritius** | **NoCOVIDginiB** | 3.83 | 3.931 | 4.011 | 1.436 | 1.974 |
| **Mexico** | **GDPrGiniBase** | -8.309 | 5.3 | 2.8 | 1.332 | 1.081 |
| **Mexico** | **GDPrHGiniH** | -8.309 | 5.3 | 4.3 | 1.368 | 1.086 |
| **Mexico** | **GDPrHGiniHv** | -8.309 | 5.3 | 4.3 | 1.359 | 1.085 |
| **Mexico** | **GDPrHGiniLow** | -8.309 | 5.3 | 4.3 | 1.38 | 1.086 |
| **Mexico** | **GDPrLGiniH** | -8.309 | 5.3 | 1.3 | 1.29 | 1.074 |
| **Mexico** | **GDPrLGiniHv** | -8.309 | 5.3 | 1.3 | 1.28 | 1.074 |
| **Mexico** | **GDPrLGiniLow** | -8.309 | 5.3 | 1.3 | 1.301 | 1.074 |
| **Mexico** | **NoCOVIDginiB** | 1.307 | 1.899 | 2.108 | 1.465 | 1.073 |
| **Micronesia** | **GDPrGiniBase** | -1.763 | -3.248 | 0.586 | 0.539 | 1.132 |
| **Micronesia** | **GDPrHGiniH** | -1.763 | -3.248 | 2.086 | 0.681 | 1.214 |
| **Micronesia** | **GDPrHGiniHv** | -1.763 | -3.248 | 2.086 | 0.674 | 1.204 |
| **Micronesia** | **GDPrHGiniLow** | -1.763 | -3.248 | 2.086 | 0.694 | 1.221 |
| **Micronesia** | **GDPrLGiniH** | -1.763 | -3.248 | -0.914 | 0.38 | 1.022 |
| **Micronesia** | **GDPrLGiniHv** | -1.763 | -3.248 | -0.914 | 0.373 | 1.017 |
| **Micronesia** | **GDPrLGiniLow** | -1.763 | -3.248 | -0.914 | 0.393 | 1.031 |
| **Micronesia** | **NoCOVIDginiB** | 0.791 | 0.722 | 0.592 | 0.746 | 1.166 |
| **Moldova, Republic of** | **GDPrGiniBase** | -6.969 | 4.5 | 5.172 | 2.464 | 1.425 |
| **Moldova, Republic of** | **GDPrHGiniH** | -6.969 | 4.5 | 6.672 | 2.513 | 1.404 |
| **Moldova, Republic of** | **GDPrHGiniHv** | -6.969 | 4.5 | 6.672 | 2.509 | 1.403 |
| **Moldova, Republic of** | **GDPrHGiniLow** | -6.969 | 4.5 | 6.672 | 2.519 | 1.406 |
| **Moldova, Republic of** | **GDPrLGiniH** | -6.969 | 4.5 | 3.672 | 2.401 | 1.462 |
| **Moldova, Republic of** | **GDPrLGiniHv** | -6.969 | 4.5 | 3.672 | 2.396 | 1.462 |
| **Moldova, Republic of** | **GDPrLGiniLow** | -6.969 | 4.5 | 3.672 | 2.407 | 1.462 |
| **Moldova, Republic of** | **NoCOVIDginiB** | 3.8 | 3.799 | 3.801 | 2.738 | 1.397 |
| **Mongolia** | **GDPrGiniBase** | -4.558 | 5.2 | 7.5 | 6.348 | 5.472 |
| **Mongolia** | **GDPrHGiniH** | -4.558 | 5.2 | 9 | 6.372 | 5.429 |
| **Mongolia** | **GDPrHGiniHv** | -4.558 | 5.2 | 9 | 6.365 | 5.428 |
| **Mongolia** | **GDPrHGiniLow** | -4.558 | 5.2 | 9 | 6.382 | 5.431 |
| **Mongolia** | **GDPrLGiniH** | -4.558 | 5.2 | 6 | 6.332 | 5.508 |
| **Mongolia** | **GDPrLGiniHv** | -4.558 | 5.2 | 6 | 6.325 | 5.507 |
| **Mongolia** | **GDPrLGiniLow** | -4.558 | 5.2 | 6 | 6.342 | 5.509 |
| **Mongolia** | **NoCOVIDginiB** | 5.367 | 5.142 | 5.585 | 6.614 | 5.323 |
| **Montenegro** | **GDPrGiniBase** | -15.31 | 7 | 5.6 | 1.481 | 2.45 |
| **Montenegro** | **GDPrHGiniH** | -15.31 | 7 | 7.1 | 1.568 | 2.443 |
| **Montenegro** | **GDPrHGiniHv** | -15.31 | 7 | 7.1 | 1.563 | 2.442 |
| **Montenegro** | **GDPrHGiniLow** | -15.31 | 7 | 7.1 | 1.575 | 2.444 |
| **Montenegro** | **GDPrLGiniH** | -15.31 | 7 | 4.1 | 1.399 | 2.463 |
| **Montenegro** | **GDPrLGiniHv** | -15.31 | 7 | 4.1 | 1.393 | 2.463 |
| **Montenegro** | **GDPrLGiniLow** | -15.31 | 7 | 4.1 | 1.406 | 2.463 |
| **Montenegro** | **NoCOVIDginiB** | 2.478 | 2.915 | 3.175 | 1.903 | 2.386 |
| **Morocco** | **GDPrGiniBase** | -6.293 | 5.732 | 3.129 | 2.667 | 2.57 |
| **Morocco** | **GDPrHGiniH** | -6.293 | 5.732 | 4.629 | 2.726 | 2.545 |
| **Morocco** | **GDPrHGiniHv** | -6.293 | 5.732 | 4.629 | 2.72 | 2.541 |
| **Morocco** | **GDPrHGiniLow** | -6.293 | 5.732 | 4.629 | 2.732 | 2.55 |
| **Morocco** | **GDPrLGiniH** | -6.293 | 5.732 | 1.629 | 2.592 | 2.582 |
| **Morocco** | **GDPrLGiniHv** | -6.293 | 5.732 | 1.629 | 2.586 | 2.577 |
| **Morocco** | **GDPrLGiniLow** | -6.293 | 5.732 | 1.629 | 2.599 | 2.588 |
| **Morocco** | **NoCOVIDginiB** | 3.688 | 4.13 | 4.299 | 2.926 | 2.541 |
| **Mozambique** | **GDPrGiniBase** | -1.234 | 2.458 | 5.298 | 5.301 | 6.819 |
| **Mozambique** | **GDPrHGiniH** | -1.234 | 2.458 | 6.798 | 5.395 | 6.74 |
| **Mozambique** | **GDPrHGiniHv** | -1.234 | 2.458 | 6.798 | 5.364 | 6.666 |
| **Mozambique** | **GDPrHGiniLow** | -1.234 | 2.458 | 6.798 | 5.436 | 6.837 |
| **Mozambique** | **GDPrLGiniH** | -1.234 | 2.458 | 3.798 | 5.165 | 6.792 |
| **Mozambique** | **GDPrLGiniHv** | -1.234 | 2.458 | 3.798 | 5.135 | 6.719 |
| **Mozambique** | **GDPrLGiniLow** | -1.234 | 2.458 | 3.798 | 5.208 | 6.888 |
| **Mozambique** | **NoCOVIDginiB** | 6.043 | 4 | 4 | 5.638 | 6.723 |
| **Myanmar** | **GDPrGiniBase** | 3.174 | -17.94 | -0.059 | 2.634 | 2.442 |
| **Myanmar** | **GDPrHGiniH** | 3.174 | -17.94 | 1.441 | 2.714 | 2.472 |
| **Myanmar** | **GDPrHGiniHv** | 3.174 | -17.94 | 1.441 | 2.709 | 2.465 |
| **Myanmar** | **GDPrHGiniLow** | 3.174 | -17.94 | 1.441 | 2.719 | 2.482 |
| **Myanmar** | **GDPrLGiniH** | 3.174 | -17.94 | -1.559 | 2.573 | 2.399 |
| **Myanmar** | **GDPrLGiniHv** | 3.174 | -17.94 | -1.559 | 2.569 | 2.392 |
| **Myanmar** | **GDPrLGiniLow** | 3.174 | -17.94 | -1.559 | 2.579 | 2.41 |
| **Myanmar** | **NoCOVIDginiB** | 6.259 | 6.013 | 6.126 | 3.685 | 2.771 |
| **Namibia** | **GDPrGiniBase** | -8.5 | 1.326 | 3.563 | 4.218 | 6.047 |
| **Namibia** | **GDPrHGiniH** | -8.5 | 1.326 | 5.063 | 4.3 | 6.004 |
| **Namibia** | **GDPrHGiniHv** | -8.5 | 1.326 | 5.063 | 4.284 | 6.004 |
| **Namibia** | **GDPrHGiniLow** | -8.5 | 1.326 | 5.063 | 4.321 | 6.004 |
| **Namibia** | **GDPrLGiniH** | -8.5 | 1.326 | 2.063 | 4.099 | 6.076 |
| **Namibia** | **GDPrLGiniHv** | -8.5 | 1.326 | 2.063 | 4.083 | 6.076 |
| **Namibia** | **GDPrLGiniLow** | -8.5 | 1.326 | 2.063 | 4.12 | 6.076 |
| **Namibia** | **NoCOVIDginiB** | 1.551 | 2.433 | 3.202 | 4.826 | 5.857 |
| **Nepal** | **GDPrGiniBase** | -2.088 | 1.803 | 4.403 | 3.077 | 3.418 |
| **Nepal** | **GDPrHGiniH** | -2.088 | 1.803 | 5.903 | 3.173 | 3.473 |
| **Nepal** | **GDPrHGiniHv** | -2.088 | 1.803 | 5.903 | 3.161 | 3.459 |
| **Nepal** | **GDPrHGiniLow** | -2.088 | 1.803 | 5.903 | 3.199 | 3.492 |
| **Nepal** | **GDPrLGiniH** | -2.088 | 1.803 | 2.903 | 2.966 | 3.366 |
| **Nepal** | **GDPrLGiniHv** | -2.088 | 1.803 | 2.903 | 2.954 | 3.351 |
| **Nepal** | **GDPrLGiniLow** | -2.088 | 1.803 | 2.903 | 2.981 | 3.387 |
| **Nepal** | **NoCOVIDginiB** | 6.27 | 5.759 | 5.295 | 3.465 | 3.644 |
| **Netherlands** | **GDPrGiniBase** | -3.799 | 4.5 | 3.3 | 0.719 | 0.985 |
| **Netherlands** | **GDPrHGiniH** | -3.799 | 4.5 | 4.8 | 0.717 | 0.965 |
| **Netherlands** | **GDPrHGiniHv** | -3.799 | 4.5 | 4.8 | 0.716 | 0.965 |
| **Netherlands** | **GDPrHGiniLow** | -3.799 | 4.5 | 4.8 | 0.719 | 0.965 |
| **Netherlands** | **GDPrLGiniH** | -3.799 | 4.5 | 1.8 | 0.715 | 1.007 |
| **Netherlands** | **GDPrLGiniHv** | -3.799 | 4.5 | 1.8 | 0.714 | 1.007 |
| **Netherlands** | **GDPrLGiniLow** | -3.799 | 4.5 | 1.8 | 0.717 | 1.007 |
| **Netherlands** | **NoCOVIDginiB** | 1.639 | 1.526 | 1.489 | 0.742 | 0.971 |
| **New Zealand** | **GDPrGiniBase** | 1.863 | 5.058 | 3.328 | 2.188 | 1.857 |
| **New Zealand** | **GDPrHGiniH** | 1.863 | 5.058 | 4.828 | 2.108 | 1.85 |
| **New Zealand** | **GDPrHGiniHv** | 1.863 | 5.058 | 4.828 | 2.106 | 1.851 |
| **New Zealand** | **GDPrHGiniLow** | 1.863 | 5.058 | 4.828 | 2.11 | 1.849 |
| **New Zealand** | **GDPrLGiniH** | 1.863 | 5.058 | 1.828 | 2.24 | 1.863 |
| **New Zealand** | **GDPrLGiniHv** | 1.863 | 5.058 | 1.828 | 2.238 | 1.864 |
| **New Zealand** | **GDPrLGiniLow** | 1.863 | 5.058 | 1.828 | 2.243 | 1.862 |
| **New Zealand** | **NoCOVIDginiB** | 2.699 | 2.62 | 2.595 | 2.354 | 1.849 |
| **Nicaragua** | **GDPrGiniBase** | -1.977 | 5 | 3.5 | 2.586 | 2.377 |
| **Nicaragua** | **GDPrHGiniH** | -1.977 | 5 | 5 | 2.657 | 2.38 |
| **Nicaragua** | **GDPrHGiniHv** | -1.977 | 5 | 5 | 2.646 | 2.374 |
| **Nicaragua** | **GDPrHGiniLow** | -1.977 | 5 | 5 | 2.671 | 2.387 |
| **Nicaragua** | **GDPrLGiniH** | -1.977 | 5 | 2 | 2.494 | 2.357 |
| **Nicaragua** | **GDPrLGiniHv** | -1.977 | 5 | 2 | 2.482 | 2.353 |
| **Nicaragua** | **GDPrLGiniLow** | -1.977 | 5 | 2 | 2.509 | 2.364 |
| **Nicaragua** | **NoCOVIDginiB** | -0.8 | -0.1 | 0.445 | 2.268 | 2.267 |
| **Niger** | **GDPrGiniBase** | 3.58 | 5.36 | 6.583 | 6.47 | 8.843 |
| **Niger** | **GDPrHGiniH** | 3.58 | 5.36 | 8.083 | 6.63 | 8.841 |
| **Niger** | **GDPrHGiniHv** | 3.58 | 5.36 | 8.083 | 6.603 | 8.788 |
| **Niger** | **GDPrHGiniLow** | 3.58 | 5.36 | 8.083 | 6.668 | 8.926 |
| **Niger** | **GDPrLGiniH** | 3.58 | 5.36 | 5.083 | 6.266 | 8.774 |
| **Niger** | **GDPrLGiniHv** | 3.58 | 5.36 | 5.083 | 6.24 | 8.708 |
| **Niger** | **GDPrLGiniLow** | 3.58 | 5.36 | 5.083 | 6.303 | 8.844 |
| **Niger** | **NoCOVIDginiB** | 6.051 | 5.594 | 11.89 | 6.794 | 8.88 |
| **Nigeria** | **GDPrGiniBase** | -1.794 | 3 | 2.7 | 4.537 | 6.166 |
| **Nigeria** | **GDPrHGiniH** | -1.794 | 3 | 4.2 | 4.603 | 6.17 |
| **Nigeria** | **GDPrHGiniHv** | -1.794 | 3 | 4.2 | 4.59 | 6.159 |
| **Nigeria** | **GDPrHGiniLow** | -1.794 | 3 | 4.2 | 4.62 | 6.183 |
| **Nigeria** | **GDPrLGiniH** | -1.794 | 3 | 1.2 | 4.461 | 6.149 |
| **Nigeria** | **GDPrLGiniHv** | -1.794 | 3 | 1.2 | 4.448 | 6.139 |
| **Nigeria** | **GDPrLGiniLow** | -1.794 | 3 | 1.2 | 4.479 | 6.163 |
| **Nigeria** | **NoCOVIDginiB** | 2.521 | 2.516 | 2.731 | 4.71 | 6.202 |
| **Norway** | **GDPrGiniBase** | -0.766 | 3.026 | 4.068 | 1.24 | 1.123 |
| **Norway** | **GDPrHGiniH** | -0.766 | 3.026 | 5.568 | 1.21 | 1.122 |
| **Norway** | **GDPrHGiniHv** | -0.766 | 3.026 | 5.568 | 1.21 | 1.121 |
| **Norway** | **GDPrHGiniLow** | -0.766 | 3.026 | 5.568 | 1.211 | 1.123 |
| **Norway** | **GDPrLGiniH** | -0.766 | 3.026 | 2.568 | 1.273 | 1.114 |
| **Norway** | **GDPrLGiniHv** | -0.766 | 3.026 | 2.568 | 1.273 | 1.113 |
| **Norway** | **GDPrLGiniLow** | -0.766 | 3.026 | 2.568 | 1.273 | 1.114 |
| **Norway** | **NoCOVIDginiB** | 2.441 | 1.577 | 1.631 | 1.271 | 1.117 |
| **Oman** | **GDPrGiniBase** | -2.835 | 2.483 | 2.928 | 1.644 | -0.48 |
| **Oman** | **GDPrHGiniH** | -2.835 | 2.483 | 4.428 | 1.647 | -0.44 |
| **Oman** | **GDPrHGiniHv** | -2.835 | 2.483 | 4.428 | 1.646 | -0.44 |
| **Oman** | **GDPrHGiniLow** | -2.835 | 2.483 | 4.428 | 1.649 | -0.44 |
| **Oman** | **GDPrLGiniH** | -2.835 | 2.483 | 1.428 | 1.656 | -0.525 |
| **Oman** | **GDPrLGiniHv** | -2.835 | 2.483 | 1.428 | 1.654 | -0.526 |
| **Oman** | **GDPrLGiniLow** | -2.835 | 2.483 | 1.428 | 1.658 | -0.525 |
| **Oman** | **NoCOVIDginiB** | 3.681 | 4.321 | 0.897 | 1.896 | -0.479 |
| **Pakistan** | **GDPrGiniBase** | -0.935 | 3.9 | 4 | 3.338 | 4.34 |
| **Pakistan** | **GDPrHGiniH** | -0.935 | 3.9 | 5.5 | 3.41 | 4.346 |
| **Pakistan** | **GDPrHGiniHv** | -0.935 | 3.9 | 5.5 | 3.4 | 4.336 |
| **Pakistan** | **GDPrHGiniLow** | -0.935 | 3.9 | 5.5 | 3.424 | 4.355 |
| **Pakistan** | **GDPrLGiniH** | -0.935 | 3.9 | 2.5 | 3.244 | 4.303 |
| **Pakistan** | **GDPrLGiniHv** | -0.935 | 3.9 | 2.5 | 3.234 | 4.292 |
| **Pakistan** | **GDPrLGiniLow** | -0.935 | 3.9 | 2.5 | 3.258 | 4.317 |
| **Pakistan** | **NoCOVIDginiB** | 2.35 | 3.001 | 4.528 | 3.482 | 4.349 |
| **Palestine** | **GDPrGiniBase** | -11.46 | 4.44 | 6 | 4.295 | 6.145 |
| **Palestine** | **GDPrHGiniH** | -11.46 | 4.44 | 7.5 | 4.434 | 6.088 |
| **Palestine** | **GDPrHGiniHv** | -11.46 | 4.44 | 7.5 | 4.429 | 6.088 |
| **Palestine** | **GDPrHGiniLow** | -11.46 | 4.44 | 7.5 | 4.439 | 6.09 |
| **Palestine** | **GDPrLGiniH** | -11.46 | 4.44 | 4.5 | 4.146 | 6.26 |
| **Palestine** | **GDPrLGiniHv** | -11.46 | 4.44 | 4.5 | 4.142 | 6.26 |
| **Palestine** | **GDPrLGiniLow** | -11.46 | 4.44 | 4.5 | 4.152 | 6.261 |
| **Palestine** | **NoCOVIDginiB** | 0 | 0 | 0 | 4.2 | 6.157 |
| **Panama** | **GDPrGiniBase** | -17.94 | 11.95 | 5 | 3.081 | 2.483 |
| **Panama** | **GDPrHGiniH** | -17.94 | 11.95 | 6.5 | 3.137 | 2.467 |
| **Panama** | **GDPrHGiniHv** | -17.94 | 11.95 | 6.5 | 3.127 | 2.468 |
| **Panama** | **GDPrHGiniLow** | -17.94 | 11.95 | 6.5 | 3.149 | 2.467 |
| **Panama** | **GDPrLGiniH** | -17.94 | 11.95 | 3.5 | 3.005 | 2.492 |
| **Panama** | **GDPrLGiniHv** | -17.94 | 11.95 | 3.5 | 2.995 | 2.492 |
| **Panama** | **GDPrLGiniLow** | -17.94 | 11.95 | 3.5 | 3.018 | 2.493 |
| **Panama** | **NoCOVIDginiB** | 5.5 | 5.49 | 5.5 | 3.473 | 2.333 |
| **Papua New Guinea** | **GDPrGiniBase** | -3.5 | 1.215 | 4.015 | 4.106 | 4.851 |
| **Papua New Guinea** | **GDPrHGiniH** | -3.5 | 1.215 | 5.515 | 4.161 | 4.841 |
| **Papua New Guinea** | **GDPrHGiniHv** | -3.5 | 1.215 | 5.515 | 4.143 | 4.835 |
| **Papua New Guinea** | **GDPrHGiniLow** | -3.5 | 1.215 | 5.515 | 4.186 | 4.852 |
| **Papua New Guinea** | **GDPrLGiniH** | -3.5 | 1.215 | 2.515 | 4.022 | 4.843 |
| **Papua New Guinea** | **GDPrLGiniHv** | -3.5 | 1.215 | 2.515 | 4.003 | 4.833 |
| **Papua New Guinea** | **GDPrLGiniLow** | -3.5 | 1.215 | 2.515 | 4.047 | 4.855 |
| **Papua New Guinea** | **NoCOVIDginiB** | 2.562 | 2.521 | 3.052 | 4.254 | 4.881 |
| **Paraguay** | **GDPrGiniBase** | -0.566 | 4.5 | 3.8 | 3.006 | 2.586 |
| **Paraguay** | **GDPrHGiniH** | -0.566 | 4.5 | 5.3 | 3.032 | 2.591 |
| **Paraguay** | **GDPrHGiniHv** | -0.566 | 4.5 | 5.3 | 3.026 | 2.59 |
| **Paraguay** | **GDPrHGiniLow** | -0.566 | 4.5 | 5.3 | 3.041 | 2.591 |
| **Paraguay** | **GDPrLGiniH** | -0.566 | 4.5 | 2.3 | 2.971 | 2.574 |
| **Paraguay** | **GDPrLGiniHv** | -0.566 | 4.5 | 2.3 | 2.964 | 2.573 |
| **Paraguay** | **GDPrLGiniLow** | -0.566 | 4.5 | 2.3 | 2.98 | 2.574 |
| **Paraguay** | **NoCOVIDginiB** | 4.027 | 4.209 | 4.038 | 3.108 | 2.578 |
| **Peru** | **GDPrGiniBase** | -11.15 | 10.04 | 4.562 | 2.578 | 2.13 |
| **Peru** | **GDPrHGiniH** | -11.15 | 10.04 | 6.062 | 2.58 | 2.125 |
| **Peru** | **GDPrHGiniHv** | -11.15 | 10.04 | 6.062 | 2.574 | 2.125 |
| **Peru** | **GDPrHGiniLow** | -11.15 | 10.04 | 6.062 | 2.586 | 2.126 |
| **Peru** | **GDPrLGiniH** | -11.15 | 10.04 | 3.062 | 2.565 | 2.132 |
| **Peru** | **GDPrLGiniHv** | -11.15 | 10.04 | 3.062 | 2.559 | 2.132 |
| **Peru** | **GDPrLGiniLow** | -11.15 | 10.04 | 3.062 | 2.574 | 2.133 |
| **Peru** | **NoCOVIDginiB** | 3.619 | 4.001 | 3.994 | 2.629 | 2.121 |
| **Philippines** | **GDPrGiniBase** | -9.573 | 4.6 | 6.3 | 3.902 | 3.631 |
| **Philippines** | **GDPrHGiniH** | -9.573 | 4.6 | 7.8 | 3.944 | 3.609 |
| **Philippines** | **GDPrHGiniHv** | -9.573 | 4.6 | 7.8 | 3.932 | 3.608 |
| **Philippines** | **GDPrHGiniLow** | -9.573 | 4.6 | 7.8 | 3.96 | 3.61 |
| **Philippines** | **GDPrLGiniH** | -9.573 | 4.6 | 4.8 | 3.848 | 3.634 |
| **Philippines** | **GDPrLGiniHv** | -9.573 | 4.6 | 4.8 | 3.836 | 3.633 |
| **Philippines** | **GDPrLGiniLow** | -9.573 | 4.6 | 4.8 | 3.864 | 3.637 |
| **Philippines** | **NoCOVIDginiB** | 6.183 | 6.395 | 6.47 | 4.364 | 3.51 |
| **Poland** | **GDPrGiniBase** | -2.541 | 5.4 | 4.6 | 2.784 | 1.227 |
| **Poland** | **GDPrHGiniH** | -2.541 | 5.4 | 6.1 | 2.838 | 1.247 |
| **Poland** | **GDPrHGiniHv** | -2.541 | 5.4 | 6.1 | 2.836 | 1.246 |
| **Poland** | **GDPrHGiniLow** | -2.541 | 5.4 | 6.1 | 2.84 | 1.248 |
| **Poland** | **GDPrLGiniH** | -2.541 | 5.4 | 3.1 | 2.728 | 1.23 |
| **Poland** | **GDPrLGiniHv** | -2.541 | 5.4 | 3.1 | 2.726 | 1.23 |
| **Poland** | **GDPrLGiniLow** | -2.541 | 5.4 | 3.1 | 2.731 | 1.231 |
| **Poland** | **NoCOVIDginiB** | 3.083 | 2.69 | 2.526 | 2.812 | 1.202 |
| **Portugal** | **GDPrGiniBase** | -8.442 | 4.442 | 5.1 | 0.543 | 0.877 |
| **Portugal** | **GDPrHGiniH** | -8.442 | 4.442 | 6.6 | 0.566 | 0.887 |
| **Portugal** | **GDPrHGiniHv** | -8.442 | 4.442 | 6.6 | 0.563 | 0.887 |
| **Portugal** | **GDPrHGiniLow** | -8.442 | 4.442 | 6.6 | 0.568 | 0.888 |
| **Portugal** | **GDPrLGiniH** | -8.442 | 4.442 | 3.6 | 0.506 | 0.872 |
| **Portugal** | **GDPrLGiniHv** | -8.442 | 4.442 | 3.6 | 0.503 | 0.872 |
| **Portugal** | **GDPrLGiniLow** | -8.442 | 4.442 | 3.6 | 0.509 | 0.873 |
| **Portugal** | **NoCOVIDginiB** | 1.6 | 1.5 | 1.5 | 0.578 | 0.86 |
| **Puerto Rico** | **GDPrGiniBase** | -3.903 | -0.6 | -0.3 | 3.944 | 1.216 |
| **Puerto Rico** | **GDPrHGiniH** | -3.903 | -0.6 | 1.2 | 3.905 | 1.188 |
| **Puerto Rico** | **GDPrHGiniHv** | -3.903 | -0.6 | 1.2 | 3.896 | 1.19 |
| **Puerto Rico** | **GDPrHGiniLow** | -3.903 | -0.6 | 1.2 | 3.917 | 1.187 |
| **Puerto Rico** | **GDPrLGiniH** | -3.903 | -0.6 | -1.8 | 3.97 | 1.243 |
| **Puerto Rico** | **GDPrLGiniHv** | -3.903 | -0.6 | -1.8 | 3.961 | 1.242 |
| **Puerto Rico** | **GDPrLGiniLow** | -3.903 | -0.6 | -1.8 | 3.982 | 1.242 |
| **Puerto Rico** | **NoCOVIDginiB** | -0.693 | -0.775 | -0.775 | 3.897 | 1.151 |
| **Qatar** | **GDPrGiniBase** | -3.558 | 1.947 | 3.959 | 2.198 | -0.796 |
| **Qatar** | **GDPrHGiniH** | -3.558 | 1.947 | 5.459 | 2.237 | -0.756 |
| **Qatar** | **GDPrHGiniHv** | -3.558 | 1.947 | 5.459 | 2.236 | -0.756 |
| **Qatar** | **GDPrHGiniLow** | -3.558 | 1.947 | 5.459 | 2.239 | -0.755 |
| **Qatar** | **GDPrLGiniH** | -3.558 | 1.947 | 2.459 | 2.17 | -0.813 |
| **Qatar** | **GDPrLGiniHv** | -3.558 | 1.947 | 2.459 | 2.169 | -0.814 |
| **Qatar** | **GDPrLGiniLow** | -3.558 | 1.947 | 2.459 | 2.172 | -0.812 |
| **Qatar** | **NoCOVIDginiB** | 2.755 | 3.022 | 2.358 | 2.314 | -0.883 |
| **Romania** | **GDPrGiniBase** | -3.93 | 7 | 4.83 | 2.619 | 1.855 |
| **Romania** | **GDPrHGiniH** | -3.93 | 7 | 6.33 | 2.731 | 1.852 |
| **Romania** | **GDPrHGiniHv** | -3.93 | 7 | 6.33 | 2.778 | 1.92 |
| **Romania** | **GDPrHGiniLow** | -3.93 | 7 | 6.33 | 2.696 | 1.823 |
| **Romania** | **GDPrLGiniH** | -3.93 | 7 | 3.33 | 2.581 | 1.864 |
| **Romania** | **GDPrLGiniHv** | -3.93 | 7 | 3.33 | 2.625 | 1.948 |
| **Romania** | **GDPrLGiniLow** | -3.93 | 7 | 3.33 | 2.543 | 1.801 |
| **Romania** | **NoCOVIDginiB** | 3.5 | 3 | 3 | 2.673 | 1.83 |
| **Russian Federation** | **GDPrGiniBase** | -2.951 | 4.5 | 2.8 | 1.83 | 0.538 |
| **Russian Federation** | **GDPrHGiniH** | -2.951 | 4.5 | 4.3 | 1.838 | 0.464 |
| **Russian Federation** | **GDPrHGiniHv** | -2.951 | 4.5 | 4.3 | 1.83 | 0.463 |
| **Russian Federation** | **GDPrHGiniLow** | -2.951 | 4.5 | 4.3 | 1.848 | 0.465 |
| **Russian Federation** | **GDPrLGiniH** | -2.951 | 4.5 | 1.3 | 1.787 | 0.468 |
| **Russian Federation** | **GDPrLGiniHv** | -2.951 | 4.5 | 1.3 | 1.78 | 0.468 |
| **Russian Federation** | **GDPrLGiniLow** | -2.951 | 4.5 | 1.3 | 1.797 | 0.467 |
| **Russian Federation** | **NoCOVIDginiB** | 1.87 | 2.045 | 2.049 | 1.807 | 0.443 |
| **Rwanda** | **GDPrGiniBase** | -3.355 | 5.104 | 7.002 | 5.809 | 7.445 |
| **Rwanda** | **GDPrHGiniH** | -3.355 | 5.104 | 8.502 | 5.98 | 7.405 |
| **Rwanda** | **GDPrHGiniHv** | -3.355 | 5.104 | 8.502 | 5.942 | 7.348 |
| **Rwanda** | **GDPrHGiniLow** | -3.355 | 5.104 | 8.502 | 6.031 | 7.482 |
| **Rwanda** | **GDPrLGiniH** | -3.355 | 5.104 | 5.502 | 5.572 | 7.403 |
| **Rwanda** | **GDPrLGiniHv** | -3.355 | 5.104 | 5.502 | 5.537 | 7.343 |
| **Rwanda** | **GDPrLGiniLow** | -3.355 | 5.104 | 5.502 | 5.627 | 7.482 |
| **Rwanda** | **NoCOVIDginiB** | 8.1 | 8.2 | 8 | 6.553 | 7.506 |
| **Samoa** | **GDPrGiniBase** | -2.592 | -7.226 | 1.048 | 3.049 | 3.753 |
| **Samoa** | **GDPrHGiniH** | -2.592 | -7.226 | 2.548 | 3.121 | 3.708 |
| **Samoa** | **GDPrHGiniHv** | -2.592 | -7.226 | 2.548 | 3.111 | 3.707 |
| **Samoa** | **GDPrHGiniLow** | -2.592 | -7.226 | 2.548 | 3.138 | 3.712 |
| **Samoa** | **GDPrLGiniH** | -2.592 | -7.226 | -0.452 | 2.94 | 3.786 |
| **Samoa** | **GDPrLGiniHv** | -2.592 | -7.226 | -0.452 | 2.93 | 3.783 |
| **Samoa** | **GDPrLGiniLow** | -2.592 | -7.226 | -0.452 | 2.953 | 3.791 |
| **Samoa** | **NoCOVIDginiB** | 4.396 | 2.182 | 2.228 | 3.51 | 3.54 |
| **Sao Tome and Principe** | **GDPrGiniBase** | 3.088 | 2.132 | 2.906 | 5.767 | 6.726 |
| **Sao Tome and Principe** | **GDPrHGiniH** | 3.088 | 2.132 | 4.406 | 5.949 | 6.659 |
| **Sao Tome and Principe** | **GDPrHGiniHv** | 3.088 | 2.132 | 4.406 | 5.937 | 6.655 |
| **Sao Tome and Principe** | **GDPrHGiniLow** | 3.088 | 2.132 | 4.406 | 5.969 | 6.668 |
| **Sao Tome and Principe** | **GDPrLGiniH** | 3.088 | 2.132 | 1.406 | 5.565 | 6.786 |
| **Sao Tome and Principe** | **GDPrLGiniHv** | 3.088 | 2.132 | 1.406 | 5.55 | 6.777 |
| **Sao Tome and Principe** | **GDPrLGiniLow** | 3.088 | 2.132 | 1.406 | 5.588 | 6.794 |
| **Sao Tome and Principe** | **NoCOVIDginiB** | 3.499 | 3.99 | 4.511 | 6.152 | 6.652 |
| **Saudi Arabia** | **GDPrGiniBase** | -4.107 | 2.9 | 4.8 | 1.759 | 0.48 |
| **Saudi Arabia** | **GDPrHGiniH** | -4.107 | 2.9 | 6.3 | 1.778 | 0.674 |
| **Saudi Arabia** | **GDPrHGiniHv** | -4.107 | 2.9 | 6.3 | 1.776 | 0.674 |
| **Saudi Arabia** | **GDPrHGiniLow** | -4.107 | 2.9 | 6.3 | 1.78 | 0.675 |
| **Saudi Arabia** | **GDPrLGiniH** | -4.107 | 2.9 | 3.3 | 1.75 | 0.428 |
| **Saudi Arabia** | **GDPrLGiniHv** | -4.107 | 2.9 | 3.3 | 1.748 | 0.427 |
| **Saudi Arabia** | **GDPrLGiniLow** | -4.107 | 2.9 | 3.3 | 1.752 | 0.429 |
| **Saudi Arabia** | **NoCOVIDginiB** | 2.184 | 2.22 | 2.396 | 1.854 | 0.631 |
| **Senegal** | **GDPrGiniBase** | 1.5 | 4.747 | 5.504 | 6.369 | 7.05 |
| **Senegal** | **GDPrHGiniH** | 1.5 | 4.747 | 7.004 | 6.468 | 7.008 |
| **Senegal** | **GDPrHGiniHv** | 1.5 | 4.747 | 7.004 | 6.448 | 7.001 |
| **Senegal** | **GDPrHGiniLow** | 1.5 | 4.747 | 7.004 | 6.493 | 7.015 |
| **Senegal** | **GDPrLGiniH** | 1.5 | 4.747 | 4.004 | 6.242 | 7.091 |
| **Senegal** | **GDPrLGiniHv** | 1.5 | 4.747 | 4.004 | 6.224 | 7.085 |
| **Senegal** | **GDPrLGiniLow** | 1.5 | 4.747 | 4.004 | 6.267 | 7.097 |
| **Senegal** | **NoCOVIDginiB** | 6.751 | 6.959 | 8.375 | 6.83 | 6.861 |
| **Serbia** | **GDPrGiniBase** | -0.945 | 6.54 | 4.5 | 2.984 | 2.243 |
| **Serbia** | **GDPrHGiniH** | -0.945 | 6.54 | 6 | 2.999 | 2.251 |
| **Serbia** | **GDPrHGiniHv** | -0.945 | 6.54 | 6 | 2.994 | 2.247 |
| **Serbia** | **GDPrHGiniLow** | -0.945 | 6.54 | 6 | 3.006 | 2.257 |
| **Serbia** | **GDPrLGiniH** | -0.945 | 6.54 | 3 | 2.962 | 2.225 |
| **Serbia** | **GDPrLGiniHv** | -0.945 | 6.54 | 3 | 2.956 | 2.22 |
| **Serbia** | **GDPrLGiniLow** | -0.945 | 6.54 | 3 | 2.969 | 2.23 |
| **Serbia** | **NoCOVIDginiB** | 3.99 | 4 | 4 | 3.038 | 2.218 |
| **Seychelles** | **GDPrGiniBase** | -10.77 | 6.896 | 7.689 | 1.435 | 1.073 |
| **Seychelles** | **GDPrHGiniH** | -10.77 | 6.896 | 9.189 | 1.509 | 1.058 |
| **Seychelles** | **GDPrHGiniHv** | -10.77 | 6.896 | 9.189 | 1.505 | 1.051 |
| **Seychelles** | **GDPrHGiniLow** | -10.77 | 6.896 | 9.189 | 1.515 | 1.058 |
| **Seychelles** | **GDPrLGiniH** | -10.77 | 6.896 | 6.189 | 1.361 | 1.064 |
| **Seychelles** | **GDPrLGiniHv** | -10.77 | 6.896 | 6.189 | 1.356 | 1.064 |
| **Seychelles** | **GDPrLGiniLow** | -10.77 | 6.896 | 6.189 | 1.367 | 1.064 |
| **Seychelles** | **NoCOVIDginiB** | 3.314 | 4.138 | 4.023 | 1.608 | 1.047 |
| **Sierra Leone** | **GDPrGiniBase** | -1.969 | 3.194 | 5.884 | 4.122 | 6.379 |
| **Sierra Leone** | **GDPrHGiniH** | -1.969 | 3.194 | 7.384 | 4.25 | 6.49 |
| **Sierra Leone** | **GDPrHGiniHv** | -1.969 | 3.194 | 7.384 | 4.235 | 6.469 |
| **Sierra Leone** | **GDPrHGiniLow** | -1.969 | 3.194 | 7.384 | 4.271 | 6.524 |
| **Sierra Leone** | **GDPrLGiniH** | -1.969 | 3.194 | 4.384 | 3.971 | 6.295 |
| **Sierra Leone** | **GDPrLGiniHv** | -1.969 | 3.194 | 4.384 | 3.957 | 6.27 |
| **Sierra Leone** | **GDPrLGiniLow** | -1.969 | 3.194 | 4.384 | 3.99 | 6.33 |
| **Sierra Leone** | **NoCOVIDginiB** | 4.649 | 4.783 | 4.64 | 4.896 | 6.689 |
| **Singapore** | **GDPrGiniBase** | -5.391 | 6.029 | 3.22 | 2.091 | 0.888 |
| **Singapore** | **GDPrHGiniH** | -5.391 | 6.029 | 4.72 | 2.052 | 0.878 |
| **Singapore** | **GDPrHGiniHv** | -5.391 | 6.029 | 4.72 | 2.051 | 0.877 |
| **Singapore** | **GDPrHGiniLow** | -5.391 | 6.029 | 4.72 | 2.054 | 0.878 |
| **Singapore** | **GDPrLGiniH** | -5.391 | 6.029 | 1.72 | 2.086 | 0.892 |
| **Singapore** | **GDPrLGiniHv** | -5.391 | 6.029 | 1.72 | 2.087 | 0.891 |
| **Singapore** | **GDPrLGiniLow** | -5.391 | 6.029 | 1.72 | 2.085 | 0.892 |
| **Singapore** | **NoCOVIDginiB** | 0.993 | 1.573 | 2.216 | 2.052 | 0.753 |
| **Slovakia** | **GDPrGiniBase** | -4.359 | 4.432 | 5.192 | 1.632 | 0.384 |
| **Slovakia** | **GDPrHGiniH** | -4.359 | 4.432 | 6.692 | 1.698 | 0.403 |
| **Slovakia** | **GDPrHGiniHv** | -4.359 | 4.432 | 6.692 | 1.696 | 0.403 |
| **Slovakia** | **GDPrHGiniLow** | -4.359 | 4.432 | 6.692 | 1.7 | 0.404 |
| **Slovakia** | **GDPrLGiniH** | -4.359 | 4.432 | 3.692 | 1.571 | 0.361 |
| **Slovakia** | **GDPrLGiniHv** | -4.359 | 4.432 | 3.692 | 1.569 | 0.361 |
| **Slovakia** | **GDPrLGiniLow** | -4.359 | 4.432 | 3.692 | 1.573 | 0.361 |
| **Slovakia** | **NoCOVIDginiB** | 2.7 | 2.749 | 2.749 | 1.694 | 0.37 |
| **Slovenia** | **GDPrGiniBase** | -4.229 | 6.321 | 4.559 | 1.209 | 0.943 |
| **Slovenia** | **GDPrHGiniH** | -4.229 | 6.321 | 6.059 | 1.254 | 0.956 |
| **Slovenia** | **GDPrHGiniHv** | -4.229 | 6.321 | 6.059 | 1.253 | 0.956 |
| **Slovenia** | **GDPrHGiniLow** | -4.229 | 6.321 | 6.059 | 1.256 | 0.956 |
| **Slovenia** | **GDPrLGiniH** | -4.229 | 6.321 | 3.059 | 1.167 | 0.924 |
| **Slovenia** | **GDPrLGiniHv** | -4.229 | 6.321 | 3.059 | 1.165 | 0.924 |
| **Slovenia** | **GDPrLGiniLow** | -4.229 | 6.321 | 3.059 | 1.169 | 0.924 |
| **Slovenia** | **NoCOVIDginiB** | 2.936 | 2.734 | 2.307 | 1.256 | 0.918 |
| **Solomon Islands** | **GDPrGiniBase** | -4.317 | 1.229 | 4.395 | 3.993 | 5.032 |
| **Solomon Islands** | **GDPrHGiniH** | -4.317 | 1.229 | 5.895 | 4.117 | 5.07 |
| **Solomon Islands** | **GDPrHGiniHv** | -4.317 | 1.229 | 5.895 | 4.102 | 5.059 |
| **Solomon Islands** | **GDPrHGiniLow** | -4.317 | 1.229 | 5.895 | 4.135 | 5.085 |
| **Solomon Islands** | **GDPrLGiniH** | -4.317 | 1.229 | 2.895 | 3.847 | 4.978 |
| **Solomon Islands** | **GDPrLGiniHv** | -4.317 | 1.229 | 2.895 | 3.833 | 4.965 |
| **Solomon Islands** | **GDPrLGiniLow** | -4.317 | 1.229 | 2.895 | 3.865 | 4.993 |
| **Solomon Islands** | **NoCOVIDginiB** | 2.861 | 2.716 | 2.7 | 4.28 | 5.143 |
| **Somalia** | **GDPrGiniBase** | 2.398 | 1.6 | 3.861 | 6.071 | 11.51 |
| **Somalia** | **GDPrHGiniH** | 2.398 | 1.6 | 5.361 | 6.212 | 11.42 |
| **Somalia** | **GDPrHGiniHv** | 2.398 | 1.6 | 5.361 | 6.175 | 11.35 |
| **Somalia** | **GDPrHGiniLow** | 2.398 | 1.6 | 5.361 | 6.259 | 11.51 |
| **Somalia** | **GDPrLGiniH** | 2.398 | 1.6 | 2.361 | 5.845 | 11.4 |
| **Somalia** | **GDPrLGiniHv** | 2.398 | 1.6 | 2.361 | 5.806 | 11.31 |
| **Somalia** | **GDPrLGiniLow** | 2.398 | 1.6 | 2.361 | 5.895 | 11.52 |
| **Somalia** | **NoCOVIDginiB** | 3.201 | 3.499 | 3.501 | 6.359 | 11.46 |
| **South Africa** | **GDPrGiniBase** | -6.432 | 4.6 | 1.9 | 1.702 | 1.174 |
| **South Africa** | **GDPrHGiniH** | -6.432 | 4.6 | 3.4 | 1.69 | 1.163 |
| **South Africa** | **GDPrHGiniHv** | -6.432 | 4.6 | 3.4 | 1.664 | 1.159 |
| **South Africa** | **GDPrHGiniLow** | -6.432 | 4.6 | 3.4 | 1.727 | 1.166 |
| **South Africa** | **GDPrLGiniH** | -6.432 | 4.6 | 0.4 | 1.678 | 1.179 |
| **South Africa** | **GDPrLGiniHv** | -6.432 | 4.6 | 0.4 | 1.652 | 1.175 |
| **South Africa** | **GDPrLGiniLow** | -6.432 | 4.6 | 0.4 | 1.714 | 1.181 |
| **South Africa** | **NoCOVIDginiB** | 1.079 | 1.439 | 1.763 | 1.786 | 1.139 |
| **Spain** | **GDPrGiniBase** | -10.82 | 4.9 | 5.8 | 0.376 | 0.187 |
| **Spain** | **GDPrHGiniH** | -10.82 | 4.9 | 7.3 | 0.41 | 0.188 |
| **Spain** | **GDPrHGiniHv** | -10.82 | 4.9 | 7.3 | 0.409 | 0.188 |
| **Spain** | **GDPrHGiniLow** | -10.82 | 4.9 | 7.3 | 0.413 | 0.189 |
| **Spain** | **GDPrLGiniH** | -10.82 | 4.9 | 4.3 | 0.347 | 0.187 |
| **Spain** | **GDPrLGiniHv** | -10.82 | 4.9 | 4.3 | 0.346 | 0.187 |
| **Spain** | **GDPrLGiniLow** | -10.82 | 4.9 | 4.3 | 0.35 | 0.187 |
| **Spain** | **NoCOVIDginiB** | 1.847 | 1.702 | 1.671 | 0.41 | 0.153 |
| **Sri Lanka** | **GDPrGiniBase** | -3.569 | 3.647 | 3.296 | 2.736 | 2.91 |
| **Sri Lanka** | **GDPrHGiniH** | -3.569 | 3.647 | 4.796 | 2.768 | 2.91 |
| **Sri Lanka** | **GDPrHGiniHv** | -3.569 | 3.647 | 4.796 | 2.763 | 2.91 |
| **Sri Lanka** | **GDPrHGiniLow** | -3.569 | 3.647 | 4.796 | 2.773 | 2.908 |
| **Sri Lanka** | **GDPrLGiniH** | -3.569 | 3.647 | 1.796 | 2.701 | 2.907 |
| **Sri Lanka** | **GDPrLGiniHv** | -3.569 | 3.647 | 1.796 | 2.695 | 2.907 |
| **Sri Lanka** | **GDPrLGiniLow** | -3.569 | 3.647 | 1.796 | 2.708 | 2.904 |
| **Sri Lanka** | **NoCOVIDginiB** | 3.532 | 4.255 | 4.504 | 2.898 | 2.894 |
| **St. Lucia** | **GDPrGiniBase** | -20.37 | 3.475 | 13.14 | 2.481 | 1.444 |
| **St. Lucia** | **GDPrHGiniH** | -20.37 | 3.475 | 14.64 | 2.52 | 1.437 |
| **St. Lucia** | **GDPrHGiniHv** | -20.37 | 3.475 | 14.64 | 2.509 | 1.434 |
| **St. Lucia** | **GDPrHGiniLow** | -20.37 | 3.475 | 14.64 | 2.533 | 1.444 |
| **St. Lucia** | **GDPrLGiniH** | -20.37 | 3.475 | 11.64 | 2.428 | 1.452 |
| **St. Lucia** | **GDPrLGiniHv** | -20.37 | 3.475 | 11.64 | 2.414 | 1.45 |
| **St. Lucia** | **GDPrLGiniLow** | -20.37 | 3.475 | 11.64 | 2.442 | 1.454 |
| **St. Lucia** | **NoCOVIDginiB** | 3.218 | 2.993 | 2.397 | 2.571 | 1.433 |
| **St. Vincent and the Grenadines** | **GDPrGiniBase** | -3.253 | -6.071 | 8.33 | 2.111 | 2.103 |
| **St. Vincent and the Grenadines** | **GDPrHGiniH** | -3.253 | -6.071 | 9.83 | 2.15 | 2.087 |
| **St. Vincent and the Grenadines** | **GDPrHGiniHv** | -3.253 | -6.071 | 9.83 | 2.135 | 2.086 |
| **St. Vincent and the Grenadines** | **GDPrHGiniLow** | -3.253 | -6.071 | 9.83 | 2.168 | 2.088 |
| **St. Vincent and the Grenadines** | **GDPrLGiniH** | -3.253 | -6.071 | 6.83 | 2.023 | 2.119 |
| **St. Vincent and the Grenadines** | **GDPrLGiniHv** | -3.253 | -6.071 | 6.83 | 2.009 | 2.119 |
| **St. Vincent and the Grenadines** | **GDPrLGiniLow** | -3.253 | -6.071 | 6.83 | 2.042 | 2.124 |
| **St. Vincent and the Grenadines** | **NoCOVIDginiB** | 2.317 | 2.342 | 2.314 | 2.151 | 2.082 |
| **Sudan** | **GDPrGiniBase** | -3.63 | 0.9 | 3.5 | 3.583 | 5.246 |
| **Sudan** | **GDPrHGiniH** | -3.63 | 0.9 | 5 | 3.608 | 5.254 |
| **Sudan** | **GDPrHGiniHv** | -3.63 | 0.9 | 5 | 3.6 | 5.243 |
| **Sudan** | **GDPrHGiniLow** | -3.63 | 0.9 | 5 | 3.619 | 5.268 |
| **Sudan** | **GDPrLGiniH** | -3.63 | 0.9 | 2 | 3.536 | 5.18 |
| **Sudan** | **GDPrLGiniHv** | -3.63 | 0.9 | 2 | 3.528 | 5.169 |
| **Sudan** | **GDPrLGiniLow** | -3.63 | 0.9 | 2 | 3.547 | 5.195 |
| **Sudan** | **NoCOVIDginiB** | -1.514 | -1.065 | 0.163 | 3.342 | 5.089 |
| **Sudan South** | **GDPrGiniBase** | -6.589 | 5.325 | 6.473 | 6.152 | 8.897 |
| **Sudan South** | **GDPrHGiniH** | -6.589 | 5.325 | 7.973 | 6.367 | 8.963 |
| **Sudan South** | **GDPrHGiniHv** | -6.589 | 5.325 | 7.973 | 6.357 | 8.953 |
| **Sudan South** | **GDPrHGiniLow** | -6.589 | 5.325 | 7.973 | 6.381 | 8.974 |
| **Sudan South** | **GDPrLGiniH** | -6.589 | 5.325 | 4.973 | 5.977 | 8.721 |
| **Sudan South** | **GDPrLGiniHv** | -6.589 | 5.325 | 4.973 | 5.968 | 8.708 |
| **Sudan South** | **GDPrLGiniLow** | -6.589 | 5.325 | 4.973 | 5.99 | 8.738 |
| **Sudan South** | **NoCOVIDginiB** | 8.213 | 5.014 | 5.912 | 8.011 | 9.027 |
| **Suriname** | **GDPrGiniBase** | -15.91 | 0.684 | 1.528 | 1.094 | 1.817 |
| **Suriname** | **GDPrHGiniH** | -15.91 | 0.684 | 3.028 | 1.148 | 1.824 |
| **Suriname** | **GDPrHGiniHv** | -15.91 | 0.684 | 3.028 | 1.14 | 1.824 |
| **Suriname** | **GDPrHGiniLow** | -15.91 | 0.684 | 3.028 | 1.159 | 1.825 |
| **Suriname** | **GDPrLGiniH** | -15.91 | 0.684 | 0.028 | 1.062 | 1.814 |
| **Suriname** | **GDPrLGiniHv** | -15.91 | 0.684 | 0.028 | 1.054 | 1.813 |
| **Suriname** | **GDPrLGiniLow** | -15.91 | 0.684 | 0.028 | 1.072 | 1.814 |
| **Suriname** | **NoCOVIDginiB** | 2.501 | 2.398 | 2 | 1.47 | 1.828 |
| **Sweden** | **GDPrGiniBase** | -2.948 | 4.036 | 3.378 | 1.818 | 1.525 |
| **Sweden** | **GDPrHGiniH** | -2.948 | 4.036 | 4.878 | 1.821 | 1.516 |
| **Sweden** | **GDPrHGiniHv** | -2.948 | 4.036 | 4.878 | 1.821 | 1.515 |
| **Sweden** | **GDPrHGiniLow** | -2.948 | 4.036 | 4.878 | 1.821 | 1.516 |
| **Sweden** | **GDPrLGiniH** | -2.948 | 4.036 | 1.878 | 1.798 | 1.533 |
| **Sweden** | **GDPrLGiniHv** | -2.948 | 4.036 | 1.878 | 1.796 | 1.533 |
| **Sweden** | **GDPrLGiniLow** | -2.948 | 4.036 | 1.878 | 1.8 | 1.534 |
| **Sweden** | **NoCOVIDginiB** | 1.462 | 2.051 | 1.974 | 1.813 | 1.5 |
| **Switzerland** | **GDPrGiniBase** | -2.393 | 3.705 | 2.992 | 0.823 | 0.863 |
| **Switzerland** | **GDPrHGiniH** | -2.393 | 3.705 | 4.492 | 0.798 | 0.864 |
| **Switzerland** | **GDPrHGiniHv** | -2.393 | 3.705 | 4.492 | 0.798 | 0.864 |
| **Switzerland** | **GDPrHGiniLow** | -2.393 | 3.705 | 4.492 | 0.798 | 0.864 |
| **Switzerland** | **GDPrLGiniH** | -2.393 | 3.705 | 1.492 | 0.846 | 0.862 |
| **Switzerland** | **GDPrLGiniHv** | -2.393 | 3.705 | 1.492 | 0.847 | 0.861 |
| **Switzerland** | **GDPrLGiniLow** | -2.393 | 3.705 | 1.492 | 0.846 | 0.863 |
| **Switzerland** | **NoCOVIDginiB** | 1.26 | 1.62 | 1.588 | 0.834 | 0.848 |
| **Syrian Arab Republic** | **GDPrGiniBase** | 0 | 0 | 0 | 2.215 | 2.219 |
| **Syrian Arab Republic** | **GDPrHGiniH** | 0 | 0 | 1.5 | 2.255 | 2.255 |
| **Syrian Arab Republic** | **GDPrHGiniHv** | 0 | 0 | 1.5 | 2.25 | 2.254 |
| **Syrian Arab Republic** | **GDPrHGiniLow** | 0 | 0 | 1.5 | 2.26 | 2.257 |
| **Syrian Arab Republic** | **GDPrLGiniH** | 0 | 0 | -1.5 | 2.172 | 2.166 |
| **Syrian Arab Republic** | **GDPrLGiniHv** | 0 | 0 | -1.5 | 2.168 | 2.165 |
| **Syrian Arab Republic** | **GDPrLGiniLow** | 0 | 0 | -1.5 | 2.178 | 2.169 |
| **Syrian Arab Republic** | **NoCOVIDginiB** | 0 | 0 | 0 | 2.413 | 2.269 |
| **Taiwan** | **GDPrGiniBase** | 3.123 | 5.864 | 3.343 | 1.711 | 0.646 |
| **Taiwan** | **GDPrHGiniH** | 3.123 | 5.864 | 4.843 | 1.723 | 0.634 |
| **Taiwan** | **GDPrHGiniHv** | 3.123 | 5.864 | 4.843 | 1.721 | 0.635 |
| **Taiwan** | **GDPrHGiniLow** | 3.123 | 5.864 | 4.843 | 1.724 | 0.632 |
| **Taiwan** | **GDPrLGiniH** | 3.123 | 5.864 | 1.843 | 1.709 | 0.617 |
| **Taiwan** | **GDPrLGiniHv** | 3.123 | 5.864 | 1.843 | 1.708 | 0.616 |
| **Taiwan** | **GDPrLGiniLow** | 3.123 | 5.864 | 1.843 | 1.711 | 0.618 |
| **Taiwan** | **NoCOVIDginiB** | 1.933 | 2.094 | 2.116 | 1.879 | 0.627 |
| **Tajikistan** | **GDPrGiniBase** | 4.5 | 5 | 4.5 | 5.866 | 6.326 |
| **Tajikistan** | **GDPrHGiniH** | 4.5 | 5 | 6 | 5.933 | 6.305 |
| **Tajikistan** | **GDPrHGiniHv** | 4.5 | 5 | 6 | 5.929 | 6.305 |
| **Tajikistan** | **GDPrHGiniLow** | 4.5 | 5 | 6 | 5.937 | 6.306 |
| **Tajikistan** | **GDPrLGiniH** | 4.5 | 5 | 3 | 5.782 | 6.363 |
| **Tajikistan** | **GDPrLGiniHv** | 4.5 | 5 | 3 | 5.778 | 6.362 |
| **Tajikistan** | **GDPrLGiniLow** | 4.5 | 5 | 3 | 5.787 | 6.364 |
| **Tajikistan** | **NoCOVIDginiB** | 4.5 | 4.5 | 4.499 | 5.913 | 6.334 |
| **Tanzania** | **GDPrGiniBase** | 1.996 | 4.023 | 5.11 | 5.826 | 7.384 |
| **Tanzania** | **GDPrHGiniH** | 1.996 | 4.023 | 6.61 | 5.966 | 7.367 |
| **Tanzania** | **GDPrHGiniHv** | 1.996 | 4.023 | 6.61 | 5.942 | 7.334 |
| **Tanzania** | **GDPrHGiniLow** | 1.996 | 4.023 | 6.61 | 6.003 | 7.414 |
| **Tanzania** | **GDPrLGiniH** | 1.996 | 4.023 | 3.61 | 5.645 | 7.35 |
| **Tanzania** | **GDPrLGiniHv** | 1.996 | 4.023 | 3.61 | 5.622 | 7.317 |
| **Tanzania** | **GDPrLGiniLow** | 1.996 | 4.023 | 3.61 | 5.679 | 7.397 |
| **Tanzania** | **NoCOVIDginiB** | 5.704 | 5.99 | 6.293 | 6.186 | 7.432 |
| **Thailand** | **GDPrGiniBase** | -6.099 | 1.3 | 4.1 | 1.674 | 1.761 |
| **Thailand** | **GDPrHGiniH** | -6.099 | 1.3 | 5.6 | 1.713 | 1.779 |
| **Thailand** | **GDPrHGiniHv** | -6.099 | 1.3 | 5.6 | 1.709 | 1.782 |
| **Thailand** | **GDPrHGiniLow** | -6.099 | 1.3 | 5.6 | 1.717 | 1.773 |
| **Thailand** | **GDPrLGiniH** | -6.099 | 1.3 | 2.6 | 1.632 | 1.739 |
| **Thailand** | **GDPrLGiniHv** | -6.099 | 1.3 | 2.6 | 1.628 | 1.742 |
| **Thailand** | **GDPrLGiniLow** | -6.099 | 1.3 | 2.6 | 1.636 | 1.733 |
| **Thailand** | **NoCOVIDginiB** | 3.007 | 3.491 | 3.649 | 1.89 | 1.83 |
| **Timor-Leste** | **GDPrGiniBase** | 10.37 | 1.8 | 3.8 | 6.515 | 8.21 |
| **Timor-Leste** | **GDPrHGiniH** | 10.37 | 1.8 | 5.3 | 6.561 | 8.21 |
| **Timor-Leste** | **GDPrHGiniHv** | 10.37 | 1.8 | 5.3 | 6.554 | 8.207 |
| **Timor-Leste** | **GDPrHGiniLow** | 10.37 | 1.8 | 5.3 | 6.571 | 8.213 |
| **Timor-Leste** | **GDPrLGiniH** | 10.37 | 1.8 | 2.3 | 6.467 | 8.181 |
| **Timor-Leste** | **GDPrLGiniHv** | 10.37 | 1.8 | 2.3 | 6.459 | 8.155 |
| **Timor-Leste** | **GDPrLGiniLow** | 10.37 | 1.8 | 2.3 | 6.477 | 8.197 |
| **Timor-Leste** | **NoCOVIDginiB** | 5.006 | 4.798 | 4.803 | 6.627 | 8.222 |
| **Togo** | **GDPrGiniBase** | 1.753 | 4.776 | 5.911 | 5.639 | 8.686 |
| **Togo** | **GDPrHGiniH** | 1.753 | 4.776 | 7.411 | 5.778 | 8.715 |
| **Togo** | **GDPrHGiniHv** | 1.753 | 4.776 | 7.411 | 5.763 | 8.704 |
| **Togo** | **GDPrHGiniLow** | 1.753 | 4.776 | 7.411 | 5.797 | 8.73 |
| **Togo** | **GDPrLGiniH** | 1.753 | 4.776 | 4.411 | 5.482 | 8.629 |
| **Togo** | **GDPrLGiniHv** | 1.753 | 4.776 | 4.411 | 5.467 | 8.617 |
| **Togo** | **GDPrLGiniLow** | 1.753 | 4.776 | 4.411 | 5.501 | 8.643 |
| **Togo** | **NoCOVIDginiB** | 5.3 | 5.41 | 5.409 | 5.826 | 8.673 |
| **Tonga** | **GDPrGiniBase** | 0.654 | -2.022 | 2.894 | 1.333 | 1.856 |
| **Tonga** | **GDPrHGiniH** | 0.654 | -2.022 | 4.394 | 1.417 | 1.833 |
| **Tonga** | **GDPrHGiniHv** | 0.654 | -2.022 | 4.394 | 1.41 | 1.827 |
| **Tonga** | **GDPrHGiniLow** | 0.654 | -2.022 | 4.394 | 1.426 | 1.842 |
| **Tonga** | **GDPrLGiniH** | 0.654 | -2.022 | 1.394 | 1.232 | 1.863 |
| **Tonga** | **GDPrLGiniHv** | 0.654 | -2.022 | 1.394 | 1.22 | 1.856 |
| **Tonga** | **GDPrLGiniLow** | 0.654 | -2.022 | 1.394 | 1.245 | 1.877 |
| **Tonga** | **NoCOVIDginiB** | 3.685 | 2.876 | 2.436 | 1.445 | 1.836 |
| **Trinidad and Tobago** | **GDPrGiniBase** | -7.854 | -1.015 | 5.419 | 1.256 | 1.172 |
| **Trinidad and Tobago** | **GDPrHGiniH** | -7.854 | -1.015 | 6.919 | 1.293 | 1.14 |
| **Trinidad and Tobago** | **GDPrHGiniHv** | -7.854 | -1.015 | 6.919 | 1.283 | 1.14 |
| **Trinidad and Tobago** | **GDPrHGiniLow** | -7.854 | -1.015 | 6.919 | 1.306 | 1.14 |
| **Trinidad and Tobago** | **GDPrLGiniH** | -7.854 | -1.015 | 3.919 | 1.213 | 1.128 |
| **Trinidad and Tobago** | **GDPrLGiniHv** | -7.854 | -1.015 | 3.919 | 1.203 | 1.128 |
| **Trinidad and Tobago** | **GDPrLGiniLow** | -7.854 | -1.015 | 3.919 | 1.225 | 1.129 |
| **Trinidad and Tobago** | **NoCOVIDginiB** | 1.492 | 2.292 | 1.977 | 1.539 | 1.112 |
| **Tunisia** | **GDPrGiniBase** | -9.182 | 3.016 | 3.344 | 1.923 | 2.234 |
| **Tunisia** | **GDPrHGiniH** | -9.182 | 3.016 | 4.844 | 1.963 | 2.2 |
| **Tunisia** | **GDPrHGiniHv** | -9.182 | 3.016 | 4.844 | 1.956 | 2.196 |
| **Tunisia** | **GDPrHGiniLow** | -9.182 | 3.016 | 4.844 | 1.969 | 2.203 |
| **Tunisia** | **GDPrLGiniH** | -9.182 | 3.016 | 1.844 | 1.875 | 2.254 |
| **Tunisia** | **GDPrLGiniHv** | -9.182 | 3.016 | 1.844 | 1.868 | 2.25 |
| **Tunisia** | **GDPrLGiniLow** | -9.182 | 3.016 | 1.844 | 1.883 | 2.258 |
| **Tunisia** | **NoCOVIDginiB** | 2.428 | 2.889 | 3.803 | 2.237 | 2.156 |
| **Turkey** | **GDPrGiniBase** | 1.794 | 11 | 3.3 | 4.096 | 2.581 |
| **Turkey** | **GDPrHGiniH** | 1.794 | 11 | 4.8 | 4.154 | 2.555 |
| **Turkey** | **GDPrHGiniHv** | 1.794 | 11 | 4.8 | 4.152 | 2.555 |
| **Turkey** | **GDPrHGiniLow** | 1.794 | 11 | 4.8 | 4.157 | 2.555 |
| **Turkey** | **GDPrLGiniH** | 1.794 | 11 | 1.8 | 4.037 | 2.607 |
| **Turkey** | **GDPrLGiniHv** | 1.794 | 11 | 1.8 | 4.034 | 2.607 |
| **Turkey** | **GDPrLGiniLow** | 1.794 | 11 | 1.8 | 4.04 | 2.607 |
| **Turkey** | **NoCOVIDginiB** | 2.986 | 2.998 | 3.032 | 4.011 | 2.67 |
| **Turkmenistan** | **GDPrGiniBase** | -3.355 | 4.532 | 1.673 | 5.135 | 4.517 |
| **Turkmenistan** | **GDPrHGiniH** | -3.355 | 4.532 | 3.173 | 5.186 | 4.547 |
| **Turkmenistan** | **GDPrHGiniHv** | -3.355 | 4.532 | 3.173 | 5.182 | 4.547 |
| **Turkmenistan** | **GDPrHGiniLow** | -3.355 | 4.532 | 3.173 | 5.191 | 4.548 |
| **Turkmenistan** | **GDPrLGiniH** | -3.355 | 4.532 | 0.173 | 5.076 | 4.485 |
| **Turkmenistan** | **GDPrLGiniHv** | -3.355 | 4.532 | 0.173 | 5.073 | 4.484 |
| **Turkmenistan** | **GDPrLGiniLow** | -3.355 | 4.532 | 0.173 | 5.081 | 4.486 |
| **Turkmenistan** | **NoCOVIDginiB** | 6.038 | 5.841 | 5.944 | 5.635 | 4.43 |
| **Uganda** | **GDPrGiniBase** | 2.951 | 4.707 | 5.145 | 5.88 | 8.277 |
| **Uganda** | **GDPrHGiniH** | 2.951 | 4.707 | 6.645 | 6.122 | 8.215 |
| **Uganda** | **GDPrHGiniHv** | 2.951 | 4.707 | 6.645 | 6.093 | 8.183 |
| **Uganda** | **GDPrHGiniLow** | 2.951 | 4.707 | 6.645 | 6.173 | 8.262 |
| **Uganda** | **GDPrLGiniH** | 2.951 | 4.707 | 3.645 | 5.713 | 8.281 |
| **Uganda** | **GDPrLGiniHv** | 2.951 | 4.707 | 3.645 | 5.687 | 8.247 |
| **Uganda** | **GDPrLGiniLow** | 2.951 | 4.707 | 3.645 | 5.749 | 8.326 |
| **Uganda** | **NoCOVIDginiB** | 6.216 | 6.142 | 6.057 | 6.341 | 8.233 |
| **Ukraine** | **GDPrGiniBase** | -4 | 3.45 | 3.636 | 0.746 | -0.232 |
| **Ukraine** | **GDPrHGiniH** | -4 | 3.45 | 5.136 | 0.737 | -0.277 |
| **Ukraine** | **GDPrHGiniHv** | -4 | 3.45 | 5.136 | 0.734 | -0.275 |
| **Ukraine** | **GDPrHGiniLow** | -4 | 3.45 | 5.136 | 0.742 | -0.279 |
| **Ukraine** | **GDPrLGiniH** | -4 | 3.45 | 2.136 | 0.727 | -0.193 |
| **Ukraine** | **GDPrLGiniHv** | -4 | 3.45 | 2.136 | 0.724 | -0.191 |
| **Ukraine** | **GDPrLGiniLow** | -4 | 3.45 | 2.136 | 0.727 | -0.194 |
| **Ukraine** | **NoCOVIDginiB** | 3.011 | 3.143 | 3.19 | 0.914 | -0.326 |
| **United Arab Emirates** | **GDPrGiniBase** | -6.135 | 2.237 | 3.038 | 0.252 | -3.779 |
| **United Arab Emirates** | **GDPrHGiniH** | -6.135 | 2.237 | 4.538 | 0.295 | -3.74 |
| **United Arab Emirates** | **GDPrHGiniHv** | -6.135 | 2.237 | 4.538 | 0.293 | -3.741 |
| **United Arab Emirates** | **GDPrHGiniLow** | -6.135 | 2.237 | 4.538 | 0.297 | -3.74 |
| **United Arab Emirates** | **GDPrLGiniH** | -6.135 | 2.237 | 1.538 | 0.219 | -3.808 |
| **United Arab Emirates** | **GDPrLGiniHv** | -6.135 | 2.237 | 1.538 | 0.217 | -3.808 |
| **United Arab Emirates** | **GDPrLGiniLow** | -6.135 | 2.237 | 1.538 | 0.222 | -3.807 |
| **United Arab Emirates** | **NoCOVIDginiB** | 2.515 | 2.723 | 2.318 | 0.292 | -3.794 |
| **United Kingdom** | **GDPrGiniBase** | -9.694 | 7.2 | 4.7 | 0.802 | 1.072 |
| **United Kingdom** | **GDPrHGiniH** | -9.694 | 7.2 | 6.2 | 0.816 | 1.123 |
| **United Kingdom** | **GDPrHGiniHv** | -9.694 | 7.2 | 6.2 | 0.814 | 1.122 |
| **United Kingdom** | **GDPrHGiniLow** | -9.694 | 7.2 | 6.2 | 0.818 | 1.123 |
| **United Kingdom** | **GDPrLGiniH** | -9.694 | 7.2 | 3.2 | 0.784 | 1.03 |
| **United Kingdom** | **GDPrLGiniHv** | -9.694 | 7.2 | 3.2 | 0.782 | 1.029 |
| **United Kingdom** | **GDPrLGiniLow** | -9.694 | 7.2 | 3.2 | 0.786 | 1.03 |
| **United Kingdom** | **NoCOVIDginiB** | 1.448 | 1.543 | 1.502 | 0.813 | 1.07 |
| **United States of America** | **GDPrGiniBase** | -3.642 | 5.6 | 4 | 1.523 | 1.442 |
| **United States of America** | **GDPrHGiniH** | -3.642 | 5.6 | 5.5 | 1.573 | 1.445 |
| **United States of America** | **GDPrHGiniHv** | -3.642 | 5.6 | 5.5 | 1.568 | 1.445 |
| **United States of America** | **GDPrHGiniLow** | -3.642 | 5.6 | 5.5 | 1.578 | 1.446 |
| **United States of America** | **GDPrLGiniH** | -3.642 | 5.6 | 2.5 | 1.465 | 1.433 |
| **United States of America** | **GDPrLGiniHv** | -3.642 | 5.6 | 2.5 | 1.461 | 1.433 |
| **United States of America** | **GDPrLGiniLow** | -3.642 | 5.6 | 2.5 | 1.471 | 1.434 |
| **United States of America** | **NoCOVIDginiB** | 2.089 | 1.746 | 1.559 | 1.536 | 1.435 |
| **Uruguay** | **GDPrGiniBase** | -5.859 | 3.1 | 3.2 | 3.028 | 3.623 |
| **Uruguay** | **GDPrHGiniH** | -5.859 | 3.1 | 4.7 | 3.076 | 3.575 |
| **Uruguay** | **GDPrHGiniHv** | -5.859 | 3.1 | 4.7 | 3.071 | 3.578 |
| **Uruguay** | **GDPrHGiniLow** | -5.859 | 3.1 | 4.7 | 3.081 | 3.571 |
| **Uruguay** | **GDPrLGiniH** | -5.859 | 3.1 | 1.7 | 2.972 | 3.605 |
| **Uruguay** | **GDPrLGiniHv** | -5.859 | 3.1 | 1.7 | 2.968 | 3.604 |
| **Uruguay** | **GDPrLGiniLow** | -5.859 | 3.1 | 1.7 | 2.977 | 3.606 |
| **Uruguay** | **NoCOVIDginiB** | 2.3 | 3 | 2 | 3.218 | 3.383 |
| **Uzbekistan** | **GDPrGiniBase** | 1.715 | 6.143 | 5.375 | 5.113 | 4.474 |
| **Uzbekistan** | **GDPrHGiniH** | 1.715 | 6.143 | 6.875 | 5.183 | 4.492 |
| **Uzbekistan** | **GDPrHGiniHv** | 1.715 | 6.143 | 6.875 | 5.18 | 4.492 |
| **Uzbekistan** | **GDPrHGiniLow** | 1.715 | 6.143 | 6.875 | 5.188 | 4.491 |
| **Uzbekistan** | **GDPrLGiniH** | 1.715 | 6.143 | 3.875 | 5.033 | 4.45 |
| **Uzbekistan** | **GDPrLGiniHv** | 1.715 | 6.143 | 3.875 | 5.03 | 4.45 |
| **Uzbekistan** | **GDPrLGiniLow** | 1.715 | 6.143 | 3.875 | 5.037 | 4.45 |
| **Uzbekistan** | **NoCOVIDginiB** | 6 | 6 | 6 | 5.246 | 4.486 |
| **Vanuatu** | **GDPrGiniBase** | -6.814 | 1.219 | 2.952 | 3.393 | 4.135 |
| **Vanuatu** | **GDPrHGiniH** | -6.814 | 1.219 | 4.452 | 3.495 | 4.11 |
| **Vanuatu** | **GDPrHGiniHv** | -6.814 | 1.219 | 4.452 | 3.485 | 4.104 |
| **Vanuatu** | **GDPrHGiniLow** | -6.814 | 1.219 | 4.452 | 3.51 | 4.12 |
| **Vanuatu** | **GDPrLGiniH** | -6.814 | 1.219 | 1.452 | 3.27 | 4.163 |
| **Vanuatu** | **GDPrLGiniHv** | -6.814 | 1.219 | 1.452 | 3.258 | 4.156 |
| **Vanuatu** | **GDPrLGiniLow** | -6.814 | 1.219 | 1.452 | 3.284 | 4.174 |
| **Vanuatu** | **NoCOVIDginiB** | 3.106 | 2.801 | 2.793 | 3.69 | 4.099 |
| **Venezuela, Bolivarian Republic** | **GDPrGiniBase** | -30 | -5 | -3 | 0.695 | 3.326 |
| **Venezuela, Bolivarian Republic** | **GDPrHGiniH** | -30 | -5 | -1.5 | 0.685 | 3.373 |
| **Venezuela, Bolivarian Republic** | **GDPrHGiniHv** | -30 | -5 | -1.5 | 0.672 | 3.368 |
| **Venezuela, Bolivarian Republic** | **GDPrHGiniLow** | -30 | -5 | -1.5 | 0.702 | 3.38 |
| **Venezuela, Bolivarian Republic** | **GDPrLGiniH** | -30 | -5 | -4.5 | 0.596 | 3.289 |
| **Venezuela, Bolivarian Republic** | **GDPrLGiniHv** | -30 | -5 | -4.5 | 0.584 | 3.284 |
| **Venezuela, Bolivarian Republic** | **GDPrLGiniLow** | -30 | -5 | -4.5 | 0.613 | 3.297 |
| **Venezuela, Bolivarian Republic** | **NoCOVIDginiB** | -10 | -5 | 0 | 1.499 | 3.526 |
| **Viet Nam** | **GDPrGiniBase** | 2.906 | 3.78 | 6.628 | 5.113 | 3.87 |
| **Viet Nam** | **GDPrHGiniH** | 2.906 | 3.78 | 8.128 | 5.143 | 3.811 |
| **Viet Nam** | **GDPrHGiniHv** | 2.906 | 3.78 | 8.128 | 5.142 | 3.814 |
| **Viet Nam** | **GDPrHGiniLow** | 2.906 | 3.78 | 8.128 | 5.144 | 3.807 |
| **Viet Nam** | **GDPrLGiniH** | 2.906 | 3.78 | 5.128 | 5.075 | 3.874 |
| **Viet Nam** | **GDPrLGiniHv** | 2.906 | 3.78 | 5.128 | 5.073 | 3.875 |
| **Viet Nam** | **GDPrLGiniLow** | 2.906 | 3.78 | 5.128 | 5.078 | 3.872 |
| **Viet Nam** | **NoCOVIDginiB** | 6.5 | 6.5 | 6.5 | 5.175 | 3.797 |
| **Yemen** | **GDPrGiniBase** | -8.5 | -2 | 1 | 5.204 | 6.508 |
| **Yemen** | **GDPrHGiniH** | -8.5 | -2 | 2.5 | 5.348 | 6.51 |
| **Yemen** | **GDPrHGiniHv** | -8.5 | -2 | 2.5 | 5.343 | 6.502 |
| **Yemen** | **GDPrHGiniLow** | -8.5 | -2 | 2.5 | 5.357 | 6.522 |
| **Yemen** | **GDPrLGiniH** | -8.5 | -2 | -0.5 | 5.015 | 6.482 |
| **Yemen** | **GDPrLGiniHv** | -8.5 | -2 | -0.5 | 5.007 | 6.472 |
| **Yemen** | **GDPrLGiniLow** | -8.5 | -2 | -0.5 | 5.027 | 6.495 |
| **Yemen** | **NoCOVIDginiB** | 1.984 | 13.62 | 8.123 | 6.557 | 6.571 |
| **Zambia** | **GDPrGiniBase** | -2.785 | 1.005 | 1.145 | 4.184 | 3.74 |
| **Zambia** | **GDPrHGiniH** | -2.785 | 1.005 | 2.645 | 4.305 | 3.756 |
| **Zambia** | **GDPrHGiniHv** | -2.785 | 1.005 | 2.645 | 4.284 | 3.744 |
| **Zambia** | **GDPrHGiniLow** | -2.785 | 1.005 | 2.645 | 4.333 | 3.771 |
| **Zambia** | **GDPrLGiniH** | -2.785 | 1.005 | -0.355 | 4.038 | 3.716 |
| **Zambia** | **GDPrLGiniHv** | -2.785 | 1.005 | -0.355 | 4.018 | 3.701 |
| **Zambia** | **GDPrLGiniLow** | -2.785 | 1.005 | -0.355 | 4.065 | 3.736 |
| **Zambia** | **NoCOVIDginiB** | 1.695 | 1.728 | 1.6 | 4.415 | 3.882 |
| **Zimbabwe** | **GDPrGiniBase** | -6.249 | 5.133 | 3.081 | 4.844 | 6.508 |
| **Zimbabwe** | **GDPrHGiniH** | -6.249 | 5.133 | 4.581 | 4.959 | 6.526 |
| **Zimbabwe** | **GDPrHGiniHv** | -6.249 | 5.133 | 4.581 | 4.937 | 6.511 |
| **Zimbabwe** | **GDPrHGiniLow** | -6.249 | 5.133 | 4.581 | 4.987 | 6.545 |
| **Zimbabwe** | **GDPrLGiniH** | -6.249 | 5.133 | 1.581 | 4.719 | 6.426 |
| **Zimbabwe** | **GDPrLGiniHv** | -6.249 | 5.133 | 1.581 | 4.698 | 6.34 |
| **Zimbabwe** | **GDPrLGiniLow** | -6.249 | 5.133 | 1.581 | 4.747 | 6.454 |
| **Zimbabwe** | **NoCOVIDginiB** | 2.721 | 2.522 | 2.504 | 5.222 | 6.554 |

**Table 5. Model results for the number of people living on less than $1.90 per day in millions by scenario and selected years.**

| **Country** | **Scenario** | **2020** | **2021** | **2022** | **2030** | **2050** |
| --- | --- | --- | --- | --- | --- | --- |
| **Afghanistan** | **GDPrGiniBase** | 15.16 | 15.68 | 16.23 | 18.21 | 8.232 |
| **Afghanistan** | **GDPrHGiniH** | 15.4 | 15.92 | 15.88 | 17.34 | 7.504 |
| **Afghanistan** | **GDPrHGiniHv** | 15.75 | 16.27 | 16.24 | 17.83 | 8.252 |
| **Afghanistan** | **GDPrHGiniLow** | 14.92 | 15.43 | 15.37 | 16.67 | 6.533 |
| **Afghanistan** | **GDPrLGiniH** | 15.4 | 15.92 | 16.84 | 19.78 | 10.29 |
| **Afghanistan** | **GDPrLGiniHv** | 15.75 | 16.27 | 17.18 | 20.22 | 11.12 |
| **Afghanistan** | **GDPrLGiniLow** | 14.92 | 15.43 | 16.36 | 19.18 | 9.174 |
| **Afghanistan** | **NoCOVIDginiB** | 13.46 | 13.9 | 14.34 | 15.97 | 6.21 |
| **Albania** | **GDPrGiniBase** | 0.038 | 0.035 | 0.033 | 0.021 | 0.003 |
| **Albania** | **GDPrHGiniH** | 0.044 | 0.041 | 0.036 | 0.023 | 0.003 |
| **Albania** | **GDPrHGiniHv** | 0.054 | 0.051 | 0.045 | 0.03 | 0.005 |
| **Albania** | **GDPrHGiniLow** | 0.032 | 0.03 | 0.026 | 0.016 | 0.002 |
| **Albania** | **GDPrLGiniH** | 0.044 | 0.041 | 0.041 | 0.028 | 0.004 |
| **Albania** | **GDPrLGiniHv** | 0.054 | 0.051 | 0.051 | 0.035 | 0.006 |
| **Albania** | **GDPrLGiniLow** | 0.032 | 0.03 | 0.03 | 0.02 | 0.003 |
| **Albania** | **NoCOVIDginiB** | 0.026 | 0.027 | 0.027 | 0.018 | 0.002 |
| **Algeria** | **GDPrGiniBase** | 0.144 | 0.144 | 0.165 | 0.38 | 0.043 |
| **Algeria** | **GDPrHGiniH** | 0.175 | 0.176 | 0.192 | 0.407 | 0.047 |
| **Algeria** | **GDPrHGiniHv** | 0.231 | 0.232 | 0.253 | 0.516 | 0.068 |
| **Algeria** | **GDPrHGiniLow** | 0.117 | 0.117 | 0.129 | 0.288 | 0.027 |
| **Algeria** | **GDPrLGiniH** | 0.175 | 0.176 | 0.208 | 0.497 | 0.066 |
| **Algeria** | **GDPrLGiniHv** | 0.231 | 0.232 | 0.272 | 0.623 | 0.094 |
| **Algeria** | **GDPrLGiniLow** | 0.117 | 0.117 | 0.14 | 0.357 | 0.039 |
| **Algeria** | **NoCOVIDginiB** | 0.117 | 0.106 | 0.106 | 0.348 | 0.03 |
| **Angola** | **GDPrGiniBase** | 17.1 | 18.18 | 18.91 | 21.79 | 29.69 |
| **Angola** | **GDPrHGiniH** | 17.39 | 18.46 | 19.05 | 21.87 | 29.63 |
| **Angola** | **GDPrHGiniHv** | 17.82 | 18.88 | 19.49 | 22.54 | 31.1 |
| **Angola** | **GDPrHGiniLow** | 16.81 | 17.89 | 18.46 | 20.95 | 27.65 |
| **Angola** | **GDPrLGiniH** | 17.39 | 18.46 | 19.34 | 22.64 | 31.76 |
| **Angola** | **GDPrLGiniHv** | 17.82 | 18.88 | 19.77 | 23.29 | 33.19 |
| **Angola** | **GDPrLGiniLow** | 16.81 | 17.89 | 18.76 | 21.77 | 29.83 |
| **Angola** | **NoCOVIDginiB** | 16.47 | 17.1 | 17.55 | 20.02 | 25.29 |
| **Argentina** | **GDPrGiniBase** | 2.497 | 2.363 | 2.304 | 1.904 | 1.153 |
| **Argentina** | **GDPrHGiniH** | 2.768 | 2.627 | 2.518 | 1.999 | 1.255 |
| **Argentina** | **GDPrHGiniHv** | 3.199 | 3.048 | 2.932 | 2.372 | 1.544 |
| **Argentina** | **GDPrHGiniLow** | 2.24 | 2.114 | 2.016 | 1.558 | 0.927 |
| **Argentina** | **GDPrLGiniH** | 2.768 | 2.627 | 2.612 | 2.301 | 1.416 |
| **Argentina** | **GDPrLGiniHv** | 3.199 | 3.048 | 3.035 | 2.706 | 1.729 |
| **Argentina** | **GDPrLGiniLow** | 2.24 | 2.114 | 2.099 | 1.817 | 1.058 |
| **Argentina** | **NoCOVIDginiB** | 2.217 | 2.302 | 2.231 | 1.89 | 1.119 |
| **Armenia** | **GDPrGiniBase** | 0.031 | 0.028 | 0.027 | 0.014 | 0.002 |
| **Armenia** | **GDPrHGiniH** | 0.036 | 0.033 | 0.031 | 0.015 | 0.002 |
| **Armenia** | **GDPrHGiniHv** | 0.045 | 0.042 | 0.039 | 0.02 | 0.003 |
| **Armenia** | **GDPrHGiniLow** | 0.026 | 0.023 | 0.022 | 0.01 | 0.001 |
| **Armenia** | **GDPrLGiniH** | 0.036 | 0.033 | 0.034 | 0.018 | 0.003 |
| **Armenia** | **GDPrLGiniHv** | 0.045 | 0.042 | 0.043 | 0.024 | 0.004 |
| **Armenia** | **GDPrLGiniLow** | 0.026 | 0.023 | 0.024 | 0.012 | 0.002 |
| **Armenia** | **NoCOVIDginiB** | 0.018 | 0.018 | 0.018 | 0.01 | 0.001 |
| **Australia** | **GDPrGiniBase** | 0.117 | 0.11 | 0.097 | 0.071 | 0.017 |
| **Australia** | **GDPrHGiniH** | 0.143 | 0.134 | 0.112 | 0.08 | 0.02 |
| **Australia** | **GDPrHGiniHv** | 0.188 | 0.177 | 0.149 | 0.11 | 0.03 |
| **Australia** | **GDPrHGiniLow** | 0.095 | 0.089 | 0.073 | 0.05 | 0.011 |
| **Australia** | **GDPrLGiniH** | 0.143 | 0.134 | 0.126 | 0.099 | 0.025 |
| **Australia** | **GDPrLGiniHv** | 0.188 | 0.177 | 0.167 | 0.134 | 0.038 |
| **Australia** | **GDPrLGiniLow** | 0.095 | 0.089 | 0.083 | 0.063 | 0.014 |
| **Australia** | **NoCOVIDginiB** | 0.111 | 0.107 | 0.102 | 0.073 | 0.018 |
| **Austria** | **GDPrGiniBase** | 0.03 | 0.033 | 0.036 | 0.019 | 0.005 |
| **Austria** | **GDPrHGiniH** | 0.037 | 0.04 | 0.043 | 0.02 | 0.005 |
| **Austria** | **GDPrHGiniHv** | 0.049 | 0.053 | 0.056 | 0.028 | 0.008 |
| **Austria** | **GDPrHGiniLow** | 0.024 | 0.027 | 0.029 | 0.013 | 0.003 |
| **Austria** | **GDPrLGiniH** | 0.037 | 0.04 | 0.045 | 0.027 | 0.008 |
| **Austria** | **GDPrLGiniHv** | 0.049 | 0.053 | 0.059 | 0.037 | 0.011 |
| **Austria** | **GDPrLGiniLow** | 0.024 | 0.027 | 0.03 | 0.017 | 0.004 |
| **Austria** | **NoCOVIDginiB** | 0.026 | 0.026 | 0.026 | 0.017 | 0.004 |
| **Azerbaijan** | **GDPrGiniBase** | 0.011 | 0.009 | 0.007 | 0 | 0 |
| **Azerbaijan** | **GDPrHGiniH** | 0.014 | 0.012 | 0.008 | 0 | 0 |
| **Azerbaijan** | **GDPrHGiniHv** | 0.02 | 0.017 | 0.012 | 0.001 | 0 |
| **Azerbaijan** | **GDPrHGiniLow** | 0.009 | 0.007 | 0.005 | 0 | 0 |
| **Azerbaijan** | **GDPrLGiniH** | 0.014 | 0.012 | 0.009 | 0.001 | 0 |
| **Azerbaijan** | **GDPrLGiniHv** | 0.02 | 0.017 | 0.013 | 0.001 | 0 |
| **Azerbaijan** | **GDPrLGiniLow** | 0.009 | 0.007 | 0.005 | 0 | 0 |
| **Azerbaijan** | **NoCOVIDginiB** | 0.009 | 0.007 | 0.005 | 0 | 0 |
| **Bahamas** | **GDPrGiniBase** | 0.0008 | 0.0009 | 0.0009 | 0.0008 | 0.001 |
| **Bahamas** | **GDPrHGiniH** | 0.001 | 0.0012 | 0.0011 | 0.0009 | 0.0012 |
| **Bahamas** | **GDPrHGiniHv** | 0.0014 | 0.0016 | 0.0015 | 0.0013 | 0.0017 |
| **Bahamas** | **GDPrHGiniLow** | 0.0006 | 0.0007 | 0.0006 | 0.0005 | 0.0007 |
| **Bahamas** | **GDPrLGiniH** | 0.001 | 0.0012 | 0.0012 | 0.0011 | 0.0015 |
| **Bahamas** | **GDPrLGiniHv** | 0.0014 | 0.0016 | 0.0016 | 0.0015 | 0.0021 |
| **Bahamas** | **GDPrLGiniLow** | 0.0006 | 0.0007 | 0.0007 | 0.0007 | 0.0009 |
| **Bahamas** | **NoCOVIDginiB** | 0.0004 | 0.0005 | 0.0005 | 0.0007 | 0.0008 |
| **Bahrain** | **GDPrGiniBase** | 0.003 | 0.004 | 0.005 | 0.002 | 0 |
| **Bahrain** | **GDPrHGiniH** | 0.004 | 0.006 | 0.006 | 0.002 | 0 |
| **Bahrain** | **GDPrHGiniHv** | 0.006 | 0.008 | 0.009 | 0.004 | 0.001 |
| **Bahrain** | **GDPrHGiniLow** | 0.003 | 0.003 | 0.004 | 0.001 | 0 |
| **Bahrain** | **GDPrLGiniH** | 0.004 | 0.006 | 0.007 | 0.003 | 0.001 |
| **Bahrain** | **GDPrLGiniHv** | 0.006 | 0.008 | 0.009 | 0.004 | 0.001 |
| **Bahrain** | **GDPrLGiniLow** | 0.003 | 0.003 | 0.004 | 0.002 | 0 |
| **Bahrain** | **NoCOVIDginiB** | 0.003 | 0.003 | 0.004 | 0.001 | 0 |
| **Bangladesh** | **GDPrGiniBase** | 12.93 | 12.51 | 11.63 | 5.269 | 0.486 |
| **Bangladesh** | **GDPrHGiniH** | 13.97 | 13.55 | 12.1 | 5.475 | 0.522 |
| **Bangladesh** | **GDPrHGiniHv** | 15.58 | 15.14 | 13.62 | 6.484 | 0.717 |
| **Bangladesh** | **GDPrHGiniLow** | 11.92 | 11.51 | 10.18 | 4.274 | 0.327 |
| **Bangladesh** | **GDPrLGiniH** | 13.97 | 13.55 | 13.13 | 6.375 | 0.694 |
| **Bangladesh** | **GDPrLGiniHv** | 15.58 | 15.14 | 14.71 | 7.48 | 0.938 |
| **Bangladesh** | **GDPrLGiniLow** | 11.92 | 11.51 | 11.13 | 5.045 | 0.446 |
| **Bangladesh** | **NoCOVIDginiB** | 11.51 | 10.27 | 9.275 | 4.215 | 0.321 |
| **Barbados** | **GDPrGiniBase** | 0.025 | 0.023 | 0.019 | 0.013 | 0.005 |
| **Barbados** | **GDPrHGiniH** | 0.027 | 0.025 | 0.02 | 0.014 | 0.005 |
| **Barbados** | **GDPrHGiniHv** | 0.03 | 0.028 | 0.023 | 0.016 | 0.006 |
| **Barbados** | **GDPrHGiniLow** | 0.023 | 0.021 | 0.017 | 0.011 | 0.004 |
| **Barbados** | **GDPrLGiniH** | 0.027 | 0.025 | 0.022 | 0.015 | 0.006 |
| **Barbados** | **GDPrLGiniHv** | 0.03 | 0.028 | 0.025 | 0.018 | 0.007 |
| **Barbados** | **GDPrLGiniLow** | 0.023 | 0.021 | 0.018 | 0.012 | 0.004 |
| **Barbados** | **NoCOVIDginiB** | 0.015 | 0.014 | 0.013 | 0.01 | 0.004 |
| **Belarus** | **GDPrGiniBase** | 0.009 | 0.009 | 0.008 | 0.002 | 0 |
| **Belarus** | **GDPrHGiniH** | 0.011 | 0.011 | 0.01 | 0.003 | 0 |
| **Belarus** | **GDPrHGiniHv** | 0.016 | 0.015 | 0.014 | 0.004 | 0 |
| **Belarus** | **GDPrHGiniLow** | 0.007 | 0.007 | 0.006 | 0.001 | 0 |
| **Belarus** | **GDPrLGiniH** | 0.011 | 0.011 | 0.011 | 0.004 | 0 |
| **Belarus** | **GDPrLGiniHv** | 0.016 | 0.015 | 0.016 | 0.006 | 0.001 |
| **Belarus** | **GDPrLGiniLow** | 0.007 | 0.007 | 0.007 | 0.002 | 0 |
| **Belarus** | **NoCOVIDginiB** | 0.008 | 0.009 | 0.009 | 0.003 | 0 |
| **Belgium** | **GDPrGiniBase** | 0.017 | 0.019 | 0.02 | 0.006 | 0.001 |
| **Belgium** | **GDPrHGiniH** | 0.021 | 0.024 | 0.025 | 0.007 | 0.001 |
| **Belgium** | **GDPrHGiniHv** | 0.029 | 0.033 | 0.034 | 0.01 | 0.002 |
| **Belgium** | **GDPrHGiniLow** | 0.013 | 0.015 | 0.016 | 0.004 | 0 |
| **Belgium** | **GDPrLGiniH** | 0.021 | 0.024 | 0.026 | 0.009 | 0.002 |
| **Belgium** | **GDPrLGiniHv** | 0.029 | 0.033 | 0.036 | 0.014 | 0.002 |
| **Belgium** | **GDPrLGiniLow** | 0.013 | 0.015 | 0.017 | 0.005 | 0.001 |
| **Belgium** | **NoCOVIDginiB** | 0.015 | 0.017 | 0.017 | 0.006 | 0.001 |
| **Belize** | **GDPrGiniBase** | 0.07 | 0.07 | 0.072 | 0.068 | 0.054 |
| **Belize** | **GDPrHGiniH** | 0.074 | 0.075 | 0.076 | 0.071 | 0.056 |
| **Belize** | **GDPrHGiniHv** | 0.082 | 0.083 | 0.084 | 0.079 | 0.065 |
| **Belize** | **GDPrHGiniLow** | 0.065 | 0.066 | 0.066 | 0.06 | 0.046 |
| **Belize** | **GDPrLGiniH** | 0.074 | 0.075 | 0.079 | 0.076 | 0.063 |
| **Belize** | **GDPrLGiniHv** | 0.082 | 0.083 | 0.086 | 0.085 | 0.072 |
| **Belize** | **GDPrLGiniLow** | 0.065 | 0.066 | 0.069 | 0.066 | 0.052 |
| **Belize** | **NoCOVIDginiB** | 0.055 | 0.059 | 0.063 | 0.063 | 0.042 |
| **Benin** | **GDPrGiniBase** | 5.476 | 5.541 | 5.535 | 5.743 | 2.982 |
| **Benin** | **GDPrHGiniH** | 5.589 | 5.66 | 5.584 | 5.769 | 3.037 |
| **Benin** | **GDPrHGiniHv** | 5.756 | 5.836 | 5.77 | 6.022 | 3.416 |
| **Benin** | **GDPrHGiniLow** | 5.361 | 5.421 | 5.331 | 5.427 | 2.559 |
| **Benin** | **GDPrLGiniH** | 5.589 | 5.66 | 5.736 | 6.06 | 3.456 |
| **Benin** | **GDPrLGiniHv** | 5.756 | 5.836 | 5.918 | 6.308 | 3.855 |
| **Benin** | **GDPrLGiniLow** | 5.361 | 5.421 | 5.489 | 5.723 | 2.948 |
| **Benin** | **NoCOVIDginiB** | 5.291 | 5.317 | 5.357 | 5.517 | 2.712 |
| **Bhutan** | **GDPrGiniBase** | 0.015 | 0.02 | 0.026 | 0.036 | 0.011 |
| **Bhutan** | **GDPrHGiniH** | 0.017 | 0.023 | 0.028 | 0.037 | 0.012 |
| **Bhutan** | **GDPrHGiniHv** | 0.021 | 0.028 | 0.033 | 0.043 | 0.015 |
| **Bhutan** | **GDPrHGiniLow** | 0.013 | 0.018 | 0.022 | 0.029 | 0.008 |
| **Bhutan** | **GDPrLGiniH** | 0.017 | 0.023 | 0.031 | 0.043 | 0.014 |
| **Bhutan** | **GDPrLGiniHv** | 0.021 | 0.028 | 0.036 | 0.05 | 0.018 |
| **Bhutan** | **GDPrLGiniLow** | 0.013 | 0.018 | 0.024 | 0.035 | 0.01 |
| **Bhutan** | **NoCOVIDginiB** | 0.008 | 0.008 | 0.008 | 0.015 | 0.003 |
| **Bolivia** | **GDPrGiniBase** | 0.622 | 0.639 | 0.66 | 0.504 | 0.144 |
| **Bolivia** | **GDPrHGiniH** | 0.692 | 0.71 | 0.713 | 0.533 | 0.16 |
| **Bolivia** | **GDPrHGiniHv** | 0.803 | 0.823 | 0.827 | 0.634 | 0.208 |
| **Bolivia** | **GDPrHGiniLow** | 0.557 | 0.572 | 0.574 | 0.413 | 0.108 |
| **Bolivia** | **GDPrLGiniH** | 0.692 | 0.71 | 0.752 | 0.607 | 0.188 |
| **Bolivia** | **GDPrLGiniHv** | 0.803 | 0.823 | 0.87 | 0.716 | 0.243 |
| **Bolivia** | **GDPrLGiniLow** | 0.557 | 0.572 | 0.608 | 0.476 | 0.13 |
| **Bolivia** | **NoCOVIDginiB** | 0.471 | 0.472 | 0.473 | 0.38 | 0.1 |
| **Bosnia and Herzegovina** | **GDPrGiniBase** | 0.005 | 0.006 | 0.008 | 0.013 | 0.001 |
| **Bosnia and Herzegovina** | **GDPrHGiniH** | 0.007 | 0.008 | 0.009 | 0.014 | 0.002 |
| **Bosnia and Herzegovina** | **GDPrHGiniHv** | 0.009 | 0.011 | 0.013 | 0.019 | 0.002 |
| **Bosnia and Herzegovina** | **GDPrHGiniLow** | 0.004 | 0.005 | 0.006 | 0.009 | 0.001 |
| **Bosnia and Herzegovina** | **GDPrLGiniH** | 0.007 | 0.008 | 0.011 | 0.019 | 0.002 |
| **Bosnia and Herzegovina** | **GDPrLGiniHv** | 0.009 | 0.011 | 0.015 | 0.024 | 0.003 |
| **Bosnia and Herzegovina** | **GDPrLGiniLow** | 0.004 | 0.005 | 0.007 | 0.013 | 0.001 |
| **Bosnia and Herzegovina** | **NoCOVIDginiB** | 0.004 | 0.005 | 0.007 | 0.011 | 0.001 |
| **Botswana** | **GDPrGiniBase** | 0.369 | 0.366 | 0.39 | 0.351 | 0.228 |
| **Botswana** | **GDPrHGiniH** | 0.396 | 0.394 | 0.411 | 0.367 | 0.244 |
| **Botswana** | **GDPrHGiniHv** | 0.439 | 0.437 | 0.455 | 0.414 | 0.288 |
| **Botswana** | **GDPrHGiniLow** | 0.342 | 0.339 | 0.354 | 0.309 | 0.191 |
| **Botswana** | **GDPrLGiniH** | 0.396 | 0.394 | 0.428 | 0.395 | 0.268 |
| **Botswana** | **GDPrLGiniHv** | 0.439 | 0.437 | 0.472 | 0.443 | 0.314 |
| **Botswana** | **GDPrLGiniLow** | 0.342 | 0.339 | 0.37 | 0.335 | 0.212 |
| **Botswana** | **NoCOVIDginiB** | 0.304 | 0.309 | 0.322 | 0.31 | 0.201 |
| **Brazil** | **GDPrGiniBase** | 10.39 | 10.03 | 10.05 | 8.149 | 4.25 |
| **Brazil** | **GDPrHGiniH** | 11.85 | 11.46 | 11.31 | 8.992 | 4.904 |
| **Brazil** | **GDPrHGiniHv** | 14.25 | 13.82 | 13.67 | 11.1 | 6.357 |
| **Brazil** | **GDPrHGiniLow** | 9.053 | 8.716 | 8.58 | 6.609 | 3.354 |
| **Brazil** | **GDPrLGiniH** | 11.85 | 11.46 | 11.66 | 9.928 | 5.307 |
| **Brazil** | **GDPrLGiniHv** | 14.25 | 13.82 | 14.06 | 12.17 | 6.84 |
| **Brazil** | **GDPrLGiniLow** | 9.053 | 8.716 | 8.876 | 7.377 | 3.661 |
| **Brazil** | **NoCOVIDginiB** | 9.726 | 9.575 | 9.345 | 7.283 | 3.795 |
| **Brunei Darussalam** | **GDPrGiniBase** | 0.0003 | 0.0003 | 0.0002 | 0 | 0 |
| **Brunei Darussalam** | **GDPrHGiniH** | 0.0004 | 0.0004 | 0.0003 | 0 | 0 |
| **Brunei Darussalam** | **GDPrHGiniHv** | 0.0006 | 0.0006 | 0.0004 | 0.0001 | 0 |
| **Brunei Darussalam** | **GDPrHGiniLow** | 0.0002 | 0.0002 | 0.0002 | 0 | 0 |
| **Brunei Darussalam** | **GDPrLGiniH** | 0.0004 | 0.0004 | 0.0003 | 0.0001 | 0 |
| **Brunei Darussalam** | **GDPrLGiniHv** | 0.0006 | 0.0006 | 0.0005 | 0.0001 | 0 |
| **Brunei Darussalam** | **GDPrLGiniLow** | 0.0002 | 0.0002 | 0.0002 | 0 | 0 |
| **Brunei Darussalam** | **NoCOVIDginiB** | 0.0003 | 0.0002 | 0.0002 | 0 | 0 |
| **Bulgaria** | **GDPrGiniBase** | 0.083 | 0.079 | 0.073 | 0.032 | 0.012 |
| **Bulgaria** | **GDPrHGiniH** | 0.098 | 0.094 | 0.084 | 0.037 | 0.015 |
| **Bulgaria** | **GDPrHGiniHv** | 0.124 | 0.119 | 0.107 | 0.049 | 0.02 |
| **Bulgaria** | **GDPrHGiniLow** | 0.069 | 0.066 | 0.058 | 0.024 | 0.009 |
| **Bulgaria** | **GDPrLGiniH** | 0.098 | 0.094 | 0.09 | 0.043 | 0.017 |
| **Bulgaria** | **GDPrLGiniHv** | 0.124 | 0.119 | 0.114 | 0.057 | 0.024 |
| **Bulgaria** | **GDPrLGiniLow** | 0.069 | 0.066 | 0.063 | 0.029 | 0.011 |
| **Bulgaria** | **NoCOVIDginiB** | 0.071 | 0.067 | 0.065 | 0.03 | 0.012 |
| **Burkina Faso** | **GDPrGiniBase** | 7.356 | 7.259 | 7.055 | 5.439 | 1.35 |
| **Burkina Faso** | **GDPrHGiniH** | 7.525 | 7.439 | 7.047 | 5.364 | 1.371 |
| **Burkina Faso** | **GDPrHGiniHv** | 7.773 | 7.703 | 7.322 | 5.716 | 1.651 |
| **Burkina Faso** | **GDPrHGiniLow** | 7.185 | 7.077 | 6.671 | 4.894 | 1.043 |
| **Burkina Faso** | **GDPrLGiniH** | 7.525 | 7.439 | 7.44 | 6.018 | 1.721 |
| **Burkina Faso** | **GDPrLGiniHv** | 7.773 | 7.703 | 7.709 | 6.376 | 2.041 |
| **Burkina Faso** | **GDPrLGiniLow** | 7.185 | 7.077 | 7.073 | 5.539 | 1.335 |
| **Burkina Faso** | **NoCOVIDginiB** | 6.772 | 6.68 | 6.59 | 4.953 | 1.139 |
| **Burundi** | **GDPrGiniBase** | 9.538 | 9.872 | 10.11 | 11.96 | 15.1 |
| **Burundi** | **GDPrHGiniH** | 9.53 | 9.863 | 10.04 | 11.72 | 14.49 |
| **Burundi** | **GDPrHGiniHv** | 9.52 | 9.853 | 10.03 | 11.75 | 14.7 |
| **Burundi** | **GDPrHGiniLow** | 9.548 | 9.882 | 10.06 | 11.7 | 14.19 |
| **Burundi** | **GDPrLGiniH** | 9.53 | 9.863 | 10.16 | 12.21 | 16.11 |
| **Burundi** | **GDPrLGiniHv** | 9.52 | 9.853 | 10.15 | 12.21 | 16.26 |
| **Burundi** | **GDPrLGiniLow** | 9.548 | 9.882 | 10.18 | 12.2 | 15.91 |
| **Burundi** | **NoCOVIDginiB** | 9.531 | 9.929 | 10.33 | 12.51 | 16.47 |
| **Cabo Verde** | **GDPrGiniBase** | 0.021 | 0.024 | 0.025 | 0.014 | 0.006 |
| **Cabo Verde** | **GDPrHGiniH** | 0.024 | 0.027 | 0.027 | 0.014 | 0.006 |
| **Cabo Verde** | **GDPrHGiniHv** | 0.028 | 0.032 | 0.032 | 0.017 | 0.008 |
| **Cabo Verde** | **GDPrHGiniLow** | 0.019 | 0.021 | 0.021 | 0.011 | 0.004 |
| **Cabo Verde** | **GDPrLGiniH** | 0.024 | 0.027 | 0.03 | 0.018 | 0.007 |
| **Cabo Verde** | **GDPrLGiniHv** | 0.028 | 0.032 | 0.035 | 0.021 | 0.01 |
| **Cabo Verde** | **GDPrLGiniLow** | 0.019 | 0.021 | 0.024 | 0.013 | 0.005 |
| **Cabo Verde** | **NoCOVIDginiB** | 0.011 | 0.012 | 0.012 | 0.007 | 0.003 |
| **Cambodia** | **GDPrGiniBase** | 3.749 | 3.972 | 3.867 | 1.985 | 0.183 |
| **Cambodia** | **GDPrHGiniH** | 3.887 | 4.112 | 3.882 | 1.986 | 0.188 |
| **Cambodia** | **GDPrHGiniHv** | 4.092 | 4.319 | 4.092 | 2.183 | 0.239 |
| **Cambodia** | **GDPrHGiniLow** | 3.609 | 3.831 | 3.597 | 1.731 | 0.132 |
| **Cambodia** | **GDPrLGiniH** | 3.887 | 4.112 | 4.14 | 2.255 | 0.246 |
| **Cambodia** | **GDPrLGiniHv** | 4.092 | 4.319 | 4.349 | 2.461 | 0.309 |
| **Cambodia** | **GDPrLGiniLow** | 3.609 | 3.831 | 3.856 | 1.986 | 0.177 |
| **Cambodia** | **NoCOVIDginiB** | 3.017 | 2.875 | 2.764 | 1.333 | 0.074 |
| **Cameroon** | **GDPrGiniBase** | 6.317 | 6.434 | 6.402 | 6.86 | 7.261 |
| **Cameroon** | **GDPrHGiniH** | 6.616 | 6.741 | 6.586 | 7.022 | 7.244 |
| **Cameroon** | **GDPrHGiniHv** | 7.064 | 7.202 | 7.058 | 7.596 | 8.06 |
| **Cameroon** | **GDPrHGiniLow** | 6.019 | 6.127 | 5.961 | 6.27 | 6.198 |
| **Cameroon** | **GDPrLGiniH** | 6.616 | 6.741 | 6.849 | 7.467 | 8.458 |
| **Cameroon** | **GDPrLGiniHv** | 7.064 | 7.202 | 7.321 | 8.044 | 9.322 |
| **Cameroon** | **GDPrLGiniLow** | 6.019 | 6.127 | 6.222 | 6.706 | 7.343 |
| **Cameroon** | **NoCOVIDginiB** | 6.013 | 6.031 | 6.001 | 6.464 | 6.303 |
| **Canada** | **GDPrGiniBase** | 0.101 | 0.1 | 0.098 | 0.081 | 0.046 |
| **Canada** | **GDPrHGiniH** | 0.126 | 0.125 | 0.118 | 0.093 | 0.052 |
| **Canada** | **GDPrHGiniHv** | 0.171 | 0.169 | 0.16 | 0.129 | 0.075 |
| **Canada** | **GDPrHGiniLow** | 0.08 | 0.08 | 0.075 | 0.057 | 0.03 |
| **Canada** | **GDPrLGiniH** | 0.126 | 0.125 | 0.126 | 0.113 | 0.068 |
| **Canada** | **GDPrLGiniHv** | 0.171 | 0.169 | 0.171 | 0.154 | 0.097 |
| **Canada** | **GDPrLGiniLow** | 0.08 | 0.08 | 0.08 | 0.071 | 0.04 |
| **Canada** | **NoCOVIDginiB** | 0.086 | 0.085 | 0.085 | 0.081 | 0.045 |
| **Central African Republic** | **GDPrGiniBase** | 3.452 | 3.58 | 3.661 | 4.393 | 5.872 |
| **Central African Republic** | **GDPrHGiniH** | 3.472 | 3.598 | 3.657 | 4.311 | 5.763 |
| **Central African Republic** | **GDPrHGiniHv** | 3.503 | 3.627 | 3.686 | 4.348 | 5.855 |
| **Central African Republic** | **GDPrHGiniLow** | 3.432 | 3.561 | 3.619 | 4.264 | 5.638 |
| **Central African Republic** | **GDPrLGiniH** | 3.472 | 3.598 | 3.695 | 4.509 | 5.954 |
| **Central African Republic** | **GDPrLGiniHv** | 3.503 | 3.627 | 3.723 | 4.535 | 6.036 |
| **Central African Republic** | **GDPrLGiniLow** | 3.432 | 3.561 | 3.66 | 4.475 | 5.898 |
| **Central African Republic** | **NoCOVIDginiB** | 3.376 | 3.416 | 3.477 | 4.102 | 5.257 |
| **Chad** | **GDPrGiniBase** | 7.012 | 7.434 | 7.733 | 8.395 | 7.156 |
| **Chad** | **GDPrHGiniH** | 7.155 | 7.578 | 7.755 | 8.391 | 7.058 |
| **Chad** | **GDPrHGiniHv** | 7.367 | 7.791 | 7.976 | 8.719 | 7.698 |
| **Chad** | **GDPrHGiniLow** | 6.866 | 7.287 | 7.453 | 7.946 | 6.224 |
| **Chad** | **GDPrLGiniH** | 7.155 | 7.578 | 8.005 | 8.877 | 8.142 |
| **Chad** | **GDPrLGiniHv** | 7.367 | 7.791 | 8.219 | 9.194 | 8.802 |
| **Chad** | **GDPrLGiniLow** | 6.866 | 7.287 | 7.712 | 8.444 | 7.269 |
| **Chad** | **NoCOVIDginiB** | 6.542 | 6.663 | 6.778 | 7.273 | 4.768 |
| **Chile** | **GDPrGiniBase** | 0.067 | 0.059 | 0.057 | 0.042 | 0.021 |
| **Chile** | **GDPrHGiniH** | 0.085 | 0.075 | 0.069 | 0.051 | 0.026 |
| **Chile** | **GDPrHGiniHv** | 0.117 | 0.104 | 0.098 | 0.073 | 0.04 |
| **Chile** | **GDPrHGiniLow** | 0.053 | 0.046 | 0.042 | 0.03 | 0.014 |
| **Chile** | **GDPrLGiniH** | 0.085 | 0.075 | 0.076 | 0.058 | 0.031 |
| **Chile** | **GDPrLGiniHv** | 0.117 | 0.104 | 0.106 | 0.083 | 0.046 |
| **Chile** | **GDPrLGiniLow** | 0.053 | 0.046 | 0.047 | 0.035 | 0.017 |
| **Chile** | **NoCOVIDginiB** | 0.054 | 0.054 | 0.052 | 0.039 | 0.02 |
| **China** | **GDPrGiniBase** | 2.908 | 2.207 | 1.883 | 0.339 | 0.014 |
| **China** | **GDPrHGiniH** | 3.7 | 2.842 | 2.291 | 0.422 | 0.02 |
| **China** | **GDPrHGiniHv** | 5.182 | 4.049 | 3.309 | 0.68 | 0.039 |
| **China** | **GDPrHGiniLow** | 2.254 | 1.689 | 1.334 | 0.209 | 0.007 |
| **China** | **GDPrLGiniH** | 3.7 | 2.842 | 2.596 | 0.531 | 0.027 |
| **China** | **GDPrLGiniHv** | 5.182 | 4.049 | 3.721 | 0.842 | 0.052 |
| **China** | **GDPrLGiniLow** | 2.254 | 1.689 | 1.529 | 0.269 | 0.01 |
| **China** | **NoCOVIDginiB** | 2.596 | 2.06 | 1.686 | 0.286 | 0.013 |
| **Colombia** | **GDPrGiniBase** | 2.616 | 2.476 | 2.397 | 2.066 | 2.664 |
| **Colombia** | **GDPrHGiniH** | 2.956 | 2.807 | 2.673 | 2.251 | 2.899 |
| **Colombia** | **GDPrHGiniHv** | 3.511 | 3.349 | 3.202 | 2.738 | 3.489 |
| **Colombia** | **GDPrHGiniLow** | 2.3 | 2.17 | 2.053 | 1.69 | 2.211 |
| **Colombia** | **GDPrLGiniH** | 2.956 | 2.807 | 2.774 | 2.492 | 3.154 |
| **Colombia** | **GDPrLGiniHv** | 3.511 | 3.349 | 3.314 | 3.01 | 3.772 |
| **Colombia** | **GDPrLGiniLow** | 2.3 | 2.17 | 2.139 | 1.891 | 2.43 |
| **Colombia** | **NoCOVIDginiB** | 2.271 | 2.218 | 2.13 | 1.847 | 2.395 |
| **Comoros** | **GDPrGiniBase** | 0.169 | 0.175 | 0.175 | 0.181 | 0.125 |
| **Comoros** | **GDPrHGiniH** | 0.178 | 0.185 | 0.18 | 0.186 | 0.127 |
| **Comoros** | **GDPrHGiniHv** | 0.192 | 0.199 | 0.194 | 0.203 | 0.146 |
| **Comoros** | **GDPrHGiniLow** | 0.159 | 0.165 | 0.16 | 0.164 | 0.104 |
| **Comoros** | **GDPrLGiniH** | 0.178 | 0.185 | 0.189 | 0.199 | 0.148 |
| **Comoros** | **GDPrLGiniHv** | 0.192 | 0.199 | 0.204 | 0.216 | 0.168 |
| **Comoros** | **GDPrLGiniLow** | 0.159 | 0.165 | 0.169 | 0.177 | 0.123 |
| **Comoros** | **NoCOVIDginiB** | 0.156 | 0.158 | 0.16 | 0.169 | 0.104 |
| **Congo** | **GDPrGiniBase** | 2.718 | 2.847 | 2.989 | 2.784 | 1.192 |
| **Congo** | **GDPrHGiniH** | 2.766 | 2.895 | 2.999 | 2.771 | 1.229 |
| **Congo** | **GDPrHGiniHv** | 2.837 | 2.966 | 3.07 | 2.882 | 1.383 |
| **Congo** | **GDPrHGiniLow** | 2.669 | 2.799 | 2.903 | 2.62 | 1.035 |
| **Congo** | **GDPrLGiniH** | 2.766 | 2.895 | 3.07 | 2.953 | 1.352 |
| **Congo** | **GDPrLGiniHv** | 2.837 | 2.966 | 3.138 | 3.06 | 1.512 |
| **Congo** | **GDPrLGiniLow** | 2.669 | 2.799 | 2.977 | 2.808 | 1.148 |
| **Congo** | **NoCOVIDginiB** | 2.466 | 2.54 | 2.743 | 2.518 | 1.019 |
| **Congo, Dem. Republic of the** | **GDPrGiniBase** | 64.36 | 65.82 | 66.53 | 66.95 | 18.39 |
| **Congo, Dem. Republic of the** | **GDPrHGiniH** | 64.51 | 65.99 | 66.15 | 65.8 | 18.09 |
| **Congo, Dem. Republic of the** | **GDPrHGiniHv** | 64.74 | 66.24 | 66.49 | 66.9 | 20.55 |
| **Congo, Dem. Republic of the** | **GDPrHGiniLow** | 64.22 | 65.66 | 65.72 | 64.29 | 15.01 |
| **Congo, Dem. Republic of the** | **GDPrLGiniH** | 64.51 | 65.99 | 67.33 | 69.52 | 22.17 |
| **Congo, Dem. Republic of the** | **GDPrLGiniHv** | 64.74 | 66.24 | 67.62 | 70.5 | 24.86 |
| **Congo, Dem. Republic of the** | **GDPrLGiniLow** | 64.22 | 65.66 | 66.96 | 68.19 | 18.73 |
| **Congo, Dem. Republic of the** | **NoCOVIDginiB** | 63.55 | 65.46 | 66.87 | 68.65 | 20.76 |
| **Costa Rica** | **GDPrGiniBase** | 0.054 | 0.052 | 0.049 | 0.033 | 0.012 |
| **Costa Rica** | **GDPrHGiniH** | 0.066 | 0.063 | 0.058 | 0.038 | 0.015 |
| **Costa Rica** | **GDPrHGiniHv** | 0.086 | 0.083 | 0.076 | 0.052 | 0.022 |
| **Costa Rica** | **GDPrHGiniLow** | 0.044 | 0.042 | 0.038 | 0.024 | 0.009 |
| **Costa Rica** | **GDPrLGiniH** | 0.066 | 0.063 | 0.062 | 0.043 | 0.017 |
| **Costa Rica** | **GDPrLGiniHv** | 0.086 | 0.083 | 0.081 | 0.058 | 0.025 |
| **Costa Rica** | **GDPrLGiniLow** | 0.044 | 0.042 | 0.041 | 0.028 | 0.01 |
| **Costa Rica** | **NoCOVIDginiB** | 0.046 | 0.045 | 0.044 | 0.03 | 0.011 |
| **Cote D'Ivoire** | **GDPrGiniBase** | 5.913 | 5.804 | 5.604 | 4.659 | 1.675 |
| **Cote D'Ivoire** | **GDPrHGiniH** | 6.181 | 6.078 | 5.758 | 4.769 | 1.763 |
| **Cote D'Ivoire** | **GDPrHGiniHv** | 6.581 | 6.489 | 6.176 | 5.239 | 2.121 |
| **Cote D'Ivoire** | **GDPrHGiniLow** | 5.646 | 5.531 | 5.203 | 4.16 | 1.346 |
| **Cote D'Ivoire** | **GDPrLGiniH** | 6.181 | 6.078 | 6.012 | 5.183 | 2.06 |
| **Cote D'Ivoire** | **GDPrLGiniHv** | 6.581 | 6.489 | 6.432 | 5.666 | 2.455 |
| **Cote D'Ivoire** | **GDPrLGiniLow** | 5.646 | 5.531 | 5.454 | 4.556 | 1.594 |
| **Cote D'Ivoire** | **NoCOVIDginiB** | 5.452 | 5.253 | 5.094 | 4.235 | 1.374 |
| **Croatia** | **GDPrGiniBase** | 0.016 | 0.015 | 0.014 | 0.002 | 0 |
| **Croatia** | **GDPrHGiniH** | 0.02 | 0.018 | 0.017 | 0.003 | 0 |
| **Croatia** | **GDPrHGiniHv** | 0.026 | 0.024 | 0.022 | 0.004 | 0.001 |
| **Croatia** | **GDPrHGiniLow** | 0.013 | 0.012 | 0.011 | 0.002 | 0 |
| **Croatia** | **GDPrLGiniH** | 0.02 | 0.018 | 0.018 | 0.004 | 0.001 |
| **Croatia** | **GDPrLGiniHv** | 0.026 | 0.024 | 0.024 | 0.005 | 0.001 |
| **Croatia** | **GDPrLGiniLow** | 0.013 | 0.012 | 0.012 | 0.002 | 0 |
| **Croatia** | **NoCOVIDginiB** | 0.012 | 0.011 | 0.01 | 0.002 | 0 |
| **Cuba** | **GDPrGiniBase** | 0.21 | 0.199 | 0.197 | 0.113 | 0.041 |
| **Cuba** | **GDPrHGiniH** | 0.245 | 0.232 | 0.224 | 0.127 | 0.046 |
| **Cuba** | **GDPrHGiniHv** | 0.302 | 0.288 | 0.279 | 0.163 | 0.063 |
| **Cuba** | **GDPrHGiniLow** | 0.179 | 0.17 | 0.163 | 0.087 | 0.029 |
| **Cuba** | **GDPrLGiniH** | 0.245 | 0.232 | 0.236 | 0.145 | 0.056 |
| **Cuba** | **GDPrLGiniHv** | 0.302 | 0.288 | 0.292 | 0.185 | 0.076 |
| **Cuba** | **GDPrLGiniLow** | 0.179 | 0.17 | 0.172 | 0.101 | 0.036 |
| **Cuba** | **NoCOVIDginiB** | 0.172 | 0.172 | 0.17 | 0.095 | 0.028 |
| **Cyprus** | **GDPrGiniBase** | 0.0015 | 0.0016 | 0.0016 | 0.0011 | 0.0005 |
| **Cyprus** | **GDPrHGiniH** | 0.0019 | 0.002 | 0.0019 | 0.0013 | 0.0005 |
| **Cyprus** | **GDPrHGiniHv** | 0.0027 | 0.0028 | 0.0027 | 0.0018 | 0.0008 |
| **Cyprus** | **GDPrHGiniLow** | 0.0012 | 0.0012 | 0.0012 | 0.0007 | 0.0003 |
| **Cyprus** | **GDPrLGiniH** | 0.0019 | 0.002 | 0.0022 | 0.0016 | 0.0008 |
| **Cyprus** | **GDPrLGiniHv** | 0.0027 | 0.0028 | 0.0031 | 0.0023 | 0.0012 |
| **Cyprus** | **GDPrLGiniLow** | 0.0012 | 0.0012 | 0.0014 | 0.001 | 0.0004 |
| **Cyprus** | **NoCOVIDginiB** | 0.001 | 0.0011 | 0.0012 | 0.001 | 0.0004 |
| **Czech Republic** | **GDPrGiniBase** | 0.012 | 0.013 | 0.015 | 0.005 | 0 |
| **Czech Republic** | **GDPrHGiniH** | 0.016 | 0.017 | 0.018 | 0.005 | 0 |
| **Czech Republic** | **GDPrHGiniHv** | 0.022 | 0.023 | 0.025 | 0.007 | 0.001 |
| **Czech Republic** | **GDPrHGiniLow** | 0.01 | 0.01 | 0.011 | 0.003 | 0 |
| **Czech Republic** | **GDPrLGiniH** | 0.016 | 0.017 | 0.02 | 0.007 | 0.001 |
| **Czech Republic** | **GDPrLGiniHv** | 0.022 | 0.023 | 0.027 | 0.01 | 0.001 |
| **Czech Republic** | **GDPrLGiniLow** | 0.01 | 0.01 | 0.012 | 0.004 | 0 |
| **Czech Republic** | **NoCOVIDginiB** | 0.009 | 0.009 | 0.01 | 0.004 | 0 |
| **Denmark** | **GDPrGiniBase** | 0.006 | 0.006 | 0.005 | 0.002 | 0 |
| **Denmark** | **GDPrHGiniH** | 0.008 | 0.007 | 0.006 | 0.002 | 0 |
| **Denmark** | **GDPrHGiniHv** | 0.011 | 0.01 | 0.009 | 0.003 | 0 |
| **Denmark** | **GDPrHGiniLow** | 0.005 | 0.004 | 0.004 | 0.001 | 0 |
| **Denmark** | **GDPrLGiniH** | 0.008 | 0.007 | 0.007 | 0.002 | 0 |
| **Denmark** | **GDPrLGiniHv** | 0.011 | 0.01 | 0.01 | 0.004 | 0 |
| **Denmark** | **GDPrLGiniLow** | 0.005 | 0.004 | 0.004 | 0.001 | 0 |
| **Denmark** | **NoCOVIDginiB** | 0.005 | 0.005 | 0.005 | 0.002 | 0 |
| **Djibouti** | **GDPrGiniBase** | 0.16 | 0.171 | 0.175 | 0.151 | 0.044 |
| **Djibouti** | **GDPrHGiniH** | 0.17 | 0.181 | 0.179 | 0.151 | 0.047 |
| **Djibouti** | **GDPrHGiniHv** | 0.184 | 0.195 | 0.194 | 0.167 | 0.056 |
| **Djibouti** | **GDPrHGiniLow** | 0.151 | 0.161 | 0.16 | 0.131 | 0.036 |
| **Djibouti** | **GDPrLGiniH** | 0.17 | 0.181 | 0.191 | 0.17 | 0.054 |
| **Djibouti** | **GDPrLGiniHv** | 0.184 | 0.195 | 0.207 | 0.186 | 0.065 |
| **Djibouti** | **GDPrLGiniLow** | 0.151 | 0.161 | 0.172 | 0.149 | 0.042 |
| **Djibouti** | **NoCOVIDginiB** | 0.138 | 0.147 | 0.153 | 0.13 | 0.038 |
| **Dominican Republic** | **GDPrGiniBase** | 0.107 | 0.092 | 0.083 | 0.05 | 0.015 |
| **Dominican Republic** | **GDPrHGiniH** | 0.128 | 0.111 | 0.097 | 0.059 | 0.018 |
| **Dominican Republic** | **GDPrHGiniHv** | 0.166 | 0.145 | 0.128 | 0.08 | 0.027 |
| **Dominican Republic** | **GDPrHGiniLow** | 0.088 | 0.075 | 0.065 | 0.037 | 0.01 |
| **Dominican Republic** | **GDPrLGiniH** | 0.128 | 0.111 | 0.105 | 0.067 | 0.021 |
| **Dominican Republic** | **GDPrLGiniHv** | 0.166 | 0.145 | 0.138 | 0.091 | 0.032 |
| **Dominican Republic** | **GDPrLGiniLow** | 0.088 | 0.075 | 0.071 | 0.043 | 0.012 |
| **Dominican Republic** | **NoCOVIDginiB** | 0.075 | 0.072 | 0.068 | 0.043 | 0.012 |
| **Ecuador** | **GDPrGiniBase** | 0.84 | 0.883 | 0.895 | 0.595 | 0.437 |
| **Ecuador** | **GDPrHGiniH** | 0.944 | 0.991 | 0.984 | 0.643 | 0.482 |
| **Ecuador** | **GDPrHGiniHv** | 1.112 | 1.166 | 1.159 | 0.783 | 0.605 |
| **Ecuador** | **GDPrHGiniLow** | 0.743 | 0.782 | 0.775 | 0.481 | 0.345 |
| **Ecuador** | **GDPrLGiniH** | 0.944 | 0.991 | 1.026 | 0.726 | 0.544 |
| **Ecuador** | **GDPrLGiniHv** | 1.112 | 1.166 | 1.205 | 0.877 | 0.677 |
| **Ecuador** | **GDPrLGiniLow** | 0.743 | 0.782 | 0.811 | 0.55 | 0.394 |
| **Ecuador** | **NoCOVIDginiB** | 0.736 | 0.761 | 0.733 | 0.516 | 0.39 |
| **Egypt** | **GDPrGiniBase** | 1.192 | 1.248 | 1.21 | 1.099 | 0.219 |
| **Egypt** | **GDPrHGiniH** | 1.381 | 1.445 | 1.318 | 1.159 | 0.239 |
| **Egypt** | **GDPrHGiniHv** | 1.698 | 1.774 | 1.629 | 1.454 | 0.334 |
| **Egypt** | **GDPrHGiniLow** | 1.02 | 1.07 | 0.967 | 0.831 | 0.147 |
| **Egypt** | **GDPrLGiniH** | 1.381 | 1.445 | 1.468 | 1.429 | 0.319 |
| **Egypt** | **GDPrLGiniHv** | 1.698 | 1.774 | 1.803 | 1.771 | 0.438 |
| **Egypt** | **GDPrLGiniLow** | 1.02 | 1.07 | 1.086 | 1.043 | 0.201 |
| **Egypt** | **NoCOVIDginiB** | 1.091 | 1.036 | 0.987 | 0.9 | 0.162 |
| **El Salvador** | **GDPrGiniBase** | 0.17 | 0.144 | 0.137 | 0.091 | 0.023 |
| **El Salvador** | **GDPrHGiniH** | 0.194 | 0.165 | 0.15 | 0.099 | 0.027 |
| **El Salvador** | **GDPrHGiniHv** | 0.233 | 0.201 | 0.183 | 0.124 | 0.037 |
| **El Salvador** | **GDPrHGiniLow** | 0.148 | 0.125 | 0.112 | 0.07 | 0.017 |
| **El Salvador** | **GDPrLGiniH** | 0.194 | 0.165 | 0.165 | 0.115 | 0.031 |
| **El Salvador** | **GDPrLGiniHv** | 0.233 | 0.201 | 0.2 | 0.143 | 0.043 |
| **El Salvador** | **GDPrLGiniLow** | 0.148 | 0.125 | 0.124 | 0.083 | 0.02 |
| **El Salvador** | **NoCOVIDginiB** | 0.114 | 0.123 | 0.126 | 0.086 | 0.022 |
| **Equatorial Guinea** | **GDPrGiniBase** | 0.037 | 0.036 | 0.043 | 0.032 | 0.019 |
| **Equatorial Guinea** | **GDPrHGiniH** | 0.043 | 0.043 | 0.049 | 0.036 | 0.022 |
| **Equatorial Guinea** | **GDPrHGiniHv** | 0.053 | 0.053 | 0.06 | 0.046 | 0.031 |
| **Equatorial Guinea** | **GDPrHGiniLow** | 0.031 | 0.031 | 0.036 | 0.025 | 0.014 |
| **Equatorial Guinea** | **GDPrLGiniH** | 0.043 | 0.043 | 0.052 | 0.041 | 0.025 |
| **Equatorial Guinea** | **GDPrLGiniHv** | 0.053 | 0.053 | 0.064 | 0.053 | 0.034 |
| **Equatorial Guinea** | **GDPrLGiniLow** | 0.031 | 0.031 | 0.038 | 0.029 | 0.016 |
| **Equatorial Guinea** | **NoCOVIDginiB** | 0.037 | 0.045 | 0.052 | 0.041 | 0.024 |
| **Eritrea** | **GDPrGiniBase** | 1.279 | 1.403 | 1.517 | 1.296 | 0.113 |
| **Eritrea** | **GDPrHGiniH** | 1.314 | 1.437 | 1.523 | 1.292 | 0.121 |
| **Eritrea** | **GDPrHGiniHv** | 1.365 | 1.487 | 1.571 | 1.354 | 0.152 |
| **Eritrea** | **GDPrHGiniLow** | 1.244 | 1.369 | 1.458 | 1.209 | 0.086 |
| **Eritrea** | **GDPrLGiniH** | 1.314 | 1.437 | 1.575 | 1.383 | 0.146 |
| **Eritrea** | **GDPrLGiniHv** | 1.365 | 1.487 | 1.621 | 1.443 | 0.182 |
| **Eritrea** | **GDPrLGiniLow** | 1.244 | 1.369 | 1.512 | 1.301 | 0.105 |
| **Eritrea** | **NoCOVIDginiB** | 1.207 | 1.305 | 1.385 | 1.165 | 0.077 |
| **Estonia** | **GDPrGiniBase** | 0.004 | 0.004 | 0.004 | 0.002 | 0 |
| **Estonia** | **GDPrHGiniH** | 0.005 | 0.005 | 0.004 | 0.002 | 0 |
| **Estonia** | **GDPrHGiniHv** | 0.007 | 0.006 | 0.006 | 0.003 | 0 |
| **Estonia** | **GDPrHGiniLow** | 0.003 | 0.003 | 0.003 | 0.001 | 0 |
| **Estonia** | **GDPrLGiniH** | 0.005 | 0.005 | 0.005 | 0.003 | 0 |
| **Estonia** | **GDPrLGiniHv** | 0.007 | 0.006 | 0.006 | 0.004 | 0 |
| **Estonia** | **GDPrLGiniLow** | 0.003 | 0.003 | 0.003 | 0.002 | 0 |
| **Estonia** | **NoCOVIDginiB** | 0.003 | 0.004 | 0.004 | 0.002 | 0 |
| **Eswatini** | **GDPrGiniBase** | 0.331 | 0.337 | 0.342 | 0.288 | 0.176 |
| **Eswatini** | **GDPrHGiniH** | 0.347 | 0.353 | 0.352 | 0.297 | 0.185 |
| **Eswatini** | **GDPrHGiniHv** | 0.37 | 0.376 | 0.376 | 0.323 | 0.212 |
| **Eswatini** | **GDPrHGiniLow** | 0.316 | 0.322 | 0.321 | 0.263 | 0.151 |
| **Eswatini** | **GDPrLGiniH** | 0.347 | 0.353 | 0.363 | 0.313 | 0.203 |
| **Eswatini** | **GDPrLGiniHv** | 0.37 | 0.376 | 0.386 | 0.339 | 0.232 |
| **Eswatini** | **GDPrLGiniLow** | 0.316 | 0.322 | 0.331 | 0.279 | 0.168 |
| **Eswatini** | **NoCOVIDginiB** | 0.321 | 0.331 | 0.341 | 0.292 | 0.181 |
| **Ethiopia** | **GDPrGiniBase** | 24.23 | 25.48 | 23.78 | 14.78 | 1.551 |
| **Ethiopia** | **GDPrHGiniH** | 25.25 | 26.54 | 24.05 | 14.79 | 1.602 |
| **Ethiopia** | **GDPrHGiniHv** | 26.78 | 28.13 | 25.64 | 16.37 | 2.115 |
| **Ethiopia** | **GDPrHGiniLow** | 23.2 | 24.41 | 21.94 | 12.77 | 1.062 |
| **Ethiopia** | **GDPrLGiniH** | 25.25 | 26.54 | 25.66 | 16.97 | 2.236 |
| **Ethiopia** | **GDPrLGiniHv** | 26.78 | 28.13 | 27.25 | 18.64 | 2.898 |
| **Ethiopia** | **GDPrLGiniLow** | 23.2 | 24.41 | 23.53 | 14.82 | 1.515 |
| **Ethiopia** | **NoCOVIDginiB** | 22.98 | 22.21 | 21.57 | 13.44 | 1.297 |
| **Fiji** | **GDPrGiniBase** | 0.004 | 0.006 | 0.006 | 0.008 | 0.002 |
| **Fiji** | **GDPrHGiniH** | 0.005 | 0.008 | 0.007 | 0.008 | 0.002 |
| **Fiji** | **GDPrHGiniHv** | 0.007 | 0.01 | 0.009 | 0.01 | 0.003 |
| **Fiji** | **GDPrHGiniLow** | 0.003 | 0.005 | 0.005 | 0.005 | 0.001 |
| **Fiji** | **GDPrLGiniH** | 0.005 | 0.008 | 0.008 | 0.01 | 0.002 |
| **Fiji** | **GDPrLGiniHv** | 0.007 | 0.01 | 0.011 | 0.013 | 0.003 |
| **Fiji** | **GDPrLGiniLow** | 0.003 | 0.005 | 0.006 | 0.007 | 0.001 |
| **Fiji** | **NoCOVIDginiB** | 0.002 | 0.002 | 0.002 | 0.004 | 0.001 |
| **Finland** | **GDPrGiniBase** | 0.006 | 0.005 | 0.005 | 0.003 | 0.001 |
| **Finland** | **GDPrHGiniH** | 0.008 | 0.007 | 0.006 | 0.003 | 0.001 |
| **Finland** | **GDPrHGiniHv** | 0.011 | 0.01 | 0.009 | 0.005 | 0.002 |
| **Finland** | **GDPrHGiniLow** | 0.005 | 0.004 | 0.004 | 0.002 | 0.001 |
| **Finland** | **GDPrLGiniH** | 0.008 | 0.007 | 0.006 | 0.005 | 0.002 |
| **Finland** | **GDPrLGiniHv** | 0.011 | 0.01 | 0.009 | 0.007 | 0.002 |
| **Finland** | **GDPrLGiniLow** | 0.005 | 0.004 | 0.004 | 0.003 | 0.001 |
| **Finland** | **NoCOVIDginiB** | 0.006 | 0.005 | 0.004 | 0.003 | 0.001 |
| **France** | **GDPrGiniBase** | 0.08 | 0.083 | 0.082 | 0.042 | 0.016 |
| **France** | **GDPrHGiniH** | 0.102 | 0.106 | 0.103 | 0.048 | 0.019 |
| **France** | **GDPrHGiniHv** | 0.144 | 0.149 | 0.145 | 0.072 | 0.03 |
| **France** | **GDPrHGiniLow** | 0.062 | 0.064 | 0.062 | 0.027 | 0.01 |
| **France** | **GDPrLGiniH** | 0.102 | 0.106 | 0.107 | 0.064 | 0.026 |
| **France** | **GDPrLGiniHv** | 0.144 | 0.149 | 0.15 | 0.092 | 0.04 |
| **France** | **GDPrLGiniLow** | 0.062 | 0.064 | 0.065 | 0.037 | 0.014 |
| **France** | **NoCOVIDginiB** | 0.07 | 0.066 | 0.063 | 0.04 | 0.015 |
| **Gabon** | **GDPrGiniBase** | 0.078 | 0.073 | 0.063 | 0.021 | 0.022 |
| **Gabon** | **GDPrHGiniH** | 0.088 | 0.083 | 0.068 | 0.024 | 0.024 |
| **Gabon** | **GDPrHGiniHv** | 0.103 | 0.098 | 0.082 | 0.031 | 0.032 |
| **Gabon** | **GDPrHGiniLow** | 0.069 | 0.065 | 0.052 | 0.016 | 0.016 |
| **Gabon** | **GDPrLGiniH** | 0.088 | 0.083 | 0.074 | 0.027 | 0.031 |
| **Gabon** | **GDPrLGiniHv** | 0.103 | 0.098 | 0.089 | 0.035 | 0.04 |
| **Gabon** | **GDPrLGiniLow** | 0.069 | 0.065 | 0.057 | 0.019 | 0.021 |
| **Gabon** | **NoCOVIDginiB** | 0.067 | 0.06 | 0.051 | 0.017 | 0.015 |
| **Gambia** | **GDPrGiniBase** | 0.228 | 0.242 | 0.244 | 0.177 | 0.036 |
| **Gambia** | **GDPrHGiniH** | 0.246 | 0.26 | 0.251 | 0.181 | 0.037 |
| **Gambia** | **GDPrHGiniHv** | 0.273 | 0.289 | 0.279 | 0.208 | 0.049 |
| **Gambia** | **GDPrHGiniLow** | 0.211 | 0.224 | 0.215 | 0.148 | 0.025 |
| **Gambia** | **GDPrLGiniH** | 0.246 | 0.26 | 0.274 | 0.208 | 0.049 |
| **Gambia** | **GDPrLGiniHv** | 0.273 | 0.289 | 0.303 | 0.236 | 0.064 |
| **Gambia** | **GDPrLGiniLow** | 0.211 | 0.224 | 0.236 | 0.172 | 0.034 |
| **Gambia** | **NoCOVIDginiB** | 0.185 | 0.194 | 0.21 | 0.153 | 0.027 |
| **Georgia** | **GDPrGiniBase** | 0.163 | 0.148 | 0.134 | 0.063 | 0.017 |
| **Georgia** | **GDPrHGiniH** | 0.182 | 0.166 | 0.143 | 0.066 | 0.019 |
| **Georgia** | **GDPrHGiniHv** | 0.212 | 0.194 | 0.169 | 0.081 | 0.026 |
| **Georgia** | **GDPrHGiniLow** | 0.146 | 0.131 | 0.112 | 0.048 | 0.013 |
| **Georgia** | **GDPrLGiniH** | 0.182 | 0.166 | 0.157 | 0.079 | 0.022 |
| **Georgia** | **GDPrLGiniHv** | 0.212 | 0.194 | 0.185 | 0.096 | 0.029 |
| **Georgia** | **GDPrLGiniLow** | 0.146 | 0.131 | 0.124 | 0.059 | 0.015 |
| **Georgia** | **NoCOVIDginiB** | 0.109 | 0.106 | 0.101 | 0.051 | 0.014 |
| **Germany** | **GDPrGiniBase** | 0.09 | 0.091 | 0.091 | 0.052 | 0.018 |
| **Germany** | **GDPrHGiniH** | 0.116 | 0.117 | 0.114 | 0.061 | 0.02 |
| **Germany** | **GDPrHGiniHv** | 0.165 | 0.167 | 0.163 | 0.091 | 0.032 |
| **Germany** | **GDPrHGiniLow** | 0.069 | 0.07 | 0.068 | 0.035 | 0.01 |
| **Germany** | **GDPrLGiniH** | 0.116 | 0.117 | 0.121 | 0.078 | 0.029 |
| **Germany** | **GDPrLGiniHv** | 0.165 | 0.167 | 0.172 | 0.113 | 0.045 |
| **Germany** | **GDPrLGiniLow** | 0.069 | 0.07 | 0.072 | 0.045 | 0.015 |
| **Germany** | **NoCOVIDginiB** | 0.079 | 0.078 | 0.076 | 0.051 | 0.017 |
| **Ghana** | **GDPrGiniBase** | 3.647 | 3.674 | 3.516 | 2.692 | 0.619 |
| **Ghana** | **GDPrHGiniH** | 3.924 | 3.957 | 3.698 | 2.82 | 0.673 |
| **Ghana** | **GDPrHGiniHv** | 4.353 | 4.394 | 4.128 | 3.238 | 0.872 |
| **Ghana** | **GDPrHGiniLow** | 3.376 | 3.398 | 3.15 | 2.306 | 0.461 |
| **Ghana** | **GDPrLGiniH** | 3.924 | 3.957 | 3.9 | 3.107 | 0.821 |
| **Ghana** | **GDPrLGiniHv** | 4.353 | 4.394 | 4.34 | 3.544 | 1.05 |
| **Ghana** | **GDPrLGiniLow** | 3.376 | 3.398 | 3.34 | 2.564 | 0.572 |
| **Ghana** | **NoCOVIDginiB** | 3.31 | 3.341 | 3.33 | 2.554 | 0.542 |
| **Greece** | **GDPrGiniBase** | 0.091 | 0.081 | 0.079 | 0.055 | 0.015 |
| **Greece** | **GDPrHGiniH** | 0.108 | 0.097 | 0.092 | 0.059 | 0.016 |
| **Greece** | **GDPrHGiniHv** | 0.137 | 0.124 | 0.118 | 0.077 | 0.023 |
| **Greece** | **GDPrHGiniLow** | 0.076 | 0.068 | 0.064 | 0.04 | 0.01 |
| **Greece** | **GDPrLGiniH** | 0.108 | 0.097 | 0.096 | 0.074 | 0.021 |
| **Greece** | **GDPrLGiniHv** | 0.137 | 0.124 | 0.123 | 0.096 | 0.029 |
| **Greece** | **GDPrLGiniLow** | 0.076 | 0.068 | 0.067 | 0.051 | 0.013 |
| **Greece** | **NoCOVIDginiB** | 0.076 | 0.07 | 0.066 | 0.051 | 0.013 |
| **Grenada** | **GDPrGiniBase** | 0.012 | 0.013 | 0.012 | 0.008 | 0.003 |
| **Grenada** | **GDPrHGiniH** | 0.013 | 0.014 | 0.012 | 0.008 | 0.004 |
| **Grenada** | **GDPrHGiniHv** | 0.014 | 0.015 | 0.014 | 0.009 | 0.004 |
| **Grenada** | **GDPrHGiniLow** | 0.011 | 0.012 | 0.01 | 0.007 | 0.003 |
| **Grenada** | **GDPrLGiniH** | 0.013 | 0.014 | 0.013 | 0.009 | 0.004 |
| **Grenada** | **GDPrLGiniHv** | 0.014 | 0.015 | 0.014 | 0.01 | 0.005 |
| **Grenada** | **GDPrLGiniLow** | 0.011 | 0.012 | 0.011 | 0.007 | 0.003 |
| **Grenada** | **NoCOVIDginiB** | 0.008 | 0.009 | 0.009 | 0.006 | 0.003 |
| **Guatemala** | **GDPrGiniBase** | 1.376 | 1.328 | 1.294 | 1.221 | 0.767 |
| **Guatemala** | **GDPrHGiniH** | 1.518 | 1.47 | 1.397 | 1.31 | 0.841 |
| **Guatemala** | **GDPrHGiniHv** | 1.745 | 1.695 | 1.618 | 1.54 | 1.038 |
| **Guatemala** | **GDPrHGiniLow** | 1.24 | 1.194 | 1.127 | 1.033 | 0.618 |
| **Guatemala** | **GDPrLGiniH** | 1.518 | 1.47 | 1.47 | 1.423 | 0.939 |
| **Guatemala** | **GDPrLGiniHv** | 1.745 | 1.695 | 1.697 | 1.663 | 1.15 |
| **Guatemala** | **GDPrLGiniLow** | 1.24 | 1.194 | 1.192 | 1.132 | 0.697 |
| **Guatemala** | **NoCOVIDginiB** | 1.247 | 1.255 | 1.257 | 1.195 | 0.738 |
| **Guinea** | **GDPrGiniBase** | 2.802 | 2.847 | 2.812 | 3.67 | 0.93 |
| **Guinea** | **GDPrHGiniH** | 2.916 | 2.964 | 2.845 | 3.63 | 0.943 |
| **Guinea** | **GDPrHGiniHv** | 3.086 | 3.139 | 3.024 | 3.853 | 1.115 |
| **Guinea** | **GDPrHGiniLow** | 2.687 | 2.729 | 2.605 | 3.331 | 0.737 |
| **Guinea** | **GDPrLGiniH** | 2.916 | 2.964 | 3.022 | 4.018 | 1.149 |
| **Guinea** | **GDPrLGiniHv** | 3.086 | 3.139 | 3.201 | 4.241 | 1.343 |
| **Guinea** | **GDPrLGiniLow** | 2.687 | 2.729 | 2.781 | 3.717 | 0.914 |
| **Guinea** | **NoCOVIDginiB** | 2.793 | 2.832 | 2.936 | 3.703 | 1.041 |
| **Guinea Bissau** | **GDPrGiniBase** | 1.3 | 1.331 | 1.356 | 1.475 | 1.48 |
| **Guinea Bissau** | **GDPrHGiniH** | 1.309 | 1.341 | 1.353 | 1.461 | 1.449 |
| **Guinea Bissau** | **GDPrHGiniHv** | 1.324 | 1.356 | 1.369 | 1.488 | 1.516 |
| **Guinea Bissau** | **GDPrHGiniLow** | 1.29 | 1.321 | 1.332 | 1.425 | 1.358 |
| **Guinea Bissau** | **GDPrLGiniH** | 1.309 | 1.341 | 1.377 | 1.524 | 1.605 |
| **Guinea Bissau** | **GDPrLGiniHv** | 1.324 | 1.356 | 1.392 | 1.549 | 1.669 |
| **Guinea Bissau** | **GDPrLGiniLow** | 1.29 | 1.321 | 1.357 | 1.492 | 1.519 |
| **Guinea Bissau** | **NoCOVIDginiB** | 1.247 | 1.262 | 1.277 | 1.374 | 1.219 |
| **Guyana** | **GDPrGiniBase** | 0.017 | 0.012 | 0.004 | 0.001 | 0 |
| **Guyana** | **GDPrHGiniH** | 0.02 | 0.014 | 0.005 | 0.001 | 0 |
| **Guyana** | **GDPrHGiniHv** | 0.025 | 0.018 | 0.007 | 0.001 | 0 |
| **Guyana** | **GDPrHGiniLow** | 0.015 | 0.01 | 0.003 | 0 | 0 |
| **Guyana** | **GDPrLGiniH** | 0.02 | 0.014 | 0.006 | 0.001 | 0 |
| **Guyana** | **GDPrLGiniHv** | 0.025 | 0.018 | 0.007 | 0.001 | 0 |
| **Guyana** | **GDPrLGiniLow** | 0.015 | 0.01 | 0.004 | 0 | 0 |
| **Guyana** | **NoCOVIDginiB** | 0.009 | 0.008 | 0.006 | 0.001 | 0 |
| **Haiti** | **GDPrGiniBase** | 3.292 | 3.468 | 3.582 | 4.148 | 3.223 |
| **Haiti** | **GDPrHGiniH** | 3.404 | 3.581 | 3.613 | 4.092 | 3.141 |
| **Haiti** | **GDPrHGiniHv** | 3.571 | 3.748 | 3.783 | 4.278 | 3.378 |
| **Haiti** | **GDPrHGiniLow** | 3.178 | 3.354 | 3.384 | 3.838 | 2.826 |
| **Haiti** | **GDPrLGiniH** | 3.404 | 3.581 | 3.765 | 4.444 | 3.658 |
| **Haiti** | **GDPrLGiniHv** | 3.571 | 3.748 | 3.933 | 4.624 | 3.899 |
| **Haiti** | **GDPrLGiniLow** | 3.178 | 3.354 | 3.538 | 4.199 | 3.335 |
| **Haiti** | **NoCOVIDginiB** | 3.065 | 3.128 | 3.209 | 3.799 | 2.662 |
| **Honduras** | **GDPrGiniBase** | 2.17 | 2.195 | 2.174 | 2.054 | 1.411 |
| **Honduras** | **GDPrHGiniH** | 2.294 | 2.321 | 2.254 | 2.116 | 1.485 |
| **Honduras** | **GDPrHGiniHv** | 2.481 | 2.511 | 2.446 | 2.325 | 1.699 |
| **Honduras** | **GDPrHGiniLow** | 2.048 | 2.071 | 2.003 | 1.847 | 1.221 |
| **Honduras** | **GDPrLGiniH** | 2.294 | 2.321 | 2.341 | 2.262 | 1.616 |
| **Honduras** | **GDPrLGiniHv** | 2.481 | 2.511 | 2.533 | 2.473 | 1.838 |
| **Honduras** | **GDPrLGiniLow** | 2.048 | 2.071 | 2.088 | 1.988 | 1.341 |
| **Honduras** | **NoCOVIDginiB** | 1.802 | 1.834 | 1.85 | 1.795 | 1.168 |
| **Hong Kong** | **GDPrGiniBase** | 0.011 | 0.01 | 0.009 | 0.014 | 0.003 |
| **Hong Kong** | **GDPrHGiniH** | 0.015 | 0.013 | 0.012 | 0.017 | 0.004 |
| **Hong Kong** | **GDPrHGiniHv** | 0.021 | 0.019 | 0.017 | 0.024 | 0.006 |
| **Hong Kong** | **GDPrHGiniLow** | 0.009 | 0.008 | 0.007 | 0.01 | 0.002 |
| **Hong Kong** | **GDPrLGiniH** | 0.015 | 0.013 | 0.013 | 0.02 | 0.005 |
| **Hong Kong** | **GDPrLGiniHv** | 0.021 | 0.019 | 0.019 | 0.028 | 0.007 |
| **Hong Kong** | **GDPrLGiniLow** | 0.009 | 0.008 | 0.008 | 0.012 | 0.002 |
| **Hong Kong** | **NoCOVIDginiB** | 0.009 | 0.009 | 0.008 | 0.014 | 0.003 |
| **Hungary** | **GDPrGiniBase** | 0.038 | 0.038 | 0.035 | 0.009 | 0.001 |
| **Hungary** | **GDPrHGiniH** | 0.046 | 0.046 | 0.04 | 0.01 | 0.001 |
| **Hungary** | **GDPrHGiniHv** | 0.061 | 0.061 | 0.053 | 0.015 | 0.002 |
| **Hungary** | **GDPrHGiniLow** | 0.031 | 0.031 | 0.027 | 0.006 | 0.001 |
| **Hungary** | **GDPrLGiniH** | 0.046 | 0.046 | 0.044 | 0.014 | 0.002 |
| **Hungary** | **GDPrLGiniHv** | 0.061 | 0.061 | 0.058 | 0.02 | 0.003 |
| **Hungary** | **GDPrLGiniLow** | 0.031 | 0.031 | 0.03 | 0.008 | 0.001 |
| **Hungary** | **NoCOVIDginiB** | 0.032 | 0.032 | 0.032 | 0.01 | 0.001 |
| **Iceland** | **GDPrGiniBase** | 0.0006 | 0.0007 | 0.0013 | 0.0003 | 0 |
| **Iceland** | **GDPrHGiniH** | 0.0007 | 0.0009 | 0.0014 | 0.0003 | 0 |
| **Iceland** | **GDPrHGiniHv** | 0.001 | 0.0012 | 0.0018 | 0.0004 | 0 |
| **Iceland** | **GDPrHGiniLow** | 0.0005 | 0.0006 | 0.0009 | 0.0002 | 0 |
| **Iceland** | **GDPrLGiniH** | 0.0007 | 0.0009 | 0.0016 | 0.0004 | 0 |
| **Iceland** | **GDPrLGiniHv** | 0.001 | 0.0012 | 0.0021 | 0.0006 | 0 |
| **Iceland** | **GDPrLGiniLow** | 0.0005 | 0.0006 | 0.0011 | 0.0002 | 0 |
| **Iceland** | **NoCOVIDginiB** | 0.0003 | 0.0005 | 0.0008 | 0.0002 | 0 |
| **India** | **GDPrGiniBase** | 134 | 125.4 | 117.5 | 52.61 | 8.425 |
| **India** | **GDPrHGiniH** | 144.4 | 135.6 | 123.4 | 54.95 | 9.265 |
| **India** | **GDPrHGiniHv** | 160.4 | 151.3 | 138.6 | 65.09 | 12.36 |
| **India** | **GDPrHGiniLow** | 123.8 | 115.6 | 104.3 | 42.89 | 6.073 |
| **India** | **GDPrLGiniH** | 144.4 | 135.6 | 130.5 | 63.55 | 11.5 |
| **India** | **GDPrLGiniHv** | 160.4 | 151.3 | 146 | 74.61 | 15.13 |
| **India** | **GDPrLGiniLow** | 123.8 | 115.6 | 110.8 | 50.26 | 7.689 |
| **India** | **NoCOVIDginiB** | 101.1 | 88.81 | 78.01 | 36.26 | 5.455 |
| **Indonesia** | **GDPrGiniBase** | 9.936 | 9.729 | 8.851 | 5.755 | 3.168 |
| **Indonesia** | **GDPrHGiniH** | 11.17 | 10.96 | 9.635 | 6.164 | 3.475 |
| **Indonesia** | **GDPrHGiniHv** | 13.18 | 12.94 | 11.48 | 7.592 | 4.463 |
| **Indonesia** | **GDPrHGiniLow** | 8.778 | 8.584 | 7.452 | 4.542 | 2.409 |
| **Indonesia** | **GDPrLGiniH** | 11.17 | 10.96 | 10.41 | 7.203 | 4.095 |
| **Indonesia** | **GDPrLGiniHv** | 13.18 | 12.94 | 12.35 | 8.788 | 5.206 |
| **Indonesia** | **GDPrLGiniLow** | 8.778 | 8.584 | 8.108 | 5.383 | 2.882 |
| **Indonesia** | **NoCOVIDginiB** | 8.291 | 7.455 | 6.699 | 4.577 | 2.5 |
| **Iran** | **GDPrGiniBase** | 0.482 | 0.463 | 0.432 | 0.367 | 0.114 |
| **Iran** | **GDPrHGiniH** | 0.589 | 0.567 | 0.506 | 0.42 | 0.136 |
| **Iran** | **GDPrHGiniHv** | 0.78 | 0.754 | 0.678 | 0.572 | 0.201 |
| **Iran** | **GDPrHGiniLow** | 0.39 | 0.373 | 0.329 | 0.266 | 0.077 |
| **Iran** | **GDPrLGiniH** | 0.589 | 0.567 | 0.559 | 0.499 | 0.166 |
| **Iran** | **GDPrLGiniHv** | 0.78 | 0.754 | 0.744 | 0.672 | 0.241 |
| **Iran** | **GDPrLGiniLow** | 0.39 | 0.373 | 0.367 | 0.322 | 0.095 |
| **Iran** | **NoCOVIDginiB** | 0.537 | 0.564 | 0.565 | 0.46 | 0.133 |
| **Iraq** | **GDPrGiniBase** | 0.773 | 0.837 | 0.782 | 0.316 | 0.019 |
| **Iraq** | **GDPrHGiniH** | 0.88 | 0.951 | 0.858 | 0.345 | 0.022 |
| **Iraq** | **GDPrHGiniHv** | 1.056 | 1.137 | 1.034 | 0.443 | 0.034 |
| **Iraq** | **GDPrHGiniLow** | 0.675 | 0.732 | 0.654 | 0.239 | 0.012 |
| **Iraq** | **GDPrLGiniH** | 0.88 | 0.951 | 0.926 | 0.411 | 0.029 |
| **Iraq** | **GDPrLGiniHv** | 1.056 | 1.137 | 1.11 | 0.522 | 0.044 |
| **Iraq** | **GDPrLGiniLow** | 0.675 | 0.732 | 0.71 | 0.29 | 0.016 |
| **Iraq** | **NoCOVIDginiB** | 0.382 | 0.368 | 0.386 | 0.25 | 0.013 |
| **Ireland** | **GDPrGiniBase** | 0.003 | 0.002 | 0.001 | 0 | 0 |
| **Ireland** | **GDPrHGiniH** | 0.004 | 0.002 | 0.001 | 0 | 0 |
| **Ireland** | **GDPrHGiniHv** | 0.006 | 0.003 | 0.002 | 0 | 0 |
| **Ireland** | **GDPrHGiniLow** | 0.002 | 0.001 | 0.001 | 0 | 0 |
| **Ireland** | **GDPrLGiniH** | 0.004 | 0.002 | 0.002 | 0 | 0 |
| **Ireland** | **GDPrLGiniHv** | 0.006 | 0.003 | 0.002 | 0 | 0 |
| **Ireland** | **GDPrLGiniLow** | 0.002 | 0.001 | 0.001 | 0 | 0 |
| **Ireland** | **NoCOVIDginiB** | 0.003 | 0.003 | 0.002 | 0 | 0 |
| **Israel** | **GDPrGiniBase** | 0.044 | 0.051 | 0.063 | 0.084 | 0.01 |
| **Israel** | **GDPrHGiniH** | 0.054 | 0.062 | 0.074 | 0.094 | 0.012 |
| **Israel** | **GDPrHGiniHv** | 0.072 | 0.083 | 0.098 | 0.126 | 0.019 |
| **Israel** | **GDPrHGiniLow** | 0.035 | 0.041 | 0.049 | 0.061 | 0.006 |
| **Israel** | **GDPrLGiniH** | 0.054 | 0.062 | 0.079 | 0.114 | 0.015 |
| **Israel** | **GDPrLGiniHv** | 0.072 | 0.083 | 0.104 | 0.152 | 0.024 |
| **Israel** | **GDPrLGiniLow** | 0.035 | 0.041 | 0.053 | 0.075 | 0.008 |
| **Israel** | **NoCOVIDginiB** | 0.039 | 0.05 | 0.063 | 0.088 | 0.01 |
| **Italy** | **GDPrGiniBase** | 0.9 | 0.867 | 0.864 | 0.492 | 0.152 |
| **Italy** | **GDPrHGiniH** | 1.045 | 1.009 | 0.989 | 0.529 | 0.17 |
| **Italy** | **GDPrHGiniHv** | 1.29 | 1.247 | 1.224 | 0.68 | 0.233 |
| **Italy** | **GDPrHGiniLow** | 0.768 | 0.739 | 0.723 | 0.367 | 0.107 |
| **Italy** | **GDPrLGiniH** | 1.045 | 1.009 | 1.023 | 0.651 | 0.212 |
| **Italy** | **GDPrLGiniHv** | 1.29 | 1.247 | 1.264 | 0.825 | 0.286 |
| **Italy** | **GDPrLGiniLow** | 0.768 | 0.739 | 0.751 | 0.459 | 0.136 |
| **Italy** | **NoCOVIDginiB** | 0.799 | 0.782 | 0.757 | 0.5 | 0.153 |
| **Jamaica** | **GDPrGiniBase** | 0.069 | 0.067 | 0.065 | 0.06 | 0.039 |
| **Jamaica** | **GDPrHGiniH** | 0.08 | 0.078 | 0.073 | 0.067 | 0.044 |
| **Jamaica** | **GDPrHGiniHv** | 0.099 | 0.096 | 0.09 | 0.083 | 0.057 |
| **Jamaica** | **GDPrHGiniLow** | 0.059 | 0.057 | 0.053 | 0.048 | 0.031 |
| **Jamaica** | **GDPrLGiniH** | 0.08 | 0.078 | 0.078 | 0.075 | 0.049 |
| **Jamaica** | **GDPrLGiniHv** | 0.099 | 0.096 | 0.096 | 0.093 | 0.063 |
| **Jamaica** | **GDPrLGiniLow** | 0.059 | 0.057 | 0.058 | 0.054 | 0.034 |
| **Jamaica** | **NoCOVIDginiB** | 0.05 | 0.053 | 0.055 | 0.052 | 0.033 |
| **Japan** | **GDPrGiniBase** | 0.964 | 0.929 | 0.888 | 0.646 | 0.28 |
| **Japan** | **GDPrHGiniH** | 1.145 | 1.106 | 1.027 | 0.702 | 0.307 |
| **Japan** | **GDPrHGiniHv** | 1.458 | 1.411 | 1.316 | 0.918 | 0.419 |
| **Japan** | **GDPrHGiniLow** | 0.803 | 0.773 | 0.714 | 0.473 | 0.195 |
| **Japan** | **GDPrLGiniH** | 1.145 | 1.106 | 1.092 | 0.87 | 0.394 |
| **Japan** | **GDPrLGiniHv** | 1.458 | 1.411 | 1.395 | 1.123 | 0.53 |
| **Japan** | **GDPrLGiniLow** | 0.803 | 0.773 | 0.763 | 0.597 | 0.255 |
| **Japan** | **NoCOVIDginiB** | 0.861 | 0.847 | 0.829 | 0.661 | 0.283 |
| **Jordan** | **GDPrGiniBase** | 0.027 | 0.027 | 0.025 | 0.018 | 0.006 |
| **Jordan** | **GDPrHGiniH** | 0.033 | 0.033 | 0.029 | 0.02 | 0.007 |
| **Jordan** | **GDPrHGiniHv** | 0.045 | 0.045 | 0.04 | 0.028 | 0.011 |
| **Jordan** | **GDPrHGiniLow** | 0.021 | 0.021 | 0.018 | 0.012 | 0.004 |
| **Jordan** | **GDPrLGiniH** | 0.033 | 0.033 | 0.032 | 0.025 | 0.009 |
| **Jordan** | **GDPrLGiniHv** | 0.045 | 0.045 | 0.044 | 0.035 | 0.013 |
| **Jordan** | **GDPrLGiniLow** | 0.021 | 0.021 | 0.021 | 0.016 | 0.005 |
| **Jordan** | **NoCOVIDginiB** | 0.023 | 0.022 | 0.021 | 0.015 | 0.005 |
| **Kazakhstan** | **GDPrGiniBase** | 0.022 | 0.022 | 0.02 | 0.003 | 0 |
| **Kazakhstan** | **GDPrHGiniH** | 0.028 | 0.028 | 0.024 | 0.004 | 0 |
| **Kazakhstan** | **GDPrHGiniHv** | 0.039 | 0.039 | 0.033 | 0.006 | 0.001 |
| **Kazakhstan** | **GDPrHGiniLow** | 0.017 | 0.017 | 0.014 | 0.002 | 0 |
| **Kazakhstan** | **GDPrLGiniH** | 0.028 | 0.028 | 0.027 | 0.005 | 0.001 |
| **Kazakhstan** | **GDPrLGiniHv** | 0.039 | 0.039 | 0.037 | 0.008 | 0.001 |
| **Kazakhstan** | **GDPrLGiniLow** | 0.017 | 0.017 | 0.016 | 0.003 | 0 |
| **Kazakhstan** | **NoCOVIDginiB** | 0.017 | 0.015 | 0.014 | 0.002 | 0 |
| **Kenya** | **GDPrGiniBase** | 17.52 | 17.35 | 16.87 | 14.28 | 5.174 |
| **Kenya** | **GDPrHGiniH** | 18.03 | 17.88 | 17.06 | 14.34 | 5.21 |
| **Kenya** | **GDPrHGiniHv** | 18.78 | 18.66 | 17.87 | 15.33 | 6.057 |
| **Kenya** | **GDPrHGiniLow** | 17.01 | 16.82 | 15.96 | 13.03 | 4.183 |
| **Kenya** | **GDPrLGiniH** | 18.03 | 17.88 | 17.77 | 15.52 | 6.279 |
| **Kenya** | **GDPrLGiniHv** | 18.78 | 18.66 | 18.57 | 16.5 | 7.215 |
| **Kenya** | **GDPrLGiniLow** | 17.01 | 16.82 | 16.69 | 14.2 | 5.129 |
| **Kenya** | **NoCOVIDginiB** | 16.13 | 15.74 | 15.35 | 13.01 | 4.076 |
| **Korea, Dem. People's Republic** | **GDPrGiniBase** | 8.037 | 7.955 | 7.915 | 6.821 | 3.189 |
| **Korea, Dem. People's Republic** | **GDPrHGiniH** | 8.277 | 8.196 | 8.01 | 6.87 | 3.254 |
| **Korea, Dem. People's Republic** | **GDPrHGiniHv** | 8.631 | 8.554 | 8.371 | 7.253 | 3.588 |
| **Korea, Dem. People's Republic** | **GDPrHGiniLow** | 7.794 | 7.71 | 7.518 | 6.353 | 2.819 |
| **Korea, Dem. People's Republic** | **GDPrLGiniH** | 8.277 | 8.196 | 8.312 | 7.302 | 3.602 |
| **Korea, Dem. People's Republic** | **GDPrLGiniHv** | 8.631 | 8.554 | 8.669 | 7.683 | 3.951 |
| **Korea, Dem. People's Republic** | **GDPrLGiniLow** | 7.794 | 7.71 | 7.825 | 6.787 | 3.148 |
| **Korea, Dem. People's Republic** | **NoCOVIDginiB** | 8.02 | 7.956 | 7.92 | 6.755 | 3.143 |
| **Korea, Republic of** | **GDPrGiniBase** | 0.105 | 0.096 | 0.096 | 0.033 | 0.001 |
| **Korea, Republic of** | **GDPrHGiniH** | 0.131 | 0.12 | 0.112 | 0.037 | 0.001 |
| **Korea, Republic of** | **GDPrHGiniHv** | 0.179 | 0.165 | 0.155 | 0.054 | 0.002 |
| **Korea, Republic of** | **GDPrHGiniLow** | 0.083 | 0.075 | 0.07 | 0.021 | 0.001 |
| **Korea, Republic of** | **GDPrLGiniH** | 0.131 | 0.12 | 0.13 | 0.05 | 0.002 |
| **Korea, Republic of** | **GDPrLGiniHv** | 0.179 | 0.165 | 0.178 | 0.072 | 0.003 |
| **Korea, Republic of** | **GDPrLGiniLow** | 0.083 | 0.075 | 0.082 | 0.029 | 0.001 |
| **Korea, Republic of** | **NoCOVIDginiB** | 0.089 | 0.086 | 0.084 | 0.028 | 0.001 |
| **Kosovo** | **GDPrGiniBase** | 0.018 | 0.014 | 0.013 | 0.026 | 0.001 |
| **Kosovo** | **GDPrHGiniH** | 0.021 | 0.017 | 0.014 | 0.026 | 0.001 |
| **Kosovo** | **GDPrHGiniHv** | 0.026 | 0.021 | 0.018 | 0.032 | 0.002 |
| **Kosovo** | **GDPrHGiniLow** | 0.015 | 0.012 | 0.01 | 0.019 | 0.001 |
| **Kosovo** | **GDPrLGiniH** | 0.021 | 0.017 | 0.017 | 0.035 | 0.001 |
| **Kosovo** | **GDPrLGiniHv** | 0.026 | 0.021 | 0.021 | 0.043 | 0.002 |
| **Kosovo** | **GDPrLGiniLow** | 0.015 | 0.012 | 0.012 | 0.027 | 0.001 |
| **Kosovo** | **NoCOVIDginiB** | 0.011 | 0.009 | 0.009 | 0.019 | 0.001 |
| **Kuwait** | **GDPrGiniBase** | 0.006 | 0.007 | 0.007 | 0.003 | 0.004 |
| **Kuwait** | **GDPrHGiniH** | 0.007 | 0.009 | 0.008 | 0.003 | 0.004 |
| **Kuwait** | **GDPrHGiniHv** | 0.01 | 0.013 | 0.012 | 0.005 | 0.006 |
| **Kuwait** | **GDPrHGiniLow** | 0.004 | 0.006 | 0.005 | 0.002 | 0.002 |
| **Kuwait** | **GDPrLGiniH** | 0.007 | 0.009 | 0.009 | 0.004 | 0.006 |
| **Kuwait** | **GDPrLGiniHv** | 0.01 | 0.013 | 0.012 | 0.006 | 0.008 |
| **Kuwait** | **GDPrLGiniLow** | 0.004 | 0.006 | 0.005 | 0.002 | 0.003 |
| **Kuwait** | **NoCOVIDginiB** | 0.004 | 0.004 | 0.004 | 0.002 | 0.002 |
| **Kyrgyzstan** | **GDPrGiniBase** | 0.124 | 0.157 | 0.164 | 0.204 | 0.111 |
| **Kyrgyzstan** | **GDPrHGiniH** | 0.141 | 0.177 | 0.168 | 0.191 | 0.113 |
| **Kyrgyzstan** | **GDPrHGiniHv** | 0.169 | 0.21 | 0.199 | 0.226 | 0.14 |
| **Kyrgyzstan** | **GDPrHGiniLow** | 0.109 | 0.139 | 0.131 | 0.15 | 0.082 |
| **Kyrgyzstan** | **GDPrLGiniH** | 0.141 | 0.177 | 0.199 | 0.272 | 0.146 |
| **Kyrgyzstan** | **GDPrLGiniHv** | 0.169 | 0.21 | 0.234 | 0.315 | 0.178 |
| **Kyrgyzstan** | **GDPrLGiniLow** | 0.109 | 0.139 | 0.158 | 0.22 | 0.109 |
| **Kyrgyzstan** | **NoCOVIDginiB** | 0.061 | 0.067 | 0.074 | 0.094 | 0.067 |
| **Lao People's Dem. Republic** | **GDPrGiniBase** | 0.617 | 0.633 | 0.613 | 0.353 | 0.024 |
| **Lao People's Dem. Republic** | **GDPrHGiniH** | 0.668 | 0.685 | 0.642 | 0.366 | 0.027 |
| **Lao People's Dem. Republic** | **GDPrHGiniHv** | 0.746 | 0.765 | 0.72 | 0.427 | 0.037 |
| **Lao People's Dem. Republic** | **GDPrHGiniLow** | 0.568 | 0.583 | 0.543 | 0.292 | 0.017 |
| **Lao People's Dem. Republic** | **GDPrLGiniH** | 0.668 | 0.685 | 0.688 | 0.423 | 0.033 |
| **Lao People's Dem. Republic** | **GDPrLGiniHv** | 0.746 | 0.765 | 0.769 | 0.489 | 0.045 |
| **Lao People's Dem. Republic** | **GDPrLGiniLow** | 0.568 | 0.583 | 0.586 | 0.342 | 0.021 |
| **Lao People's Dem. Republic** | **NoCOVIDginiB** | 0.527 | 0.487 | 0.45 | 0.225 | 0.013 |
| **Latvia** | **GDPrGiniBase** | 0.01 | 0.01 | 0.01 | 0.004 | 0 |
| **Latvia** | **GDPrHGiniH** | 0.013 | 0.012 | 0.011 | 0.004 | 0 |
| **Latvia** | **GDPrHGiniHv** | 0.016 | 0.016 | 0.015 | 0.006 | 0.001 |
| **Latvia** | **GDPrHGiniLow** | 0.008 | 0.008 | 0.008 | 0.003 | 0 |
| **Latvia** | **GDPrLGiniH** | 0.013 | 0.012 | 0.013 | 0.006 | 0.001 |
| **Latvia** | **GDPrLGiniHv** | 0.016 | 0.016 | 0.017 | 0.008 | 0.001 |
| **Latvia** | **GDPrLGiniLow** | 0.008 | 0.008 | 0.009 | 0.004 | 0 |
| **Latvia** | **NoCOVIDginiB** | 0.008 | 0.008 | 0.009 | 0.004 | 0 |
| **Lebanon** | **GDPrGiniBase** | 0.902 | 0.961 | 0.983 | 1.415 | 0.353 |
| **Lebanon** | **GDPrHGiniH** | 0.955 | 1.016 | 1.011 | 1.419 | 0.361 |
| **Lebanon** | **GDPrHGiniHv** | 1.037 | 1.1 | 1.094 | 1.509 | 0.417 |
| **Lebanon** | **GDPrHGiniLow** | 0.848 | 0.906 | 0.901 | 1.299 | 0.29 |
| **Lebanon** | **GDPrLGiniH** | 0.955 | 1.016 | 1.068 | 1.526 | 0.429 |
| **Lebanon** | **GDPrLGiniHv** | 1.037 | 1.1 | 1.152 | 1.615 | 0.492 |
| **Lebanon** | **GDPrLGiniLow** | 0.848 | 0.906 | 0.955 | 1.404 | 0.351 |
| **Lebanon** | **NoCOVIDginiB** | 0.438 | 0.45 | 0.42 | 0.589 | 0.177 |
| **Lesotho** | **GDPrGiniBase** | 0.704 | 0.724 | 0.759 | 0.791 | 0.319 |
| **Lesotho** | **GDPrHGiniH** | 0.726 | 0.746 | 0.761 | 0.774 | 0.327 |
| **Lesotho** | **GDPrHGiniHv** | 0.759 | 0.779 | 0.794 | 0.809 | 0.367 |
| **Lesotho** | **GDPrHGiniLow** | 0.682 | 0.702 | 0.718 | 0.728 | 0.277 |
| **Lesotho** | **GDPrLGiniH** | 0.726 | 0.746 | 0.793 | 0.847 | 0.363 |
| **Lesotho** | **GDPrLGiniHv** | 0.759 | 0.779 | 0.825 | 0.879 | 0.403 |
| **Lesotho** | **GDPrLGiniLow** | 0.682 | 0.702 | 0.75 | 0.803 | 0.312 |
| **Lesotho** | **NoCOVIDginiB** | 0.595 | 0.602 | 0.597 | 0.65 | 0.245 |
| **Liberia** | **GDPrGiniBase** | 2.655 | 2.674 | 2.644 | 3.669 | 2.021 |
| **Liberia** | **GDPrHGiniH** | 2.679 | 2.701 | 2.634 | 3.521 | 1.875 |
| **Liberia** | **GDPrHGiniHv** | 2.715 | 2.74 | 2.676 | 3.554 | 2.032 |
| **Liberia** | **GDPrHGiniLow** | 2.63 | 2.647 | 2.577 | 3.478 | 1.665 |
| **Liberia** | **GDPrLGiniH** | 2.679 | 2.701 | 2.71 | 3.831 | 2.432 |
| **Liberia** | **GDPrLGiniHv** | 2.715 | 2.74 | 2.75 | 3.852 | 2.589 |
| **Liberia** | **GDPrLGiniLow** | 2.63 | 2.647 | 2.656 | 3.804 | 2.217 |
| **Liberia** | **NoCOVIDginiB** | 2.507 | 2.623 | 2.791 | 4.004 | 2.655 |
| **Libya** | **GDPrGiniBase** | 0.319 | 0.075 | 0.041 | 0.005 | 0.001 |
| **Libya** | **GDPrHGiniH** | 0.353 | 0.088 | 0.048 | 0.005 | 0.001 |
| **Libya** | **GDPrHGiniHv** | 0.407 | 0.11 | 0.062 | 0.008 | 0.002 |
| **Libya** | **GDPrHGiniLow** | 0.286 | 0.063 | 0.032 | 0.003 | 0.001 |
| **Libya** | **GDPrLGiniH** | 0.353 | 0.088 | 0.051 | 0.008 | 0.002 |
| **Libya** | **GDPrLGiniHv** | 0.407 | 0.11 | 0.066 | 0.012 | 0.003 |
| **Libya** | **GDPrLGiniLow** | 0.286 | 0.063 | 0.034 | 0.005 | 0.001 |
| **Libya** | **NoCOVIDginiB** | 0.165 | 0.18 | 0.192 | 0.08 | 0.007 |
| **Lithuania** | **GDPrGiniBase** | 0.018 | 0.016 | 0.015 | 0.007 | 0.001 |
| **Lithuania** | **GDPrHGiniH** | 0.022 | 0.02 | 0.017 | 0.008 | 0.001 |
| **Lithuania** | **GDPrHGiniHv** | 0.028 | 0.025 | 0.022 | 0.011 | 0.001 |
| **Lithuania** | **GDPrHGiniLow** | 0.015 | 0.013 | 0.011 | 0.005 | 0 |
| **Lithuania** | **GDPrLGiniH** | 0.022 | 0.02 | 0.019 | 0.01 | 0.001 |
| **Lithuania** | **GDPrLGiniHv** | 0.028 | 0.025 | 0.024 | 0.014 | 0.001 |
| **Lithuania** | **GDPrLGiniLow** | 0.015 | 0.013 | 0.013 | 0.007 | 0 |
| **Lithuania** | **NoCOVIDginiB** | 0.017 | 0.016 | 0.015 | 0.008 | 0.001 |
| **Luxembourg** | **GDPrGiniBase** | 0.0015 | 0.0014 | 0.0012 | 0.0003 | 0.0001 |
| **Luxembourg** | **GDPrHGiniH** | 0.0019 | 0.0017 | 0.0014 | 0.0004 | 0.0001 |
| **Luxembourg** | **GDPrHGiniHv** | 0.0026 | 0.0024 | 0.002 | 0.0006 | 0.0002 |
| **Luxembourg** | **GDPrHGiniLow** | 0.0012 | 0.0011 | 0.0009 | 0.0002 | 0 |
| **Luxembourg** | **GDPrLGiniH** | 0.0019 | 0.0017 | 0.0014 | 0.0005 | 0.0001 |
| **Luxembourg** | **GDPrLGiniHv** | 0.0026 | 0.0024 | 0.002 | 0.0007 | 0.0002 |
| **Luxembourg** | **GDPrLGiniLow** | 0.0012 | 0.0011 | 0.0009 | 0.0003 | 0.0001 |
| **Luxembourg** | **NoCOVIDginiB** | 0.0014 | 0.0013 | 0.0011 | 0.0004 | 0.0001 |
| **Macedonia, North** | **GDPrGiniBase** | 0.1 | 0.101 | 0.102 | 0.085 | 0.025 |
| **Macedonia, North** | **GDPrHGiniH** | 0.11 | 0.112 | 0.108 | 0.087 | 0.026 |
| **Macedonia, North** | **GDPrHGiniHv** | 0.127 | 0.128 | 0.124 | 0.101 | 0.032 |
| **Macedonia, North** | **GDPrHGiniLow** | 0.09 | 0.091 | 0.088 | 0.07 | 0.019 |
| **Macedonia, North** | **GDPrLGiniH** | 0.11 | 0.112 | 0.117 | 0.103 | 0.032 |
| **Macedonia, North** | **GDPrLGiniHv** | 0.127 | 0.128 | 0.134 | 0.119 | 0.04 |
| **Macedonia, North** | **GDPrLGiniLow** | 0.09 | 0.091 | 0.096 | 0.084 | 0.024 |
| **Macedonia, North** | **NoCOVIDginiB** | 0.075 | 0.077 | 0.081 | 0.071 | 0.02 |
| **Madagascar** | **GDPrGiniBase** | 21.59 | 22.18 | 22.42 | 24.47 | 31.01 |
| **Madagascar** | **GDPrHGiniH** | 21.59 | 22.18 | 22.27 | 24.14 | 29.92 |
| **Madagascar** | **GDPrHGiniHv** | 21.6 | 22.19 | 22.29 | 24.27 | 30.4 |
| **Madagascar** | **GDPrHGiniLow** | 21.59 | 22.18 | 22.25 | 23.99 | 29.23 |
| **Madagascar** | **GDPrLGiniH** | 21.59 | 22.18 | 22.59 | 24.93 | 32.75 |
| **Madagascar** | **GDPrLGiniHv** | 21.6 | 22.19 | 22.6 | 25.03 | 33.11 |
| **Madagascar** | **GDPrLGiniLow** | 21.59 | 22.18 | 22.59 | 24.81 | 32.2 |
| **Madagascar** | **NoCOVIDginiB** | 20.35 | 20.56 | 20.78 | 22.27 | 23.34 |
| **Malawi** | **GDPrGiniBase** | 13.09 | 13.49 | 13.71 | 13.6 | 6.767 |
| **Malawi** | **GDPrHGiniH** | 13.14 | 13.55 | 13.57 | 13.37 | 6.511 |
| **Malawi** | **GDPrHGiniHv** | 13.22 | 13.63 | 13.66 | 13.57 | 7.127 |
| **Malawi** | **GDPrHGiniLow** | 13.03 | 13.44 | 13.45 | 13.09 | 5.703 |
| **Malawi** | **GDPrLGiniH** | 13.14 | 13.55 | 13.97 | 14.07 | 7.969 |
| **Malawi** | **GDPrLGiniHv** | 13.22 | 13.63 | 14.04 | 14.25 | 8.623 |
| **Malawi** | **GDPrLGiniLow** | 13.03 | 13.44 | 13.87 | 13.83 | 7.101 |
| **Malawi** | **NoCOVIDginiB** | 12.62 | 12.73 | 12.78 | 11.77 | 4.442 |
| **Malaysia** | **GDPrGiniBase** | 0.044 | 0.045 | 0.046 | 0.027 | 0.009 |
| **Malaysia** | **GDPrHGiniH** | 0.057 | 0.059 | 0.058 | 0.033 | 0.011 |
| **Malaysia** | **GDPrHGiniHv** | 0.082 | 0.086 | 0.084 | 0.049 | 0.018 |
| **Malaysia** | **GDPrHGiniLow** | 0.033 | 0.034 | 0.034 | 0.018 | 0.005 |
| **Malaysia** | **GDPrLGiniH** | 0.057 | 0.059 | 0.062 | 0.039 | 0.014 |
| **Malaysia** | **GDPrLGiniHv** | 0.082 | 0.086 | 0.09 | 0.058 | 0.022 |
| **Malaysia** | **GDPrLGiniLow** | 0.033 | 0.034 | 0.036 | 0.021 | 0.007 |
| **Malaysia** | **NoCOVIDginiB** | 0.032 | 0.031 | 0.03 | 0.02 | 0.006 |
| **Maldives** | **GDPrGiniBase** | 0.067 | 0.05 | 0.045 | 0.026 | 0.022 |
| **Maldives** | **GDPrHGiniH** | 0.071 | 0.054 | 0.047 | 0.027 | 0.022 |
| **Maldives** | **GDPrHGiniHv** | 0.077 | 0.059 | 0.052 | 0.031 | 0.026 |
| **Maldives** | **GDPrHGiniLow** | 0.063 | 0.046 | 0.04 | 0.022 | 0.018 |
| **Maldives** | **GDPrLGiniH** | 0.071 | 0.054 | 0.05 | 0.03 | 0.027 |
| **Maldives** | **GDPrLGiniHv** | 0.077 | 0.059 | 0.056 | 0.035 | 0.031 |
| **Maldives** | **GDPrLGiniLow** | 0.063 | 0.046 | 0.043 | 0.025 | 0.022 |
| **Maldives** | **NoCOVIDginiB** | 0.018 | 0.018 | 0.019 | 0.015 | 0.011 |
| **Mali** | **GDPrGiniBase** | 9.27 | 9.742 | 9.901 | 10.34 | 6.221 |
| **Mali** | **GDPrHGiniH** | 9.39 | 9.865 | 9.793 | 10 | 5.924 |
| **Mali** | **GDPrHGiniHv** | 9.566 | 10.05 | 9.982 | 10.3 | 6.518 |
| **Mali** | **GDPrHGiniLow** | 9.147 | 9.617 | 9.532 | 9.605 | 5.158 |
| **Mali** | **GDPrLGiniH** | 9.39 | 9.865 | 10.27 | 11.08 | 7.448 |
| **Mali** | **GDPrLGiniHv** | 9.566 | 10.05 | 10.44 | 11.34 | 8.082 |
| **Mali** | **GDPrLGiniLow** | 9.147 | 9.617 | 10.02 | 10.71 | 6.6 |
| **Mali** | **NoCOVIDginiB** | 8.374 | 8.622 | 8.922 | 9.316 | 4.694 |
| **Malta** | **GDPrGiniBase** | 0.0006 | 0.0006 | 0.0006 | 0.0001 | 0 |
| **Malta** | **GDPrHGiniH** | 0.0008 | 0.0007 | 0.0007 | 0.0001 | 0 |
| **Malta** | **GDPrHGiniHv** | 0.0011 | 0.001 | 0.0009 | 0.0001 | 0 |
| **Malta** | **GDPrHGiniLow** | 0.0005 | 0.0005 | 0.0004 | 0 | 0 |
| **Malta** | **GDPrLGiniH** | 0.0008 | 0.0007 | 0.0008 | 0.0001 | 0 |
| **Malta** | **GDPrLGiniHv** | 0.0011 | 0.001 | 0.0011 | 0.0002 | 0 |
| **Malta** | **GDPrLGiniLow** | 0.0005 | 0.0005 | 0.0005 | 0.0001 | 0 |
| **Malta** | **NoCOVIDginiB** | 0.0003 | 0.0003 | 0.0003 | 0.0001 | 0 |
| **Mauritania** | **GDPrGiniBase** | 0.303 | 0.33 | 0.355 | 0.502 | 0.192 |
| **Mauritania** | **GDPrHGiniH** | 0.33 | 0.359 | 0.366 | 0.489 | 0.19 |
| **Mauritania** | **GDPrHGiniHv** | 0.372 | 0.403 | 0.411 | 0.547 | 0.232 |
| **Mauritania** | **GDPrHGiniLow** | 0.277 | 0.302 | 0.308 | 0.416 | 0.142 |
| **Mauritania** | **GDPrLGiniH** | 0.33 | 0.359 | 0.405 | 0.603 | 0.255 |
| **Mauritania** | **GDPrLGiniHv** | 0.372 | 0.403 | 0.453 | 0.667 | 0.306 |
| **Mauritania** | **GDPrLGiniLow** | 0.277 | 0.302 | 0.345 | 0.521 | 0.195 |
| **Mauritania** | **NoCOVIDginiB** | 0.23 | 0.223 | 0.205 | 0.305 | 0.091 |
| **Mauritius** | **GDPrGiniBase** | 0.005 | 0.005 | 0.005 | 0.008 | 0.004 |
| **Mauritius** | **GDPrHGiniH** | 0.006 | 0.006 | 0.005 | 0.009 | 0.004 |
| **Mauritius** | **GDPrHGiniHv** | 0.008 | 0.008 | 0.007 | 0.012 | 0.006 |
| **Mauritius** | **GDPrHGiniLow** | 0.004 | 0.004 | 0.004 | 0.006 | 0.003 |
| **Mauritius** | **GDPrLGiniH** | 0.006 | 0.006 | 0.006 | 0.011 | 0.005 |
| **Mauritius** | **GDPrLGiniHv** | 0.008 | 0.008 | 0.008 | 0.014 | 0.007 |
| **Mauritius** | **GDPrLGiniLow** | 0.004 | 0.004 | 0.004 | 0.008 | 0.003 |
| **Mauritius** | **NoCOVIDginiB** | 0.002 | 0.002 | 0.003 | 0.005 | 0.002 |
| **Mexico** | **GDPrGiniBase** | 3.027 | 2.899 | 2.839 | 2.584 | 2.442 |
| **Mexico** | **GDPrHGiniH** | 3.509 | 3.369 | 3.225 | 2.867 | 2.719 |
| **Mexico** | **GDPrHGiniHv** | 4.319 | 4.161 | 3.998 | 3.602 | 3.458 |
| **Mexico** | **GDPrHGiniLow** | 2.59 | 2.473 | 2.355 | 2.053 | 1.912 |
| **Mexico** | **GDPrLGiniH** | 3.509 | 3.369 | 3.386 | 3.205 | 3.077 |
| **Mexico** | **GDPrLGiniHv** | 4.319 | 4.161 | 4.184 | 3.997 | 3.881 |
| **Mexico** | **GDPrLGiniLow** | 2.59 | 2.473 | 2.484 | 2.319 | 2.19 |
| **Mexico** | **NoCOVIDginiB** | 2.521 | 2.526 | 2.471 | 2.309 | 2.127 |
| **Micronesia** | **GDPrGiniBase** | 0.017 | 0.018 | 0.018 | 0.014 | 0.018 |
| **Micronesia** | **GDPrHGiniH** | 0.018 | 0.019 | 0.018 | 0.015 | 0.017 |
| **Micronesia** | **GDPrHGiniHv** | 0.02 | 0.02 | 0.02 | 0.017 | 0.019 |
| **Micronesia** | **GDPrHGiniLow** | 0.016 | 0.017 | 0.016 | 0.013 | 0.015 |
| **Micronesia** | **GDPrLGiniH** | 0.018 | 0.019 | 0.019 | 0.016 | 0.021 |
| **Micronesia** | **GDPrLGiniHv** | 0.02 | 0.02 | 0.021 | 0.017 | 0.023 |
| **Micronesia** | **GDPrLGiniLow** | 0.016 | 0.017 | 0.017 | 0.014 | 0.019 |
| **Micronesia** | **NoCOVIDginiB** | 0.016 | 0.016 | 0.015 | 0.013 | 0.019 |
| **Moldova, Republic of** | **GDPrGiniBase** | 0.007 | 0.007 | 0.007 | 0.007 | 0.001 |
| **Moldova, Republic of** | **GDPrHGiniH** | 0.009 | 0.009 | 0.008 | 0.008 | 0.001 |
| **Moldova, Republic of** | **GDPrHGiniHv** | 0.012 | 0.012 | 0.011 | 0.01 | 0.001 |
| **Moldova, Republic of** | **GDPrHGiniLow** | 0.005 | 0.006 | 0.005 | 0.005 | 0 |
| **Moldova, Republic of** | **GDPrLGiniH** | 0.009 | 0.009 | 0.01 | 0.011 | 0.001 |
| **Moldova, Republic of** | **GDPrLGiniHv** | 0.012 | 0.012 | 0.013 | 0.015 | 0.001 |
| **Moldova, Republic of** | **GDPrLGiniLow** | 0.005 | 0.006 | 0.006 | 0.007 | 0 |
| **Moldova, Republic of** | **NoCOVIDginiB** | 0.003 | 0.003 | 0.004 | 0.005 | 0 |
| **Mongolia** | **GDPrGiniBase** | 0.016 | 0.02 | 0.021 | 0.002 | 0 |
| **Mongolia** | **GDPrHGiniH** | 0.019 | 0.024 | 0.024 | 0.002 | 0 |
| **Mongolia** | **GDPrHGiniHv** | 0.025 | 0.031 | 0.031 | 0.004 | 0 |
| **Mongolia** | **GDPrHGiniLow** | 0.013 | 0.017 | 0.016 | 0.001 | 0 |
| **Mongolia** | **GDPrLGiniH** | 0.019 | 0.024 | 0.028 | 0.004 | 0 |
| **Mongolia** | **GDPrLGiniHv** | 0.025 | 0.031 | 0.035 | 0.005 | 0 |
| **Mongolia** | **GDPrLGiniLow** | 0.013 | 0.017 | 0.019 | 0.002 | 0 |
| **Mongolia** | **NoCOVIDginiB** | 0.009 | 0.011 | 0.013 | 0.001 | 0 |
| **Montenegro** | **GDPrGiniBase** | 0.011 | 0.01 | 0.01 | 0.011 | 0.003 |
| **Montenegro** | **GDPrHGiniH** | 0.012 | 0.012 | 0.011 | 0.011 | 0.004 |
| **Montenegro** | **GDPrHGiniHv** | 0.015 | 0.015 | 0.014 | 0.013 | 0.005 |
| **Montenegro** | **GDPrHGiniLow** | 0.009 | 0.009 | 0.008 | 0.008 | 0.002 |
| **Montenegro** | **GDPrLGiniH** | 0.012 | 0.012 | 0.012 | 0.014 | 0.004 |
| **Montenegro** | **GDPrLGiniHv** | 0.015 | 0.015 | 0.015 | 0.018 | 0.005 |
| **Montenegro** | **GDPrLGiniLow** | 0.009 | 0.009 | 0.009 | 0.011 | 0.003 |
| **Montenegro** | **NoCOVIDginiB** | 0.005 | 0.006 | 0.006 | 0.007 | 0.002 |
| **Morocco** | **GDPrGiniBase** | 0.277 | 0.272 | 0.284 | 0.167 | 0.06 |
| **Morocco** | **GDPrHGiniH** | 0.334 | 0.328 | 0.328 | 0.186 | 0.071 |
| **Morocco** | **GDPrHGiniHv** | 0.433 | 0.427 | 0.426 | 0.252 | 0.103 |
| **Morocco** | **GDPrHGiniLow** | 0.228 | 0.223 | 0.223 | 0.119 | 0.041 |
| **Morocco** | **GDPrLGiniH** | 0.334 | 0.328 | 0.355 | 0.229 | 0.086 |
| **Morocco** | **GDPrLGiniHv** | 0.433 | 0.427 | 0.459 | 0.306 | 0.124 |
| **Morocco** | **GDPrLGiniLow** | 0.228 | 0.223 | 0.243 | 0.15 | 0.051 |
| **Morocco** | **NoCOVIDginiB** | 0.203 | 0.195 | 0.183 | 0.113 | 0.042 |
| **Mozambique** | **GDPrGiniBase** | 19.97 | 20.94 | 21.3 | 23.42 | 16.61 |
| **Mozambique** | **GDPrHGiniH** | 20.16 | 21.13 | 21.25 | 23.06 | 16.75 |
| **Mozambique** | **GDPrHGiniHv** | 20.45 | 21.41 | 21.55 | 23.56 | 18.15 |
| **Mozambique** | **GDPrHGiniLow** | 19.78 | 20.75 | 20.84 | 22.39 | 14.91 |
| **Mozambique** | **GDPrLGiniH** | 20.16 | 21.13 | 21.72 | 24.49 | 18.43 |
| **Mozambique** | **GDPrLGiniHv** | 20.45 | 21.41 | 22 | 24.94 | 19.83 |
| **Mozambique** | **GDPrLGiniLow** | 19.78 | 20.75 | 21.35 | 23.88 | 16.56 |
| **Mozambique** | **NoCOVIDginiB** | 19.03 | 19.6 | 20.19 | 22.02 | 15.81 |
| **Myanmar** | **GDPrGiniBase** | 0.372 | 0.823 | 1.094 | 0.376 | 0.045 |
| **Myanmar** | **GDPrHGiniH** | 0.442 | 0.947 | 1.173 | 0.386 | 0.05 |
| **Myanmar** | **GDPrHGiniHv** | 0.563 | 1.155 | 1.412 | 0.498 | 0.074 |
| **Myanmar** | **GDPrHGiniLow** | 0.31 | 0.709 | 0.893 | 0.265 | 0.028 |
| **Myanmar** | **GDPrLGiniH** | 0.442 | 0.947 | 1.317 | 0.518 | 0.068 |
| **Myanmar** | **GDPrLGiniHv** | 0.563 | 1.155 | 1.575 | 0.658 | 0.098 |
| **Myanmar** | **GDPrLGiniLow** | 0.31 | 0.709 | 1.013 | 0.366 | 0.039 |
| **Myanmar** | **NoCOVIDginiB** | 0.316 | 0.226 | 0.176 | 0.043 | 0.004 |
| **Namibia** | **GDPrGiniBase** | 0.462 | 0.499 | 0.524 | 0.622 | 0.264 |
| **Namibia** | **GDPrHGiniH** | 0.497 | 0.536 | 0.553 | 0.639 | 0.29 |
| **Namibia** | **GDPrHGiniHv** | 0.553 | 0.594 | 0.612 | 0.707 | 0.349 |
| **Namibia** | **GDPrHGiniLow** | 0.427 | 0.463 | 0.478 | 0.552 | 0.222 |
| **Namibia** | **GDPrLGiniH** | 0.497 | 0.536 | 0.571 | 0.695 | 0.311 |
| **Namibia** | **GDPrLGiniHv** | 0.553 | 0.594 | 0.629 | 0.763 | 0.372 |
| **Namibia** | **GDPrLGiniLow** | 0.427 | 0.463 | 0.495 | 0.606 | 0.24 |
| **Namibia** | **NoCOVIDginiB** | 0.408 | 0.434 | 0.454 | 0.539 | 0.217 |
| **Nepal** | **GDPrGiniBase** | 1.47 | 1.652 | 1.749 | 1.392 | 0.749 |
| **Nepal** | **GDPrHGiniH** | 1.617 | 1.811 | 1.795 | 1.385 | 0.737 |
| **Nepal** | **GDPrHGiniHv** | 1.848 | 2.06 | 2.043 | 1.611 | 0.904 |
| **Nepal** | **GDPrHGiniLow** | 1.33 | 1.5 | 1.485 | 1.112 | 0.546 |
| **Nepal** | **GDPrLGiniH** | 1.617 | 1.811 | 1.999 | 1.696 | 1 |
| **Nepal** | **GDPrLGiniHv** | 1.848 | 2.06 | 2.261 | 1.949 | 1.204 |
| **Nepal** | **GDPrLGiniLow** | 1.33 | 1.5 | 1.669 | 1.386 | 0.762 |
| **Nepal** | **NoCOVIDginiB** | 1.039 | 0.992 | 1.009 | 0.904 | 0.39 |
| **Netherlands** | **GDPrGiniBase** | 0.032 | 0.028 | 0.022 | 0.009 | 0.001 |
| **Netherlands** | **GDPrHGiniH** | 0.04 | 0.035 | 0.027 | 0.01 | 0.001 |
| **Netherlands** | **GDPrHGiniHv** | 0.055 | 0.048 | 0.038 | 0.015 | 0.002 |
| **Netherlands** | **GDPrHGiniLow** | 0.025 | 0.022 | 0.017 | 0.006 | 0.001 |
| **Netherlands** | **GDPrLGiniH** | 0.04 | 0.035 | 0.029 | 0.014 | 0.002 |
| **Netherlands** | **GDPrLGiniHv** | 0.055 | 0.048 | 0.041 | 0.02 | 0.003 |
| **Netherlands** | **GDPrLGiniLow** | 0.025 | 0.022 | 0.018 | 0.008 | 0.001 |
| **Netherlands** | **NoCOVIDginiB** | 0.027 | 0.026 | 0.022 | 0.01 | 0.001 |
| **New Zealand** | **GDPrGiniBase** | 0.004 | 0.003 | 0.003 | 0.002 | 0 |
| **New Zealand** | **GDPrHGiniH** | 0.005 | 0.004 | 0.004 | 0.002 | 0 |
| **New Zealand** | **GDPrHGiniHv** | 0.007 | 0.006 | 0.005 | 0.003 | 0.001 |
| **New Zealand** | **GDPrHGiniLow** | 0.003 | 0.002 | 0.002 | 0.001 | 0 |
| **New Zealand** | **GDPrLGiniH** | 0.005 | 0.004 | 0.004 | 0.002 | 0.001 |
| **New Zealand** | **GDPrLGiniHv** | 0.007 | 0.006 | 0.006 | 0.004 | 0.001 |
| **New Zealand** | **GDPrLGiniLow** | 0.003 | 0.002 | 0.002 | 0.001 | 0 |
| **New Zealand** | **NoCOVIDginiB** | 0.004 | 0.004 | 0.003 | 0.002 | 0 |
| **Nicaragua** | **GDPrGiniBase** | 0.207 | 0.198 | 0.182 | 0.173 | 0.116 |
| **Nicaragua** | **GDPrHGiniH** | 0.237 | 0.228 | 0.202 | 0.183 | 0.127 |
| **Nicaragua** | **GDPrHGiniHv** | 0.287 | 0.277 | 0.248 | 0.228 | 0.163 |
| **Nicaragua** | **GDPrHGiniLow** | 0.179 | 0.171 | 0.15 | 0.134 | 0.087 |
| **Nicaragua** | **GDPrLGiniH** | 0.237 | 0.228 | 0.219 | 0.222 | 0.152 |
| **Nicaragua** | **GDPrLGiniHv** | 0.287 | 0.277 | 0.267 | 0.272 | 0.193 |
| **Nicaragua** | **GDPrLGiniLow** | 0.179 | 0.171 | 0.163 | 0.164 | 0.106 |
| **Nicaragua** | **NoCOVIDginiB** | 0.198 | 0.212 | 0.219 | 0.227 | 0.156 |
| **Niger** | **GDPrGiniBase** | 9.933 | 10.32 | 10.2 | 9.591 | 2.542 |
| **Niger** | **GDPrHGiniH** | 10.1 | 10.5 | 10.1 | 9.252 | 2.466 |
| **Niger** | **GDPrHGiniHv** | 10.35 | 10.76 | 10.39 | 9.685 | 2.916 |
| **Niger** | **GDPrHGiniLow** | 9.761 | 10.14 | 9.705 | 8.666 | 1.907 |
| **Niger** | **GDPrLGiniH** | 10.1 | 10.5 | 10.69 | 10.58 | 3.303 |
| **Niger** | **GDPrLGiniHv** | 10.35 | 10.76 | 10.96 | 11 | 3.84 |
| **Niger** | **GDPrLGiniLow** | 9.761 | 10.14 | 10.32 | 10.01 | 2.629 |
| **Niger** | **NoCOVIDginiB** | 9.238 | 9.564 | 9.017 | 8.664 | 2.059 |
| **Nigeria** | **GDPrGiniBase** | 92.04 | 94.44 | 96.83 | 105.8 | 114.7 |
| **Nigeria** | **GDPrHGiniH** | 93.77 | 96.22 | 97.37 | 105.9 | 114.1 |
| **Nigeria** | **GDPrHGiniHv** | 96.31 | 98.85 | 100.1 | 109.8 | 121.5 |
| **Nigeria** | **GDPrHGiniLow** | 90.28 | 92.62 | 93.61 | 100.7 | 104.2 |
| **Nigeria** | **GDPrLGiniH** | 93.77 | 96.22 | 100 | 110.8 | 125.1 |
| **Nigeria** | **GDPrLGiniHv** | 96.31 | 98.85 | 102.7 | 114.5 | 132.5 |
| **Nigeria** | **GDPrLGiniLow** | 90.28 | 92.62 | 96.34 | 105.6 | 115.2 |
| **Nigeria** | **NoCOVIDginiB** | 88.14 | 91.29 | 93.96 | 103 | 107.2 |
| **Norway** | **GDPrGiniBase** | 0.011 | 0.011 | 0.011 | 0.004 | 0.001 |
| **Norway** | **GDPrHGiniH** | 0.014 | 0.014 | 0.014 | 0.005 | 0.001 |
| **Norway** | **GDPrHGiniHv** | 0.018 | 0.019 | 0.019 | 0.007 | 0.002 |
| **Norway** | **GDPrHGiniLow** | 0.009 | 0.009 | 0.009 | 0.003 | 0.001 |
| **Norway** | **GDPrLGiniH** | 0.014 | 0.014 | 0.015 | 0.007 | 0.001 |
| **Norway** | **GDPrLGiniHv** | 0.018 | 0.019 | 0.02 | 0.009 | 0.002 |
| **Norway** | **GDPrLGiniLow** | 0.009 | 0.009 | 0.009 | 0.004 | 0.001 |
| **Norway** | **NoCOVIDginiB** | 0.01 | 0.01 | 0.011 | 0.005 | 0.001 |
| **Oman** | **GDPrGiniBase** | 0.007 | 0.008 | 0.007 | 0.005 | 0.006 |
| **Oman** | **GDPrHGiniH** | 0.009 | 0.01 | 0.009 | 0.006 | 0.007 |
| **Oman** | **GDPrHGiniHv** | 0.013 | 0.014 | 0.013 | 0.008 | 0.01 |
| **Oman** | **GDPrHGiniLow** | 0.005 | 0.006 | 0.005 | 0.003 | 0.004 |
| **Oman** | **GDPrLGiniH** | 0.009 | 0.01 | 0.01 | 0.006 | 0.008 |
| **Oman** | **GDPrLGiniHv** | 0.013 | 0.014 | 0.014 | 0.009 | 0.012 |
| **Oman** | **GDPrLGiniLow** | 0.005 | 0.006 | 0.006 | 0.004 | 0.005 |
| **Oman** | **NoCOVIDginiB** | 0.005 | 0.005 | 0.006 | 0.004 | 0.006 |
| **Pakistan** | **GDPrGiniBase** | 5.313 | 5.367 | 5.369 | 4.874 | 4.339 |
| **Pakistan** | **GDPrHGiniH** | 6.028 | 6.093 | 5.82 | 5.186 | 4.362 |
| **Pakistan** | **GDPrHGiniHv** | 7.198 | 7.281 | 6.98 | 6.318 | 5.503 |
| **Pakistan** | **GDPrHGiniLow** | 4.65 | 4.695 | 4.46 | 3.883 | 3.104 |
| **Pakistan** | **GDPrLGiniH** | 6.028 | 6.093 | 6.373 | 6.022 | 5.995 |
| **Pakistan** | **GDPrLGiniHv** | 7.198 | 7.281 | 7.603 | 7.276 | 7.422 |
| **Pakistan** | **GDPrLGiniLow** | 4.65 | 4.695 | 4.923 | 4.568 | 4.383 |
| **Pakistan** | **NoCOVIDginiB** | 4.831 | 4.972 | 4.87 | 4.462 | 3.632 |
| **Palestine** | **GDPrGiniBase** | 0.095 | 0.103 | 0.103 | 0.131 | 0.007 |
| **Palestine** | **GDPrHGiniH** | 0.109 | 0.118 | 0.112 | 0.135 | 0.007 |
| **Palestine** | **GDPrHGiniHv** | 0.133 | 0.143 | 0.136 | 0.164 | 0.011 |
| **Palestine** | **GDPrHGiniLow** | 0.083 | 0.089 | 0.084 | 0.102 | 0.004 |
| **Palestine** | **GDPrLGiniH** | 0.109 | 0.118 | 0.123 | 0.166 | 0.01 |
| **Palestine** | **GDPrLGiniHv** | 0.133 | 0.143 | 0.149 | 0.199 | 0.015 |
| **Palestine** | **GDPrLGiniLow** | 0.083 | 0.089 | 0.094 | 0.128 | 0.006 |
| **Palestine** | **NoCOVIDginiB** | 0.059 | 0.074 | 0.093 | 0.155 | 0.008 |
| **Panama** | **GDPrGiniBase** | 0.128 | 0.116 | 0.125 | 0.096 | 0.056 |
| **Panama** | **GDPrHGiniH** | 0.148 | 0.135 | 0.141 | 0.104 | 0.065 |
| **Panama** | **GDPrHGiniHv** | 0.181 | 0.167 | 0.174 | 0.132 | 0.086 |
| **Panama** | **GDPrHGiniLow** | 0.11 | 0.099 | 0.104 | 0.073 | 0.043 |
| **Panama** | **GDPrLGiniH** | 0.148 | 0.135 | 0.148 | 0.123 | 0.072 |
| **Panama** | **GDPrLGiniHv** | 0.181 | 0.167 | 0.182 | 0.155 | 0.095 |
| **Panama** | **GDPrLGiniLow** | 0.11 | 0.099 | 0.11 | 0.089 | 0.048 |
| **Panama** | **NoCOVIDginiB** | 0.082 | 0.077 | 0.072 | 0.05 | 0.038 |
| **Papua New Guinea** | **GDPrGiniBase** | 2.312 | 2.275 | 2.175 | 1.024 | 0.381 |
| **Papua New Guinea** | **GDPrHGiniH** | 2.403 | 2.368 | 2.22 | 1.06 | 0.404 |
| **Papua New Guinea** | **GDPrHGiniHv** | 2.538 | 2.506 | 2.361 | 1.189 | 0.492 |
| **Papua New Guinea** | **GDPrHGiniLow** | 2.22 | 2.182 | 2.032 | 0.896 | 0.303 |
| **Papua New Guinea** | **GDPrLGiniH** | 2.403 | 2.368 | 2.315 | 1.162 | 0.474 |
| **Papua New Guinea** | **GDPrLGiniHv** | 2.538 | 2.506 | 2.456 | 1.297 | 0.571 |
| **Papua New Guinea** | **GDPrLGiniLow** | 2.22 | 2.182 | 2.127 | 0.991 | 0.36 |
| **Papua New Guinea** | **NoCOVIDginiB** | 2.121 | 2.044 | 1.948 | 0.899 | 0.309 |
| **Paraguay** | **GDPrGiniBase** | 0.042 | 0.04 | 0.037 | 0.024 | 0.01 |
| **Paraguay** | **GDPrHGiniH** | 0.052 | 0.049 | 0.045 | 0.028 | 0.012 |
| **Paraguay** | **GDPrHGiniHv** | 0.071 | 0.067 | 0.061 | 0.039 | 0.018 |
| **Paraguay** | **GDPrHGiniLow** | 0.034 | 0.032 | 0.028 | 0.017 | 0.007 |
| **Paraguay** | **GDPrLGiniH** | 0.052 | 0.049 | 0.048 | 0.033 | 0.014 |
| **Paraguay** | **GDPrLGiniHv** | 0.071 | 0.067 | 0.065 | 0.047 | 0.021 |
| **Paraguay** | **GDPrLGiniLow** | 0.034 | 0.032 | 0.031 | 0.02 | 0.008 |
| **Paraguay** | **NoCOVIDginiB** | 0.037 | 0.035 | 0.033 | 0.021 | 0.008 |
| **Peru** | **GDPrGiniBase** | 1.478 | 1.368 | 1.345 | 0.847 | 0.521 |
| **Peru** | **GDPrHGiniH** | 1.659 | 1.542 | 1.479 | 0.92 | 0.583 |
| **Peru** | **GDPrHGiniHv** | 1.952 | 1.825 | 1.757 | 1.13 | 0.745 |
| **Peru** | **GDPrHGiniLow** | 1.308 | 1.205 | 1.15 | 0.68 | 0.407 |
| **Peru** | **GDPrLGiniH** | 1.659 | 1.542 | 1.559 | 1.04 | 0.654 |
| **Peru** | **GDPrLGiniHv** | 1.952 | 1.825 | 1.846 | 1.268 | 0.829 |
| **Peru** | **GDPrLGiniLow** | 1.308 | 1.205 | 1.219 | 0.778 | 0.461 |
| **Peru** | **NoCOVIDginiB** | 1.098 | 1.076 | 1.022 | 0.694 | 0.427 |
| **Philippines** | **GDPrGiniBase** | 6.477 | 6.728 | 6.915 | 5.712 | 2.93 |
| **Philippines** | **GDPrHGiniH** | 7.191 | 7.462 | 7.474 | 6.111 | 3.186 |
| **Philippines** | **GDPrHGiniHv** | 8.331 | 8.632 | 8.652 | 7.217 | 3.975 |
| **Philippines** | **GDPrHGiniLow** | 5.801 | 6.033 | 6.037 | 4.792 | 2.302 |
| **Philippines** | **GDPrLGiniH** | 7.191 | 7.462 | 7.859 | 6.741 | 3.658 |
| **Philippines** | **GDPrLGiniHv** | 8.331 | 8.632 | 9.069 | 7.911 | 4.521 |
| **Philippines** | **GDPrLGiniLow** | 5.801 | 6.033 | 6.378 | 5.336 | 2.679 |
| **Philippines** | **NoCOVIDginiB** | 4.586 | 4.447 | 4.339 | 4.089 | 1.83 |
| **Poland** | **GDPrGiniBase** | 0.078 | 0.071 | 0.065 | 0.013 | 0.001 |
| **Poland** | **GDPrHGiniH** | 0.098 | 0.089 | 0.079 | 0.015 | 0.001 |
| **Poland** | **GDPrHGiniHv** | 0.133 | 0.122 | 0.108 | 0.023 | 0.002 |
| **Poland** | **GDPrHGiniLow** | 0.062 | 0.056 | 0.049 | 0.008 | 0.001 |
| **Poland** | **GDPrLGiniH** | 0.098 | 0.089 | 0.084 | 0.02 | 0.002 |
| **Poland** | **GDPrLGiniHv** | 0.133 | 0.122 | 0.115 | 0.03 | 0.003 |
| **Poland** | **GDPrLGiniLow** | 0.062 | 0.056 | 0.053 | 0.011 | 0.001 |
| **Poland** | **NoCOVIDginiB** | 0.069 | 0.062 | 0.058 | 0.015 | 0.001 |
| **Portugal** | **GDPrGiniBase** | 0.039 | 0.037 | 0.037 | 0.02 | 0.005 |
| **Portugal** | **GDPrHGiniH** | 0.048 | 0.046 | 0.044 | 0.022 | 0.006 |
| **Portugal** | **GDPrHGiniHv** | 0.064 | 0.061 | 0.059 | 0.03 | 0.009 |
| **Portugal** | **GDPrHGiniLow** | 0.032 | 0.03 | 0.029 | 0.013 | 0.003 |
| **Portugal** | **GDPrLGiniH** | 0.048 | 0.046 | 0.047 | 0.03 | 0.008 |
| **Portugal** | **GDPrLGiniHv** | 0.064 | 0.061 | 0.062 | 0.042 | 0.012 |
| **Portugal** | **GDPrLGiniLow** | 0.032 | 0.03 | 0.031 | 0.019 | 0.004 |
| **Portugal** | **NoCOVIDginiB** | 0.032 | 0.031 | 0.029 | 0.018 | 0.005 |
| **Puerto Rico** | **GDPrGiniBase** | 0.0019 | 0.0017 | 0.0016 | 0.0005 | 0 |
| **Puerto Rico** | **GDPrHGiniH** | 0.0027 | 0.0023 | 0.0021 | 0.0008 | 0.0001 |
| **Puerto Rico** | **GDPrHGiniHv** | 0.004 | 0.004 | 0.003 | 0.001 | 0 |
| **Puerto Rico** | **GDPrHGiniLow** | 0.0014 | 0.0012 | 0.001 | 0.0003 | 0 |
| **Puerto Rico** | **GDPrLGiniH** | 0.0027 | 0.0023 | 0.0023 | 0.0009 | 0.0001 |
| **Puerto Rico** | **GDPrLGiniHv** | 0.004 | 0.004 | 0.004 | 0.002 | 0 |
| **Puerto Rico** | **GDPrLGiniLow** | 0.0014 | 0.0012 | 0.0011 | 0.0004 | 0 |
| **Puerto Rico** | **NoCOVIDginiB** | 0.0018 | 0.0016 | 0.0015 | 0.0005 | 0 |
| **Qatar** | **GDPrGiniBase** | 0.003 | 0.003 | 0.003 | 0.001 | 0 |
| **Qatar** | **GDPrHGiniH** | 0.004 | 0.005 | 0.004 | 0.001 | 0 |
| **Qatar** | **GDPrHGiniHv** | 0.006 | 0.007 | 0.006 | 0.002 | 0.001 |
| **Qatar** | **GDPrHGiniLow** | 0.0025 | 0.0026 | 0.0023 | 0.0006 | 0.0001 |
| **Qatar** | **GDPrLGiniH** | 0.004 | 0.005 | 0.004 | 0.002 | 0 |
| **Qatar** | **GDPrLGiniHv** | 0.006 | 0.007 | 0.006 | 0.002 | 0.001 |
| **Qatar** | **GDPrLGiniLow** | 0.0025 | 0.0026 | 0.0024 | 0.0008 | 0.0002 |
| **Qatar** | **NoCOVIDginiB** | 0.0028 | 0.0028 | 0.0026 | 0.0009 | 0.0002 |
| **Romania** | **GDPrGiniBase** | 0.463 | 0.409 | 0.371 | 0.187 | 0.056 |
| **Romania** | **GDPrHGiniH** | 0.527 | 0.466 | 0.408 | 0.202 | 0.061 |
| **Romania** | **GDPrHGiniHv** | 0.633 | 0.561 | 0.496 | 0.257 | 0.081 |
| **Romania** | **GDPrHGiniLow** | 0.404 | 0.355 | 0.304 | 0.142 | 0.039 |
| **Romania** | **GDPrLGiniH** | 0.527 | 0.466 | 0.443 | 0.242 | 0.076 |
| **Romania** | **GDPrLGiniHv** | 0.633 | 0.561 | 0.536 | 0.306 | 0.1 |
| **Romania** | **GDPrLGiniLow** | 0.404 | 0.355 | 0.333 | 0.173 | 0.05 |
| **Romania** | **NoCOVIDginiB** | 0.393 | 0.368 | 0.345 | 0.186 | 0.055 |
| **Russian Federation** | **GDPrGiniBase** | 0.176 | 0.159 | 0.151 | 0.051 | 0.017 |
| **Russian Federation** | **GDPrHGiniH** | 0.228 | 0.208 | 0.191 | 0.062 | 0.021 |
| **Russian Federation** | **GDPrHGiniHv** | 0.328 | 0.301 | 0.278 | 0.097 | 0.035 |
| **Russian Federation** | **GDPrHGiniLow** | 0.134 | 0.121 | 0.11 | 0.032 | 0.01 |
| **Russian Federation** | **GDPrLGiniH** | 0.228 | 0.208 | 0.204 | 0.079 | 0.027 |
| **Russian Federation** | **GDPrLGiniHv** | 0.328 | 0.301 | 0.295 | 0.121 | 0.045 |
| **Russian Federation** | **GDPrLGiniLow** | 0.134 | 0.121 | 0.118 | 0.042 | 0.013 |
| **Russian Federation** | **NoCOVIDginiB** | 0.158 | 0.145 | 0.133 | 0.048 | 0.016 |
| **Rwanda** | **GDPrGiniBase** | 7.047 | 7.302 | 7.308 | 7 | 3.218 |
| **Rwanda** | **GDPrHGiniH** | 7.128 | 7.386 | 7.281 | 6.917 | 3.262 |
| **Rwanda** | **GDPrHGiniHv** | 7.247 | 7.509 | 7.411 | 7.125 | 3.628 |
| **Rwanda** | **GDPrHGiniLow** | 6.965 | 7.217 | 7.105 | 6.633 | 2.792 |
| **Rwanda** | **GDPrLGiniH** | 7.128 | 7.386 | 7.508 | 7.355 | 3.685 |
| **Rwanda** | **GDPrLGiniHv** | 7.247 | 7.509 | 7.63 | 7.55 | 4.066 |
| **Rwanda** | **GDPrLGiniLow** | 6.965 | 7.217 | 7.343 | 7.086 | 3.191 |
| **Rwanda** | **NoCOVIDginiB** | 6.291 | 6.257 | 6.272 | 5.975 | 2.28 |
| **Samoa** | **GDPrGiniBase** | 0.0011 | 0.0014 | 0.0014 | 0.0006 | 0.0002 |
| **Samoa** | **GDPrHGiniH** | 0.0013 | 0.0017 | 0.0016 | 0.0007 | 0.0002 |
| **Samoa** | **GDPrHGiniHv** | 0.0017 | 0.0022 | 0.002 | 0.0009 | 0.0003 |
| **Samoa** | **GDPrHGiniLow** | 0.0009 | 0.0011 | 0.0011 | 0.0004 | 0.0001 |
| **Samoa** | **GDPrLGiniH** | 0.0013 | 0.0017 | 0.0017 | 0.0009 | 0.0003 |
| **Samoa** | **GDPrLGiniHv** | 0.0017 | 0.0022 | 0.0023 | 0.0012 | 0.0004 |
| **Samoa** | **GDPrLGiniLow** | 0.0009 | 0.0011 | 0.0012 | 0.0006 | 0.0001 |
| **Samoa** | **NoCOVIDginiB** | 0.0008 | 0.0007 | 0.0007 | 0.0003 | 0.0001 |
| **Sao Tome and Principe** | **GDPrGiniBase** | 0.08 | 0.084 | 0.086 | 0.099 | 0.084 |
| **Sao Tome and Principe** | **GDPrHGiniH** | 0.083 | 0.087 | 0.087 | 0.099 | 0.085 |
| **Sao Tome and Principe** | **GDPrHGiniHv** | 0.087 | 0.091 | 0.092 | 0.105 | 0.093 |
| **Sao Tome and Principe** | **GDPrHGiniLow** | 0.077 | 0.081 | 0.081 | 0.092 | 0.075 |
| **Sao Tome and Principe** | **GDPrLGiniH** | 0.083 | 0.087 | 0.09 | 0.106 | 0.094 |
| **Sao Tome and Principe** | **GDPrLGiniHv** | 0.087 | 0.091 | 0.094 | 0.111 | 0.102 |
| **Sao Tome and Principe** | **GDPrLGiniLow** | 0.077 | 0.081 | 0.084 | 0.099 | 0.083 |
| **Sao Tome and Principe** | **NoCOVIDginiB** | 0.079 | 0.082 | 0.084 | 0.095 | 0.078 |
| **Saudi Arabia** | **GDPrGiniBase** | 0.043 | 0.045 | 0.041 | 0.016 | 0.007 |
| **Saudi Arabia** | **GDPrHGiniH** | 0.056 | 0.058 | 0.05 | 0.02 | 0.009 |
| **Saudi Arabia** | **GDPrHGiniHv** | 0.08 | 0.083 | 0.073 | 0.031 | 0.015 |
| **Saudi Arabia** | **GDPrHGiniLow** | 0.033 | 0.034 | 0.029 | 0.011 | 0.004 |
| **Saudi Arabia** | **GDPrLGiniH** | 0.056 | 0.058 | 0.055 | 0.024 | 0.012 |
| **Saudi Arabia** | **GDPrLGiniHv** | 0.08 | 0.083 | 0.079 | 0.036 | 0.019 |
| **Saudi Arabia** | **GDPrLGiniLow** | 0.033 | 0.034 | 0.032 | 0.013 | 0.006 |
| **Saudi Arabia** | **NoCOVIDginiB** | 0.035 | 0.034 | 0.033 | 0.015 | 0.007 |
| **Senegal** | **GDPrGiniBase** | 4.975 | 5.076 | 5.062 | 4.391 | 1.192 |
| **Senegal** | **GDPrHGiniH** | 5.137 | 5.245 | 5.102 | 4.388 | 1.241 |
| **Senegal** | **GDPrHGiniHv** | 5.378 | 5.494 | 5.359 | 4.704 | 1.482 |
| **Senegal** | **GDPrHGiniLow** | 4.811 | 4.907 | 4.756 | 3.97 | 0.958 |
| **Senegal** | **GDPrLGiniH** | 5.137 | 5.245 | 5.357 | 4.813 | 1.463 |
| **Senegal** | **GDPrLGiniHv** | 5.378 | 5.494 | 5.61 | 5.13 | 1.728 |
| **Senegal** | **GDPrLGiniLow** | 4.811 | 4.907 | 5.014 | 4.39 | 1.146 |
| **Senegal** | **NoCOVIDginiB** | 4.564 | 4.483 | 4.312 | 3.638 | 0.848 |
| **Serbia** | **GDPrGiniBase** | 0.358 | 0.321 | 0.288 | 0.114 | 0.028 |
| **Serbia** | **GDPrHGiniH** | 0.399 | 0.36 | 0.309 | 0.121 | 0.031 |
| **Serbia** | **GDPrHGiniHv** | 0.465 | 0.421 | 0.365 | 0.151 | 0.042 |
| **Serbia** | **GDPrHGiniLow** | 0.319 | 0.285 | 0.242 | 0.088 | 0.02 |
| **Serbia** | **GDPrLGiniH** | 0.399 | 0.36 | 0.331 | 0.145 | 0.037 |
| **Serbia** | **GDPrLGiniHv** | 0.465 | 0.421 | 0.39 | 0.179 | 0.05 |
| **Serbia** | **GDPrLGiniLow** | 0.319 | 0.285 | 0.261 | 0.106 | 0.024 |
| **Serbia** | **NoCOVIDginiB** | 0.329 | 0.301 | 0.275 | 0.112 | 0.027 |
| **Seychelles** | **GDPrGiniBase** | 0.0013 | 0.0012 | 0.0011 | 0.0007 | 0.0007 |
| **Seychelles** | **GDPrHGiniH** | 0.0015 | 0.0015 | 0.0013 | 0.0008 | 0.0007 |
| **Seychelles** | **GDPrHGiniHv** | 0.002 | 0.0019 | 0.0017 | 0.0011 | 0.001 |
| **Seychelles** | **GDPrHGiniLow** | 0.0011 | 0.001 | 0.0009 | 0.0005 | 0.0005 |
| **Seychelles** | **GDPrLGiniH** | 0.0015 | 0.0015 | 0.0014 | 0.001 | 0.0009 |
| **Seychelles** | **GDPrLGiniHv** | 0.002 | 0.0019 | 0.0018 | 0.0013 | 0.0012 |
| **Seychelles** | **GDPrLGiniLow** | 0.0011 | 0.001 | 0.001 | 0.0007 | 0.0006 |
| **Seychelles** | **NoCOVIDginiB** | 0.0008 | 0.0009 | 0.0009 | 0.0007 | 0.0006 |
| **Sierra Leone** | **GDPrGiniBase** | 3.62 | 3.797 | 3.792 | 4.106 | 3.332 |
| **Sierra Leone** | **GDPrHGiniH** | 3.672 | 3.85 | 3.748 | 3.988 | 3.048 |
| **Sierra Leone** | **GDPrHGiniHv** | 3.75 | 3.927 | 3.831 | 4.098 | 3.26 |
| **Sierra Leone** | **GDPrHGiniLow** | 3.566 | 3.744 | 3.634 | 3.838 | 2.756 |
| **Sierra Leone** | **GDPrLGiniH** | 3.672 | 3.85 | 3.948 | 4.395 | 3.955 |
| **Sierra Leone** | **GDPrLGiniHv** | 3.75 | 3.927 | 4.025 | 4.495 | 4.172 |
| **Sierra Leone** | **GDPrLGiniLow** | 3.566 | 3.744 | 3.843 | 4.259 | 3.66 |
| **Sierra Leone** | **NoCOVIDginiB** | 3.273 | 3.351 | 3.47 | 3.635 | 2.313 |
| **Singapore** | **GDPrGiniBase** | 0.006 | 0.005 | 0.005 | 0.001 | 0 |
| **Singapore** | **GDPrHGiniH** | 0.008 | 0.007 | 0.006 | 0.001 | 0 |
| **Singapore** | **GDPrHGiniHv** | 0.011 | 0.01 | 0.009 | 0.002 | 0 |
| **Singapore** | **GDPrHGiniLow** | 0.004 | 0.004 | 0.003 | 0.001 | 0 |
| **Singapore** | **GDPrLGiniH** | 0.008 | 0.007 | 0.006 | 0.002 | 0 |
| **Singapore** | **GDPrLGiniHv** | 0.011 | 0.01 | 0.009 | 0.003 | 0 |
| **Singapore** | **GDPrLGiniLow** | 0.004 | 0.004 | 0.004 | 0.001 | 0 |
| **Singapore** | **NoCOVIDginiB** | 0.005 | 0.005 | 0.005 | 0.001 | 0 |
| **Slovakia** | **GDPrGiniBase** | 0.06 | 0.059 | 0.058 | 0.017 | 0.003 |
| **Slovakia** | **GDPrHGiniH** | 0.069 | 0.068 | 0.065 | 0.018 | 0.003 |
| **Slovakia** | **GDPrHGiniHv** | 0.085 | 0.083 | 0.08 | 0.024 | 0.004 |
| **Slovakia** | **GDPrHGiniLow** | 0.051 | 0.05 | 0.048 | 0.012 | 0.002 |
| **Slovakia** | **GDPrLGiniH** | 0.069 | 0.068 | 0.07 | 0.024 | 0.004 |
| **Slovakia** | **GDPrLGiniHv** | 0.085 | 0.083 | 0.086 | 0.031 | 0.006 |
| **Slovakia** | **GDPrLGiniLow** | 0.051 | 0.05 | 0.052 | 0.016 | 0.003 |
| **Slovakia** | **NoCOVIDginiB** | 0.049 | 0.048 | 0.046 | 0.017 | 0.002 |
| **Slovenia** | **GDPrGiniBase** | 0.0013 | 0.0011 | 0.001 | 0.0002 | 0 |
| **Slovenia** | **GDPrHGiniH** | 0.0016 | 0.0014 | 0.0013 | 0.0002 | 0 |
| **Slovenia** | **GDPrHGiniHv** | 0.0024 | 0.0021 | 0.0019 | 0.0003 | 0 |
| **Slovenia** | **GDPrHGiniLow** | 0.001 | 0.0008 | 0.0007 | 0.0001 | 0 |
| **Slovenia** | **GDPrLGiniH** | 0.0016 | 0.0014 | 0.0014 | 0.0003 | 0 |
| **Slovenia** | **GDPrLGiniHv** | 0.0024 | 0.0021 | 0.0021 | 0.0005 | 0 |
| **Slovenia** | **GDPrLGiniLow** | 0.001 | 0.0008 | 0.0008 | 0.0002 | 0 |
| **Slovenia** | **NoCOVIDginiB** | 0.001 | 0.0009 | 0.0008 | 0.0002 | 0 |
| **Solomon Islands** | **GDPrGiniBase** | 0.194 | 0.202 | 0.198 | 0.183 | 0.146 |
| **Solomon Islands** | **GDPrHGiniH** | 0.2 | 0.208 | 0.199 | 0.179 | 0.137 |
| **Solomon Islands** | **GDPrHGiniHv** | 0.209 | 0.218 | 0.209 | 0.19 | 0.152 |
| **Solomon Islands** | **GDPrHGiniLow** | 0.187 | 0.195 | 0.186 | 0.163 | 0.118 |
| **Solomon Islands** | **GDPrLGiniH** | 0.2 | 0.208 | 0.208 | 0.201 | 0.175 |
| **Solomon Islands** | **GDPrLGiniHv** | 0.209 | 0.218 | 0.218 | 0.213 | 0.191 |
| **Solomon Islands** | **GDPrLGiniLow** | 0.187 | 0.195 | 0.195 | 0.186 | 0.154 |
| **Solomon Islands** | **NoCOVIDginiB** | 0.171 | 0.174 | 0.176 | 0.166 | 0.113 |
| **Somalia** | **GDPrGiniBase** | 6.342 | 6.801 | 7.098 | 7.524 | 1.612 |
| **Somalia** | **GDPrHGiniH** | 6.475 | 6.936 | 7.058 | 7.417 | 1.645 |
| **Somalia** | **GDPrHGiniHv** | 6.671 | 7.133 | 7.26 | 7.663 | 1.971 |
| **Somalia** | **GDPrHGiniLow** | 6.207 | 6.665 | 6.781 | 7.083 | 1.264 |
| **Somalia** | **GDPrLGiniH** | 6.475 | 6.936 | 7.415 | 7.952 | 2.042 |
| **Somalia** | **GDPrLGiniHv** | 6.671 | 7.133 | 7.607 | 8.187 | 2.412 |
| **Somalia** | **GDPrLGiniLow** | 6.207 | 6.665 | 7.151 | 7.637 | 1.6 |
| **Somalia** | **NoCOVIDginiB** | 6.091 | 6.504 | 7.041 | 7.221 | 1.464 |
| **South Africa** | **GDPrGiniBase** | 12.03 | 11.9 | 11.93 | 12.33 | 12.39 |
| **South Africa** | **GDPrHGiniH** | 12.97 | 12.84 | 12.76 | 13.08 | 13.2 |
| **South Africa** | **GDPrHGiniHv** | 14.42 | 14.32 | 14.24 | 14.68 | 14.97 |
| **South Africa** | **GDPrHGiniLow** | 11.12 | 10.98 | 10.88 | 11.08 | 11 |
| **South Africa** | **GDPrLGiniH** | 12.97 | 12.84 | 13.02 | 13.66 | 13.87 |
| **South Africa** | **GDPrLGiniHv** | 14.42 | 14.32 | 14.51 | 15.27 | 15.67 |
| **South Africa** | **GDPrLGiniLow** | 11.12 | 10.98 | 11.13 | 11.62 | 11.63 |
| **South Africa** | **NoCOVIDginiB** | 11.38 | 11.47 | 11.52 | 11.91 | 11.84 |
| **Spain** | **GDPrGiniBase** | 0.339 | 0.324 | 0.315 | 0.149 | 0.045 |
| **Spain** | **GDPrHGiniH** | 0.405 | 0.388 | 0.368 | 0.163 | 0.052 |
| **Spain** | **GDPrHGiniHv** | 0.52 | 0.499 | 0.475 | 0.221 | 0.075 |
| **Spain** | **GDPrHGiniLow** | 0.281 | 0.268 | 0.253 | 0.104 | 0.03 |
| **Spain** | **GDPrLGiniH** | 0.405 | 0.388 | 0.387 | 0.211 | 0.067 |
| **Spain** | **GDPrLGiniHv** | 0.52 | 0.499 | 0.498 | 0.281 | 0.095 |
| **Spain** | **GDPrLGiniLow** | 0.281 | 0.268 | 0.267 | 0.138 | 0.04 |
| **Spain** | **NoCOVIDginiB** | 0.267 | 0.252 | 0.237 | 0.129 | 0.039 |
| **Sri Lanka** | **GDPrGiniBase** | 0.178 | 0.167 | 0.156 | 0.086 | 0.019 |
| **Sri Lanka** | **GDPrHGiniH** | 0.214 | 0.201 | 0.18 | 0.097 | 0.023 |
| **Sri Lanka** | **GDPrHGiniHv** | 0.276 | 0.26 | 0.235 | 0.132 | 0.035 |
| **Sri Lanka** | **GDPrHGiniLow** | 0.147 | 0.137 | 0.122 | 0.062 | 0.013 |
| **Sri Lanka** | **GDPrLGiniH** | 0.214 | 0.201 | 0.196 | 0.116 | 0.028 |
| **Sri Lanka** | **GDPrLGiniHv** | 0.276 | 0.26 | 0.254 | 0.156 | 0.042 |
| **Sri Lanka** | **GDPrLGiniLow** | 0.147 | 0.137 | 0.133 | 0.075 | 0.016 |
| **Sri Lanka** | **NoCOVIDginiB** | 0.142 | 0.128 | 0.113 | 0.062 | 0.013 |
| **St. Lucia** | **GDPrGiniBase** | 0.013 | 0.014 | 0.013 | 0.008 | 0.005 |
| **St. Lucia** | **GDPrHGiniH** | 0.015 | 0.016 | 0.014 | 0.009 | 0.005 |
| **St. Lucia** | **GDPrHGiniHv** | 0.017 | 0.018 | 0.016 | 0.011 | 0.007 |
| **St. Lucia** | **GDPrHGiniLow** | 0.012 | 0.013 | 0.011 | 0.007 | 0.004 |
| **St. Lucia** | **GDPrLGiniH** | 0.015 | 0.016 | 0.014 | 0.01 | 0.006 |
| **St. Lucia** | **GDPrLGiniHv** | 0.017 | 0.018 | 0.017 | 0.012 | 0.007 |
| **St. Lucia** | **GDPrLGiniLow** | 0.012 | 0.013 | 0.012 | 0.007 | 0.004 |
| **St. Lucia** | **NoCOVIDginiB** | 0.008 | 0.009 | 0.009 | 0.007 | 0.004 |
| **St. Vincent and the Grenadines** | **GDPrGiniBase** | 0.013 | 0.016 | 0.014 | 0.013 | 0.008 |
| **St. Vincent and the Grenadines** | **GDPrHGiniH** | 0.014 | 0.017 | 0.015 | 0.014 | 0.009 |
| **St. Vincent and the Grenadines** | **GDPrHGiniHv** | 0.016 | 0.018 | 0.016 | 0.015 | 0.01 |
| **St. Vincent and the Grenadines** | **GDPrHGiniLow** | 0.012 | 0.015 | 0.013 | 0.012 | 0.007 |
| **St. Vincent and the Grenadines** | **GDPrLGiniH** | 0.014 | 0.017 | 0.016 | 0.015 | 0.009 |
| **St. Vincent and the Grenadines** | **GDPrLGiniHv** | 0.016 | 0.018 | 0.017 | 0.016 | 0.011 |
| **St. Vincent and the Grenadines** | **GDPrLGiniLow** | 0.012 | 0.015 | 0.014 | 0.013 | 0.008 |
| **St. Vincent and the Grenadines** | **NoCOVIDginiB** | 0.012 | 0.012 | 0.012 | 0.012 | 0.007 |
| **Sudan** | **GDPrGiniBase** | 6.838 | 7.442 | 7.333 | 5.741 | 3.536 |
| **Sudan** | **GDPrHGiniH** | 7.207 | 7.826 | 7.483 | 5.864 | 3.56 |
| **Sudan** | **GDPrHGiniHv** | 7.764 | 8.404 | 8.067 | 6.485 | 4.171 |
| **Sudan** | **GDPrHGiniLow** | 6.471 | 7.059 | 6.71 | 5.064 | 2.823 |
| **Sudan** | **GDPrLGiniH** | 7.207 | 7.826 | 7.975 | 6.456 | 4.377 |
| **Sudan** | **GDPrLGiniHv** | 7.764 | 8.404 | 8.567 | 7.101 | 5.065 |
| **Sudan** | **GDPrLGiniLow** | 6.471 | 7.059 | 7.189 | 5.621 | 3.535 |
| **Sudan** | **NoCOVIDginiB** | 6.431 | 7.34 | 7.883 | 6.732 | 4.606 |
| **Sudan South** | **GDPrGiniBase** | 8.214 | 7.744 | 7.511 | 7.986 | 5.531 |
| **Sudan South** | **GDPrHGiniH** | 8.207 | 7.741 | 7.471 | 7.887 | 5.442 |
| **Sudan South** | **GDPrHGiniHv** | 8.2 | 7.738 | 7.473 | 7.918 | 5.711 |
| **Sudan South** | **GDPrHGiniLow** | 8.222 | 7.749 | 7.472 | 7.847 | 5.075 |
| **Sudan South** | **GDPrLGiniH** | 8.207 | 7.741 | 7.543 | 8.115 | 5.993 |
| **Sudan South** | **GDPrLGiniHv** | 8.2 | 7.738 | 7.543 | 8.138 | 6.254 |
| **Sudan South** | **GDPrLGiniLow** | 8.222 | 7.749 | 7.547 | 8.088 | 5.639 |
| **Sudan South** | **NoCOVIDginiB** | 7.79 | 7.348 | 7.21 | 7.365 | 4.445 |
| **Suriname** | **GDPrGiniBase** | 0.117 | 0.117 | 0.123 | 0.096 | 0.07 |
| **Suriname** | **GDPrHGiniH** | 0.126 | 0.125 | 0.129 | 0.1 | 0.075 |
| **Suriname** | **GDPrHGiniHv** | 0.138 | 0.138 | 0.142 | 0.113 | 0.087 |
| **Suriname** | **GDPrHGiniLow** | 0.109 | 0.109 | 0.113 | 0.084 | 0.061 |
| **Suriname** | **GDPrLGiniH** | 0.126 | 0.125 | 0.133 | 0.107 | 0.081 |
| **Suriname** | **GDPrLGiniHv** | 0.138 | 0.138 | 0.146 | 0.12 | 0.093 |
| **Suriname** | **GDPrLGiniLow** | 0.109 | 0.109 | 0.117 | 0.091 | 0.066 |
| **Suriname** | **NoCOVIDginiB** | 0.094 | 0.091 | 0.088 | 0.07 | 0.052 |
| **Sweden** | **GDPrGiniBase** | 0.022 | 0.023 | 0.023 | 0.01 | 0.001 |
| **Sweden** | **GDPrHGiniH** | 0.027 | 0.028 | 0.028 | 0.011 | 0.001 |
| **Sweden** | **GDPrHGiniHv** | 0.037 | 0.038 | 0.037 | 0.016 | 0.002 |
| **Sweden** | **GDPrHGiniLow** | 0.018 | 0.018 | 0.018 | 0.006 | 0.001 |
| **Sweden** | **GDPrLGiniH** | 0.027 | 0.028 | 0.029 | 0.015 | 0.002 |
| **Sweden** | **GDPrLGiniHv** | 0.037 | 0.038 | 0.04 | 0.021 | 0.003 |
| **Sweden** | **GDPrLGiniLow** | 0.018 | 0.018 | 0.019 | 0.009 | 0.001 |
| **Sweden** | **NoCOVIDginiB** | 0.02 | 0.021 | 0.02 | 0.01 | 0.001 |
| **Switzerland** | **GDPrGiniBase** | 0.008 | 0.007 | 0.007 | 0.004 | 0.001 |
| **Switzerland** | **GDPrHGiniH** | 0.011 | 0.009 | 0.008 | 0.005 | 0.001 |
| **Switzerland** | **GDPrHGiniHv** | 0.015 | 0.014 | 0.012 | 0.007 | 0.002 |
| **Switzerland** | **GDPrHGiniLow** | 0.006 | 0.005 | 0.005 | 0.003 | 0.001 |
| **Switzerland** | **GDPrLGiniH** | 0.011 | 0.009 | 0.009 | 0.006 | 0.002 |
| **Switzerland** | **GDPrLGiniHv** | 0.015 | 0.014 | 0.013 | 0.009 | 0.003 |
| **Switzerland** | **GDPrLGiniLow** | 0.006 | 0.005 | 0.005 | 0.003 | 0.001 |
| **Switzerland** | **NoCOVIDginiB** | 0.007 | 0.007 | 0.007 | 0.004 | 0.001 |
| **Syrian Arab Republic** | **GDPrGiniBase** | 4.94 | 5.323 | 5.943 | 7.205 | 8.04 |
| **Syrian Arab Republic** | **GDPrHGiniH** | 5.087 | 5.47 | 5.969 | 7.164 | 7.873 |
| **Syrian Arab Republic** | **GDPrHGiniHv** | 5.303 | 5.686 | 6.187 | 7.478 | 8.265 |
| **Syrian Arab Republic** | **GDPrHGiniLow** | 4.792 | 5.175 | 5.671 | 6.737 | 7.343 |
| **Syrian Arab Republic** | **GDPrLGiniH** | 5.087 | 5.47 | 6.214 | 7.681 | 8.753 |
| **Syrian Arab Republic** | **GDPrLGiniHv** | 5.303 | 5.686 | 6.427 | 7.988 | 9.137 |
| **Syrian Arab Republic** | **GDPrLGiniLow** | 4.792 | 5.175 | 5.922 | 7.262 | 8.23 |
| **Syrian Arab Republic** | **NoCOVIDginiB** | 4.945 | 5.334 | 5.96 | 7.236 | 7.816 |
| **Taiwan** | **GDPrGiniBase** | 0.013 | 0.009 | 0.007 | 0.001 | 0 |
| **Taiwan** | **GDPrHGiniH** | 0.017 | 0.012 | 0.009 | 0.002 | 0 |
| **Taiwan** | **GDPrHGiniHv** | 0.025 | 0.017 | 0.013 | 0.003 | 0 |
| **Taiwan** | **GDPrHGiniLow** | 0.01 | 0.007 | 0.005 | 0.001 | 0 |
| **Taiwan** | **GDPrLGiniH** | 0.017 | 0.012 | 0.01 | 0.002 | 0 |
| **Taiwan** | **GDPrLGiniHv** | 0.025 | 0.017 | 0.016 | 0.004 | 0 |
| **Taiwan** | **GDPrLGiniLow** | 0.01 | 0.007 | 0.006 | 0.001 | 0 |
| **Taiwan** | **NoCOVIDginiB** | 0.014 | 0.012 | 0.01 | 0.002 | 0 |
| **Tajikistan** | **GDPrGiniBase** | 0.193 | 0.195 | 0.197 | 0.123 | 0.008 |
| **Tajikistan** | **GDPrHGiniH** | 0.221 | 0.223 | 0.21 | 0.129 | 0.009 |
| **Tajikistan** | **GDPrHGiniHv** | 0.266 | 0.27 | 0.255 | 0.162 | 0.013 |
| **Tajikistan** | **GDPrHGiniLow** | 0.167 | 0.169 | 0.158 | 0.092 | 0.005 |
| **Tajikistan** | **GDPrLGiniH** | 0.221 | 0.223 | 0.238 | 0.159 | 0.012 |
| **Tajikistan** | **GDPrLGiniHv** | 0.266 | 0.27 | 0.287 | 0.198 | 0.018 |
| **Tajikistan** | **GDPrLGiniLow** | 0.167 | 0.169 | 0.181 | 0.116 | 0.007 |
| **Tajikistan** | **NoCOVIDginiB** | 0.187 | 0.196 | 0.209 | 0.126 | 0.008 |
| **Tanzania** | **GDPrGiniBase** | 27.67 | 28.58 | 28.86 | 25.37 | 15.99 |
| **Tanzania** | **GDPrHGiniH** | 28.1 | 29.03 | 28.8 | 25.06 | 15.72 |
| **Tanzania** | **GDPrHGiniHv** | 28.74 | 29.7 | 29.49 | 26.13 | 17.49 |
| **Tanzania** | **GDPrHGiniLow** | 27.23 | 28.12 | 27.86 | 23.61 | 13.43 |
| **Tanzania** | **GDPrLGiniH** | 28.1 | 29.03 | 29.84 | 27.14 | 18.83 |
| **Tanzania** | **GDPrLGiniHv** | 28.74 | 29.7 | 30.5 | 28.18 | 20.72 |
| **Tanzania** | **GDPrLGiniLow** | 27.23 | 28.12 | 28.94 | 25.73 | 16.38 |
| **Tanzania** | **NoCOVIDginiB** | 26.37 | 26.51 | 26.56 | 22.5 | 12.51 |
| **Thailand** | **GDPrGiniBase** | 0.091 | 0.1 | 0.097 | 0.019 | 0.005 |
| **Thailand** | **GDPrHGiniH** | 0.118 | 0.128 | 0.117 | 0.024 | 0.007 |
| **Thailand** | **GDPrHGiniHv** | 0.168 | 0.182 | 0.167 | 0.038 | 0.012 |
| **Thailand** | **GDPrHGiniLow** | 0.07 | 0.076 | 0.069 | 0.012 | 0.003 |
| **Thailand** | **GDPrLGiniH** | 0.118 | 0.128 | 0.132 | 0.03 | 0.009 |
| **Thailand** | **GDPrLGiniHv** | 0.168 | 0.182 | 0.188 | 0.046 | 0.015 |
| **Thailand** | **GDPrLGiniLow** | 0.07 | 0.076 | 0.079 | 0.015 | 0.004 |
| **Thailand** | **NoCOVIDginiB** | 0.062 | 0.058 | 0.053 | 0.014 | 0.003 |
| **Timor-Leste** | **GDPrGiniBase** | 0.13 | 0.136 | 0.158 | 0.044 | 0 |
| **Timor-Leste** | **GDPrHGiniH** | 0.139 | 0.145 | 0.153 | 0.041 | 0 |
| **Timor-Leste** | **GDPrHGiniHv** | 0.152 | 0.158 | 0.167 | 0.049 | 0 |
| **Timor-Leste** | **GDPrHGiniLow** | 0.122 | 0.127 | 0.134 | 0.032 | 0 |
| **Timor-Leste** | **GDPrLGiniH** | 0.139 | 0.145 | 0.177 | 0.055 | 0 |
| **Timor-Leste** | **GDPrLGiniHv** | 0.152 | 0.158 | 0.192 | 0.064 | 0 |
| **Timor-Leste** | **GDPrLGiniLow** | 0.122 | 0.127 | 0.158 | 0.044 | 0 |
| **Timor-Leste** | **NoCOVIDginiB** | 0.145 | 0.155 | 0.193 | 0.044 | 0 |
| **Togo** | **GDPrGiniBase** | 3.829 | 3.927 | 3.956 | 4.219 | 1.462 |
| **Togo** | **GDPrHGiniH** | 3.894 | 3.994 | 3.963 | 4.159 | 1.452 |
| **Togo** | **GDPrHGiniHv** | 3.991 | 4.093 | 4.067 | 4.301 | 1.644 |
| **Togo** | **GDPrHGiniLow** | 3.762 | 3.859 | 3.82 | 3.964 | 1.212 |
| **Togo** | **GDPrLGiniH** | 3.894 | 3.994 | 4.091 | 4.469 | 1.739 |
| **Togo** | **GDPrLGiniHv** | 3.991 | 4.093 | 4.191 | 4.603 | 1.947 |
| **Togo** | **GDPrLGiniLow** | 3.762 | 3.859 | 3.953 | 4.285 | 1.476 |
| **Togo** | **NoCOVIDginiB** | 3.66 | 3.719 | 3.809 | 4.089 | 1.318 |
| **Tonga** | **GDPrGiniBase** | 0.001 | 0.002 | 0.002 | 0.004 | 0.003 |
| **Tonga** | **GDPrHGiniH** | 0.001 | 0.002 | 0.002 | 0.003 | 0.003 |
| **Tonga** | **GDPrHGiniHv** | 0.002 | 0.002 | 0.002 | 0.004 | 0.003 |
| **Tonga** | **GDPrHGiniLow** | 0.001 | 0.0013 | 0.0013 | 0.0026 | 0.002 |
| **Tonga** | **GDPrLGiniH** | 0.001 | 0.002 | 0.002 | 0.005 | 0.003 |
| **Tonga** | **GDPrLGiniHv** | 0.002 | 0.002 | 0.002 | 0.006 | 0.004 |
| **Tonga** | **GDPrLGiniLow** | 0.001 | 0.001 | 0.001 | 0.004 | 0.002 |
| **Tonga** | **NoCOVIDginiB** | 0.001 | 0.001 | 0.001 | 0.003 | 0.002 |
| **Trinidad and Tobago** | **GDPrGiniBase** | 0.006 | 0.007 | 0.007 | 0.004 | 0.002 |
| **Trinidad and Tobago** | **GDPrHGiniH** | 0.007 | 0.009 | 0.009 | 0.004 | 0.002 |
| **Trinidad and Tobago** | **GDPrHGiniHv** | 0.01 | 0.012 | 0.011 | 0.006 | 0.003 |
| **Trinidad and Tobago** | **GDPrHGiniLow** | 0.005 | 0.006 | 0.006 | 0.003 | 0.001 |
| **Trinidad and Tobago** | **GDPrLGiniH** | 0.007 | 0.009 | 0.009 | 0.005 | 0.002 |
| **Trinidad and Tobago** | **GDPrLGiniHv** | 0.01 | 0.012 | 0.013 | 0.007 | 0.003 |
| **Trinidad and Tobago** | **GDPrLGiniLow** | 0.005 | 0.006 | 0.006 | 0.003 | 0.001 |
| **Trinidad and Tobago** | **NoCOVIDginiB** | 0.004 | 0.005 | 0.005 | 0.003 | 0.001 |
| **Tunisia** | **GDPrGiniBase** | 0.047 | 0.051 | 0.057 | 0.085 | 0.037 |
| **Tunisia** | **GDPrHGiniH** | 0.057 | 0.062 | 0.066 | 0.088 | 0.041 |
| **Tunisia** | **GDPrHGiniHv** | 0.076 | 0.082 | 0.086 | 0.114 | 0.056 |
| **Tunisia** | **GDPrHGiniLow** | 0.038 | 0.041 | 0.044 | 0.061 | 0.026 |
| **Tunisia** | **GDPrLGiniH** | 0.057 | 0.062 | 0.072 | 0.115 | 0.05 |
| **Tunisia** | **GDPrLGiniHv** | 0.076 | 0.082 | 0.094 | 0.146 | 0.067 |
| **Tunisia** | **GDPrLGiniLow** | 0.038 | 0.041 | 0.048 | 0.08 | 0.032 |
| **Tunisia** | **NoCOVIDginiB** | 0.032 | 0.033 | 0.033 | 0.046 | 0.016 |
| **Turkey** | **GDPrGiniBase** | 0.132 | 0.108 | 0.098 | 0.043 | 0.008 |
| **Turkey** | **GDPrHGiniH** | 0.172 | 0.141 | 0.123 | 0.053 | 0.01 |
| **Turkey** | **GDPrHGiniHv** | 0.248 | 0.206 | 0.181 | 0.083 | 0.017 |
| **Turkey** | **GDPrHGiniLow** | 0.1 | 0.081 | 0.069 | 0.028 | 0.004 |
| **Turkey** | **GDPrLGiniH** | 0.172 | 0.141 | 0.132 | 0.064 | 0.013 |
| **Turkey** | **GDPrLGiniHv** | 0.248 | 0.206 | 0.194 | 0.099 | 0.023 |
| **Turkey** | **GDPrLGiniLow** | 0.1 | 0.081 | 0.075 | 0.034 | 0.006 |
| **Turkey** | **NoCOVIDginiB** | 0.129 | 0.128 | 0.122 | 0.054 | 0.011 |
| **Turkmenistan** | **GDPrGiniBase** | 0.047 | 0.038 | 0.035 | 0.009 | 0 |
| **Turkmenistan** | **GDPrHGiniH** | 0.056 | 0.046 | 0.041 | 0.011 | 0.001 |
| **Turkmenistan** | **GDPrHGiniHv** | 0.073 | 0.061 | 0.055 | 0.016 | 0.001 |
| **Turkmenistan** | **GDPrHGiniLow** | 0.038 | 0.031 | 0.027 | 0.006 | 0 |
| **Turkmenistan** | **GDPrLGiniH** | 0.056 | 0.046 | 0.045 | 0.013 | 0.001 |
| **Turkmenistan** | **GDPrLGiniHv** | 0.073 | 0.061 | 0.059 | 0.019 | 0.001 |
| **Turkmenistan** | **GDPrLGiniLow** | 0.038 | 0.031 | 0.03 | 0.008 | 0 |
| **Turkmenistan** | **NoCOVIDginiB** | 0.035 | 0.027 | 0.021 | 0.004 | 0 |
| **Uganda** | **GDPrGiniBase** | 22.17 | 23.42 | 23.73 | 21.4 | 14.08 |
| **Uganda** | **GDPrHGiniH** | 22.58 | 23.85 | 23.77 | 21.26 | 14.01 |
| **Uganda** | **GDPrHGiniHv** | 23.19 | 24.48 | 24.44 | 22.17 | 15.52 |
| **Uganda** | **GDPrHGiniLow** | 21.75 | 22.98 | 22.85 | 20.02 | 12.04 |
| **Uganda** | **GDPrLGiniH** | 22.58 | 23.85 | 24.59 | 22.75 | 16.22 |
| **Uganda** | **GDPrLGiniHv** | 23.19 | 24.48 | 25.23 | 23.62 | 17.8 |
| **Uganda** | **GDPrLGiniLow** | 21.75 | 22.98 | 23.7 | 21.57 | 14.16 |
| **Uganda** | **NoCOVIDginiB** | 21.44 | 22.24 | 22.36 | 19.81 | 11.61 |
| **Ukraine** | **GDPrGiniBase** | 0.039 | 0.037 | 0.032 | 0.005 | 0.002 |
| **Ukraine** | **GDPrHGiniH** | 0.05 | 0.048 | 0.039 | 0.006 | 0.003 |
| **Ukraine** | **GDPrHGiniHv** | 0.071 | 0.068 | 0.056 | 0.01 | 0.004 |
| **Ukraine** | **GDPrHGiniLow** | 0.03 | 0.029 | 0.023 | 0.003 | 0.001 |
| **Ukraine** | **GDPrLGiniH** | 0.05 | 0.048 | 0.044 | 0.009 | 0.003 |
| **Ukraine** | **GDPrLGiniHv** | 0.071 | 0.068 | 0.063 | 0.014 | 0.005 |
| **Ukraine** | **GDPrLGiniLow** | 0.03 | 0.029 | 0.026 | 0.005 | 0.001 |
| **Ukraine** | **NoCOVIDginiB** | 0.028 | 0.024 | 0.02 | 0.003 | 0.002 |
| **United Arab Emirates** | **GDPrGiniBase** | 0.013 | 0.013 | 0.014 | 0.007 | 0.002 |
| **United Arab Emirates** | **GDPrHGiniH** | 0.016 | 0.016 | 0.017 | 0.009 | 0.002 |
| **United Arab Emirates** | **GDPrHGiniHv** | 0.023 | 0.023 | 0.024 | 0.013 | 0.004 |
| **United Arab Emirates** | **GDPrHGiniLow** | 0.01 | 0.01 | 0.01 | 0.005 | 0.001 |
| **United Arab Emirates** | **GDPrLGiniH** | 0.016 | 0.016 | 0.019 | 0.011 | 0.003 |
| **United Arab Emirates** | **GDPrLGiniHv** | 0.023 | 0.023 | 0.027 | 0.016 | 0.005 |
| **United Arab Emirates** | **GDPrLGiniLow** | 0.01 | 0.01 | 0.012 | 0.006 | 0.002 |
| **United Arab Emirates** | **NoCOVIDginiB** | 0.009 | 0.009 | 0.009 | 0.006 | 0.002 |
| **United Kingdom** | **GDPrGiniBase** | 0.179 | 0.161 | 0.155 | 0.168 | 0.065 |
| **United Kingdom** | **GDPrHGiniH** | 0.224 | 0.202 | 0.189 | 0.191 | 0.074 |
| **United Kingdom** | **GDPrHGiniHv** | 0.304 | 0.277 | 0.26 | 0.263 | 0.108 |
| **United Kingdom** | **GDPrHGiniLow** | 0.142 | 0.127 | 0.118 | 0.119 | 0.042 |
| **United Kingdom** | **GDPrLGiniH** | 0.224 | 0.202 | 0.202 | 0.231 | 0.098 |
| **United Kingdom** | **GDPrLGiniHv** | 0.304 | 0.277 | 0.276 | 0.315 | 0.141 |
| **United Kingdom** | **GDPrLGiniLow** | 0.142 | 0.127 | 0.127 | 0.147 | 0.057 |
| **United Kingdom** | **NoCOVIDginiB** | 0.136 | 0.137 | 0.137 | 0.166 | 0.059 |
| **United States of America** | **GDPrGiniBase** | 3.366 | 3.183 | 3.082 | 2.888 | 1.803 |
| **United States of America** | **GDPrHGiniH** | 4.016 | 3.808 | 3.533 | 3.234 | 2.012 |
| **United States of America** | **GDPrHGiniHv** | 5.145 | 4.898 | 4.567 | 4.218 | 2.72 |
| **United States of America** | **GDPrHGiniLow** | 2.792 | 2.633 | 2.425 | 2.19 | 1.292 |
| **United States of America** | **GDPrLGiniH** | 4.016 | 3.808 | 3.832 | 3.747 | 2.474 |
| **United States of America** | **GDPrLGiniHv** | 5.145 | 4.898 | 4.929 | 4.841 | 3.3 |
| **United States of America** | **GDPrLGiniLow** | 2.792 | 2.633 | 2.65 | 2.573 | 1.621 |
| **United States of America** | **NoCOVIDginiB** | 3.02 | 3.044 | 3.1 | 3.012 | 1.887 |
| **Uruguay** | **GDPrGiniBase** | 0.006 | 0.006 | 0.006 | 0.003 | 0 |
| **Uruguay** | **GDPrHGiniH** | 0.008 | 0.008 | 0.007 | 0.004 | 0 |
| **Uruguay** | **GDPrHGiniHv** | 0.011 | 0.011 | 0.011 | 0.005 | 0.001 |
| **Uruguay** | **GDPrHGiniLow** | 0.004 | 0.004 | 0.004 | 0.002 | 0 |
| **Uruguay** | **GDPrLGiniH** | 0.008 | 0.008 | 0.008 | 0.004 | 0.001 |
| **Uruguay** | **GDPrLGiniHv** | 0.011 | 0.011 | 0.011 | 0.007 | 0.001 |
| **Uruguay** | **GDPrLGiniLow** | 0.004 | 0.004 | 0.005 | 0.003 | 0 |
| **Uruguay** | **NoCOVIDginiB** | 0.005 | 0.005 | 0.005 | 0.002 | 0 |
| **Uzbekistan** | **GDPrGiniBase** | 3.431 | 3.224 | 3.058 | 1.167 | 0.214 |
| **Uzbekistan** | **GDPrHGiniH** | 3.68 | 3.469 | 3.17 | 1.205 | 0.228 |
| **Uzbekistan** | **GDPrHGiniHv** | 4.061 | 3.845 | 3.535 | 1.431 | 0.302 |
| **Uzbekistan** | **GDPrHGiniLow** | 3.188 | 2.986 | 2.704 | 0.936 | 0.151 |
| **Uzbekistan** | **GDPrLGiniH** | 3.68 | 3.469 | 3.385 | 1.428 | 0.298 |
| **Uzbekistan** | **GDPrLGiniHv** | 4.061 | 3.845 | 3.76 | 1.68 | 0.388 |
| **Uzbekistan** | **GDPrLGiniLow** | 3.188 | 2.986 | 2.904 | 1.127 | 0.202 |
| **Uzbekistan** | **NoCOVIDginiB** | 3.093 | 2.877 | 2.67 | 0.971 | 0.172 |
| **Vanuatu** | **GDPrGiniBase** | 0.053 | 0.061 | 0.066 | 0.056 | 0.036 |
| **Vanuatu** | **GDPrHGiniH** | 0.056 | 0.064 | 0.067 | 0.053 | 0.035 |
| **Vanuatu** | **GDPrHGiniHv** | 0.06 | 0.068 | 0.072 | 0.058 | 0.04 |
| **Vanuatu** | **GDPrHGiniLow** | 0.05 | 0.058 | 0.062 | 0.047 | 0.029 |
| **Vanuatu** | **GDPrLGiniH** | 0.056 | 0.064 | 0.071 | 0.067 | 0.045 |
| **Vanuatu** | **GDPrLGiniHv** | 0.06 | 0.068 | 0.075 | 0.072 | 0.051 |
| **Vanuatu** | **GDPrLGiniLow** | 0.05 | 0.058 | 0.065 | 0.06 | 0.038 |
| **Vanuatu** | **NoCOVIDginiB** | 0.042 | 0.046 | 0.051 | 0.042 | 0.023 |
| **Venezuela, Bolivarian Republic** | **GDPrGiniBase** | 12.37 | 11.43 | 12.25 | 17.94 | 10.08 |
| **Venezuela, Bolivarian Republic** | **GDPrHGiniH** | 12.59 | 11.67 | 12.29 | 17.42 | 9.94 |
| **Venezuela, Bolivarian Republic** | **GDPrHGiniHv** | 12.91 | 12.01 | 12.62 | 17.76 | 10.59 |
| **Venezuela, Bolivarian Republic** | **GDPrHGiniLow** | 12.14 | 11.2 | 11.84 | 16.97 | 9.076 |
| **Venezuela, Bolivarian Republic** | **GDPrLGiniH** | 12.59 | 11.67 | 12.65 | 18.87 | 11.1 |
| **Venezuela, Bolivarian Republic** | **GDPrLGiniHv** | 12.91 | 12.01 | 12.97 | 19.15 | 11.74 |
| **Venezuela, Bolivarian Republic** | **GDPrLGiniLow** | 12.14 | 11.2 | 12.21 | 18.49 | 10.23 |
| **Venezuela, Bolivarian Republic** | **NoCOVIDginiB** | 10.69 | 9.868 | 8.905 | 13.52 | 5.507 |
| **Viet Nam** | **GDPrGiniBase** | 1.068 | 1.056 | 0.919 | 0.213 | 0.006 |
| **Viet Nam** | **GDPrHGiniH** | 1.256 | 1.244 | 1.028 | 0.238 | 0.008 |
| **Viet Nam** | **GDPrHGiniHv** | 1.577 | 1.564 | 1.308 | 0.332 | 0.013 |
| **Viet Nam** | **GDPrHGiniLow** | 0.899 | 0.889 | 0.722 | 0.145 | 0.003 |
| **Viet Nam** | **GDPrLGiniH** | 1.256 | 1.244 | 1.144 | 0.3 | 0.01 |
| **Viet Nam** | **GDPrLGiniHv** | 1.577 | 1.564 | 1.446 | 0.412 | 0.017 |
| **Viet Nam** | **GDPrLGiniLow** | 0.899 | 0.889 | 0.812 | 0.187 | 0.004 |
| **Viet Nam** | **NoCOVIDginiB** | 0.926 | 0.822 | 0.749 | 0.155 | 0.005 |
| **Yemen** | **GDPrGiniBase** | 16.75 | 17.96 | 18.51 | 15.56 | 6.445 |
| **Yemen** | **GDPrHGiniH** | 16.88 | 18.07 | 18.37 | 15.18 | 6.249 |
| **Yemen** | **GDPrHGiniHv** | 17.07 | 18.24 | 18.55 | 15.6 | 6.889 |
| **Yemen** | **GDPrHGiniLow** | 16.62 | 17.84 | 18.13 | 14.6 | 5.42 |
| **Yemen** | **GDPrLGiniH** | 16.88 | 18.07 | 18.85 | 16.41 | 7.556 |
| **Yemen** | **GDPrLGiniHv** | 17.07 | 18.24 | 19.01 | 16.8 | 8.238 |
| **Yemen** | **GDPrLGiniLow** | 16.62 | 17.84 | 18.63 | 15.88 | 6.664 |
| **Yemen** | **NoCOVIDginiB** | 14.77 | 13.27 | 12.58 | 9.178 | 2.243 |
| **Zambia** | **GDPrGiniBase** | 10.86 | 11.27 | 11.59 | 12.96 | 15.48 |
| **Zambia** | **GDPrHGiniH** | 11.01 | 11.42 | 11.68 | 12.95 | 15.58 |
| **Zambia** | **GDPrHGiniHv** | 11.24 | 11.65 | 11.92 | 13.31 | 16.35 |
| **Zambia** | **GDPrHGiniLow** | 10.7 | 11.11 | 11.36 | 12.45 | 14.54 |
| **Zambia** | **GDPrLGiniH** | 11.01 | 11.42 | 11.83 | 13.45 | 16.43 |
| **Zambia** | **GDPrLGiniHv** | 11.24 | 11.65 | 12.06 | 13.79 | 17.18 |
| **Zambia** | **GDPrLGiniLow** | 10.7 | 11.11 | 11.51 | 12.99 | 15.41 |
| **Zambia** | **NoCOVIDginiB** | 10.64 | 10.97 | 11.2 | 12.28 | 14.64 |
| **Zimbabwe** | **GDPrGiniBase** | 5.775 | 5.603 | 5.537 | 4.806 | 1.997 |
| **Zimbabwe** | **GDPrHGiniH** | 5.916 | 5.751 | 5.577 | 4.839 | 2.033 |
| **Zimbabwe** | **GDPrHGiniHv** | 6.125 | 5.968 | 5.803 | 5.126 | 2.336 |
| **Zimbabwe** | **GDPrHGiniLow** | 5.632 | 5.454 | 5.269 | 4.454 | 1.661 |
| **Zimbabwe** | **GDPrLGiniH** | 5.916 | 5.751 | 5.786 | 5.152 | 2.354 |
| **Zimbabwe** | **GDPrLGiniHv** | 6.125 | 5.968 | 6.008 | 5.436 | 2.673 |
| **Zimbabwe** | **GDPrLGiniLow** | 5.632 | 5.454 | 5.484 | 4.77 | 1.953 |
| **Zimbabwe** | **NoCOVIDginiB** | 5.07 | 5.121 | 5.144 | 4.478 | 1.757 |

**Table 6. Model results for the percent of the population living on less than $1.90 per day by scenario and selected years.**

| **Country** | **Scenario** | **2020** | **2021** | **2022** | **2030** | **2050** |
| --- | --- | --- | --- | --- | --- | --- |
| **Afghanistan** | **GDPrGiniBase** | 39.28 | 39.77 | 40.28 | 37.71 | 11.94 |
| **Afghanistan** | **GDPrHGiniH** | 39.89 | 40.38 | 39.42 | 35.92 | 10.92 |
| **Afghanistan** | **GDPrHGiniHv** | 40.79 | 41.27 | 40.33 | 36.94 | 12 |
| **Afghanistan** | **GDPrHGiniLow** | 38.65 | 39.15 | 38.16 | 34.52 | 9.504 |
| **Afghanistan** | **GDPrLGiniH** | 39.89 | 40.38 | 41.8 | 40.95 | 14.88 |
| **Afghanistan** | **GDPrLGiniHv** | 40.79 | 41.27 | 42.65 | 41.85 | 16.09 |
| **Afghanistan** | **GDPrLGiniLow** | 38.65 | 39.15 | 40.62 | 39.71 | 13.27 |
| **Afghanistan** | **NoCOVIDginiB** | 34.86 | 35.25 | 35.6 | 33.1 | 9.065 |
| **Albania** | **GDPrGiniBase** | 1.304 | 1.228 | 1.148 | 0.752 | 0.113 |
| **Albania** | **GDPrHGiniH** | 1.516 | 1.432 | 1.263 | 0.811 | 0.126 |
| **Albania** | **GDPrHGiniHv** | 1.874 | 1.777 | 1.577 | 1.039 | 0.181 |
| **Albania** | **GDPrHGiniLow** | 1.112 | 1.044 | 0.912 | 0.563 | 0.074 |
| **Albania** | **GDPrLGiniH** | 1.516 | 1.432 | 1.416 | 0.98 | 0.166 |
| **Albania** | **GDPrLGiniHv** | 1.874 | 1.777 | 1.757 | 1.242 | 0.235 |
| **Albania** | **GDPrLGiniLow** | 1.112 | 1.044 | 1.033 | 0.693 | 0.099 |
| **Albania** | **NoCOVIDginiB** | 0.917 | 0.921 | 0.92 | 0.615 | 0.08 |
| **Algeria** | **GDPrGiniBase** | 0.326 | 0.321 | 0.36 | 0.737 | 0.069 |
| **Algeria** | **GDPrHGiniH** | 0.397 | 0.392 | 0.421 | 0.79 | 0.075 |
| **Algeria** | **GDPrHGiniHv** | 0.524 | 0.517 | 0.553 | 1.002 | 0.109 |
| **Algeria** | **GDPrHGiniLow** | 0.265 | 0.261 | 0.282 | 0.558 | 0.043 |
| **Algeria** | **GDPrLGiniH** | 0.397 | 0.392 | 0.454 | 0.964 | 0.106 |
| **Algeria** | **GDPrLGiniHv** | 0.524 | 0.517 | 0.595 | 1.208 | 0.151 |
| **Algeria** | **GDPrLGiniLow** | 0.265 | 0.261 | 0.306 | 0.693 | 0.063 |
| **Algeria** | **NoCOVIDginiB** | 0.265 | 0.236 | 0.232 | 0.675 | 0.049 |
| **Angola** | **GDPrGiniBase** | 51.89 | 53.38 | 53.74 | 47.8 | 36.92 |
| **Angola** | **GDPrHGiniH** | 52.76 | 54.22 | 54.16 | 47.98 | 36.94 |
| **Angola** | **GDPrHGiniHv** | 54.05 | 55.45 | 55.4 | 49.45 | 38.76 |
| **Angola** | **GDPrHGiniLow** | 51 | 52.53 | 52.47 | 45.98 | 34.47 |
| **Angola** | **GDPrLGiniH** | 52.76 | 54.22 | 54.98 | 49.66 | 39.41 |
| **Angola** | **GDPrLGiniHv** | 54.05 | 55.45 | 56.19 | 51.07 | 41.18 |
| **Angola** | **GDPrLGiniLow** | 51 | 52.53 | 53.33 | 47.74 | 37.02 |
| **Angola** | **NoCOVIDginiB** | 49.96 | 50.22 | 49.88 | 43.98 | 31.82 |
| **Argentina** | **GDPrGiniBase** | 5.519 | 5.176 | 5 | 3.895 | 2.145 |
| **Argentina** | **GDPrHGiniH** | 6.118 | 5.753 | 5.465 | 4.09 | 2.334 |
| **Argentina** | **GDPrHGiniHv** | 7.072 | 6.677 | 6.364 | 4.852 | 2.871 |
| **Argentina** | **GDPrHGiniLow** | 4.951 | 4.63 | 4.376 | 3.187 | 1.724 |
| **Argentina** | **GDPrLGiniH** | 6.118 | 5.753 | 5.671 | 4.705 | 2.634 |
| **Argentina** | **GDPrLGiniHv** | 7.072 | 6.677 | 6.588 | 5.533 | 3.215 |
| **Argentina** | **GDPrLGiniLow** | 4.951 | 4.63 | 4.556 | 3.714 | 1.968 |
| **Argentina** | **NoCOVIDginiB** | 4.901 | 5.042 | 4.843 | 3.867 | 2.082 |
| **Armenia** | **GDPrGiniBase** | 1.041 | 0.945 | 0.927 | 0.466 | 0.067 |
| **Armenia** | **GDPrHGiniH** | 1.224 | 1.117 | 1.031 | 0.514 | 0.078 |
| **Armenia** | **GDPrHGiniHv** | 1.537 | 1.411 | 1.307 | 0.68 | 0.116 |
| **Armenia** | **GDPrHGiniLow** | 0.877 | 0.793 | 0.728 | 0.342 | 0.043 |
| **Armenia** | **GDPrLGiniH** | 1.224 | 1.117 | 1.155 | 0.62 | 0.1 |
| **Armenia** | **GDPrLGiniHv** | 1.537 | 1.411 | 1.455 | 0.811 | 0.146 |
| **Armenia** | **GDPrLGiniLow** | 0.877 | 0.793 | 0.824 | 0.419 | 0.057 |
| **Armenia** | **NoCOVIDginiB** | 0.616 | 0.617 | 0.624 | 0.326 | 0.04 |
| **Australia** | **GDPrGiniBase** | 0.463 | 0.429 | 0.374 | 0.255 | 0.053 |
| **Australia** | **GDPrHGiniH** | 0.563 | 0.524 | 0.432 | 0.287 | 0.062 |
| **Australia** | **GDPrHGiniHv** | 0.741 | 0.692 | 0.578 | 0.394 | 0.094 |
| **Australia** | **GDPrHGiniLow** | 0.376 | 0.347 | 0.282 | 0.18 | 0.034 |
| **Australia** | **GDPrLGiniH** | 0.563 | 0.524 | 0.487 | 0.356 | 0.079 |
| **Australia** | **GDPrLGiniHv** | 0.741 | 0.692 | 0.647 | 0.482 | 0.117 |
| **Australia** | **GDPrLGiniLow** | 0.376 | 0.347 | 0.321 | 0.228 | 0.044 |
| **Australia** | **NoCOVIDginiB** | 0.439 | 0.419 | 0.393 | 0.263 | 0.055 |
| **Austria** | **GDPrGiniBase** | 0.332 | 0.362 | 0.395 | 0.203 | 0.054 |
| **Austria** | **GDPrHGiniH** | 0.406 | 0.441 | 0.469 | 0.22 | 0.059 |
| **Austria** | **GDPrHGiniHv** | 0.538 | 0.582 | 0.616 | 0.302 | 0.087 |
| **Austria** | **GDPrHGiniLow** | 0.269 | 0.294 | 0.314 | 0.138 | 0.033 |
| **Austria** | **GDPrLGiniH** | 0.406 | 0.441 | 0.489 | 0.293 | 0.086 |
| **Austria** | **GDPrLGiniHv** | 0.538 | 0.582 | 0.641 | 0.395 | 0.125 |
| **Austria** | **GDPrLGiniLow** | 0.269 | 0.294 | 0.329 | 0.188 | 0.05 |
| **Austria** | **NoCOVIDginiB** | 0.292 | 0.289 | 0.28 | 0.179 | 0.048 |
| **Azerbaijan** | **GDPrGiniBase** | 0.111 | 0.091 | 0.064 | 0.003 | 0 |
| **Azerbaijan** | **GDPrHGiniH** | 0.141 | 0.117 | 0.079 | 0.003 | 0 |
| **Azerbaijan** | **GDPrHGiniHv** | 0.197 | 0.165 | 0.114 | 0.006 | 0.001 |
| **Azerbaijan** | **GDPrHGiniLow** | 0.086 | 0.07 | 0.046 | 0.001 | 0 |
| **Azerbaijan** | **GDPrLGiniH** | 0.141 | 0.117 | 0.088 | 0.005 | 0 |
| **Azerbaijan** | **GDPrLGiniHv** | 0.197 | 0.165 | 0.127 | 0.008 | 0.001 |
| **Azerbaijan** | **GDPrLGiniLow** | 0.086 | 0.07 | 0.052 | 0.002 | 0 |
| **Azerbaijan** | **NoCOVIDginiB** | 0.086 | 0.068 | 0.049 | 0.002 | 0 |
| **Bahamas** | **GDPrGiniBase** | 0.2 | 0.231 | 0.22 | 0.179 | 0.219 |
| **Bahamas** | **GDPrHGiniH** | 0.256 | 0.293 | 0.266 | 0.209 | 0.252 |
| **Bahamas** | **GDPrHGiniHv** | 0.36 | 0.409 | 0.373 | 0.299 | 0.356 |
| **Bahamas** | **GDPrHGiniLow** | 0.155 | 0.18 | 0.161 | 0.124 | 0.151 |
| **Bahamas** | **GDPrLGiniH** | 0.256 | 0.293 | 0.294 | 0.254 | 0.308 |
| **Bahamas** | **GDPrLGiniHv** | 0.36 | 0.409 | 0.41 | 0.358 | 0.43 |
| **Bahamas** | **GDPrLGiniLow** | 0.155 | 0.18 | 0.181 | 0.153 | 0.189 |
| **Bahamas** | **NoCOVIDginiB** | 0.112 | 0.124 | 0.136 | 0.153 | 0.172 |
| **Bahrain** | **GDPrGiniBase** | 0.19 | 0.227 | 0.264 | 0.082 | 0.012 |
| **Bahrain** | **GDPrHGiniH** | 0.242 | 0.286 | 0.317 | 0.099 | 0.015 |
| **Bahrain** | **GDPrHGiniHv** | 0.338 | 0.395 | 0.436 | 0.146 | 0.025 |
| **Bahrain** | **GDPrHGiniLow** | 0.148 | 0.178 | 0.199 | 0.056 | 0.007 |
| **Bahrain** | **GDPrLGiniH** | 0.242 | 0.286 | 0.341 | 0.116 | 0.019 |
| **Bahrain** | **GDPrLGiniHv** | 0.338 | 0.395 | 0.467 | 0.17 | 0.032 |
| **Bahrain** | **GDPrLGiniLow** | 0.148 | 0.178 | 0.216 | 0.066 | 0.009 |
| **Bahrain** | **NoCOVIDginiB** | 0.153 | 0.174 | 0.178 | 0.058 | 0.01 |
| **Bangladesh** | **GDPrGiniBase** | 7.846 | 7.515 | 6.916 | 2.918 | 0.243 |
| **Bangladesh** | **GDPrHGiniH** | 8.478 | 8.135 | 7.192 | 3.032 | 0.261 |
| **Bangladesh** | **GDPrHGiniHv** | 9.455 | 9.096 | 8.096 | 3.59 | 0.359 |
| **Bangladesh** | **GDPrHGiniLow** | 7.232 | 6.913 | 6.053 | 2.367 | 0.163 |
| **Bangladesh** | **GDPrLGiniH** | 8.478 | 8.135 | 7.807 | 3.53 | 0.347 |
| **Bangladesh** | **GDPrLGiniHv** | 9.455 | 9.096 | 8.747 | 4.142 | 0.469 |
| **Bangladesh** | **GDPrLGiniLow** | 7.232 | 6.913 | 6.616 | 2.793 | 0.223 |
| **Bangladesh** | **NoCOVIDginiB** | 6.983 | 6.167 | 5.515 | 2.334 | 0.161 |
| **Barbados** | **GDPrGiniBase** | 8.771 | 8.087 | 6.781 | 4.522 | 1.709 |
| **Barbados** | **GDPrHGiniH** | 9.479 | 8.768 | 7.168 | 4.738 | 1.814 |
| **Barbados** | **GDPrHGiniHv** | 10.58 | 9.828 | 8.132 | 5.515 | 2.241 |
| **Barbados** | **GDPrHGiniLow** | 8.083 | 7.426 | 5.964 | 3.797 | 1.33 |
| **Barbados** | **GDPrLGiniH** | 9.479 | 8.768 | 7.653 | 5.324 | 2.159 |
| **Barbados** | **GDPrLGiniHv** | 10.58 | 9.828 | 8.647 | 6.152 | 2.638 |
| **Barbados** | **GDPrLGiniLow** | 8.083 | 7.426 | 6.404 | 4.31 | 1.609 |
| **Barbados** | **NoCOVIDginiB** | 5.101 | 4.965 | 4.724 | 3.649 | 1.485 |
| **Belarus** | **GDPrGiniBase** | 0.093 | 0.091 | 0.089 | 0.026 | 0.002 |
| **Belarus** | **GDPrHGiniH** | 0.119 | 0.116 | 0.108 | 0.029 | 0.002 |
| **Belarus** | **GDPrHGiniHv** | 0.167 | 0.163 | 0.153 | 0.045 | 0.004 |
| **Belarus** | **GDPrHGiniLow** | 0.072 | 0.07 | 0.065 | 0.016 | 0.001 |
| **Belarus** | **GDPrLGiniH** | 0.119 | 0.116 | 0.119 | 0.041 | 0.004 |
| **Belarus** | **GDPrLGiniHv** | 0.167 | 0.163 | 0.167 | 0.061 | 0.007 |
| **Belarus** | **GDPrLGiniLow** | 0.072 | 0.07 | 0.072 | 0.023 | 0.002 |
| **Belarus** | **NoCOVIDginiB** | 0.09 | 0.096 | 0.101 | 0.03 | 0.002 |
| **Belgium** | **GDPrGiniBase** | 0.144 | 0.167 | 0.175 | 0.051 | 0.007 |
| **Belgium** | **GDPrHGiniH** | 0.181 | 0.209 | 0.213 | 0.055 | 0.009 |
| **Belgium** | **GDPrHGiniHv** | 0.25 | 0.286 | 0.291 | 0.082 | 0.014 |
| **Belgium** | **GDPrHGiniLow** | 0.113 | 0.132 | 0.134 | 0.031 | 0.004 |
| **Belgium** | **GDPrLGiniH** | 0.181 | 0.209 | 0.224 | 0.08 | 0.013 |
| **Belgium** | **GDPrLGiniHv** | 0.25 | 0.286 | 0.306 | 0.116 | 0.021 |
| **Belgium** | **GDPrLGiniLow** | 0.113 | 0.132 | 0.142 | 0.047 | 0.006 |
| **Belgium** | **NoCOVIDginiB** | 0.126 | 0.143 | 0.143 | 0.05 | 0.007 |
| **Belize** | **GDPrGiniBase** | 17.56 | 17.46 | 17.64 | 14.67 | 9.525 |
| **Belize** | **GDPrHGiniH** | 18.78 | 18.69 | 18.53 | 15.27 | 9.937 |
| **Belize** | **GDPrHGiniHv** | 20.66 | 20.57 | 20.4 | 17.05 | 11.47 |
| **Belize** | **GDPrHGiniLow** | 16.36 | 16.26 | 16.12 | 13.01 | 8.063 |
| **Belize** | **GDPrLGiniH** | 18.78 | 18.69 | 19.21 | 16.45 | 11.1 |
| **Belize** | **GDPrLGiniHv** | 20.66 | 20.57 | 21.1 | 18.27 | 12.72 |
| **Belize** | **GDPrLGiniLow** | 16.36 | 16.26 | 16.78 | 14.13 | 9.107 |
| **Belize** | **NoCOVIDginiB** | 13.91 | 14.68 | 15.37 | 13.61 | 7.483 |
| **Benin** | **GDPrGiniBase** | 45.07 | 44.37 | 43.12 | 36.23 | 12.4 |
| **Benin** | **GDPrHGiniH** | 46 | 45.32 | 43.5 | 36.41 | 12.65 |
| **Benin** | **GDPrHGiniHv** | 47.37 | 46.72 | 44.95 | 38.01 | 14.23 |
| **Benin** | **GDPrHGiniLow** | 44.12 | 43.41 | 41.53 | 34.25 | 10.67 |
| **Benin** | **GDPrLGiniH** | 46 | 45.32 | 44.69 | 38.23 | 14.34 |
| **Benin** | **GDPrLGiniHv** | 47.37 | 46.72 | 46.1 | 39.8 | 15.99 |
| **Benin** | **GDPrLGiniLow** | 44.12 | 43.41 | 42.76 | 36.1 | 12.23 |
| **Benin** | **NoCOVIDginiB** | 43.55 | 42.57 | 41.73 | 34.83 | 11.32 |
| **Bhutan** | **GDPrGiniBase** | 1.912 | 2.61 | 3.264 | 4.084 | 1.079 |
| **Bhutan** | **GDPrHGiniH** | 2.203 | 2.971 | 3.487 | 4.206 | 1.146 |
| **Bhutan** | **GDPrHGiniHv** | 2.689 | 3.563 | 4.137 | 4.937 | 1.455 |
| **Bhutan** | **GDPrHGiniLow** | 1.646 | 2.276 | 2.716 | 3.328 | 0.807 |
| **Bhutan** | **GDPrLGiniH** | 2.203 | 2.971 | 3.884 | 4.955 | 1.421 |
| **Bhutan** | **GDPrLGiniHv** | 2.689 | 3.563 | 4.578 | 5.758 | 1.781 |
| **Bhutan** | **GDPrLGiniLow** | 1.646 | 2.276 | 3.055 | 3.978 | 1.021 |
| **Bhutan** | **NoCOVIDginiB** | 1.035 | 1.012 | 1.052 | 1.685 | 0.296 |
| **Bolivia** | **GDPrGiniBase** | 5.322 | 5.385 | 5.483 | 3.772 | 0.907 |
| **Bolivia** | **GDPrHGiniH** | 5.916 | 5.984 | 5.927 | 3.988 | 1.007 |
| **Bolivia** | **GDPrHGiniHv** | 6.865 | 6.94 | 6.877 | 4.745 | 1.312 |
| **Bolivia** | **GDPrHGiniLow** | 4.761 | 4.819 | 4.771 | 3.093 | 0.683 |
| **Bolivia** | **GDPrLGiniH** | 5.916 | 5.984 | 6.251 | 4.54 | 1.184 |
| **Bolivia** | **GDPrLGiniHv** | 6.865 | 6.94 | 7.228 | 5.355 | 1.526 |
| **Bolivia** | **GDPrLGiniLow** | 4.761 | 4.819 | 5.057 | 3.565 | 0.815 |
| **Bolivia** | **NoCOVIDginiB** | 4.029 | 3.984 | 3.929 | 2.848 | 0.632 |
| **Bosnia and Herzegovina** | **GDPrGiniBase** | 0.159 | 0.194 | 0.248 | 0.418 | 0.048 |
| **Bosnia and Herzegovina** | **GDPrHGiniH** | 0.201 | 0.244 | 0.285 | 0.446 | 0.056 |
| **Bosnia and Herzegovina** | **GDPrHGiniHv** | 0.28 | 0.337 | 0.389 | 0.591 | 0.084 |
| **Bosnia and Herzegovina** | **GDPrHGiniLow** | 0.124 | 0.153 | 0.181 | 0.294 | 0.031 |
| **Bosnia and Herzegovina** | **GDPrLGiniH** | 0.201 | 0.244 | 0.331 | 0.585 | 0.071 |
| **Bosnia and Herzegovina** | **GDPrLGiniHv** | 0.28 | 0.337 | 0.448 | 0.762 | 0.105 |
| **Bosnia and Herzegovina** | **GDPrLGiniLow** | 0.124 | 0.153 | 0.213 | 0.396 | 0.039 |
| **Bosnia and Herzegovina** | **NoCOVIDginiB** | 0.113 | 0.145 | 0.2 | 0.337 | 0.037 |
| **Botswana** | **GDPrGiniBase** | 15.78 | 15.39 | 16.11 | 12.77 | 6.581 |
| **Botswana** | **GDPrHGiniH** | 16.97 | 16.57 | 16.96 | 13.38 | 7.065 |
| **Botswana** | **GDPrHGiniHv** | 18.8 | 18.39 | 18.79 | 15.08 | 8.347 |
| **Botswana** | **GDPrHGiniLow** | 14.63 | 14.25 | 14.62 | 11.25 | 5.539 |
| **Botswana** | **GDPrLGiniH** | 16.97 | 16.57 | 17.67 | 14.39 | 7.73 |
| **Botswana** | **GDPrLGiniHv** | 18.8 | 18.39 | 19.52 | 16.14 | 9.075 |
| **Botswana** | **GDPrLGiniLow** | 14.63 | 14.25 | 15.29 | 12.19 | 6.119 |
| **Botswana** | **NoCOVIDginiB** | 13.03 | 12.98 | 13.32 | 11.29 | 5.83 |
| **Brazil** | **GDPrGiniBase** | 4.88 | 4.673 | 4.647 | 3.588 | 1.786 |
| **Brazil** | **GDPrHGiniH** | 5.563 | 5.34 | 5.231 | 3.958 | 2.059 |
| **Brazil** | **GDPrHGiniHv** | 6.691 | 6.441 | 6.321 | 4.886 | 2.669 |
| **Brazil** | **GDPrHGiniLow** | 4.25 | 4.061 | 3.968 | 2.909 | 1.408 |
| **Brazil** | **GDPrLGiniH** | 5.563 | 5.34 | 5.394 | 4.372 | 2.231 |
| **Brazil** | **GDPrLGiniHv** | 6.691 | 6.441 | 6.503 | 5.357 | 2.875 |
| **Brazil** | **GDPrLGiniLow** | 4.25 | 4.061 | 4.105 | 3.248 | 1.539 |
| **Brazil** | **NoCOVIDginiB** | 4.566 | 4.461 | 4.322 | 3.205 | 1.592 |
| **Brunei Darussalam** | **GDPrGiniBase** | 0.068 | 0.062 | 0.05 | 0.008 | 0.003 |
| **Brunei Darussalam** | **GDPrHGiniH** | 0.091 | 0.084 | 0.064 | 0.01 | 0.003 |
| **Brunei Darussalam** | **GDPrHGiniHv** | 0.139 | 0.129 | 0.1 | 0.018 | 0.006 |
| **Brunei Darussalam** | **GDPrHGiniLow** | 0.049 | 0.045 | 0.033 | 0.004 | 0.001 |
| **Brunei Darussalam** | **GDPrLGiniH** | 0.091 | 0.084 | 0.072 | 0.013 | 0.005 |
| **Brunei Darussalam** | **GDPrLGiniHv** | 0.139 | 0.129 | 0.112 | 0.023 | 0.009 |
| **Brunei Darussalam** | **GDPrLGiniLow** | 0.049 | 0.045 | 0.038 | 0.006 | 0.002 |
| **Brunei Darussalam** | **NoCOVIDginiB** | 0.059 | 0.049 | 0.041 | 0.006 | 0.002 |
| **Bulgaria** | **GDPrGiniBase** | 1.189 | 1.148 | 1.069 | 0.503 | 0.231 |
| **Bulgaria** | **GDPrHGiniH** | 1.406 | 1.36 | 1.216 | 0.568 | 0.27 |
| **Bulgaria** | **GDPrHGiniHv** | 1.78 | 1.725 | 1.553 | 0.762 | 0.38 |
| **Bulgaria** | **GDPrHGiniLow** | 0.996 | 0.96 | 0.849 | 0.369 | 0.164 |
| **Bulgaria** | **GDPrLGiniH** | 1.406 | 1.36 | 1.305 | 0.672 | 0.319 |
| **Bulgaria** | **GDPrLGiniHv** | 1.78 | 1.725 | 1.659 | 0.891 | 0.444 |
| **Bulgaria** | **GDPrLGiniLow** | 0.996 | 0.96 | 0.917 | 0.443 | 0.196 |
| **Bulgaria** | **NoCOVIDginiB** | 1.012 | 0.97 | 0.941 | 0.468 | 0.216 |
| **Burkina Faso** | **GDPrGiniBase** | 35.32 | 33.91 | 32.07 | 19.93 | 3.177 |
| **Burkina Faso** | **GDPrHGiniH** | 36.13 | 34.75 | 32.03 | 19.66 | 3.23 |
| **Burkina Faso** | **GDPrHGiniHv** | 37.32 | 35.99 | 33.29 | 20.95 | 3.89 |
| **Burkina Faso** | **GDPrHGiniLow** | 34.49 | 33.06 | 30.33 | 17.94 | 2.459 |
| **Burkina Faso** | **GDPrLGiniH** | 36.13 | 34.75 | 33.82 | 22.05 | 4.04 |
| **Burkina Faso** | **GDPrLGiniHv** | 37.32 | 35.99 | 35.05 | 23.36 | 4.791 |
| **Burkina Faso** | **GDPrLGiniLow** | 34.49 | 33.06 | 32.15 | 20.3 | 3.138 |
| **Burkina Faso** | **NoCOVIDginiB** | 32.51 | 31.21 | 29.96 | 18.16 | 2.688 |
| **Burundi** | **GDPrGiniBase** | 79.89 | 80.05 | 79.43 | 74.08 | 58.1 |
| **Burundi** | **GDPrHGiniH** | 79.82 | 79.98 | 78.86 | 72.63 | 55.88 |
| **Burundi** | **GDPrHGiniHv** | 79.74 | 79.89 | 78.81 | 72.77 | 56.7 |
| **Burundi** | **GDPrHGiniLow** | 79.97 | 80.13 | 78.98 | 72.48 | 54.74 |
| **Burundi** | **GDPrLGiniH** | 79.82 | 79.98 | 79.84 | 75.59 | 61.85 |
| **Burundi** | **GDPrLGiniHv** | 79.74 | 79.89 | 79.75 | 75.62 | 62.4 |
| **Burundi** | **GDPrLGiniLow** | 79.97 | 80.13 | 79.99 | 75.58 | 61.08 |
| **Burundi** | **NoCOVIDginiB** | 79.83 | 80.51 | 81.1 | 77.4 | 62.95 |
| **Cabo Verde** | **GDPrGiniBase** | 3.814 | 4.245 | 4.429 | 2.219 | 0.779 |
| **Cabo Verde** | **GDPrHGiniH** | 4.303 | 4.773 | 4.711 | 2.302 | 0.873 |
| **Cabo Verde** | **GDPrHGiniHv** | 5.098 | 5.626 | 5.543 | 2.831 | 1.151 |
| **Cabo Verde** | **GDPrHGiniLow** | 3.359 | 3.752 | 3.716 | 1.7 | 0.579 |
| **Cabo Verde** | **GDPrLGiniH** | 4.303 | 4.773 | 5.187 | 2.836 | 1.04 |
| **Cabo Verde** | **GDPrLGiniHv** | 5.098 | 5.626 | 6.066 | 3.44 | 1.357 |
| **Cabo Verde** | **GDPrLGiniLow** | 3.359 | 3.752 | 4.129 | 2.139 | 0.703 |
| **Cabo Verde** | **NoCOVIDginiB** | 2.029 | 2.042 | 2.093 | 1.2 | 0.454 |
| **Cambodia** | **GDPrGiniBase** | 22.45 | 23.47 | 22.55 | 10.6 | 0.86 |
| **Cambodia** | **GDPrHGiniH** | 23.28 | 24.3 | 22.64 | 10.61 | 0.886 |
| **Cambodia** | **GDPrHGiniHv** | 24.5 | 25.52 | 23.86 | 11.66 | 1.127 |
| **Cambodia** | **GDPrHGiniLow** | 21.61 | 22.63 | 20.98 | 9.252 | 0.622 |
| **Cambodia** | **GDPrLGiniH** | 23.28 | 24.3 | 24.14 | 12.05 | 1.159 |
| **Cambodia** | **GDPrLGiniHv** | 24.5 | 25.52 | 25.36 | 13.14 | 1.452 |
| **Cambodia** | **GDPrLGiniLow** | 21.61 | 22.63 | 22.48 | 10.61 | 0.833 |
| **Cambodia** | **NoCOVIDginiB** | 18.06 | 16.99 | 16.12 | 7.137 | 0.349 |
| **Cameroon** | **GDPrGiniBase** | 23.76 | 23.58 | 22.87 | 20.03 | 13.89 |
| **Cameroon** | **GDPrHGiniH** | 24.89 | 24.71 | 23.53 | 20.51 | 13.89 |
| **Cameroon** | **GDPrHGiniHv** | 26.57 | 26.4 | 25.21 | 22.19 | 15.45 |
| **Cameroon** | **GDPrHGiniLow** | 22.64 | 22.46 | 21.29 | 18.31 | 11.88 |
| **Cameroon** | **GDPrLGiniH** | 24.89 | 24.71 | 24.47 | 21.8 | 16.14 |
| **Cameroon** | **GDPrLGiniHv** | 26.57 | 26.4 | 26.15 | 23.49 | 17.79 |
| **Cameroon** | **GDPrLGiniLow** | 22.64 | 22.46 | 22.22 | 19.58 | 14.02 |
| **Cameroon** | **NoCOVIDginiB** | 22.62 | 22.11 | 21.44 | 18.88 | 12.11 |
| **Canada** | **GDPrGiniBase** | 0.269 | 0.264 | 0.256 | 0.2 | 0.102 |
| **Canada** | **GDPrHGiniH** | 0.334 | 0.329 | 0.308 | 0.228 | 0.116 |
| **Canada** | **GDPrHGiniHv** | 0.453 | 0.446 | 0.419 | 0.317 | 0.167 |
| **Canada** | **GDPrHGiniLow** | 0.214 | 0.21 | 0.195 | 0.141 | 0.067 |
| **Canada** | **GDPrLGiniH** | 0.334 | 0.329 | 0.329 | 0.277 | 0.151 |
| **Canada** | **GDPrLGiniHv** | 0.453 | 0.446 | 0.447 | 0.38 | 0.215 |
| **Canada** | **GDPrLGiniLow** | 0.214 | 0.21 | 0.21 | 0.174 | 0.09 |
| **Canada** | **NoCOVIDginiB** | 0.228 | 0.225 | 0.224 | 0.199 | 0.1 |
| **Central African Republic** | **GDPrGiniBase** | 72.08 | 73.52 | 73.84 | 74.4 | 67.42 |
| **Central African Republic** | **GDPrHGiniH** | 72.51 | 73.9 | 73.76 | 73.02 | 66.26 |
| **Central African Republic** | **GDPrHGiniHv** | 73.15 | 74.48 | 74.34 | 73.64 | 67.3 |
| **Central African Republic** | **GDPrHGiniLow** | 71.66 | 73.14 | 73 | 72.22 | 64.85 |
| **Central African Republic** | **GDPrLGiniH** | 72.51 | 73.9 | 74.53 | 76.36 | 68.21 |
| **Central African Republic** | **GDPrLGiniHv** | 73.15 | 74.48 | 75.08 | 76.82 | 69.13 |
| **Central African Republic** | **GDPrLGiniLow** | 71.66 | 73.14 | 73.83 | 75.78 | 67.6 |
| **Central African Republic** | **NoCOVIDginiB** | 70.49 | 70.14 | 70.11 | 69.49 | 60.81 |
| **Chad** | **GDPrGiniBase** | 42.46 | 43.61 | 43.95 | 36.9 | 18.09 |
| **Chad** | **GDPrHGiniH** | 43.32 | 44.46 | 44.07 | 36.89 | 17.87 |
| **Chad** | **GDPrHGiniHv** | 44.6 | 45.71 | 45.33 | 38.33 | 19.49 |
| **Chad** | **GDPrHGiniLow** | 41.57 | 42.75 | 42.35 | 34.93 | 15.76 |
| **Chad** | **GDPrLGiniH** | 43.32 | 44.46 | 45.49 | 39.03 | 20.54 |
| **Chad** | **GDPrLGiniHv** | 44.6 | 45.71 | 46.71 | 40.42 | 22.2 |
| **Chad** | **GDPrLGiniLow** | 41.57 | 42.75 | 43.83 | 37.12 | 18.34 |
| **Chad** | **NoCOVIDginiB** | 39.61 | 39.09 | 38.51 | 31.97 | 12.17 |
| **Chile** | **GDPrGiniBase** | 0.348 | 0.302 | 0.292 | 0.213 | 0.102 |
| **Chile** | **GDPrHGiniH** | 0.439 | 0.383 | 0.354 | 0.256 | 0.126 |
| **Chile** | **GDPrHGiniHv** | 0.608 | 0.535 | 0.497 | 0.367 | 0.19 |
| **Chile** | **GDPrHGiniLow** | 0.272 | 0.234 | 0.215 | 0.151 | 0.069 |
| **Chile** | **GDPrLGiniH** | 0.439 | 0.383 | 0.388 | 0.295 | 0.147 |
| **Chile** | **GDPrLGiniHv** | 0.608 | 0.535 | 0.542 | 0.419 | 0.219 |
| **Chile** | **GDPrLGiniLow** | 0.272 | 0.234 | 0.238 | 0.176 | 0.082 |
| **Chile** | **NoCOVIDginiB** | 0.281 | 0.277 | 0.265 | 0.195 | 0.095 |
| **China** | **GDPrGiniBase** | 0.201 | 0.152 | 0.129 | 0.023 | 0.001 |
| **China** | **GDPrHGiniH** | 0.256 | 0.196 | 0.157 | 0.029 | 0.001 |
| **China** | **GDPrHGiniHv** | 0.359 | 0.279 | 0.227 | 0.046 | 0.003 |
| **China** | **GDPrHGiniLow** | 0.156 | 0.116 | 0.092 | 0.014 | 0.001 |
| **China** | **GDPrLGiniH** | 0.256 | 0.196 | 0.178 | 0.036 | 0.002 |
| **China** | **GDPrLGiniHv** | 0.359 | 0.279 | 0.255 | 0.057 | 0.004 |
| **China** | **GDPrLGiniLow** | 0.156 | 0.116 | 0.105 | 0.018 | 0.001 |
| **China** | **NoCOVIDginiB** | 0.18 | 0.142 | 0.116 | 0.019 | 0.001 |
| **Colombia** | **GDPrGiniBase** | 5.047 | 4.716 | 4.528 | 3.778 | 4.509 |
| **Colombia** | **GDPrHGiniH** | 5.703 | 5.347 | 5.05 | 4.116 | 4.905 |
| **Colombia** | **GDPrHGiniHv** | 6.772 | 6.379 | 6.049 | 5.007 | 5.904 |
| **Colombia** | **GDPrHGiniLow** | 4.437 | 4.132 | 3.879 | 3.09 | 3.742 |
| **Colombia** | **GDPrLGiniH** | 5.703 | 5.347 | 5.24 | 4.557 | 5.342 |
| **Colombia** | **GDPrLGiniHv** | 6.772 | 6.379 | 6.261 | 5.504 | 6.388 |
| **Colombia** | **GDPrLGiniLow** | 4.437 | 4.132 | 4.04 | 3.458 | 4.115 |
| **Colombia** | **NoCOVIDginiB** | 4.381 | 4.225 | 4.024 | 3.376 | 4.048 |
| **Comoros** | **GDPrGiniBase** | 19.33 | 19.59 | 19.12 | 16.68 | 8.149 |
| **Comoros** | **GDPrHGiniH** | 20.39 | 20.66 | 19.68 | 17.1 | 8.289 |
| **Comoros** | **GDPrHGiniHv** | 22.01 | 22.28 | 21.28 | 18.67 | 9.512 |
| **Comoros** | **GDPrHGiniLow** | 18.27 | 18.53 | 17.56 | 15.05 | 6.779 |
| **Comoros** | **GDPrLGiniH** | 20.39 | 20.66 | 20.65 | 18.3 | 9.621 |
| **Comoros** | **GDPrLGiniHv** | 22.01 | 22.28 | 22.27 | 19.89 | 10.93 |
| **Comoros** | **GDPrLGiniLow** | 18.27 | 18.53 | 18.52 | 16.22 | 7.99 |
| **Comoros** | **NoCOVIDginiB** | 17.9 | 17.65 | 17.54 | 15.54 | 6.796 |
| **Congo** | **GDPrGiniBase** | 49.31 | 50.4 | 51.61 | 39.75 | 11.72 |
| **Congo** | **GDPrHGiniH** | 50.18 | 51.24 | 51.8 | 39.58 | 12.1 |
| **Congo** | **GDPrHGiniHv** | 51.46 | 52.49 | 53.02 | 41.17 | 13.62 |
| **Congo** | **GDPrHGiniLow** | 48.43 | 49.54 | 50.13 | 37.42 | 10.19 |
| **Congo** | **GDPrLGiniH** | 50.18 | 51.24 | 53.02 | 42.16 | 13.26 |
| **Congo** | **GDPrLGiniHv** | 51.46 | 52.49 | 54.2 | 43.68 | 14.83 |
| **Congo** | **GDPrLGiniLow** | 48.43 | 49.54 | 51.41 | 40.09 | 11.26 |
| **Congo** | **NoCOVIDginiB** | 44.73 | 44.96 | 47.36 | 36 | 10.1 |
| **Congo, Dem. Republic of the** | **GDPrGiniBase** | 71.7 | 71.03 | 69.58 | 55.09 | 9.246 |
| **Congo, Dem. Republic of the** | **GDPrHGiniH** | 71.86 | 71.21 | 69.18 | 54.15 | 9.116 |
| **Congo, Dem. Republic of the** | **GDPrHGiniHv** | 72.12 | 71.49 | 69.53 | 55.06 | 10.36 |
| **Congo, Dem. Republic of the** | **GDPrHGiniLow** | 71.55 | 70.86 | 68.73 | 52.91 | 7.564 |
| **Congo, Dem. Republic of the** | **GDPrLGiniH** | 71.86 | 71.21 | 70.41 | 57.19 | 11.12 |
| **Congo, Dem. Republic of the** | **GDPrLGiniHv** | 72.12 | 71.49 | 70.72 | 57.99 | 12.47 |
| **Congo, Dem. Republic of the** | **GDPrLGiniLow** | 71.55 | 70.86 | 70.03 | 56.09 | 9.395 |
| **Congo, Dem. Republic of the** | **NoCOVIDginiB** | 70.8 | 70.64 | 69.93 | 56.47 | 10.41 |
| **Costa Rica** | **GDPrGiniBase** | 1.065 | 1.009 | 0.94 | 0.584 | 0.206 |
| **Costa Rica** | **GDPrHGiniH** | 1.288 | 1.224 | 1.105 | 0.685 | 0.252 |
| **Costa Rica** | **GDPrHGiniHv** | 1.681 | 1.603 | 1.458 | 0.934 | 0.368 |
| **Costa Rica** | **GDPrHGiniLow** | 0.871 | 0.823 | 0.736 | 0.434 | 0.144 |
| **Costa Rica** | **GDPrLGiniH** | 1.288 | 1.224 | 1.185 | 0.772 | 0.289 |
| **Costa Rica** | **GDPrLGiniHv** | 1.681 | 1.603 | 1.556 | 1.044 | 0.419 |
| **Costa Rica** | **GDPrLGiniLow** | 0.871 | 0.823 | 0.795 | 0.495 | 0.168 |
| **Costa Rica** | **NoCOVIDginiB** | 0.899 | 0.871 | 0.835 | 0.531 | 0.179 |
| **Cote D'Ivoire** | **GDPrGiniBase** | 22.39 | 21.41 | 20.14 | 13.65 | 3.268 |
| **Cote D'Ivoire** | **GDPrHGiniH** | 23.4 | 22.42 | 20.69 | 13.98 | 3.446 |
| **Cote D'Ivoire** | **GDPrHGiniHv** | 24.91 | 23.93 | 22.19 | 15.36 | 4.145 |
| **Cote D'Ivoire** | **GDPrHGiniLow** | 21.37 | 20.4 | 18.7 | 12.19 | 2.63 |
| **Cote D'Ivoire** | **GDPrLGiniH** | 23.4 | 22.42 | 21.6 | 15.19 | 4.011 |
| **Cote D'Ivoire** | **GDPrLGiniHv** | 24.91 | 23.93 | 23.11 | 16.6 | 4.78 |
| **Cote D'Ivoire** | **GDPrLGiniLow** | 21.37 | 20.4 | 19.6 | 13.35 | 3.104 |
| **Cote D'Ivoire** | **NoCOVIDginiB** | 20.64 | 19.37 | 18.3 | 12.42 | 2.693 |
| **Croatia** | **GDPrGiniBase** | 0.399 | 0.37 | 0.353 | 0.065 | 0.011 |
| **Croatia** | **GDPrHGiniH** | 0.485 | 0.451 | 0.415 | 0.071 | 0.013 |
| **Croatia** | **GDPrHGiniHv** | 0.637 | 0.595 | 0.55 | 0.104 | 0.021 |
| **Croatia** | **GDPrHGiniLow** | 0.325 | 0.301 | 0.274 | 0.04 | 0.006 |
| **Croatia** | **GDPrLGiniH** | 0.485 | 0.451 | 0.444 | 0.099 | 0.018 |
| **Croatia** | **GDPrLGiniHv** | 0.637 | 0.595 | 0.586 | 0.143 | 0.029 |
| **Croatia** | **GDPrLGiniLow** | 0.325 | 0.301 | 0.295 | 0.058 | 0.009 |
| **Croatia** | **NoCOVIDginiB** | 0.302 | 0.276 | 0.257 | 0.059 | 0.01 |
| **Cuba** | **GDPrGiniBase** | 1.855 | 1.759 | 1.741 | 1.006 | 0.395 |
| **Cuba** | **GDPrHGiniH** | 2.157 | 2.05 | 1.98 | 1.126 | 0.447 |
| **Cuba** | **GDPrHGiniHv** | 2.665 | 2.541 | 2.46 | 1.451 | 0.612 |
| **Cuba** | **GDPrHGiniLow** | 1.582 | 1.496 | 1.44 | 0.776 | 0.282 |
| **Cuba** | **GDPrLGiniH** | 2.157 | 2.05 | 2.081 | 1.291 | 0.541 |
| **Cuba** | **GDPrLGiniHv** | 2.665 | 2.541 | 2.578 | 1.65 | 0.732 |
| **Cuba** | **GDPrLGiniLow** | 1.582 | 1.496 | 1.521 | 0.901 | 0.347 |
| **Cuba** | **NoCOVIDginiB** | 1.518 | 1.519 | 1.502 | 0.843 | 0.266 |
| **Cyprus** | **GDPrGiniBase** | 0.123 | 0.127 | 0.132 | 0.084 | 0.034 |
| **Cyprus** | **GDPrHGiniH** | 0.157 | 0.162 | 0.157 | 0.097 | 0.038 |
| **Cyprus** | **GDPrHGiniHv** | 0.222 | 0.228 | 0.221 | 0.14 | 0.058 |
| **Cyprus** | **GDPrHGiniLow** | 0.095 | 0.098 | 0.095 | 0.056 | 0.02 |
| **Cyprus** | **GDPrLGiniH** | 0.157 | 0.162 | 0.182 | 0.123 | 0.056 |
| **Cyprus** | **GDPrLGiniHv** | 0.222 | 0.228 | 0.254 | 0.176 | 0.084 |
| **Cyprus** | **GDPrLGiniLow** | 0.095 | 0.098 | 0.111 | 0.073 | 0.031 |
| **Cyprus** | **NoCOVIDginiB** | 0.086 | 0.093 | 0.1 | 0.074 | 0.03 |
| **Czech Republic** | **GDPrGiniBase** | 0.114 | 0.123 | 0.138 | 0.042 | 0.004 |
| **Czech Republic** | **GDPrHGiniH** | 0.144 | 0.154 | 0.165 | 0.045 | 0.005 |
| **Czech Republic** | **GDPrHGiniHv** | 0.2 | 0.214 | 0.228 | 0.067 | 0.008 |
| **Czech Republic** | **GDPrHGiniLow** | 0.089 | 0.096 | 0.103 | 0.025 | 0.002 |
| **Czech Republic** | **GDPrLGiniH** | 0.144 | 0.154 | 0.181 | 0.065 | 0.008 |
| **Czech Republic** | **GDPrLGiniHv** | 0.2 | 0.214 | 0.248 | 0.094 | 0.014 |
| **Czech Republic** | **GDPrLGiniLow** | 0.089 | 0.096 | 0.114 | 0.037 | 0.004 |
| **Czech Republic** | **NoCOVIDginiB** | 0.087 | 0.087 | 0.089 | 0.033 | 0.003 |
| **Denmark** | **GDPrGiniBase** | 0.104 | 0.096 | 0.088 | 0.025 | 0.002 |
| **Denmark** | **GDPrHGiniH** | 0.133 | 0.123 | 0.11 | 0.03 | 0.002 |
| **Denmark** | **GDPrHGiniHv** | 0.187 | 0.174 | 0.156 | 0.046 | 0.004 |
| **Denmark** | **GDPrHGiniLow** | 0.08 | 0.074 | 0.065 | 0.016 | 0.001 |
| **Denmark** | **GDPrLGiniH** | 0.133 | 0.123 | 0.118 | 0.04 | 0.003 |
| **Denmark** | **GDPrLGiniHv** | 0.187 | 0.174 | 0.168 | 0.061 | 0.005 |
| **Denmark** | **GDPrLGiniLow** | 0.08 | 0.074 | 0.071 | 0.022 | 0.001 |
| **Denmark** | **NoCOVIDginiB** | 0.094 | 0.087 | 0.082 | 0.025 | 0.002 |
| **Djibouti** | **GDPrGiniBase** | 16.2 | 16.99 | 17.17 | 13.18 | 3.224 |
| **Djibouti** | **GDPrHGiniH** | 17.16 | 17.96 | 17.57 | 13.22 | 3.412 |
| **Djibouti** | **GDPrHGiniHv** | 18.62 | 19.44 | 19.03 | 14.58 | 4.108 |
| **Djibouti** | **GDPrHGiniLow** | 15.25 | 16.02 | 15.65 | 11.48 | 2.597 |
| **Djibouti** | **GDPrLGiniH** | 17.16 | 17.96 | 18.75 | 14.85 | 3.946 |
| **Djibouti** | **GDPrLGiniHv** | 18.62 | 19.44 | 20.23 | 16.25 | 4.709 |
| **Djibouti** | **GDPrLGiniLow** | 15.25 | 16.02 | 16.8 | 13.03 | 3.045 |
| **Djibouti** | **NoCOVIDginiB** | 13.98 | 14.61 | 15 | 11.36 | 2.761 |
| **Dominican Republic** | **GDPrGiniBase** | 0.983 | 0.833 | 0.75 | 0.422 | 0.113 |
| **Dominican Republic** | **GDPrHGiniH** | 1.181 | 1.008 | 0.873 | 0.492 | 0.138 |
| **Dominican Republic** | **GDPrHGiniHv** | 1.527 | 1.318 | 1.151 | 0.674 | 0.206 |
| **Dominican Republic** | **GDPrHGiniLow** | 0.81 | 0.681 | 0.581 | 0.309 | 0.076 |
| **Dominican Republic** | **GDPrLGiniH** | 1.181 | 1.008 | 0.95 | 0.561 | 0.162 |
| **Dominican Republic** | **GDPrLGiniHv** | 1.527 | 1.318 | 1.246 | 0.763 | 0.24 |
| **Dominican Republic** | **GDPrLGiniLow** | 0.81 | 0.681 | 0.638 | 0.358 | 0.091 |
| **Dominican Republic** | **NoCOVIDginiB** | 0.688 | 0.657 | 0.614 | 0.359 | 0.091 |
| **Ecuador** | **GDPrGiniBase** | 4.661 | 4.814 | 4.807 | 2.967 | 1.885 |
| **Ecuador** | **GDPrHGiniH** | 5.238 | 5.402 | 5.286 | 3.204 | 2.079 |
| **Ecuador** | **GDPrHGiniHv** | 6.173 | 6.353 | 6.226 | 3.904 | 2.609 |
| **Ecuador** | **GDPrHGiniLow** | 4.122 | 4.263 | 4.162 | 2.401 | 1.49 |
| **Ecuador** | **GDPrLGiniH** | 5.238 | 5.402 | 5.508 | 3.619 | 2.344 |
| **Ecuador** | **GDPrLGiniHv** | 6.173 | 6.353 | 6.471 | 4.373 | 2.918 |
| **Ecuador** | **GDPrLGiniLow** | 4.122 | 4.263 | 4.355 | 2.743 | 1.7 |
| **Ecuador** | **NoCOVIDginiB** | 4.087 | 4.146 | 3.935 | 2.575 | 1.683 |
| **Egypt** | **GDPrGiniBase** | 1.161 | 1.192 | 1.134 | 0.905 | 0.142 |
| **Egypt** | **GDPrHGiniH** | 1.345 | 1.38 | 1.235 | 0.955 | 0.156 |
| **Egypt** | **GDPrHGiniHv** | 1.654 | 1.695 | 1.527 | 1.197 | 0.218 |
| **Egypt** | **GDPrHGiniLow** | 0.993 | 1.022 | 0.906 | 0.685 | 0.096 |
| **Egypt** | **GDPrLGiniH** | 1.345 | 1.38 | 1.376 | 1.176 | 0.207 |
| **Egypt** | **GDPrLGiniHv** | 1.654 | 1.695 | 1.689 | 1.458 | 0.285 |
| **Egypt** | **GDPrLGiniLow** | 0.993 | 1.022 | 1.018 | 0.859 | 0.13 |
| **Egypt** | **NoCOVIDginiB** | 1.062 | 0.989 | 0.925 | 0.742 | 0.106 |
| **El Salvador** | **GDPrGiniBase** | 2.618 | 2.206 | 2.09 | 1.322 | 0.32 |
| **El Salvador** | **GDPrHGiniH** | 2.983 | 2.532 | 2.283 | 1.436 | 0.369 |
| **El Salvador** | **GDPrHGiniHv** | 3.583 | 3.074 | 2.788 | 1.811 | 0.51 |
| **El Salvador** | **GDPrHGiniLow** | 2.281 | 1.907 | 1.703 | 1.022 | 0.23 |
| **El Salvador** | **GDPrLGiniH** | 2.983 | 2.532 | 2.505 | 1.67 | 0.435 |
| **El Salvador** | **GDPrLGiniHv** | 3.583 | 3.074 | 3.042 | 2.084 | 0.593 |
| **El Salvador** | **GDPrLGiniLow** | 2.281 | 1.907 | 1.885 | 1.207 | 0.275 |
| **El Salvador** | **NoCOVIDginiB** | 1.749 | 1.888 | 1.923 | 1.25 | 0.297 |
| **Equatorial Guinea** | **GDPrGiniBase** | 2.615 | 2.523 | 2.893 | 1.693 | 0.604 |
| **Equatorial Guinea** | **GDPrHGiniH** | 3.053 | 2.951 | 3.26 | 1.882 | 0.713 |
| **Equatorial Guinea** | **GDPrHGiniHv** | 3.795 | 3.676 | 4.033 | 2.421 | 0.984 |
| **Equatorial Guinea** | **GDPrHGiniLow** | 2.221 | 2.139 | 2.388 | 1.301 | 0.445 |
| **Equatorial Guinea** | **GDPrLGiniH** | 3.053 | 2.951 | 3.468 | 2.172 | 0.804 |
| **Equatorial Guinea** | **GDPrLGiniHv** | 3.795 | 3.676 | 4.271 | 2.767 | 1.1 |
| **Equatorial Guinea** | **GDPrLGiniLow** | 2.221 | 2.139 | 2.556 | 1.525 | 0.508 |
| **Equatorial Guinea** | **NoCOVIDginiB** | 2.62 | 3.078 | 3.452 | 2.146 | 0.766 |
| **Eritrea** | **GDPrGiniBase** | 36.95 | 40.15 | 42.88 | 31.97 | 2.039 |
| **Eritrea** | **GDPrHGiniH** | 37.96 | 41.12 | 43.06 | 31.89 | 2.193 |
| **Eritrea** | **GDPrHGiniHv** | 39.44 | 42.55 | 44.4 | 33.4 | 2.757 |
| **Eritrea** | **GDPrHGiniLow** | 35.94 | 39.17 | 41.23 | 29.84 | 1.555 |
| **Eritrea** | **GDPrLGiniH** | 37.96 | 41.12 | 44.51 | 34.1 | 2.63 |
| **Eritrea** | **GDPrLGiniHv** | 39.44 | 42.55 | 45.82 | 35.59 | 3.281 |
| **Eritrea** | **GDPrLGiniLow** | 35.94 | 39.17 | 42.74 | 32.08 | 1.901 |
| **Eritrea** | **NoCOVIDginiB** | 34.87 | 37.34 | 39.16 | 28.77 | 1.398 |
| **Estonia** | **GDPrGiniBase** | 0.307 | 0.266 | 0.251 | 0.136 | 0.014 |
| **Estonia** | **GDPrHGiniH** | 0.377 | 0.328 | 0.29 | 0.148 | 0.015 |
| **Estonia** | **GDPrHGiniHv** | 0.503 | 0.441 | 0.392 | 0.208 | 0.025 |
| **Estonia** | **GDPrHGiniLow** | 0.247 | 0.212 | 0.186 | 0.089 | 0.007 |
| **Estonia** | **GDPrLGiniH** | 0.377 | 0.328 | 0.334 | 0.202 | 0.024 |
| **Estonia** | **GDPrLGiniHv** | 0.503 | 0.441 | 0.448 | 0.28 | 0.038 |
| **Estonia** | **GDPrLGiniLow** | 0.247 | 0.212 | 0.217 | 0.125 | 0.012 |
| **Estonia** | **NoCOVIDginiB** | 0.228 | 0.26 | 0.292 | 0.139 | 0.016 |
| **Eswatini** | **GDPrGiniBase** | 28.72 | 28.97 | 29.03 | 22.27 | 10.78 |
| **Eswatini** | **GDPrHGiniH** | 30.06 | 30.3 | 29.92 | 22.98 | 11.31 |
| **Eswatini** | **GDPrHGiniHv** | 32.07 | 32.31 | 31.93 | 25 | 12.99 |
| **Eswatini** | **GDPrHGiniLow** | 27.39 | 27.63 | 27.25 | 20.35 | 9.248 |
| **Eswatini** | **GDPrLGiniH** | 30.06 | 30.3 | 30.83 | 24.21 | 12.42 |
| **Eswatini** | **GDPrLGiniHv** | 32.07 | 32.31 | 32.83 | 26.23 | 14.17 |
| **Eswatini** | **GDPrLGiniLow** | 27.39 | 27.63 | 28.16 | 21.56 | 10.26 |
| **Eswatini** | **NoCOVIDginiB** | 27.8 | 28.41 | 28.94 | 22.56 | 11.04 |
| **Ethiopia** | **GDPrGiniBase** | 21.13 | 21.67 | 19.74 | 10.07 | 0.724 |
| **Ethiopia** | **GDPrHGiniH** | 22.02 | 22.58 | 19.96 | 10.09 | 0.749 |
| **Ethiopia** | **GDPrHGiniHv** | 23.35 | 23.93 | 21.27 | 11.16 | 0.989 |
| **Ethiopia** | **GDPrHGiniLow** | 20.23 | 20.76 | 18.21 | 8.706 | 0.497 |
| **Ethiopia** | **GDPrLGiniH** | 22.02 | 22.58 | 21.3 | 11.56 | 1.042 |
| **Ethiopia** | **GDPrLGiniHv** | 23.35 | 23.93 | 22.62 | 12.7 | 1.349 |
| **Ethiopia** | **GDPrLGiniLow** | 20.23 | 20.76 | 19.53 | 10.1 | 0.706 |
| **Ethiopia** | **NoCOVIDginiB** | 20.04 | 18.9 | 17.9 | 9.164 | 0.607 |
| **Fiji** | **GDPrGiniBase** | 0.478 | 0.714 | 0.709 | 0.781 | 0.15 |
| **Fiji** | **GDPrHGiniH** | 0.584 | 0.857 | 0.804 | 0.831 | 0.172 |
| **Fiji** | **GDPrHGiniHv** | 0.772 | 1.107 | 1.042 | 1.075 | 0.246 |
| **Fiji** | **GDPrHGiniLow** | 0.387 | 0.589 | 0.549 | 0.569 | 0.101 |
| **Fiji** | **GDPrLGiniH** | 0.584 | 0.857 | 0.901 | 1.048 | 0.218 |
| **Fiji** | **GDPrLGiniHv** | 0.772 | 1.107 | 1.159 | 1.337 | 0.307 |
| **Fiji** | **GDPrLGiniLow** | 0.387 | 0.589 | 0.622 | 0.734 | 0.131 |
| **Fiji** | **NoCOVIDginiB** | 0.203 | 0.216 | 0.236 | 0.41 | 0.059 |
| **Finland** | **GDPrGiniBase** | 0.11 | 0.099 | 0.086 | 0.053 | 0.017 |
| **Finland** | **GDPrHGiniH** | 0.14 | 0.127 | 0.108 | 0.059 | 0.019 |
| **Finland** | **GDPrHGiniHv** | 0.197 | 0.178 | 0.154 | 0.087 | 0.029 |
| **Finland** | **GDPrHGiniLow** | 0.085 | 0.076 | 0.064 | 0.033 | 0.01 |
| **Finland** | **GDPrLGiniH** | 0.14 | 0.127 | 0.114 | 0.081 | 0.028 |
| **Finland** | **GDPrLGiniHv** | 0.197 | 0.178 | 0.161 | 0.118 | 0.044 |
| **Finland** | **GDPrLGiniLow** | 0.085 | 0.076 | 0.068 | 0.047 | 0.015 |
| **Finland** | **NoCOVIDginiB** | 0.102 | 0.088 | 0.077 | 0.052 | 0.016 |
| **France** | **GDPrGiniBase** | 0.122 | 0.127 | 0.125 | 0.063 | 0.024 |
| **France** | **GDPrHGiniH** | 0.156 | 0.162 | 0.156 | 0.073 | 0.028 |
| **France** | **GDPrHGiniHv** | 0.22 | 0.227 | 0.22 | 0.107 | 0.044 |
| **France** | **GDPrHGiniLow** | 0.094 | 0.098 | 0.094 | 0.041 | 0.015 |
| **France** | **GDPrLGiniH** | 0.156 | 0.162 | 0.163 | 0.095 | 0.039 |
| **France** | **GDPrLGiniHv** | 0.22 | 0.227 | 0.229 | 0.139 | 0.059 |
| **France** | **GDPrLGiniLow** | 0.094 | 0.098 | 0.099 | 0.055 | 0.02 |
| **France** | **NoCOVIDginiB** | 0.107 | 0.101 | 0.096 | 0.061 | 0.023 |
| **Gabon** | **GDPrGiniBase** | 3.488 | 3.211 | 2.674 | 0.764 | 0.564 |
| **Gabon** | **GDPrHGiniH** | 3.923 | 3.624 | 2.919 | 0.854 | 0.611 |
| **Gabon** | **GDPrHGiniHv** | 4.628 | 4.296 | 3.507 | 1.107 | 0.811 |
| **Gabon** | **GDPrHGiniLow** | 3.081 | 2.826 | 2.232 | 0.583 | 0.404 |
| **Gabon** | **GDPrLGiniH** | 3.923 | 3.624 | 3.17 | 0.98 | 0.773 |
| **Gabon** | **GDPrLGiniHv** | 4.628 | 4.296 | 3.788 | 1.26 | 1.01 |
| **Gabon** | **GDPrLGiniLow** | 3.081 | 2.826 | 2.442 | 0.678 | 0.522 |
| **Gabon** | **NoCOVIDginiB** | 2.993 | 2.621 | 2.192 | 0.602 | 0.374 |
| **Gambia** | **GDPrGiniBase** | 9.382 | 9.66 | 9.444 | 5.448 | 0.7 |
| **Gambia** | **GDPrHGiniH** | 10.1 | 10.39 | 9.706 | 5.588 | 0.732 |
| **Gambia** | **GDPrHGiniHv** | 11.22 | 11.52 | 10.79 | 6.416 | 0.963 |
| **Gambia** | **GDPrHGiniLow** | 8.679 | 8.945 | 8.314 | 4.568 | 0.489 |
| **Gambia** | **GDPrLGiniH** | 10.1 | 10.39 | 10.6 | 6.403 | 0.967 |
| **Gambia** | **GDPrLGiniHv** | 11.22 | 11.52 | 11.73 | 7.293 | 1.249 |
| **Gambia** | **GDPrLGiniLow** | 8.679 | 8.945 | 9.149 | 5.296 | 0.663 |
| **Gambia** | **NoCOVIDginiB** | 7.629 | 7.743 | 8.123 | 4.74 | 0.524 |
| **Georgia** | **GDPrGiniBase** | 4.069 | 3.694 | 3.342 | 1.601 | 0.479 |
| **Georgia** | **GDPrHGiniH** | 4.537 | 4.137 | 3.572 | 1.686 | 0.546 |
| **Georgia** | **GDPrHGiniHv** | 5.286 | 4.85 | 4.221 | 2.084 | 0.727 |
| **Georgia** | **GDPrHGiniLow** | 3.628 | 3.279 | 2.797 | 1.236 | 0.359 |
| **Georgia** | **GDPrLGiniH** | 4.537 | 4.137 | 3.924 | 2.02 | 0.624 |
| **Georgia** | **GDPrLGiniHv** | 5.286 | 4.85 | 4.611 | 2.469 | 0.823 |
| **Georgia** | **GDPrLGiniLow** | 3.628 | 3.279 | 3.1 | 1.505 | 0.416 |
| **Georgia** | **NoCOVIDginiB** | 2.72 | 2.644 | 2.512 | 1.296 | 0.384 |
| **Germany** | **GDPrGiniBase** | 0.107 | 0.108 | 0.108 | 0.062 | 0.022 |
| **Germany** | **GDPrHGiniH** | 0.137 | 0.139 | 0.135 | 0.073 | 0.026 |
| **Germany** | **GDPrHGiniHv** | 0.196 | 0.197 | 0.192 | 0.108 | 0.04 |
| **Germany** | **GDPrHGiniLow** | 0.082 | 0.083 | 0.08 | 0.041 | 0.013 |
| **Germany** | **GDPrLGiniH** | 0.137 | 0.139 | 0.143 | 0.093 | 0.036 |
| **Germany** | **GDPrLGiniHv** | 0.196 | 0.197 | 0.203 | 0.135 | 0.056 |
| **Germany** | **GDPrLGiniLow** | 0.082 | 0.083 | 0.086 | 0.053 | 0.019 |
| **Germany** | **NoCOVIDginiB** | 0.094 | 0.092 | 0.089 | 0.061 | 0.022 |
| **Ghana** | **GDPrGiniBase** | 11.71 | 11.54 | 10.81 | 7.03 | 1.194 |
| **Ghana** | **GDPrHGiniH** | 12.6 | 12.43 | 11.36 | 7.366 | 1.301 |
| **Ghana** | **GDPrHGiniHv** | 13.97 | 13.8 | 12.69 | 8.456 | 1.684 |
| **Ghana** | **GDPrHGiniLow** | 10.84 | 10.67 | 9.682 | 6.023 | 0.89 |
| **Ghana** | **GDPrLGiniH** | 12.6 | 12.43 | 11.99 | 8.111 | 1.58 |
| **Ghana** | **GDPrLGiniHv** | 13.97 | 13.8 | 13.34 | 9.253 | 2.02 |
| **Ghana** | **GDPrLGiniLow** | 10.84 | 10.67 | 10.26 | 6.693 | 1.101 |
| **Ghana** | **NoCOVIDginiB** | 10.62 | 10.49 | 10.23 | 6.672 | 1.048 |
| **Greece** | **GDPrGiniBase** | 0.87 | 0.782 | 0.765 | 0.55 | 0.161 |
| **Greece** | **GDPrHGiniH** | 1.031 | 0.93 | 0.891 | 0.592 | 0.182 |
| **Greece** | **GDPrHGiniHv** | 1.308 | 1.188 | 1.141 | 0.777 | 0.257 |
| **Greece** | **GDPrHGiniLow** | 0.727 | 0.65 | 0.62 | 0.397 | 0.11 |
| **Greece** | **GDPrLGiniH** | 1.031 | 0.93 | 0.932 | 0.745 | 0.228 |
| **Greece** | **GDPrLGiniHv** | 1.308 | 1.188 | 1.19 | 0.964 | 0.317 |
| **Greece** | **GDPrLGiniLow** | 0.727 | 0.65 | 0.651 | 0.51 | 0.14 |
| **Greece** | **NoCOVIDginiB** | 0.723 | 0.672 | 0.636 | 0.515 | 0.147 |
| **Grenada** | **GDPrGiniBase** | 10.69 | 11.24 | 10.39 | 6.814 | 2.946 |
| **Grenada** | **GDPrHGiniH** | 11.53 | 12.09 | 10.9 | 7.041 | 3.188 |
| **Grenada** | **GDPrHGiniHv** | 12.81 | 13.41 | 12.16 | 8.064 | 3.849 |
| **Grenada** | **GDPrHGiniLow** | 9.884 | 10.41 | 9.31 | 5.777 | 2.418 |
| **Grenada** | **GDPrLGiniH** | 11.53 | 12.09 | 11.52 | 7.916 | 3.519 |
| **Grenada** | **GDPrLGiniHv** | 12.81 | 13.41 | 12.8 | 8.997 | 4.221 |
| **Grenada** | **GDPrLGiniLow** | 9.884 | 10.41 | 9.884 | 6.568 | 2.697 |
| **Grenada** | **NoCOVIDginiB** | 7.49 | 7.761 | 7.795 | 5.31 | 2.259 |
| **Guatemala** | **GDPrGiniBase** | 7.69 | 7.29 | 6.976 | 5.751 | 2.904 |
| **Guatemala** | **GDPrHGiniH** | 8.488 | 8.066 | 7.528 | 6.171 | 3.185 |
| **Guatemala** | **GDPrHGiniHv** | 9.755 | 9.303 | 8.722 | 7.258 | 3.932 |
| **Guatemala** | **GDPrHGiniLow** | 6.93 | 6.553 | 6.073 | 4.869 | 2.339 |
| **Guatemala** | **GDPrLGiniH** | 8.488 | 8.066 | 7.921 | 6.7 | 3.55 |
| **Guatemala** | **GDPrLGiniHv** | 9.755 | 9.303 | 9.146 | 7.834 | 4.349 |
| **Guatemala** | **GDPrLGiniLow** | 6.93 | 6.553 | 6.423 | 5.332 | 2.637 |
| **Guatemala** | **NoCOVIDginiB** | 6.97 | 6.887 | 6.772 | 5.629 | 2.794 |
| **Guinea** | **GDPrGiniBase** | 21.42 | 21.18 | 20.36 | 21.48 | 3.504 |
| **Guinea** | **GDPrHGiniH** | 22.29 | 22.06 | 20.61 | 21.25 | 3.557 |
| **Guinea** | **GDPrHGiniHv** | 23.59 | 23.36 | 21.9 | 22.55 | 4.207 |
| **Guinea** | **GDPrHGiniLow** | 20.54 | 20.3 | 18.87 | 19.5 | 2.781 |
| **Guinea** | **GDPrLGiniH** | 22.29 | 22.06 | 21.89 | 23.51 | 4.318 |
| **Guinea** | **GDPrLGiniHv** | 23.59 | 23.36 | 23.19 | 24.82 | 5.049 |
| **Guinea** | **GDPrLGiniLow** | 20.54 | 20.3 | 20.14 | 21.75 | 3.435 |
| **Guinea** | **NoCOVIDginiB** | 21.35 | 21.07 | 21.26 | 21.67 | 3.917 |
| **Guinea Bissau** | **GDPrGiniBase** | 65.88 | 65.8 | 65.38 | 58.73 | 40.08 |
| **Guinea Bissau** | **GDPrHGiniH** | 66.36 | 66.28 | 65.24 | 58.2 | 39.31 |
| **Guinea Bissau** | **GDPrHGiniHv** | 67.08 | 67.01 | 66 | 59.26 | 41.12 |
| **Guinea Bissau** | **GDPrHGiniLow** | 65.39 | 65.31 | 64.21 | 56.76 | 36.86 |
| **Guinea Bissau** | **GDPrLGiniH** | 66.36 | 66.28 | 66.41 | 60.69 | 43.35 |
| **Guinea Bissau** | **GDPrLGiniHv** | 67.08 | 67.01 | 67.13 | 61.65 | 45.05 |
| **Guinea Bissau** | **GDPrLGiniLow** | 65.39 | 65.31 | 65.45 | 59.38 | 41.03 |
| **Guinea Bissau** | **NoCOVIDginiB** | 63.22 | 62.38 | 61.57 | 54.76 | 33.33 |
| **Guyana** | **GDPrGiniBase** | 2.205 | 1.491 | 0.551 | 0.068 | 0.017 |
| **Guyana** | **GDPrHGiniH** | 2.559 | 1.761 | 0.662 | 0.085 | 0.023 |
| **Guyana** | **GDPrHGiniHv** | 3.154 | 2.225 | 0.893 | 0.132 | 0.04 |
| **Guyana** | **GDPrHGiniLow** | 1.884 | 1.25 | 0.427 | 0.045 | 0.011 |
| **Guyana** | **GDPrLGiniH** | 2.559 | 1.761 | 0.7 | 0.1 | 0.026 |
| **Guyana** | **GDPrLGiniHv** | 3.154 | 2.225 | 0.94 | 0.153 | 0.043 |
| **Guyana** | **GDPrLGiniLow** | 1.884 | 1.25 | 0.454 | 0.053 | 0.012 |
| **Guyana** | **NoCOVIDginiB** | 1.137 | 1.011 | 0.746 | 0.095 | 0.022 |
| **Haiti** | **GDPrGiniBase** | 28.96 | 30.16 | 30.78 | 32.41 | 21.25 |
| **Haiti** | **GDPrHGiniH** | 29.95 | 31.14 | 31.05 | 31.98 | 20.75 |
| **Haiti** | **GDPrHGiniHv** | 31.42 | 32.6 | 32.51 | 33.43 | 22.31 |
| **Haiti** | **GDPrHGiniLow** | 27.96 | 29.17 | 29.07 | 30 | 18.67 |
| **Haiti** | **GDPrLGiniH** | 29.95 | 31.14 | 32.35 | 34.71 | 24.06 |
| **Haiti** | **GDPrLGiniHv** | 31.42 | 32.6 | 33.79 | 36.12 | 25.64 |
| **Haiti** | **GDPrLGiniLow** | 27.96 | 29.17 | 30.4 | 32.79 | 21.94 |
| **Haiti** | **NoCOVIDginiB** | 26.97 | 27.21 | 27.57 | 29.71 | 17.65 |
| **Honduras** | **GDPrGiniBase** | 21.98 | 21.9 | 21.36 | 18.07 | 10.4 |
| **Honduras** | **GDPrHGiniH** | 23.23 | 23.15 | 22.15 | 18.62 | 10.95 |
| **Honduras** | **GDPrHGiniHv** | 25.13 | 25.05 | 24.03 | 20.46 | 12.52 |
| **Honduras** | **GDPrHGiniLow** | 20.74 | 20.66 | 19.68 | 16.25 | 9 |
| **Honduras** | **GDPrLGiniH** | 23.23 | 23.15 | 23 | 19.89 | 11.9 |
| **Honduras** | **GDPrLGiniHv** | 25.13 | 25.05 | 24.89 | 21.75 | 13.53 |
| **Honduras** | **GDPrLGiniLow** | 20.74 | 20.66 | 20.52 | 17.48 | 9.875 |
| **Honduras** | **NoCOVIDginiB** | 18.26 | 18.29 | 18.18 | 15.82 | 8.625 |
| **Hong Kong** | **GDPrGiniBase** | 0.157 | 0.138 | 0.129 | 0.194 | 0.044 |
| **Hong Kong** | **GDPrHGiniH** | 0.203 | 0.179 | 0.159 | 0.232 | 0.053 |
| **Hong Kong** | **GDPrHGiniHv** | 0.291 | 0.259 | 0.232 | 0.331 | 0.083 |
| **Hong Kong** | **GDPrHGiniLow** | 0.119 | 0.104 | 0.091 | 0.138 | 0.028 |
| **Hong Kong** | **GDPrLGiniH** | 0.203 | 0.179 | 0.177 | 0.266 | 0.068 |
| **Hong Kong** | **GDPrLGiniHv** | 0.291 | 0.259 | 0.256 | 0.376 | 0.105 |
| **Hong Kong** | **GDPrLGiniLow** | 0.119 | 0.104 | 0.103 | 0.161 | 0.036 |
| **Hong Kong** | **NoCOVIDginiB** | 0.117 | 0.12 | 0.112 | 0.191 | 0.039 |
| **Hungary** | **GDPrGiniBase** | 0.395 | 0.393 | 0.368 | 0.099 | 0.015 |
| **Hungary** | **GDPrHGiniH** | 0.481 | 0.478 | 0.417 | 0.108 | 0.017 |
| **Hungary** | **GDPrHGiniHv** | 0.632 | 0.629 | 0.553 | 0.155 | 0.027 |
| **Hungary** | **GDPrHGiniLow** | 0.322 | 0.32 | 0.276 | 0.063 | 0.008 |
| **Hungary** | **GDPrLGiniH** | 0.481 | 0.478 | 0.462 | 0.15 | 0.026 |
| **Hungary** | **GDPrLGiniHv** | 0.632 | 0.629 | 0.609 | 0.211 | 0.04 |
| **Hungary** | **GDPrLGiniLow** | 0.322 | 0.32 | 0.308 | 0.09 | 0.013 |
| **Hungary** | **NoCOVIDginiB** | 0.33 | 0.329 | 0.334 | 0.11 | 0.016 |
| **Iceland** | **GDPrGiniBase** | 0.168 | 0.217 | 0.364 | 0.071 | 0.001 |
| **Iceland** | **GDPrHGiniH** | 0.21 | 0.269 | 0.404 | 0.075 | 0.001 |
| **Iceland** | **GDPrHGiniHv** | 0.287 | 0.361 | 0.531 | 0.108 | 0.002 |
| **Iceland** | **GDPrHGiniLow** | 0.133 | 0.173 | 0.27 | 0.043 | 0 |
| **Iceland** | **GDPrLGiniH** | 0.21 | 0.269 | 0.47 | 0.111 | 0.002 |
| **Iceland** | **GDPrLGiniHv** | 0.287 | 0.361 | 0.613 | 0.156 | 0.003 |
| **Iceland** | **GDPrLGiniLow** | 0.133 | 0.173 | 0.319 | 0.067 | 0.001 |
| **Iceland** | **NoCOVIDginiB** | 0.102 | 0.14 | 0.226 | 0.069 | 0.001 |
| **India** | **GDPrGiniBase** | 9.705 | 9 | 8.346 | 3.495 | 0.508 |
| **India** | **GDPrHGiniH** | 10.46 | 9.728 | 8.769 | 3.651 | 0.559 |
| **India** | **GDPrHGiniHv** | 11.62 | 10.86 | 9.846 | 4.324 | 0.745 |
| **India** | **GDPrHGiniLow** | 8.971 | 8.292 | 7.407 | 2.849 | 0.366 |
| **India** | **GDPrLGiniH** | 10.46 | 9.728 | 9.27 | 4.221 | 0.694 |
| **India** | **GDPrLGiniHv** | 11.62 | 10.86 | 10.38 | 4.956 | 0.914 |
| **India** | **GDPrLGiniLow** | 8.971 | 8.292 | 7.868 | 3.338 | 0.464 |
| **India** | **NoCOVIDginiB** | 7.322 | 6.371 | 5.542 | 2.411 | 0.329 |
| **Indonesia** | **GDPrGiniBase** | 3.625 | 3.512 | 3.162 | 1.918 | 0.96 |
| **Indonesia** | **GDPrHGiniH** | 4.077 | 3.954 | 3.441 | 2.054 | 1.052 |
| **Indonesia** | **GDPrHGiniHv** | 4.808 | 4.672 | 4.102 | 2.53 | 1.352 |
| **Indonesia** | **GDPrHGiniLow** | 3.203 | 3.099 | 2.662 | 1.514 | 0.73 |
| **Indonesia** | **GDPrLGiniH** | 4.077 | 3.954 | 3.717 | 2.4 | 1.24 |
| **Indonesia** | **GDPrLGiniHv** | 4.808 | 4.672 | 4.41 | 2.928 | 1.577 |
| **Indonesia** | **GDPrLGiniLow** | 3.203 | 3.099 | 2.896 | 1.793 | 0.873 |
| **Indonesia** | **NoCOVIDginiB** | 3.025 | 2.691 | 2.393 | 1.526 | 0.757 |
| **Iran** | **GDPrGiniBase** | 0.572 | 0.543 | 0.501 | 0.396 | 0.112 |
| **Iran** | **GDPrHGiniH** | 0.699 | 0.665 | 0.586 | 0.452 | 0.134 |
| **Iran** | **GDPrHGiniHv** | 0.926 | 0.884 | 0.785 | 0.617 | 0.198 |
| **Iran** | **GDPrHGiniLow** | 0.463 | 0.438 | 0.381 | 0.287 | 0.076 |
| **Iran** | **GDPrLGiniH** | 0.699 | 0.665 | 0.647 | 0.538 | 0.163 |
| **Iran** | **GDPrLGiniHv** | 0.926 | 0.884 | 0.861 | 0.724 | 0.237 |
| **Iran** | **GDPrLGiniLow** | 0.463 | 0.438 | 0.425 | 0.347 | 0.094 |
| **Iran** | **NoCOVIDginiB** | 0.637 | 0.662 | 0.654 | 0.495 | 0.131 |
| **Iraq** | **GDPrGiniBase** | 1.925 | 2.038 | 1.863 | 0.64 | 0.029 |
| **Iraq** | **GDPrHGiniH** | 2.191 | 2.315 | 2.045 | 0.7 | 0.034 |
| **Iraq** | **GDPrHGiniHv** | 2.628 | 2.768 | 2.463 | 0.898 | 0.052 |
| **Iraq** | **GDPrHGiniLow** | 1.678 | 1.782 | 1.557 | 0.486 | 0.018 |
| **Iraq** | **GDPrLGiniH** | 2.191 | 2.315 | 2.205 | 0.833 | 0.044 |
| **Iraq** | **GDPrLGiniHv** | 2.628 | 2.768 | 2.645 | 1.058 | 0.067 |
| **Iraq** | **GDPrLGiniLow** | 1.678 | 1.782 | 1.69 | 0.587 | 0.024 |
| **Iraq** | **NoCOVIDginiB** | 0.952 | 0.895 | 0.92 | 0.508 | 0.02 |
| **Ireland** | **GDPrGiniBase** | 0.058 | 0.03 | 0.02 | 0.001 | 0 |
| **Ireland** | **GDPrHGiniH** | 0.076 | 0.041 | 0.026 | 0.002 | 0 |
| **Ireland** | **GDPrHGiniHv** | 0.113 | 0.063 | 0.041 | 0.003 | 0 |
| **Ireland** | **GDPrHGiniLow** | 0.043 | 0.022 | 0.013 | 0.001 | 0 |
| **Ireland** | **GDPrLGiniH** | 0.076 | 0.041 | 0.031 | 0.002 | 0 |
| **Ireland** | **GDPrLGiniHv** | 0.113 | 0.063 | 0.048 | 0.004 | 0 |
| **Ireland** | **GDPrLGiniLow** | 0.043 | 0.022 | 0.016 | 0.001 | 0 |
| **Ireland** | **NoCOVIDginiB** | 0.062 | 0.055 | 0.048 | 0.002 | 0 |
| **Israel** | **GDPrGiniBase** | 0.429 | 0.463 | 0.533 | 0.438 | 0.035 |
| **Israel** | **GDPrHGiniH** | 0.528 | 0.569 | 0.632 | 0.488 | 0.042 |
| **Israel** | **GDPrHGiniHv** | 0.708 | 0.759 | 0.837 | 0.658 | 0.067 |
| **Israel** | **GDPrHGiniLow** | 0.343 | 0.372 | 0.418 | 0.315 | 0.022 |
| **Israel** | **GDPrLGiniH** | 0.528 | 0.569 | 0.672 | 0.592 | 0.054 |
| **Israel** | **GDPrLGiniHv** | 0.708 | 0.759 | 0.887 | 0.788 | 0.083 |
| **Israel** | **GDPrLGiniLow** | 0.343 | 0.372 | 0.447 | 0.389 | 0.028 |
| **Israel** | **NoCOVIDginiB** | 0.381 | 0.461 | 0.539 | 0.456 | 0.036 |
| **Italy** | **GDPrGiniBase** | 1.493 | 1.443 | 1.443 | 0.844 | 0.288 |
| **Italy** | **GDPrHGiniH** | 1.734 | 1.678 | 1.65 | 0.908 | 0.322 |
| **Italy** | **GDPrHGiniHv** | 2.139 | 2.075 | 2.042 | 1.166 | 0.441 |
| **Italy** | **GDPrHGiniLow** | 1.274 | 1.23 | 1.207 | 0.629 | 0.203 |
| **Italy** | **GDPrLGiniH** | 1.734 | 1.678 | 1.707 | 1.117 | 0.403 |
| **Italy** | **GDPrLGiniHv** | 2.139 | 2.075 | 2.109 | 1.416 | 0.544 |
| **Italy** | **GDPrLGiniLow** | 1.274 | 1.23 | 1.253 | 0.788 | 0.259 |
| **Italy** | **NoCOVIDginiB** | 1.325 | 1.302 | 1.263 | 0.858 | 0.289 |
| **Jamaica** | **GDPrGiniBase** | 2.344 | 2.245 | 2.177 | 1.967 | 1.27 |
| **Jamaica** | **GDPrHGiniH** | 2.718 | 2.609 | 2.433 | 2.167 | 1.441 |
| **Jamaica** | **GDPrHGiniHv** | 3.345 | 3.221 | 3.017 | 2.71 | 1.857 |
| **Jamaica** | **GDPrHGiniLow** | 2.005 | 1.917 | 1.776 | 1.563 | 0.994 |
| **Jamaica** | **GDPrLGiniH** | 2.718 | 2.609 | 2.618 | 2.427 | 1.595 |
| **Jamaica** | **GDPrLGiniHv** | 3.345 | 3.221 | 3.23 | 3.01 | 2.04 |
| **Jamaica** | **GDPrLGiniLow** | 2.005 | 1.917 | 1.925 | 1.77 | 1.111 |
| **Jamaica** | **NoCOVIDginiB** | 1.698 | 1.791 | 1.844 | 1.699 | 1.073 |
| **Japan** | **GDPrGiniBase** | 0.761 | 0.736 | 0.706 | 0.534 | 0.268 |
| **Japan** | **GDPrHGiniH** | 0.904 | 0.876 | 0.817 | 0.58 | 0.293 |
| **Japan** | **GDPrHGiniHv** | 1.152 | 1.118 | 1.047 | 0.759 | 0.4 |
| **Japan** | **GDPrHGiniLow** | 0.634 | 0.613 | 0.568 | 0.391 | 0.186 |
| **Japan** | **GDPrLGiniH** | 0.904 | 0.876 | 0.869 | 0.719 | 0.377 |
| **Japan** | **GDPrLGiniHv** | 1.152 | 1.118 | 1.109 | 0.929 | 0.506 |
| **Japan** | **GDPrLGiniLow** | 0.634 | 0.613 | 0.607 | 0.494 | 0.244 |
| **Japan** | **NoCOVIDginiB** | 0.68 | 0.671 | 0.659 | 0.547 | 0.27 |
| **Jordan** | **GDPrGiniBase** | 0.26 | 0.253 | 0.231 | 0.153 | 0.041 |
| **Jordan** | **GDPrHGiniH** | 0.324 | 0.315 | 0.272 | 0.169 | 0.051 |
| **Jordan** | **GDPrHGiniHv** | 0.44 | 0.429 | 0.373 | 0.239 | 0.077 |
| **Jordan** | **GDPrHGiniLow** | 0.206 | 0.201 | 0.17 | 0.101 | 0.027 |
| **Jordan** | **GDPrLGiniH** | 0.324 | 0.315 | 0.304 | 0.22 | 0.061 |
| **Jordan** | **GDPrLGiniHv** | 0.44 | 0.429 | 0.415 | 0.307 | 0.092 |
| **Jordan** | **GDPrLGiniLow** | 0.206 | 0.201 | 0.193 | 0.135 | 0.033 |
| **Jordan** | **NoCOVIDginiB** | 0.221 | 0.213 | 0.199 | 0.126 | 0.035 |
| **Kazakhstan** | **GDPrGiniBase** | 0.118 | 0.115 | 0.102 | 0.016 | 0.002 |
| **Kazakhstan** | **GDPrHGiniH** | 0.149 | 0.147 | 0.123 | 0.019 | 0.002 |
| **Kazakhstan** | **GDPrHGiniHv** | 0.208 | 0.205 | 0.174 | 0.029 | 0.004 |
| **Kazakhstan** | **GDPrHGiniLow** | 0.091 | 0.09 | 0.074 | 0.009 | 0.001 |
| **Kazakhstan** | **GDPrLGiniH** | 0.149 | 0.147 | 0.138 | 0.026 | 0.003 |
| **Kazakhstan** | **GDPrLGiniHv** | 0.208 | 0.205 | 0.193 | 0.041 | 0.005 |
| **Kazakhstan** | **GDPrLGiniLow** | 0.091 | 0.09 | 0.084 | 0.014 | 0.001 |
| **Kazakhstan** | **NoCOVIDginiB** | 0.089 | 0.081 | 0.074 | 0.01 | 0.001 |
| **Kenya** | **GDPrGiniBase** | 32.84 | 31.88 | 30.36 | 21.93 | 5.933 |
| **Kenya** | **GDPrHGiniH** | 33.79 | 32.84 | 30.7 | 22.04 | 5.987 |
| **Kenya** | **GDPrHGiniHv** | 35.2 | 34.27 | 32.16 | 23.55 | 6.96 |
| **Kenya** | **GDPrHGiniLow** | 31.88 | 30.9 | 28.73 | 20.03 | 4.807 |
| **Kenya** | **GDPrLGiniH** | 33.79 | 32.84 | 31.99 | 23.83 | 7.184 |
| **Kenya** | **GDPrLGiniHv** | 35.2 | 34.27 | 33.43 | 25.35 | 8.253 |
| **Kenya** | **GDPrLGiniLow** | 31.88 | 30.9 | 30.04 | 21.81 | 5.868 |
| **Kenya** | **NoCOVIDginiB** | 30.24 | 28.92 | 27.63 | 20 | 4.699 |
| **Korea, Dem. People's Republic** | **GDPrGiniBase** | 31.19 | 30.75 | 30.48 | 25.66 | 12.15 |
| **Korea, Dem. People's Republic** | **GDPrHGiniH** | 32.13 | 31.69 | 30.85 | 25.85 | 12.39 |
| **Korea, Dem. People's Republic** | **GDPrHGiniHv** | 33.5 | 33.07 | 32.24 | 27.29 | 13.66 |
| **Korea, Dem. People's Republic** | **GDPrHGiniLow** | 30.25 | 29.81 | 28.95 | 23.9 | 10.73 |
| **Korea, Dem. People's Republic** | **GDPrLGiniH** | 32.13 | 31.69 | 32.01 | 27.47 | 13.72 |
| **Korea, Dem. People's Republic** | **GDPrLGiniHv** | 33.5 | 33.07 | 33.39 | 28.9 | 15.05 |
| **Korea, Dem. People's Republic** | **GDPrLGiniLow** | 30.25 | 29.81 | 30.14 | 25.53 | 11.99 |
| **Korea, Dem. People's Republic** | **NoCOVIDginiB** | 31.13 | 30.76 | 30.5 | 25.42 | 11.97 |
| **Korea, Republic of** | **GDPrGiniBase** | 0.204 | 0.187 | 0.188 | 0.064 | 0.002 |
| **Korea, Republic of** | **GDPrHGiniH** | 0.256 | 0.235 | 0.22 | 0.073 | 0.003 |
| **Korea, Republic of** | **GDPrHGiniHv** | 0.35 | 0.323 | 0.304 | 0.107 | 0.005 |
| **Korea, Republic of** | **GDPrHGiniLow** | 0.161 | 0.147 | 0.137 | 0.041 | 0.001 |
| **Korea, Republic of** | **GDPrLGiniH** | 0.256 | 0.235 | 0.254 | 0.099 | 0.004 |
| **Korea, Republic of** | **GDPrLGiniHv** | 0.35 | 0.323 | 0.348 | 0.143 | 0.007 |
| **Korea, Republic of** | **GDPrLGiniLow** | 0.161 | 0.147 | 0.16 | 0.057 | 0.002 |
| **Korea, Republic of** | **NoCOVIDginiB** | 0.175 | 0.169 | 0.165 | 0.055 | 0.002 |
| **Kosovo** | **GDPrGiniBase** | 1.013 | 0.799 | 0.721 | 1.469 | 0.053 |
| **Kosovo** | **GDPrHGiniH** | 1.182 | 0.941 | 0.79 | 1.461 | 0.059 |
| **Kosovo** | **GDPrHGiniHv** | 1.468 | 1.185 | 1.004 | 1.791 | 0.088 |
| **Kosovo** | **GDPrHGiniLow** | 0.86 | 0.672 | 0.555 | 1.084 | 0.033 |
| **Kosovo** | **GDPrLGiniH** | 1.182 | 0.941 | 0.922 | 1.98 | 0.081 |
| **Kosovo** | **GDPrLGiniHv** | 1.468 | 1.185 | 1.162 | 2.385 | 0.118 |
| **Kosovo** | **GDPrLGiniLow** | 0.86 | 0.672 | 0.657 | 1.508 | 0.046 |
| **Kosovo** | **NoCOVIDginiB** | 0.636 | 0.526 | 0.508 | 1.079 | 0.037 |
| **Kuwait** | **GDPrGiniBase** | 0.13 | 0.167 | 0.153 | 0.06 | 0.068 |
| **Kuwait** | **GDPrHGiniH** | 0.168 | 0.213 | 0.19 | 0.072 | 0.077 |
| **Kuwait** | **GDPrHGiniHv** | 0.24 | 0.301 | 0.27 | 0.11 | 0.116 |
| **Kuwait** | **GDPrHGiniLow** | 0.099 | 0.128 | 0.113 | 0.04 | 0.042 |
| **Kuwait** | **GDPrLGiniH** | 0.168 | 0.213 | 0.199 | 0.088 | 0.102 |
| **Kuwait** | **GDPrLGiniHv** | 0.24 | 0.301 | 0.282 | 0.131 | 0.151 |
| **Kuwait** | **GDPrLGiniLow** | 0.099 | 0.128 | 0.119 | 0.049 | 0.058 |
| **Kuwait** | **NoCOVIDginiB** | 0.103 | 0.1 | 0.097 | 0.051 | 0.041 |
| **Kyrgyzstan** | **GDPrGiniBase** | 1.898 | 2.354 | 2.412 | 2.687 | 1.195 |
| **Kyrgyzstan** | **GDPrHGiniH** | 2.156 | 2.653 | 2.47 | 2.514 | 1.221 |
| **Kyrgyzstan** | **GDPrHGiniHv** | 2.579 | 3.139 | 2.931 | 2.97 | 1.513 |
| **Kyrgyzstan** | **GDPrHGiniLow** | 1.659 | 2.074 | 1.924 | 1.97 | 0.891 |
| **Kyrgyzstan** | **GDPrLGiniH** | 2.156 | 2.653 | 2.935 | 3.578 | 1.573 |
| **Kyrgyzstan** | **GDPrLGiniHv** | 2.579 | 3.139 | 3.448 | 4.146 | 1.922 |
| **Kyrgyzstan** | **GDPrLGiniLow** | 1.659 | 2.074 | 2.318 | 2.887 | 1.173 |
| **Kyrgyzstan** | **NoCOVIDginiB** | 0.928 | 1.007 | 1.091 | 1.245 | 0.727 |
| **Lao People's Dem. Republic** | **GDPrGiniBase** | 8.478 | 8.569 | 8.18 | 4.245 | 0.246 |
| **Lao People's Dem. Republic** | **GDPrHGiniH** | 9.172 | 9.268 | 8.559 | 4.404 | 0.278 |
| **Lao People's Dem. Republic** | **GDPrHGiniHv** | 10.25 | 10.35 | 9.602 | 5.141 | 0.386 |
| **Lao People's Dem. Republic** | **GDPrHGiniLow** | 7.804 | 7.891 | 7.239 | 3.513 | 0.172 |
| **Lao People's Dem. Republic** | **GDPrLGiniH** | 9.172 | 9.268 | 9.182 | 5.088 | 0.342 |
| **Lao People's Dem. Republic** | **GDPrLGiniHv** | 10.25 | 10.35 | 10.26 | 5.887 | 0.467 |
| **Lao People's Dem. Republic** | **GDPrLGiniLow** | 7.804 | 7.891 | 7.814 | 4.111 | 0.216 |
| **Lao People's Dem. Republic** | **NoCOVIDginiB** | 7.243 | 6.59 | 5.996 | 2.717 | 0.132 |
| **Latvia** | **GDPrGiniBase** | 0.55 | 0.554 | 0.544 | 0.239 | 0.022 |
| **Latvia** | **GDPrHGiniH** | 0.666 | 0.67 | 0.618 | 0.262 | 0.025 |
| **Latvia** | **GDPrHGiniHv** | 0.871 | 0.876 | 0.812 | 0.362 | 0.041 |
| **Latvia** | **GDPrHGiniLow** | 0.449 | 0.453 | 0.414 | 0.162 | 0.013 |
| **Latvia** | **GDPrLGiniH** | 0.666 | 0.67 | 0.7 | 0.341 | 0.036 |
| **Latvia** | **GDPrLGiniHv** | 0.871 | 0.876 | 0.912 | 0.465 | 0.056 |
| **Latvia** | **GDPrLGiniLow** | 0.449 | 0.453 | 0.474 | 0.216 | 0.018 |
| **Latvia** | **NoCOVIDginiB** | 0.431 | 0.452 | 0.494 | 0.227 | 0.021 |
| **Lebanon** | **GDPrGiniBase** | 12.78 | 13.53 | 13.77 | 19.75 | 4.552 |
| **Lebanon** | **GDPrHGiniH** | 13.54 | 14.31 | 14.16 | 19.82 | 4.649 |
| **Lebanon** | **GDPrHGiniHv** | 14.7 | 15.48 | 15.33 | 21.07 | 5.374 |
| **Lebanon** | **GDPrHGiniLow** | 12.02 | 12.76 | 12.62 | 18.14 | 3.742 |
| **Lebanon** | **GDPrLGiniH** | 13.54 | 14.31 | 14.96 | 21.29 | 5.532 |
| **Lebanon** | **GDPrLGiniHv** | 14.7 | 15.48 | 16.14 | 22.55 | 6.337 |
| **Lebanon** | **GDPrLGiniLow** | 12.02 | 12.76 | 13.38 | 19.6 | 4.526 |
| **Lebanon** | **NoCOVIDginiB** | 6.203 | 6.334 | 5.884 | 8.269 | 2.291 |
| **Lesotho** | **GDPrGiniBase** | 33.28 | 34.06 | 35.47 | 34.95 | 12.88 |
| **Lesotho** | **GDPrHGiniH** | 34.32 | 35.1 | 35.6 | 34.23 | 13.21 |
| **Lesotho** | **GDPrHGiniHv** | 35.87 | 36.64 | 37.11 | 35.74 | 14.8 |
| **Lesotho** | **GDPrHGiniLow** | 32.23 | 33.01 | 33.55 | 32.18 | 11.19 |
| **Lesotho** | **GDPrLGiniH** | 34.32 | 35.1 | 37.09 | 37.43 | 14.63 |
| **Lesotho** | **GDPrLGiniHv** | 35.87 | 36.64 | 38.57 | 38.86 | 16.24 |
| **Lesotho** | **GDPrLGiniLow** | 32.23 | 33.01 | 35.08 | 35.5 | 12.57 |
| **Lesotho** | **NoCOVIDginiB** | 28.13 | 28.33 | 27.89 | 28.74 | 9.926 |
| **Liberia** | **GDPrGiniBase** | 52.97 | 52.24 | 50.55 | 58.76 | 22.39 |
| **Liberia** | **GDPrHGiniH** | 53.45 | 52.77 | 50.37 | 56.4 | 20.8 |
| **Liberia** | **GDPrHGiniHv** | 54.17 | 53.53 | 51.17 | 56.93 | 22.53 |
| **Liberia** | **GDPrHGiniLow** | 52.47 | 51.72 | 49.27 | 55.71 | 18.5 |
| **Liberia** | **GDPrLGiniH** | 53.45 | 52.77 | 51.82 | 61.33 | 26.85 |
| **Liberia** | **GDPrLGiniHv** | 54.17 | 53.53 | 52.58 | 61.67 | 28.57 |
| **Liberia** | **GDPrLGiniLow** | 52.47 | 51.72 | 50.79 | 60.91 | 24.5 |
| **Liberia** | **NoCOVIDginiB** | 50.02 | 51.24 | 53.36 | 64.09 | 29.26 |
| **Libya** | **GDPrGiniBase** | 4.625 | 1.068 | 0.573 | 0.065 | 0.012 |
| **Libya** | **GDPrHGiniH** | 5.123 | 1.257 | 0.672 | 0.07 | 0.015 |
| **Libya** | **GDPrHGiniHv** | 5.914 | 1.579 | 0.877 | 0.105 | 0.025 |
| **Libya** | **GDPrHGiniLow** | 4.153 | 0.899 | 0.454 | 0.039 | 0.007 |
| **Libya** | **GDPrLGiniH** | 5.123 | 1.257 | 0.714 | 0.108 | 0.019 |
| **Libya** | **GDPrLGiniHv** | 5.914 | 1.579 | 0.929 | 0.157 | 0.031 |
| **Libya** | **GDPrLGiniLow** | 4.153 | 0.899 | 0.486 | 0.062 | 0.009 |
| **Libya** | **NoCOVIDginiB** | 2.392 | 2.581 | 2.714 | 1.038 | 0.073 |
| **Lithuania** | **GDPrGiniBase** | 0.67 | 0.61 | 0.556 | 0.305 | 0.028 |
| **Lithuania** | **GDPrHGiniH** | 0.808 | 0.737 | 0.639 | 0.338 | 0.033 |
| **Lithuania** | **GDPrHGiniHv** | 1.048 | 0.963 | 0.842 | 0.464 | 0.053 |
| **Lithuania** | **GDPrHGiniLow** | 0.551 | 0.499 | 0.426 | 0.213 | 0.017 |
| **Lithuania** | **GDPrLGiniH** | 0.808 | 0.737 | 0.713 | 0.426 | 0.045 |
| **Lithuania** | **GDPrLGiniHv** | 1.048 | 0.963 | 0.932 | 0.575 | 0.07 |
| **Lithuania** | **GDPrLGiniLow** | 0.551 | 0.499 | 0.48 | 0.272 | 0.023 |
| **Lithuania** | **NoCOVIDginiB** | 0.62 | 0.597 | 0.593 | 0.346 | 0.031 |
| **Luxembourg** | **GDPrGiniBase** | 0.244 | 0.217 | 0.182 | 0.048 | 0.01 |
| **Luxembourg** | **GDPrHGiniH** | 0.305 | 0.273 | 0.225 | 0.059 | 0.012 |
| **Luxembourg** | **GDPrHGiniHv** | 0.418 | 0.376 | 0.313 | 0.089 | 0.021 |
| **Luxembourg** | **GDPrHGiniLow** | 0.193 | 0.17 | 0.138 | 0.032 | 0.006 |
| **Luxembourg** | **GDPrLGiniH** | 0.305 | 0.273 | 0.227 | 0.069 | 0.015 |
| **Luxembourg** | **GDPrLGiniHv** | 0.418 | 0.376 | 0.317 | 0.103 | 0.025 |
| **Luxembourg** | **GDPrLGiniLow** | 0.193 | 0.17 | 0.14 | 0.038 | 0.007 |
| **Luxembourg** | **NoCOVIDginiB** | 0.224 | 0.203 | 0.175 | 0.053 | 0.01 |
| **Macedonia, North** | **GDPrGiniBase** | 4.796 | 4.855 | 4.883 | 4.134 | 1.31 |
| **Macedonia, North** | **GDPrHGiniH** | 5.295 | 5.359 | 5.161 | 4.213 | 1.368 |
| **Macedonia, North** | **GDPrHGiniHv** | 6.088 | 6.158 | 5.942 | 4.906 | 1.714 |
| **Macedonia, North** | **GDPrHGiniLow** | 4.319 | 4.375 | 4.202 | 3.37 | 0.988 |
| **Macedonia, North** | **GDPrLGiniH** | 5.295 | 5.359 | 5.622 | 5.007 | 1.716 |
| **Macedonia, North** | **GDPrLGiniHv** | 6.088 | 6.158 | 6.44 | 5.772 | 2.113 |
| **Macedonia, North** | **GDPrLGiniLow** | 4.319 | 4.375 | 4.611 | 4.066 | 1.264 |
| **Macedonia, North** | **NoCOVIDginiB** | 3.608 | 3.692 | 3.881 | 3.415 | 1.034 |
| **Madagascar** | **GDPrGiniBase** | 78.51 | 78.76 | 77.71 | 70.14 | 60.55 |
| **Madagascar** | **GDPrHGiniH** | 78.52 | 78.77 | 77.18 | 69.22 | 58.54 |
| **Madagascar** | **GDPrHGiniHv** | 78.56 | 78.81 | 77.25 | 69.59 | 59.47 |
| **Madagascar** | **GDPrHGiniLow** | 78.52 | 78.76 | 77.12 | 68.77 | 57.21 |
| **Madagascar** | **GDPrLGiniH** | 78.52 | 78.77 | 78.29 | 71.44 | 63.78 |
| **Madagascar** | **GDPrLGiniHv** | 78.56 | 78.81 | 78.32 | 71.73 | 64.47 |
| **Madagascar** | **GDPrLGiniLow** | 78.52 | 78.76 | 78.28 | 71.1 | 62.73 |
| **Madagascar** | **NoCOVIDginiB** | 74.01 | 73.01 | 72.01 | 63.95 | 46.24 |
| **Malawi** | **GDPrGiniBase** | 68.85 | 69.3 | 68.71 | 56.16 | 19.57 |
| **Malawi** | **GDPrHGiniH** | 69.13 | 69.58 | 68 | 55.22 | 18.87 |
| **Malawi** | **GDPrHGiniHv** | 69.56 | 70 | 68.45 | 56.07 | 20.65 |
| **Malawi** | **GDPrHGiniLow** | 68.58 | 69.02 | 67.41 | 54.09 | 16.54 |
| **Malawi** | **GDPrLGiniH** | 69.13 | 69.58 | 69.98 | 58.1 | 22.98 |
| **Malawi** | **GDPrLGiniHv** | 69.56 | 70 | 70.36 | 58.83 | 24.85 |
| **Malawi** | **GDPrLGiniLow** | 68.58 | 69.02 | 69.51 | 57.12 | 20.5 |
| **Malawi** | **NoCOVIDginiB** | 66.38 | 65.34 | 64.04 | 48.68 | 13 |
| **Malaysia** | **GDPrGiniBase** | 0.133 | 0.137 | 0.137 | 0.071 | 0.02 |
| **Malaysia** | **GDPrHGiniH** | 0.174 | 0.178 | 0.172 | 0.087 | 0.025 |
| **Malaysia** | **GDPrHGiniHv** | 0.252 | 0.258 | 0.25 | 0.131 | 0.041 |
| **Malaysia** | **GDPrHGiniLow** | 0.101 | 0.104 | 0.1 | 0.047 | 0.012 |
| **Malaysia** | **GDPrLGiniH** | 0.174 | 0.178 | 0.186 | 0.104 | 0.032 |
| **Malaysia** | **GDPrLGiniHv** | 0.252 | 0.258 | 0.268 | 0.155 | 0.051 |
| **Malaysia** | **GDPrLGiniLow** | 0.101 | 0.104 | 0.108 | 0.057 | 0.015 |
| **Malaysia** | **NoCOVIDginiB** | 0.1 | 0.093 | 0.088 | 0.052 | 0.014 |
| **Maldives** | **GDPrGiniBase** | 12.36 | 9.072 | 8.125 | 4.833 | 3.703 |
| **Maldives** | **GDPrHGiniH** | 13.11 | 9.737 | 8.432 | 4.943 | 3.747 |
| **Maldives** | **GDPrHGiniHv** | 14.24 | 10.76 | 9.391 | 5.678 | 4.375 |
| **Maldives** | **GDPrHGiniLow** | 11.62 | 8.422 | 7.207 | 4.038 | 2.988 |
| **Maldives** | **GDPrLGiniH** | 13.11 | 9.737 | 9.016 | 5.607 | 4.462 |
| **Maldives** | **GDPrLGiniHv** | 14.24 | 10.76 | 10 | 6.392 | 5.156 |
| **Maldives** | **GDPrLGiniLow** | 11.62 | 8.422 | 7.75 | 4.631 | 3.612 |
| **Maldives** | **NoCOVIDginiB** | 3.287 | 3.325 | 3.362 | 2.805 | 1.766 |
| **Mali** | **GDPrGiniBase** | 45.73 | 46.62 | 45.95 | 37.82 | 14.02 |
| **Mali** | **GDPrHGiniH** | 46.32 | 47.21 | 45.45 | 36.6 | 13.38 |
| **Mali** | **GDPrHGiniHv** | 47.19 | 48.07 | 46.33 | 37.67 | 14.71 |
| **Mali** | **GDPrHGiniLow** | 45.12 | 46.02 | 44.24 | 35.13 | 11.65 |
| **Mali** | **GDPrLGiniH** | 46.32 | 47.21 | 47.65 | 40.51 | 16.74 |
| **Mali** | **GDPrLGiniHv** | 47.19 | 48.07 | 48.46 | 41.48 | 18.15 |
| **Mali** | **GDPrLGiniLow** | 45.12 | 46.02 | 46.53 | 39.17 | 14.84 |
| **Mali** | **NoCOVIDginiB** | 41.31 | 41.25 | 41.41 | 34.09 | 10.64 |
| **Malta** | **GDPrGiniBase** | 0.138 | 0.132 | 0.13 | 0.018 | 0 |
| **Malta** | **GDPrHGiniH** | 0.174 | 0.167 | 0.152 | 0.022 | 0.001 |
| **Malta** | **GDPrHGiniHv** | 0.243 | 0.233 | 0.213 | 0.034 | 0.001 |
| **Malta** | **GDPrHGiniLow** | 0.107 | 0.103 | 0.093 | 0.011 | 0 |
| **Malta** | **GDPrLGiniH** | 0.174 | 0.167 | 0.178 | 0.027 | 0.001 |
| **Malta** | **GDPrLGiniHv** | 0.243 | 0.233 | 0.247 | 0.043 | 0.002 |
| **Malta** | **GDPrLGiniLow** | 0.107 | 0.103 | 0.11 | 0.014 | 0 |
| **Malta** | **NoCOVIDginiB** | 0.073 | 0.075 | 0.078 | 0.014 | 0 |
| **Mauritania** | **GDPrGiniBase** | 6.476 | 6.863 | 7.177 | 8.219 | 2.065 |
| **Mauritania** | **GDPrHGiniH** | 7.052 | 7.459 | 7.398 | 8.01 | 2.047 |
| **Mauritania** | **GDPrHGiniHv** | 7.951 | 8.386 | 8.317 | 8.96 | 2.499 |
| **Mauritania** | **GDPrHGiniLow** | 5.919 | 6.288 | 6.236 | 6.803 | 1.529 |
| **Mauritania** | **GDPrLGiniH** | 7.052 | 7.459 | 8.197 | 9.866 | 2.727 |
| **Mauritania** | **GDPrLGiniHv** | 7.951 | 8.386 | 9.161 | 10.9 | 3.271 |
| **Mauritania** | **GDPrLGiniLow** | 5.919 | 6.288 | 6.971 | 8.53 | 2.089 |
| **Mauritania** | **NoCOVIDginiB** | 4.926 | 4.642 | 4.141 | 4.995 | 0.987 |
| **Mauritius** | **GDPrGiniBase** | 0.388 | 0.376 | 0.365 | 0.642 | 0.316 |
| **Mauritius** | **GDPrHGiniH** | 0.478 | 0.464 | 0.427 | 0.689 | 0.359 |
| **Mauritius** | **GDPrHGiniHv** | 0.64 | 0.622 | 0.576 | 0.902 | 0.49 |
| **Mauritius** | **GDPrHGiniLow** | 0.311 | 0.301 | 0.275 | 0.463 | 0.227 |
| **Mauritius** | **GDPrLGiniH** | 0.478 | 0.464 | 0.476 | 0.863 | 0.428 |
| **Mauritius** | **GDPrLGiniHv** | 0.64 | 0.622 | 0.637 | 1.115 | 0.577 |
| **Mauritius** | **GDPrLGiniLow** | 0.311 | 0.301 | 0.31 | 0.593 | 0.275 |
| **Mauritius** | **NoCOVIDginiB** | 0.178 | 0.185 | 0.199 | 0.385 | 0.166 |
| **Mexico** | **GDPrGiniBase** | 2.341 | 2.217 | 2.148 | 1.814 | 1.533 |
| **Mexico** | **GDPrHGiniH** | 2.714 | 2.577 | 2.44 | 2.013 | 1.706 |
| **Mexico** | **GDPrHGiniHv** | 3.34 | 3.182 | 3.024 | 2.529 | 2.17 |
| **Mexico** | **GDPrHGiniLow** | 2.003 | 1.892 | 1.781 | 1.441 | 1.2 |
| **Mexico** | **GDPrLGiniH** | 2.714 | 2.577 | 2.562 | 2.249 | 1.932 |
| **Mexico** | **GDPrLGiniHv** | 3.34 | 3.182 | 3.165 | 2.805 | 2.437 |
| **Mexico** | **GDPrLGiniLow** | 2.003 | 1.892 | 1.879 | 1.628 | 1.375 |
| **Mexico** | **NoCOVIDginiB** | 1.95 | 1.932 | 1.87 | 1.621 | 1.335 |
| **Micronesia** | **GDPrGiniBase** | 15.01 | 15.47 | 15.35 | 11.79 | 13.15 |
| **Micronesia** | **GDPrHGiniH** | 15.93 | 16.4 | 15.84 | 12.31 | 12.88 |
| **Micronesia** | **GDPrHGiniHv** | 17.32 | 17.8 | 17.22 | 13.6 | 14.24 |
| **Micronesia** | **GDPrHGiniLow** | 14.11 | 14.56 | 14.02 | 10.65 | 11.12 |
| **Micronesia** | **GDPrLGiniH** | 15.93 | 16.4 | 16.69 | 12.95 | 15.8 |
| **Micronesia** | **GDPrLGiniHv** | 17.32 | 17.8 | 18.1 | 14.26 | 17.26 |
| **Micronesia** | **GDPrLGiniLow** | 14.11 | 14.56 | 14.84 | 11.25 | 13.9 |
| **Micronesia** | **NoCOVIDginiB** | 14.3 | 13.68 | 13.34 | 10.71 | 14.26 |
| **Moldova, Republic of** | **GDPrGiniBase** | 0.17 | 0.18 | 0.178 | 0.189 | 0.015 |
| **Moldova, Republic of** | **GDPrHGiniH** | 0.211 | 0.224 | 0.198 | 0.194 | 0.018 |
| **Moldova, Republic of** | **GDPrHGiniHv** | 0.288 | 0.304 | 0.271 | 0.265 | 0.028 |
| **Moldova, Republic of** | **GDPrHGiniLow** | 0.134 | 0.143 | 0.125 | 0.123 | 0.009 |
| **Moldova, Republic of** | **GDPrLGiniH** | 0.211 | 0.224 | 0.242 | 0.282 | 0.024 |
| **Moldova, Republic of** | **GDPrLGiniHv** | 0.288 | 0.304 | 0.327 | 0.377 | 0.037 |
| **Moldova, Republic of** | **GDPrLGiniLow** | 0.134 | 0.143 | 0.156 | 0.184 | 0.013 |
| **Moldova, Republic of** | **NoCOVIDginiB** | 0.073 | 0.084 | 0.1 | 0.123 | 0.01 |
| **Mongolia** | **GDPrGiniBase** | 0.484 | 0.599 | 0.632 | 0.06 | 0 |
| **Mongolia** | **GDPrHGiniH** | 0.585 | 0.718 | 0.706 | 0.067 | 0 |
| **Mongolia** | **GDPrHGiniHv** | 0.765 | 0.928 | 0.912 | 0.099 | 0.001 |
| **Mongolia** | **GDPrHGiniLow** | 0.395 | 0.494 | 0.485 | 0.037 | 0 |
| **Mongolia** | **GDPrLGiniH** | 0.585 | 0.718 | 0.813 | 0.095 | 0 |
| **Mongolia** | **GDPrLGiniHv** | 0.765 | 0.928 | 1.041 | 0.139 | 0.001 |
| **Mongolia** | **GDPrLGiniLow** | 0.395 | 0.494 | 0.565 | 0.055 | 0 |
| **Mongolia** | **NoCOVIDginiB** | 0.288 | 0.325 | 0.376 | 0.037 | 0 |
| **Montenegro** | **GDPrGiniBase** | 1.67 | 1.618 | 1.576 | 1.67 | 0.539 |
| **Montenegro** | **GDPrHGiniH** | 1.941 | 1.884 | 1.746 | 1.705 | 0.617 |
| **Montenegro** | **GDPrHGiniHv** | 2.397 | 2.331 | 2.171 | 2.123 | 0.82 |
| **Montenegro** | **GDPrHGiniLow** | 1.424 | 1.378 | 1.269 | 1.237 | 0.406 |
| **Montenegro** | **GDPrLGiniH** | 1.941 | 1.884 | 1.932 | 2.274 | 0.685 |
| **Montenegro** | **GDPrLGiniHv** | 2.397 | 2.331 | 2.387 | 2.781 | 0.905 |
| **Montenegro** | **GDPrLGiniLow** | 1.424 | 1.378 | 1.418 | 1.694 | 0.455 |
| **Montenegro** | **NoCOVIDginiB** | 0.833 | 0.901 | 1.026 | 1.06 | 0.377 |
| **Morocco** | **GDPrGiniBase** | 0.75 | 0.727 | 0.751 | 0.41 | 0.133 |
| **Morocco** | **GDPrHGiniH** | 0.903 | 0.878 | 0.867 | 0.457 | 0.157 |
| **Morocco** | **GDPrHGiniHv** | 1.172 | 1.142 | 1.128 | 0.618 | 0.229 |
| **Morocco** | **GDPrHGiniLow** | 0.616 | 0.597 | 0.589 | 0.293 | 0.091 |
| **Morocco** | **GDPrLGiniH** | 0.903 | 0.878 | 0.938 | 0.562 | 0.191 |
| **Morocco** | **GDPrLGiniHv** | 1.172 | 1.142 | 1.215 | 0.75 | 0.274 |
| **Morocco** | **GDPrLGiniLow** | 0.616 | 0.597 | 0.642 | 0.367 | 0.112 |
| **Morocco** | **NoCOVIDginiB** | 0.551 | 0.521 | 0.484 | 0.278 | 0.093 |
| **Mozambique** | **GDPrGiniBase** | 64.12 | 65.4 | 64.71 | 57.41 | 26.7 |
| **Mozambique** | **GDPrHGiniH** | 64.73 | 66 | 64.54 | 56.55 | 26.98 |
| **Mozambique** | **GDPrHGiniHv** | 65.65 | 66.88 | 65.46 | 57.78 | 29.22 |
| **Mozambique** | **GDPrHGiniLow** | 63.51 | 64.81 | 63.32 | 54.9 | 24.03 |
| **Mozambique** | **GDPrLGiniH** | 64.73 | 66 | 65.98 | 60.01 | 29.55 |
| **Mozambique** | **GDPrLGiniHv** | 65.65 | 66.88 | 66.83 | 61.11 | 31.78 |
| **Mozambique** | **GDPrLGiniLow** | 63.51 | 64.81 | 64.84 | 58.53 | 26.57 |
| **Mozambique** | **NoCOVIDginiB** | 61.1 | 61.21 | 61.33 | 54.02 | 25.52 |
| **Myanmar** | **GDPrGiniBase** | 0.684 | 1.503 | 1.983 | 0.636 | 0.069 |
| **Myanmar** | **GDPrHGiniH** | 0.814 | 1.732 | 2.126 | 0.652 | 0.077 |
| **Myanmar** | **GDPrHGiniHv** | 1.037 | 2.111 | 2.56 | 0.842 | 0.113 |
| **Myanmar** | **GDPrHGiniLow** | 0.57 | 1.295 | 1.619 | 0.448 | 0.043 |
| **Myanmar** | **GDPrLGiniH** | 0.814 | 1.732 | 2.387 | 0.876 | 0.105 |
| **Myanmar** | **GDPrLGiniHv** | 1.037 | 2.111 | 2.855 | 1.111 | 0.151 |
| **Myanmar** | **GDPrLGiniLow** | 0.57 | 1.295 | 1.836 | 0.618 | 0.061 |
| **Myanmar** | **NoCOVIDginiB** | 0.582 | 0.413 | 0.32 | 0.072 | 0.006 |
| **Namibia** | **GDPrGiniBase** | 18.1 | 19.19 | 19.74 | 20.33 | 6.693 |
| **Namibia** | **GDPrHGiniH** | 19.5 | 20.62 | 20.85 | 20.88 | 7.377 |
| **Namibia** | **GDPrHGiniHv** | 21.67 | 22.81 | 23.05 | 23.1 | 8.869 |
| **Namibia** | **GDPrHGiniLow** | 16.74 | 17.81 | 18.03 | 18.05 | 5.641 |
| **Namibia** | **GDPrLGiniH** | 19.5 | 20.62 | 21.51 | 22.69 | 7.884 |
| **Namibia** | **GDPrLGiniHv** | 21.67 | 22.81 | 23.73 | 24.93 | 9.428 |
| **Namibia** | **GDPrLGiniLow** | 16.74 | 17.81 | 18.67 | 19.79 | 6.076 |
| **Namibia** | **NoCOVIDginiB** | 16.01 | 16.67 | 17.11 | 17.62 | 5.564 |
| **Nepal** | **GDPrGiniBase** | 5.072 | 5.598 | 5.814 | 4.051 | 1.914 |
| **Nepal** | **GDPrHGiniH** | 5.579 | 6.136 | 5.965 | 4.031 | 1.883 |
| **Nepal** | **GDPrHGiniHv** | 6.378 | 6.98 | 6.792 | 4.687 | 2.31 |
| **Nepal** | **GDPrHGiniLow** | 4.588 | 5.083 | 4.937 | 3.235 | 1.395 |
| **Nepal** | **GDPrLGiniH** | 5.579 | 6.136 | 6.644 | 4.937 | 2.556 |
| **Nepal** | **GDPrLGiniHv** | 6.378 | 6.98 | 7.516 | 5.672 | 3.077 |
| **Nepal** | **GDPrLGiniLow** | 4.588 | 5.083 | 5.549 | 4.032 | 1.946 |
| **Nepal** | **NoCOVIDginiB** | 3.585 | 3.36 | 3.355 | 2.63 | 0.994 |
| **Netherlands** | **GDPrGiniBase** | 0.187 | 0.163 | 0.129 | 0.05 | 0.007 |
| **Netherlands** | **GDPrHGiniH** | 0.234 | 0.205 | 0.157 | 0.056 | 0.009 |
| **Netherlands** | **GDPrHGiniHv** | 0.319 | 0.281 | 0.22 | 0.084 | 0.015 |
| **Netherlands** | **GDPrHGiniLow** | 0.148 | 0.128 | 0.096 | 0.032 | 0.004 |
| **Netherlands** | **GDPrLGiniH** | 0.234 | 0.205 | 0.171 | 0.078 | 0.012 |
| **Netherlands** | **GDPrLGiniHv** | 0.319 | 0.281 | 0.237 | 0.113 | 0.019 |
| **Netherlands** | **GDPrLGiniLow** | 0.148 | 0.128 | 0.105 | 0.045 | 0.006 |
| **Netherlands** | **NoCOVIDginiB** | 0.16 | 0.149 | 0.127 | 0.055 | 0.007 |
| **New Zealand** | **GDPrGiniBase** | 0.079 | 0.068 | 0.06 | 0.03 | 0.006 |
| **New Zealand** | **GDPrHGiniH** | 0.104 | 0.091 | 0.075 | 0.037 | 0.008 |
| **New Zealand** | **GDPrHGiniHv** | 0.153 | 0.134 | 0.113 | 0.058 | 0.014 |
| **New Zealand** | **GDPrHGiniLow** | 0.059 | 0.051 | 0.041 | 0.019 | 0.004 |
| **New Zealand** | **GDPrLGiniH** | 0.104 | 0.091 | 0.086 | 0.047 | 0.01 |
| **New Zealand** | **GDPrLGiniHv** | 0.153 | 0.134 | 0.128 | 0.073 | 0.018 |
| **New Zealand** | **GDPrLGiniLow** | 0.059 | 0.051 | 0.048 | 0.025 | 0.005 |
| **New Zealand** | **NoCOVIDginiB** | 0.077 | 0.073 | 0.068 | 0.032 | 0.007 |
| **Nicaragua** | **GDPrGiniBase** | 3.103 | 2.94 | 2.668 | 2.3 | 1.314 |
| **Nicaragua** | **GDPrHGiniH** | 3.557 | 3.379 | 2.955 | 2.431 | 1.436 |
| **Nicaragua** | **GDPrHGiniHv** | 4.31 | 4.109 | 3.624 | 3.018 | 1.853 |
| **Nicaragua** | **GDPrHGiniLow** | 2.686 | 2.538 | 2.191 | 1.771 | 0.987 |
| **Nicaragua** | **GDPrLGiniH** | 3.557 | 3.379 | 3.195 | 2.937 | 1.717 |
| **Nicaragua** | **GDPrLGiniHv** | 4.31 | 4.109 | 3.899 | 3.6 | 2.19 |
| **Nicaragua** | **GDPrLGiniLow** | 2.686 | 2.538 | 2.388 | 2.18 | 1.202 |
| **Nicaragua** | **NoCOVIDginiB** | 2.976 | 3.147 | 3.208 | 3.012 | 1.764 |
| **Niger** | **GDPrGiniBase** | 41.17 | 41.22 | 39.28 | 27.84 | 4.097 |
| **Niger** | **GDPrHGiniH** | 41.87 | 41.94 | 38.9 | 26.86 | 3.982 |
| **Niger** | **GDPrHGiniHv** | 42.9 | 42.99 | 40 | 28.12 | 4.707 |
| **Niger** | **GDPrHGiniLow** | 40.45 | 40.49 | 37.38 | 25.16 | 3.083 |
| **Niger** | **GDPrLGiniH** | 41.87 | 41.94 | 41.18 | 30.71 | 5.307 |
| **Niger** | **GDPrLGiniHv** | 42.9 | 42.99 | 42.23 | 31.93 | 6.168 |
| **Niger** | **GDPrLGiniLow** | 40.45 | 40.49 | 39.74 | 29.05 | 4.229 |
| **Niger** | **NoCOVIDginiB** | 38.28 | 38.21 | 34.73 | 25.16 | 3.332 |
| **Nigeria** | **GDPrGiniBase** | 44.18 | 44.05 | 43.89 | 38.3 | 25.32 |
| **Nigeria** | **GDPrHGiniH** | 45.01 | 44.88 | 44.14 | 38.35 | 25.25 |
| **Nigeria** | **GDPrHGiniHv** | 46.23 | 46.11 | 45.38 | 39.75 | 26.88 |
| **Nigeria** | **GDPrHGiniLow** | 43.33 | 43.2 | 42.43 | 36.44 | 23.07 |
| **Nigeria** | **GDPrLGiniH** | 45.01 | 44.88 | 45.33 | 40.09 | 27.56 |
| **Nigeria** | **GDPrLGiniHv** | 46.23 | 46.11 | 46.54 | 41.45 | 29.18 |
| **Nigeria** | **GDPrLGiniLow** | 43.33 | 43.2 | 43.67 | 38.24 | 25.37 |
| **Nigeria** | **NoCOVIDginiB** | 42.3 | 42.57 | 42.58 | 37.3 | 23.74 |
| **Norway** | **GDPrGiniBase** | 0.202 | 0.211 | 0.21 | 0.077 | 0.014 |
| **Norway** | **GDPrHGiniH** | 0.251 | 0.261 | 0.254 | 0.087 | 0.017 |
| **Norway** | **GDPrHGiniHv** | 0.339 | 0.352 | 0.343 | 0.125 | 0.027 |
| **Norway** | **GDPrHGiniLow** | 0.161 | 0.168 | 0.163 | 0.051 | 0.009 |
| **Norway** | **GDPrLGiniH** | 0.251 | 0.261 | 0.267 | 0.114 | 0.023 |
| **Norway** | **GDPrLGiniHv** | 0.339 | 0.352 | 0.36 | 0.162 | 0.036 |
| **Norway** | **GDPrLGiniLow** | 0.161 | 0.168 | 0.172 | 0.069 | 0.012 |
| **Norway** | **NoCOVIDginiB** | 0.189 | 0.183 | 0.194 | 0.082 | 0.015 |
| **Oman** | **GDPrGiniBase** | 0.136 | 0.147 | 0.14 | 0.081 | 0.085 |
| **Oman** | **GDPrHGiniH** | 0.175 | 0.188 | 0.174 | 0.094 | 0.099 |
| **Oman** | **GDPrHGiniHv** | 0.248 | 0.265 | 0.247 | 0.139 | 0.146 |
| **Oman** | **GDPrHGiniLow** | 0.104 | 0.113 | 0.104 | 0.053 | 0.056 |
| **Oman** | **GDPrLGiniH** | 0.175 | 0.188 | 0.18 | 0.108 | 0.116 |
| **Oman** | **GDPrLGiniHv** | 0.248 | 0.265 | 0.256 | 0.158 | 0.169 |
| **Oman** | **GDPrLGiniLow** | 0.104 | 0.113 | 0.108 | 0.062 | 0.067 |
| **Oman** | **NoCOVIDginiB** | 0.108 | 0.106 | 0.107 | 0.064 | 0.083 |
| **Pakistan** | **GDPrGiniBase** | 2.407 | 2.384 | 2.339 | 1.827 | 1.221 |
| **Pakistan** | **GDPrHGiniH** | 2.731 | 2.707 | 2.535 | 1.945 | 1.23 |
| **Pakistan** | **GDPrHGiniHv** | 3.261 | 3.234 | 3.04 | 2.37 | 1.551 |
| **Pakistan** | **GDPrHGiniLow** | 2.107 | 2.086 | 1.943 | 1.456 | 0.875 |
| **Pakistan** | **GDPrLGiniH** | 2.731 | 2.707 | 2.776 | 2.258 | 1.684 |
| **Pakistan** | **GDPrLGiniHv** | 3.261 | 3.234 | 3.312 | 2.728 | 2.084 |
| **Pakistan** | **GDPrLGiniLow** | 2.107 | 2.086 | 2.144 | 1.713 | 1.231 |
| **Pakistan** | **NoCOVIDginiB** | 2.188 | 2.208 | 2.121 | 1.674 | 1.025 |
| **Palestine** | **GDPrGiniBase** | 1.871 | 1.976 | 1.923 | 2.06 | 0.077 |
| **Palestine** | **GDPrHGiniH** | 2.146 | 2.261 | 2.092 | 2.12 | 0.086 |
| **Palestine** | **GDPrHGiniHv** | 2.6 | 2.731 | 2.539 | 2.572 | 0.127 |
| **Palestine** | **GDPrHGiniLow** | 1.619 | 1.714 | 1.576 | 1.597 | 0.049 |
| **Palestine** | **GDPrLGiniH** | 2.146 | 2.261 | 2.306 | 2.603 | 0.119 |
| **Palestine** | **GDPrLGiniHv** | 2.6 | 2.731 | 2.782 | 3.119 | 0.172 |
| **Palestine** | **GDPrLGiniLow** | 1.619 | 1.714 | 1.751 | 1.997 | 0.069 |
| **Palestine** | **NoCOVIDginiB** | 1.159 | 1.422 | 1.738 | 2.428 | 0.088 |
| **Panama** | **GDPrGiniBase** | 2.953 | 2.64 | 2.795 | 1.922 | 0.959 |
| **Panama** | **GDPrHGiniH** | 3.414 | 3.068 | 3.167 | 2.084 | 1.107 |
| **Panama** | **GDPrHGiniHv** | 4.186 | 3.79 | 3.904 | 2.647 | 1.471 |
| **Panama** | **GDPrHGiniLow** | 2.534 | 2.252 | 2.332 | 1.468 | 0.73 |
| **Panama** | **GDPrLGiniH** | 3.414 | 3.068 | 3.316 | 2.476 | 1.238 |
| **Panama** | **GDPrLGiniHv** | 4.186 | 3.79 | 4.074 | 3.107 | 1.633 |
| **Panama** | **GDPrLGiniLow** | 2.534 | 2.252 | 2.454 | 1.78 | 0.826 |
| **Panama** | **NoCOVIDginiB** | 1.897 | 1.74 | 1.609 | 1.006 | 0.658 |
| **Papua New Guinea** | **GDPrGiniBase** | 25.97 | 25.12 | 23.59 | 9.742 | 2.839 |
| **Papua New Guinea** | **GDPrHGiniH** | 26.99 | 26.14 | 24.09 | 10.08 | 3.016 |
| **Papua New Guinea** | **GDPrHGiniHv** | 28.51 | 27.67 | 25.62 | 11.31 | 3.67 |
| **Papua New Guinea** | **GDPrHGiniLow** | 24.94 | 24.09 | 22.05 | 8.527 | 2.261 |
| **Papua New Guinea** | **GDPrLGiniH** | 26.99 | 26.14 | 25.12 | 11.05 | 3.521 |
| **Papua New Guinea** | **GDPrLGiniHv** | 28.51 | 27.67 | 26.65 | 12.33 | 4.245 |
| **Papua New Guinea** | **GDPrLGiniLow** | 24.94 | 24.09 | 23.07 | 9.423 | 2.677 |
| **Papua New Guinea** | **NoCOVIDginiB** | 23.82 | 22.56 | 21.13 | 8.557 | 2.313 |
| **Paraguay** | **GDPrGiniBase** | 0.594 | 0.552 | 0.509 | 0.298 | 0.107 |
| **Paraguay** | **GDPrHGiniH** | 0.734 | 0.685 | 0.61 | 0.348 | 0.133 |
| **Paraguay** | **GDPrHGiniHv** | 0.989 | 0.927 | 0.832 | 0.493 | 0.201 |
| **Paraguay** | **GDPrHGiniLow** | 0.474 | 0.439 | 0.387 | 0.209 | 0.072 |
| **Paraguay** | **GDPrLGiniH** | 0.734 | 0.685 | 0.659 | 0.418 | 0.155 |
| **Paraguay** | **GDPrLGiniHv** | 0.989 | 0.927 | 0.895 | 0.585 | 0.233 |
| **Paraguay** | **GDPrLGiniLow** | 0.474 | 0.439 | 0.421 | 0.255 | 0.086 |
| **Paraguay** | **NoCOVIDginiB** | 0.525 | 0.486 | 0.445 | 0.261 | 0.094 |
| **Peru** | **GDPrGiniBase** | 4.332 | 3.932 | 3.815 | 2.288 | 1.232 |
| **Peru** | **GDPrHGiniH** | 4.863 | 4.433 | 4.194 | 2.486 | 1.38 |
| **Peru** | **GDPrHGiniHv** | 5.722 | 5.247 | 4.982 | 3.056 | 1.763 |
| **Peru** | **GDPrHGiniLow** | 3.834 | 3.465 | 3.261 | 1.838 | 0.963 |
| **Peru** | **GDPrLGiniH** | 4.863 | 4.433 | 4.423 | 2.809 | 1.547 |
| **Peru** | **GDPrLGiniHv** | 5.722 | 5.247 | 5.236 | 3.425 | 1.963 |
| **Peru** | **GDPrLGiniLow** | 3.834 | 3.465 | 3.456 | 2.101 | 1.092 |
| **Peru** | **NoCOVIDginiB** | 3.218 | 3.093 | 2.898 | 1.877 | 1.012 |
| **Philippines** | **GDPrGiniBase** | 5.904 | 6.05 | 6.135 | 4.593 | 2.023 |
| **Philippines** | **GDPrHGiniH** | 6.554 | 6.71 | 6.632 | 4.914 | 2.202 |
| **Philippines** | **GDPrHGiniHv** | 7.593 | 7.763 | 7.677 | 5.804 | 2.747 |
| **Philippines** | **GDPrHGiniLow** | 5.288 | 5.425 | 5.356 | 3.853 | 1.591 |
| **Philippines** | **GDPrLGiniH** | 6.554 | 6.71 | 6.973 | 5.418 | 2.524 |
| **Philippines** | **GDPrLGiniHv** | 7.593 | 7.763 | 8.046 | 6.358 | 3.12 |
| **Philippines** | **GDPrLGiniLow** | 5.288 | 5.425 | 5.659 | 4.289 | 1.849 |
| **Philippines** | **NoCOVIDginiB** | 4.18 | 3.999 | 3.85 | 3.295 | 1.27 |
| **Poland** | **GDPrGiniBase** | 0.206 | 0.188 | 0.17 | 0.034 | 0.003 |
| **Poland** | **GDPrHGiniH** | 0.257 | 0.235 | 0.207 | 0.04 | 0.004 |
| **Poland** | **GDPrHGiniHv** | 0.35 | 0.322 | 0.285 | 0.061 | 0.007 |
| **Poland** | **GDPrHGiniLow** | 0.163 | 0.148 | 0.129 | 0.022 | 0.002 |
| **Poland** | **GDPrLGiniH** | 0.257 | 0.235 | 0.221 | 0.053 | 0.006 |
| **Poland** | **GDPrLGiniHv** | 0.35 | 0.322 | 0.304 | 0.079 | 0.01 |
| **Poland** | **GDPrLGiniLow** | 0.163 | 0.148 | 0.139 | 0.029 | 0.003 |
| **Poland** | **NoCOVIDginiB** | 0.181 | 0.164 | 0.152 | 0.039 | 0.003 |
| **Portugal** | **GDPrGiniBase** | 0.383 | 0.366 | 0.363 | 0.207 | 0.059 |
| **Portugal** | **GDPrHGiniH** | 0.469 | 0.45 | 0.435 | 0.22 | 0.068 |
| **Portugal** | **GDPrHGiniHv** | 0.623 | 0.599 | 0.58 | 0.306 | 0.102 |
| **Portugal** | **GDPrHGiniLow** | 0.309 | 0.295 | 0.284 | 0.135 | 0.038 |
| **Portugal** | **GDPrLGiniH** | 0.469 | 0.45 | 0.458 | 0.308 | 0.089 |
| **Portugal** | **GDPrLGiniHv** | 0.623 | 0.599 | 0.609 | 0.421 | 0.131 |
| **Portugal** | **GDPrLGiniLow** | 0.309 | 0.295 | 0.301 | 0.196 | 0.051 |
| **Portugal** | **NoCOVIDginiB** | 0.316 | 0.3 | 0.288 | 0.178 | 0.053 |
| **Puerto Rico** | **GDPrGiniBase** | 0.073 | 0.065 | 0.062 | 0.021 | 0.002 |
| **Puerto Rico** | **GDPrHGiniH** | 0.102 | 0.091 | 0.084 | 0.029 | 0.003 |
| **Puerto Rico** | **GDPrHGiniHv** | 0.162 | 0.147 | 0.136 | 0.05 | 0.006 |
| **Puerto Rico** | **GDPrHGiniLow** | 0.051 | 0.045 | 0.041 | 0.013 | 0.001 |
| **Puerto Rico** | **GDPrLGiniH** | 0.102 | 0.091 | 0.09 | 0.033 | 0.003 |
| **Puerto Rico** | **GDPrLGiniHv** | 0.162 | 0.147 | 0.145 | 0.058 | 0.007 |
| **Puerto Rico** | **GDPrLGiniLow** | 0.051 | 0.045 | 0.045 | 0.015 | 0.001 |
| **Puerto Rico** | **NoCOVIDginiB** | 0.067 | 0.062 | 0.06 | 0.019 | 0.002 |
| **Qatar** | **GDPrGiniBase** | 0.117 | 0.118 | 0.107 | 0.031 | 0.006 |
| **Qatar** | **GDPrHGiniH** | 0.153 | 0.154 | 0.137 | 0.039 | 0.008 |
| **Qatar** | **GDPrHGiniHv** | 0.224 | 0.226 | 0.201 | 0.062 | 0.014 |
| **Qatar** | **GDPrHGiniLow** | 0.088 | 0.088 | 0.077 | 0.02 | 0.003 |
| **Qatar** | **GDPrLGiniH** | 0.153 | 0.154 | 0.142 | 0.048 | 0.011 |
| **Qatar** | **GDPrLGiniHv** | 0.224 | 0.226 | 0.209 | 0.075 | 0.019 |
| **Qatar** | **GDPrLGiniLow** | 0.088 | 0.088 | 0.081 | 0.024 | 0.005 |
| **Qatar** | **NoCOVIDginiB** | 0.099 | 0.094 | 0.089 | 0.026 | 0.005 |
| **Romania** | **GDPrGiniBase** | 2.417 | 2.149 | 1.962 | 1.026 | 0.343 |
| **Romania** | **GDPrHGiniH** | 2.752 | 2.449 | 2.155 | 1.106 | 0.371 |
| **Romania** | **GDPrHGiniHv** | 3.302 | 2.947 | 2.624 | 1.41 | 0.494 |
| **Romania** | **GDPrHGiniLow** | 2.107 | 1.866 | 1.607 | 0.778 | 0.241 |
| **Romania** | **GDPrLGiniH** | 2.752 | 2.449 | 2.34 | 1.329 | 0.466 |
| **Romania** | **GDPrLGiniHv** | 3.302 | 2.947 | 2.834 | 1.677 | 0.617 |
| **Romania** | **GDPrLGiniLow** | 2.107 | 1.866 | 1.758 | 0.947 | 0.308 |
| **Romania** | **NoCOVIDginiB** | 2.053 | 1.936 | 1.821 | 1.017 | 0.335 |
| **Russian Federation** | **GDPrGiniBase** | 0.121 | 0.11 | 0.105 | 0.037 | 0.013 |
| **Russian Federation** | **GDPrHGiniH** | 0.157 | 0.144 | 0.133 | 0.044 | 0.016 |
| **Russian Federation** | **GDPrHGiniHv** | 0.226 | 0.208 | 0.193 | 0.069 | 0.027 |
| **Russian Federation** | **GDPrHGiniLow** | 0.092 | 0.083 | 0.076 | 0.023 | 0.008 |
| **Russian Federation** | **GDPrLGiniH** | 0.157 | 0.144 | 0.141 | 0.056 | 0.021 |
| **Russian Federation** | **GDPrLGiniHv** | 0.226 | 0.208 | 0.205 | 0.087 | 0.035 |
| **Russian Federation** | **GDPrLGiniLow** | 0.092 | 0.083 | 0.082 | 0.03 | 0.01 |
| **Russian Federation** | **NoCOVIDginiB** | 0.109 | 0.1 | 0.092 | 0.034 | 0.012 |
| **Rwanda** | **GDPrGiniBase** | 54.45 | 55.02 | 53.73 | 43.01 | 14.4 |
| **Rwanda** | **GDPrHGiniH** | 55.08 | 55.66 | 53.53 | 42.51 | 14.62 |
| **Rwanda** | **GDPrHGiniHv** | 56 | 56.58 | 54.48 | 43.79 | 16.24 |
| **Rwanda** | **GDPrHGiniLow** | 53.82 | 54.39 | 52.23 | 40.77 | 12.53 |
| **Rwanda** | **GDPrLGiniH** | 55.08 | 55.66 | 55.2 | 45.17 | 16.44 |
| **Rwanda** | **GDPrLGiniHv** | 56 | 56.58 | 56.09 | 46.37 | 18.12 |
| **Rwanda** | **GDPrLGiniLow** | 53.82 | 54.39 | 53.99 | 43.52 | 14.25 |
| **Rwanda** | **NoCOVIDginiB** | 48.61 | 47.15 | 46.11 | 36.79 | 10.32 |
| **Samoa** | **GDPrGiniBase** | 0.54 | 0.705 | 0.7 | 0.293 | 0.065 |
| **Samoa** | **GDPrHGiniH** | 0.659 | 0.85 | 0.796 | 0.312 | 0.076 |
| **Samoa** | **GDPrHGiniHv** | 0.87 | 1.106 | 1.038 | 0.432 | 0.115 |
| **Samoa** | **GDPrHGiniLow** | 0.438 | 0.578 | 0.539 | 0.194 | 0.041 |
| **Samoa** | **GDPrLGiniH** | 0.659 | 0.85 | 0.885 | 0.419 | 0.1 |
| **Samoa** | **GDPrLGiniHv** | 0.87 | 1.106 | 1.146 | 0.568 | 0.15 |
| **Samoa** | **GDPrLGiniLow** | 0.438 | 0.578 | 0.605 | 0.267 | 0.055 |
| **Samoa** | **NoCOVIDginiB** | 0.392 | 0.352 | 0.355 | 0.142 | 0.03 |
| **Sao Tome and Principe** | **GDPrGiniBase** | 36.67 | 37.62 | 37.68 | 37.11 | 22.66 |
| **Sao Tome and Principe** | **GDPrHGiniH** | 38 | 38.93 | 38.4 | 37.27 | 23.03 |
| **Sao Tome and Principe** | **GDPrHGiniHv** | 39.98 | 40.9 | 40.38 | 39.28 | 25.18 |
| **Sao Tome and Principe** | **GDPrHGiniLow** | 35.34 | 36.29 | 35.75 | 34.59 | 20.24 |
| **Sao Tome and Principe** | **GDPrLGiniH** | 38 | 38.93 | 39.61 | 39.66 | 25.2 |
| **Sao Tome and Principe** | **GDPrLGiniHv** | 39.98 | 40.9 | 41.56 | 41.61 | 27.38 |
| **Sao Tome and Principe** | **GDPrLGiniLow** | 35.34 | 36.29 | 36.99 | 37.03 | 22.35 |
| **Sao Tome and Principe** | **NoCOVIDginiB** | 36.2 | 36.66 | 36.86 | 35.48 | 21.01 |
| **Saudi Arabia** | **GDPrGiniBase** | 0.124 | 0.126 | 0.114 | 0.041 | 0.016 |
| **Saudi Arabia** | **GDPrHGiniH** | 0.161 | 0.163 | 0.14 | 0.051 | 0.019 |
| **Saudi Arabia** | **GDPrHGiniHv** | 0.23 | 0.234 | 0.202 | 0.078 | 0.032 |
| **Saudi Arabia** | **GDPrHGiniLow** | 0.094 | 0.096 | 0.081 | 0.027 | 0.009 |
| **Saudi Arabia** | **GDPrLGiniH** | 0.161 | 0.163 | 0.153 | 0.06 | 0.026 |
| **Saudi Arabia** | **GDPrLGiniHv** | 0.23 | 0.234 | 0.22 | 0.092 | 0.041 |
| **Saudi Arabia** | **GDPrLGiniLow** | 0.094 | 0.096 | 0.09 | 0.032 | 0.013 |
| **Saudi Arabia** | **NoCOVIDginiB** | 0.101 | 0.097 | 0.091 | 0.038 | 0.014 |
| **Senegal** | **GDPrGiniBase** | 29.71 | 29.51 | 28.66 | 20.36 | 3.761 |
| **Senegal** | **GDPrHGiniH** | 30.68 | 30.49 | 28.89 | 20.35 | 3.923 |
| **Senegal** | **GDPrHGiniHv** | 32.12 | 31.94 | 30.34 | 21.81 | 4.683 |
| **Senegal** | **GDPrHGiniLow** | 28.74 | 28.53 | 26.93 | 18.41 | 3.028 |
| **Senegal** | **GDPrLGiniH** | 30.68 | 30.49 | 30.33 | 22.31 | 4.604 |
| **Senegal** | **GDPrLGiniHv** | 32.12 | 31.94 | 31.76 | 23.78 | 5.438 |
| **Senegal** | **GDPrLGiniLow** | 28.74 | 28.53 | 28.39 | 20.35 | 3.61 |
| **Senegal** | **NoCOVIDginiB** | 27.26 | 26.07 | 24.41 | 16.89 | 2.696 |
| **Serbia** | **GDPrGiniBase** | 4.087 | 3.681 | 3.319 | 1.367 | 0.385 |
| **Serbia** | **GDPrHGiniH** | 4.555 | 4.12 | 3.556 | 1.456 | 0.433 |
| **Serbia** | **GDPrHGiniHv** | 5.304 | 4.828 | 4.203 | 1.816 | 0.584 |
| **Serbia** | **GDPrHGiniLow** | 3.646 | 3.268 | 2.785 | 1.053 | 0.279 |
| **Serbia** | **GDPrLGiniH** | 4.555 | 4.12 | 3.811 | 1.738 | 0.518 |
| **Serbia** | **GDPrLGiniHv** | 5.304 | 4.828 | 4.486 | 2.145 | 0.691 |
| **Serbia** | **GDPrLGiniLow** | 3.646 | 3.268 | 3.003 | 1.277 | 0.339 |
| **Serbia** | **NoCOVIDginiB** | 3.754 | 3.452 | 3.16 | 1.339 | 0.372 |
| **Seychelles** | **GDPrGiniBase** | 1.302 | 1.255 | 1.143 | 0.721 | 0.633 |
| **Seychelles** | **GDPrHGiniH** | 1.555 | 1.502 | 1.322 | 0.819 | 0.709 |
| **Seychelles** | **GDPrHGiniHv** | 1.995 | 1.932 | 1.715 | 1.099 | 0.962 |
| **Seychelles** | **GDPrHGiniLow** | 1.079 | 1.038 | 0.902 | 0.533 | 0.454 |
| **Seychelles** | **GDPrLGiniH** | 1.555 | 1.502 | 1.427 | 0.956 | 0.859 |
| **Seychelles** | **GDPrLGiniHv** | 1.995 | 1.932 | 1.842 | 1.269 | 1.15 |
| **Seychelles** | **GDPrLGiniLow** | 1.079 | 1.038 | 0.982 | 0.632 | 0.561 |
| **Seychelles** | **NoCOVIDginiB** | 0.857 | 0.868 | 0.882 | 0.646 | 0.58 |
| **Sierra Leone** | **GDPrGiniBase** | 45.15 | 46.31 | 45.2 | 40.77 | 23.14 |
| **Sierra Leone** | **GDPrHGiniH** | 45.81 | 46.95 | 44.67 | 39.61 | 21.21 |
| **Sierra Leone** | **GDPrHGiniHv** | 46.77 | 47.89 | 45.66 | 40.7 | 22.68 |
| **Sierra Leone** | **GDPrHGiniLow** | 44.49 | 45.66 | 43.32 | 38.12 | 19.18 |
| **Sierra Leone** | **GDPrLGiniH** | 45.81 | 46.95 | 47.06 | 43.64 | 27.39 |
| **Sierra Leone** | **GDPrLGiniHv** | 46.77 | 47.89 | 47.98 | 44.63 | 28.88 |
| **Sierra Leone** | **GDPrLGiniLow** | 44.49 | 45.66 | 45.8 | 42.29 | 25.36 |
| **Sierra Leone** | **NoCOVIDginiB** | 40.83 | 40.86 | 41.35 | 36.11 | 16.17 |
| **Singapore** | **GDPrGiniBase** | 0.099 | 0.085 | 0.078 | 0.017 | 0.001 |
| **Singapore** | **GDPrHGiniH** | 0.129 | 0.112 | 0.1 | 0.022 | 0.002 |
| **Singapore** | **GDPrHGiniHv** | 0.188 | 0.165 | 0.148 | 0.036 | 0.004 |
| **Singapore** | **GDPrHGiniLow** | 0.075 | 0.064 | 0.056 | 0.011 | 0.001 |
| **Singapore** | **GDPrLGiniH** | 0.129 | 0.112 | 0.107 | 0.028 | 0.003 |
| **Singapore** | **GDPrLGiniHv** | 0.188 | 0.165 | 0.158 | 0.045 | 0.005 |
| **Singapore** | **GDPrLGiniLow** | 0.075 | 0.064 | 0.061 | 0.014 | 0.001 |
| **Singapore** | **NoCOVIDginiB** | 0.085 | 0.083 | 0.079 | 0.02 | 0.001 |
| **Slovakia** | **GDPrGiniBase** | 1.097 | 1.071 | 1.065 | 0.317 | 0.053 |
| **Slovakia** | **GDPrHGiniH** | 1.269 | 1.24 | 1.191 | 0.333 | 0.055 |
| **Slovakia** | **GDPrHGiniHv** | 1.556 | 1.523 | 1.466 | 0.441 | 0.081 |
| **Slovakia** | **GDPrHGiniLow** | 0.94 | 0.917 | 0.877 | 0.22 | 0.031 |
| **Slovakia** | **GDPrLGiniH** | 1.269 | 1.24 | 1.278 | 0.442 | 0.087 |
| **Slovakia** | **GDPrLGiniHv** | 1.556 | 1.523 | 1.568 | 0.576 | 0.125 |
| **Slovakia** | **GDPrLGiniLow** | 0.94 | 0.917 | 0.948 | 0.299 | 0.051 |
| **Slovakia** | **NoCOVIDginiB** | 0.904 | 0.871 | 0.831 | 0.314 | 0.048 |
| **Slovenia** | **GDPrGiniBase** | 0.06 | 0.053 | 0.05 | 0.008 | 0 |
| **Slovenia** | **GDPrHGiniH** | 0.078 | 0.069 | 0.061 | 0.009 | 0 |
| **Slovenia** | **GDPrHGiniHv** | 0.113 | 0.1 | 0.09 | 0.015 | 0.001 |
| **Slovenia** | **GDPrHGiniLow** | 0.046 | 0.04 | 0.035 | 0.005 | 0 |
| **Slovenia** | **GDPrLGiniH** | 0.078 | 0.069 | 0.068 | 0.015 | 0.001 |
| **Slovenia** | **GDPrLGiniHv** | 0.113 | 0.1 | 0.099 | 0.023 | 0.002 |
| **Slovenia** | **GDPrLGiniLow** | 0.046 | 0.04 | 0.039 | 0.008 | 0 |
| **Slovenia** | **NoCOVIDginiB** | 0.049 | 0.042 | 0.037 | 0.008 | 0 |
| **Solomon Islands** | **GDPrGiniBase** | 28.52 | 29.09 | 27.9 | 21.86 | 12.4 |
| **Solomon Islands** | **GDPrHGiniH** | 29.43 | 30 | 28.11 | 21.42 | 11.71 |
| **Solomon Islands** | **GDPrHGiniHv** | 30.79 | 31.35 | 29.48 | 22.81 | 12.98 |
| **Solomon Islands** | **GDPrHGiniLow** | 27.6 | 28.17 | 26.26 | 19.56 | 10.08 |
| **Solomon Islands** | **GDPrLGiniH** | 29.43 | 30 | 29.33 | 24.08 | 14.83 |
| **Solomon Islands** | **GDPrLGiniHv** | 30.79 | 31.35 | 30.69 | 25.47 | 16.19 |
| **Solomon Islands** | **GDPrLGiniLow** | 27.6 | 28.17 | 27.49 | 22.21 | 13.05 |
| **Solomon Islands** | **NoCOVIDginiB** | 25.22 | 25.09 | 24.84 | 19.92 | 9.645 |
| **Somalia** | **GDPrGiniBase** | 40.97 | 42.96 | 43.78 | 37.58 | 5.097 |
| **Somalia** | **GDPrHGiniH** | 41.83 | 43.82 | 43.53 | 37.06 | 5.208 |
| **Somalia** | **GDPrHGiniHv** | 43.1 | 45.06 | 44.78 | 38.29 | 6.227 |
| **Somalia** | **GDPrHGiniLow** | 40.1 | 42.1 | 41.82 | 35.39 | 4.011 |
| **Somalia** | **GDPrLGiniH** | 41.83 | 43.82 | 45.73 | 39.71 | 6.431 |
| **Somalia** | **GDPrLGiniHv** | 43.1 | 45.06 | 46.92 | 40.89 | 7.582 |
| **Somalia** | **GDPrLGiniLow** | 40.1 | 42.1 | 44.11 | 38.14 | 5.049 |
| **Somalia** | **NoCOVIDginiB** | 39.35 | 41.09 | 43.42 | 36.08 | 4.64 |
| **South Africa** | **GDPrGiniBase** | 20.29 | 19.84 | 19.66 | 18.77 | 16.75 |
| **South Africa** | **GDPrHGiniH** | 21.87 | 21.41 | 21.02 | 19.91 | 17.85 |
| **South Africa** | **GDPrHGiniHv** | 24.33 | 23.86 | 23.47 | 22.34 | 20.25 |
| **South Africa** | **GDPrHGiniLow** | 18.75 | 18.31 | 17.93 | 16.86 | 14.87 |
| **South Africa** | **GDPrLGiniH** | 21.87 | 21.41 | 21.45 | 20.79 | 18.74 |
| **South Africa** | **GDPrLGiniHv** | 24.33 | 23.86 | 23.9 | 23.24 | 21.17 |
| **South Africa** | **GDPrLGiniLow** | 18.75 | 18.31 | 18.34 | 17.69 | 15.71 |
| **South Africa** | **NoCOVIDginiB** | 19.21 | 19.11 | 18.99 | 18.13 | 16.02 |
| **Spain** | **GDPrGiniBase** | 0.727 | 0.696 | 0.676 | 0.327 | 0.108 |
| **Spain** | **GDPrHGiniH** | 0.869 | 0.833 | 0.79 | 0.357 | 0.123 |
| **Spain** | **GDPrHGiniHv** | 1.114 | 1.072 | 1.019 | 0.484 | 0.178 |
| **Spain** | **GDPrHGiniLow** | 0.603 | 0.576 | 0.543 | 0.228 | 0.071 |
| **Spain** | **GDPrLGiniH** | 0.869 | 0.833 | 0.831 | 0.461 | 0.159 |
| **Spain** | **GDPrLGiniHv** | 1.114 | 1.072 | 1.069 | 0.615 | 0.227 |
| **Spain** | **GDPrLGiniLow** | 0.603 | 0.576 | 0.574 | 0.302 | 0.094 |
| **Spain** | **NoCOVIDginiB** | 0.573 | 0.541 | 0.508 | 0.281 | 0.093 |
| **Sri Lanka** | **GDPrGiniBase** | 0.833 | 0.776 | 0.722 | 0.389 | 0.09 |
| **Sri Lanka** | **GDPrHGiniH** | 0.999 | 0.934 | 0.833 | 0.44 | 0.107 |
| **Sri Lanka** | **GDPrHGiniHv** | 1.29 | 1.211 | 1.087 | 0.599 | 0.16 |
| **Sri Lanka** | **GDPrHGiniLow** | 0.687 | 0.638 | 0.563 | 0.28 | 0.059 |
| **Sri Lanka** | **GDPrLGiniH** | 0.999 | 0.934 | 0.906 | 0.525 | 0.131 |
| **Sri Lanka** | **GDPrLGiniHv** | 1.29 | 1.211 | 1.177 | 0.706 | 0.193 |
| **Sri Lanka** | **GDPrLGiniLow** | 0.687 | 0.638 | 0.617 | 0.339 | 0.074 |
| **Sri Lanka** | **NoCOVIDginiB** | 0.664 | 0.596 | 0.523 | 0.284 | 0.062 |
| **St. Lucia** | **GDPrGiniBase** | 7.186 | 7.613 | 6.785 | 4.269 | 2.393 |
| **St. Lucia** | **GDPrHGiniH** | 7.996 | 8.45 | 7.403 | 4.677 | 2.704 |
| **St. Lucia** | **GDPrHGiniHv** | 9.295 | 9.786 | 8.651 | 5.647 | 3.399 |
| **St. Lucia** | **GDPrHGiniLow** | 6.421 | 6.822 | 5.899 | 3.55 | 1.934 |
| **St. Lucia** | **GDPrLGiniH** | 7.996 | 8.45 | 7.745 | 5.067 | 2.919 |
| **St. Lucia** | **GDPrLGiniHv** | 9.295 | 9.786 | 9.022 | 6.084 | 3.65 |
| **St. Lucia** | **GDPrLGiniLow** | 6.421 | 6.822 | 6.2 | 3.879 | 2.106 |
| **St. Lucia** | **NoCOVIDginiB** | 4.543 | 4.679 | 4.713 | 3.598 | 2.053 |
| **St. Vincent and the Grenadines** | **GDPrGiniBase** | 11.83 | 14.09 | 12.63 | 11.47 | 6.917 |
| **St. Vincent and the Grenadines** | **GDPrHGiniH** | 12.72 | 15.03 | 13.21 | 11.72 | 7.372 |
| **St. Vincent and the Grenadines** | **GDPrHGiniHv** | 14.08 | 16.48 | 14.6 | 13.05 | 8.47 |
| **St. Vincent and the Grenadines** | **GDPrHGiniLow** | 10.96 | 13.16 | 11.43 | 10.02 | 6.018 |
| **St. Vincent and the Grenadines** | **GDPrLGiniH** | 12.72 | 15.03 | 13.88 | 12.88 | 7.886 |
| **St. Vincent and the Grenadines** | **GDPrLGiniHv** | 14.08 | 16.48 | 15.29 | 14.26 | 9.021 |
| **St. Vincent and the Grenadines** | **GDPrLGiniLow** | 10.96 | 13.16 | 12.07 | 11.11 | 6.479 |
| **St. Vincent and the Grenadines** | **NoCOVIDginiB** | 10.53 | 10.79 | 11.12 | 10.43 | 6.392 |
| **Sudan** | **GDPrGiniBase** | 15.52 | 16.46 | 15.81 | 10.12 | 4.114 |
| **Sudan** | **GDPrHGiniH** | 16.35 | 17.31 | 16.13 | 10.34 | 4.151 |
| **Sudan** | **GDPrHGiniHv** | 17.61 | 18.59 | 17.39 | 11.44 | 4.863 |
| **Sudan** | **GDPrHGiniLow** | 14.68 | 15.61 | 14.46 | 8.929 | 3.292 |
| **Sudan** | **GDPrLGiniH** | 16.35 | 17.31 | 17.19 | 11.38 | 5.08 |
| **Sudan** | **GDPrLGiniHv** | 17.61 | 18.59 | 18.47 | 12.51 | 5.878 |
| **Sudan** | **GDPrLGiniLow** | 14.68 | 15.61 | 15.5 | 9.906 | 4.103 |
| **Sudan** | **NoCOVIDginiB** | 14.59 | 16.23 | 16.99 | 11.86 | 5.323 |
| **Sudan South** | **GDPrGiniBase** | 84.43 | 82.75 | 81.54 | 74.61 | 34.23 |
| **Sudan South** | **GDPrHGiniH** | 84.36 | 82.71 | 81.11 | 73.68 | 33.75 |
| **Sudan South** | **GDPrHGiniHv** | 84.29 | 82.68 | 81.13 | 73.98 | 35.42 |
| **Sudan South** | **GDPrHGiniLow** | 84.52 | 82.79 | 81.12 | 73.31 | 31.48 |
| **Sudan South** | **GDPrLGiniH** | 84.36 | 82.71 | 81.89 | 75.81 | 37 |
| **Sudan South** | **GDPrLGiniHv** | 84.29 | 82.68 | 81.88 | 76.03 | 38.61 |
| **Sudan South** | **GDPrLGiniLow** | 84.52 | 82.79 | 81.93 | 75.55 | 34.82 |
| **Sudan South** | **NoCOVIDginiB** | 80.08 | 78.49 | 78.24 | 68.82 | 27.74 |
| **Suriname** | **GDPrGiniBase** | 19.97 | 19.69 | 20.51 | 14.96 | 10.2 |
| **Suriname** | **GDPrHGiniH** | 21.35 | 21.07 | 21.54 | 15.66 | 10.9 |
| **Suriname** | **GDPrHGiniHv** | 23.49 | 23.2 | 23.68 | 17.64 | 12.63 |
| **Suriname** | **GDPrHGiniLow** | 18.61 | 18.34 | 18.79 | 13.18 | 8.799 |
| **Suriname** | **GDPrLGiniH** | 21.35 | 21.07 | 22.27 | 16.78 | 11.71 |
| **Suriname** | **GDPrLGiniHv** | 23.49 | 23.2 | 24.42 | 18.81 | 13.49 |
| **Suriname** | **GDPrLGiniLow** | 18.61 | 18.34 | 19.5 | 14.23 | 9.527 |
| **Suriname** | **NoCOVIDginiB** | 16.05 | 15.25 | 14.71 | 11.01 | 7.588 |
| **Sweden** | **GDPrGiniBase** | 0.219 | 0.223 | 0.224 | 0.092 | 0.01 |
| **Sweden** | **GDPrHGiniH** | 0.272 | 0.276 | 0.27 | 0.102 | 0.011 |
| **Sweden** | **GDPrHGiniHv** | 0.368 | 0.373 | 0.365 | 0.146 | 0.019 |
| **Sweden** | **GDPrHGiniLow** | 0.174 | 0.177 | 0.173 | 0.06 | 0.006 |
| **Sweden** | **GDPrLGiniH** | 0.272 | 0.276 | 0.287 | 0.137 | 0.016 |
| **Sweden** | **GDPrLGiniHv** | 0.368 | 0.373 | 0.387 | 0.193 | 0.026 |
| **Sweden** | **GDPrLGiniLow** | 0.174 | 0.177 | 0.185 | 0.083 | 0.008 |
| **Sweden** | **NoCOVIDginiB** | 0.198 | 0.202 | 0.2 | 0.092 | 0.01 |
| **Switzerland** | **GDPrGiniBase** | 0.096 | 0.084 | 0.076 | 0.045 | 0.01 |
| **Switzerland** | **GDPrHGiniH** | 0.124 | 0.109 | 0.092 | 0.054 | 0.013 |
| **Switzerland** | **GDPrHGiniHv** | 0.177 | 0.158 | 0.135 | 0.081 | 0.021 |
| **Switzerland** | **GDPrHGiniLow** | 0.073 | 0.063 | 0.053 | 0.029 | 0.006 |
| **Switzerland** | **GDPrLGiniH** | 0.124 | 0.109 | 0.103 | 0.066 | 0.017 |
| **Switzerland** | **GDPrLGiniHv** | 0.177 | 0.158 | 0.149 | 0.099 | 0.027 |
| **Switzerland** | **GDPrLGiniLow** | 0.073 | 0.063 | 0.059 | 0.037 | 0.008 |
| **Switzerland** | **NoCOVIDginiB** | 0.085 | 0.082 | 0.077 | 0.047 | 0.011 |
| **Syrian Arab Republic** | **GDPrGiniBase** | 29.72 | 31.41 | 33.96 | 30.12 | 27.83 |
| **Syrian Arab Republic** | **GDPrHGiniH** | 30.6 | 32.27 | 34.11 | 29.95 | 27.29 |
| **Syrian Arab Republic** | **GDPrHGiniHv** | 31.9 | 33.55 | 35.36 | 31.27 | 28.64 |
| **Syrian Arab Republic** | **GDPrHGiniLow** | 28.82 | 30.53 | 32.41 | 28.17 | 25.45 |
| **Syrian Arab Republic** | **GDPrLGiniH** | 30.6 | 32.27 | 35.51 | 32.1 | 30.27 |
| **Syrian Arab Republic** | **GDPrLGiniHv** | 31.9 | 33.55 | 36.73 | 33.38 | 31.6 |
| **Syrian Arab Republic** | **GDPrLGiniLow** | 28.82 | 30.53 | 33.84 | 30.35 | 28.46 |
| **Syrian Arab Republic** | **NoCOVIDginiB** | 29.74 | 31.47 | 34.06 | 30.25 | 27.06 |
| **Taiwan** | **GDPrGiniBase** | 0.055 | 0.037 | 0.03 | 0.006 | 0 |
| **Taiwan** | **GDPrHGiniH** | 0.072 | 0.05 | 0.037 | 0.007 | 0 |
| **Taiwan** | **GDPrHGiniHv** | 0.105 | 0.074 | 0.056 | 0.012 | 0 |
| **Taiwan** | **GDPrHGiniLow** | 0.042 | 0.028 | 0.02 | 0.003 | 0 |
| **Taiwan** | **GDPrLGiniH** | 0.072 | 0.05 | 0.044 | 0.01 | 0 |
| **Taiwan** | **GDPrLGiniHv** | 0.105 | 0.074 | 0.066 | 0.016 | 0 |
| **Taiwan** | **GDPrLGiniLow** | 0.042 | 0.028 | 0.024 | 0.005 | 0 |
| **Taiwan** | **NoCOVIDginiB** | 0.059 | 0.051 | 0.043 | 0.008 | 0 |
| **Tajikistan** | **GDPrGiniBase** | 2.021 | 1.997 | 1.975 | 1.056 | 0.05 |
| **Tajikistan** | **GDPrHGiniH** | 2.311 | 2.285 | 2.106 | 1.106 | 0.056 |
| **Tajikistan** | **GDPrHGiniHv** | 2.79 | 2.761 | 2.556 | 1.395 | 0.085 |
| **Tajikistan** | **GDPrHGiniLow** | 1.754 | 1.732 | 1.586 | 0.788 | 0.031 |
| **Tajikistan** | **GDPrLGiniH** | 2.311 | 2.285 | 2.385 | 1.37 | 0.077 |
| **Tajikistan** | **GDPrLGiniHv** | 2.79 | 2.761 | 2.874 | 1.704 | 0.114 |
| **Tajikistan** | **GDPrLGiniLow** | 1.754 | 1.732 | 1.816 | 0.994 | 0.043 |
| **Tajikistan** | **NoCOVIDginiB** | 1.96 | 2.007 | 2.09 | 1.087 | 0.053 |
| **Tanzania** | **GDPrGiniBase** | 46.49 | 46.7 | 45.87 | 32.54 | 13.18 |
| **Tanzania** | **GDPrHGiniH** | 47.22 | 47.45 | 45.78 | 32.15 | 12.99 |
| **Tanzania** | **GDPrHGiniHv** | 48.29 | 48.53 | 46.87 | 33.52 | 14.44 |
| **Tanzania** | **GDPrHGiniLow** | 45.75 | 45.96 | 44.27 | 30.29 | 11.09 |
| **Tanzania** | **GDPrLGiniH** | 47.22 | 47.45 | 47.44 | 34.8 | 15.49 |
| **Tanzania** | **GDPrLGiniHv** | 48.29 | 48.53 | 48.48 | 36.14 | 17.03 |
| **Tanzania** | **GDPrLGiniLow** | 45.75 | 45.96 | 46 | 32.99 | 13.47 |
| **Tanzania** | **NoCOVIDginiB** | 44.32 | 43.32 | 42.22 | 28.88 | 10.38 |
| **Thailand** | **GDPrGiniBase** | 0.13 | 0.142 | 0.137 | 0.027 | 0.008 |
| **Thailand** | **GDPrHGiniH** | 0.168 | 0.182 | 0.166 | 0.033 | 0.01 |
| **Thailand** | **GDPrHGiniHv** | 0.239 | 0.258 | 0.237 | 0.053 | 0.017 |
| **Thailand** | **GDPrHGiniLow** | 0.099 | 0.109 | 0.098 | 0.017 | 0.005 |
| **Thailand** | **GDPrLGiniH** | 0.168 | 0.182 | 0.188 | 0.041 | 0.013 |
| **Thailand** | **GDPrLGiniHv** | 0.239 | 0.258 | 0.266 | 0.064 | 0.022 |
| **Thailand** | **GDPrLGiniLow** | 0.099 | 0.109 | 0.112 | 0.021 | 0.006 |
| **Thailand** | **NoCOVIDginiB** | 0.089 | 0.082 | 0.075 | 0.019 | 0.005 |
| **Timor-Leste** | **GDPrGiniBase** | 9.903 | 10.12 | 11.58 | 2.726 | 0.006 |
| **Timor-Leste** | **GDPrHGiniH** | 10.57 | 10.79 | 11.18 | 2.581 | 0.006 |
| **Timor-Leste** | **GDPrHGiniHv** | 11.58 | 11.82 | 12.21 | 3.06 | 0.01 |
| **Timor-Leste** | **GDPrHGiniLow** | 9.25 | 9.46 | 9.827 | 2.012 | 0.003 |
| **Timor-Leste** | **GDPrLGiniH** | 10.57 | 10.79 | 12.97 | 3.439 | 0.01 |
| **Timor-Leste** | **GDPrLGiniHv** | 11.58 | 11.82 | 14.06 | 4.008 | 0.016 |
| **Timor-Leste** | **GDPrLGiniLow** | 9.25 | 9.46 | 11.54 | 2.749 | 0.005 |
| **Timor-Leste** | **NoCOVIDginiB** | 11.03 | 11.57 | 14.08 | 2.734 | 0.005 |
| **Togo** | **GDPrGiniBase** | 46.25 | 46.32 | 45.56 | 40.45 | 9.743 |
| **Togo** | **GDPrHGiniH** | 47.04 | 47.11 | 45.64 | 39.88 | 9.702 |
| **Togo** | **GDPrHGiniHv** | 48.2 | 48.27 | 46.84 | 41.25 | 10.98 |
| **Togo** | **GDPrHGiniLow** | 45.44 | 45.52 | 43.99 | 38.02 | 8.098 |
| **Togo** | **GDPrLGiniH** | 47.04 | 47.11 | 47.12 | 42.84 | 11.56 |
| **Togo** | **GDPrLGiniHv** | 48.2 | 48.27 | 48.28 | 44.13 | 12.94 |
| **Togo** | **GDPrLGiniLow** | 45.44 | 45.52 | 45.53 | 41.07 | 9.818 |
| **Togo** | **NoCOVIDginiB** | 44.2 | 43.87 | 43.87 | 39.22 | 8.811 |
| **Tonga** | **GDPrGiniBase** | 1.078 | 1.395 | 1.485 | 3.013 | 1.819 |
| **Tonga** | **GDPrHGiniH** | 1.274 | 1.631 | 1.621 | 2.809 | 1.951 |
| **Tonga** | **GDPrHGiniHv** | 1.609 | 2.031 | 2.015 | 3.362 | 2.402 |
| **Tonga** | **GDPrHGiniLow** | 0.904 | 1.182 | 1.177 | 2.163 | 1.441 |
| **Tonga** | **GDPrLGiniH** | 1.274 | 1.631 | 1.809 | 4.052 | 2.282 |
| **Tonga** | **GDPrLGiniHv** | 1.609 | 2.031 | 2.235 | 4.741 | 2.781 |
| **Tonga** | **GDPrLGiniLow** | 0.904 | 1.182 | 1.327 | 3.226 | 1.706 |
| **Tonga** | **NoCOVIDginiB** | 0.939 | 1.015 | 1.127 | 2.299 | 1.573 |
| **Trinidad and Tobago** | **GDPrGiniBase** | 0.412 | 0.508 | 0.521 | 0.246 | 0.111 |
| **Trinidad and Tobago** | **GDPrHGiniH** | 0.511 | 0.624 | 0.609 | 0.29 | 0.13 |
| **Trinidad and Tobago** | **GDPrHGiniHv** | 0.689 | 0.831 | 0.813 | 0.406 | 0.192 |
| **Trinidad and Tobago** | **GDPrHGiniLow** | 0.329 | 0.409 | 0.399 | 0.176 | 0.073 |
| **Trinidad and Tobago** | **GDPrLGiniH** | 0.511 | 0.624 | 0.671 | 0.341 | 0.164 |
| **Trinidad and Tobago** | **GDPrLGiniHv** | 0.689 | 0.831 | 0.89 | 0.473 | 0.239 |
| **Trinidad and Tobago** | **GDPrLGiniLow** | 0.329 | 0.409 | 0.443 | 0.211 | 0.094 |
| **Trinidad and Tobago** | **NoCOVIDginiB** | 0.293 | 0.322 | 0.356 | 0.191 | 0.088 |
| **Tunisia** | **GDPrGiniBase** | 0.397 | 0.423 | 0.466 | 0.652 | 0.26 |
| **Tunisia** | **GDPrHGiniH** | 0.484 | 0.515 | 0.54 | 0.681 | 0.294 |
| **Tunisia** | **GDPrHGiniHv** | 0.64 | 0.679 | 0.709 | 0.88 | 0.402 |
| **Tunisia** | **GDPrHGiniLow** | 0.321 | 0.343 | 0.362 | 0.467 | 0.186 |
| **Tunisia** | **GDPrLGiniH** | 0.484 | 0.515 | 0.591 | 0.883 | 0.355 |
| **Tunisia** | **GDPrLGiniHv** | 0.64 | 0.679 | 0.771 | 1.124 | 0.479 |
| **Tunisia** | **GDPrLGiniLow** | 0.321 | 0.343 | 0.399 | 0.62 | 0.228 |
| **Tunisia** | **NoCOVIDginiB** | 0.267 | 0.276 | 0.275 | 0.353 | 0.113 |
| **Turkey** | **GDPrGiniBase** | 0.156 | 0.126 | 0.113 | 0.047 | 0.008 |
| **Turkey** | **GDPrHGiniH** | 0.203 | 0.165 | 0.142 | 0.059 | 0.01 |
| **Turkey** | **GDPrHGiniHv** | 0.292 | 0.241 | 0.21 | 0.092 | 0.017 |
| **Turkey** | **GDPrHGiniLow** | 0.119 | 0.094 | 0.08 | 0.03 | 0.004 |
| **Turkey** | **GDPrLGiniH** | 0.203 | 0.165 | 0.153 | 0.071 | 0.014 |
| **Turkey** | **GDPrLGiniHv** | 0.292 | 0.241 | 0.225 | 0.11 | 0.023 |
| **Turkey** | **GDPrLGiniLow** | 0.119 | 0.094 | 0.087 | 0.038 | 0.006 |
| **Turkey** | **NoCOVIDginiB** | 0.153 | 0.149 | 0.141 | 0.059 | 0.011 |
| **Turkmenistan** | **GDPrGiniBase** | 0.767 | 0.611 | 0.559 | 0.132 | 0.005 |
| **Turkmenistan** | **GDPrHGiniH** | 0.926 | 0.744 | 0.66 | 0.156 | 0.006 |
| **Turkmenistan** | **GDPrHGiniHv** | 1.205 | 0.982 | 0.877 | 0.227 | 0.011 |
| **Turkmenistan** | **GDPrHGiniLow** | 0.629 | 0.496 | 0.434 | 0.089 | 0.003 |
| **Turkmenistan** | **GDPrLGiniH** | 0.926 | 0.744 | 0.71 | 0.19 | 0.009 |
| **Turkmenistan** | **GDPrLGiniHv** | 1.205 | 0.982 | 0.94 | 0.274 | 0.016 |
| **Turkmenistan** | **GDPrLGiniLow** | 0.629 | 0.496 | 0.471 | 0.111 | 0.004 |
| **Turkmenistan** | **NoCOVIDginiB** | 0.584 | 0.438 | 0.34 | 0.058 | 0.002 |
| **Uganda** | **GDPrGiniBase** | 44.25 | 44.71 | 43.94 | 34.55 | 14.54 |
| **Uganda** | **GDPrHGiniH** | 45.07 | 45.54 | 44.03 | 34.34 | 14.5 |
| **Uganda** | **GDPrHGiniHv** | 46.29 | 46.75 | 45.27 | 35.8 | 16.05 |
| **Uganda** | **GDPrHGiniLow** | 43.41 | 43.88 | 42.33 | 32.33 | 12.47 |
| **Uganda** | **GDPrLGiniH** | 45.07 | 45.54 | 45.53 | 36.73 | 16.72 |
| **Uganda** | **GDPrLGiniHv** | 46.29 | 46.75 | 46.73 | 38.13 | 18.34 |
| **Uganda** | **GDPrLGiniLow** | 43.41 | 43.88 | 43.89 | 34.81 | 14.6 |
| **Uganda** | **NoCOVIDginiB** | 42.8 | 42.47 | 41.42 | 32.02 | 12.05 |
| **Ukraine** | **GDPrGiniBase** | 0.089 | 0.085 | 0.075 | 0.013 | 0.006 |
| **Ukraine** | **GDPrHGiniH** | 0.113 | 0.109 | 0.09 | 0.015 | 0.007 |
| **Ukraine** | **GDPrHGiniHv** | 0.16 | 0.154 | 0.129 | 0.023 | 0.012 |
| **Ukraine** | **GDPrHGiniLow** | 0.068 | 0.065 | 0.053 | 0.007 | 0.003 |
| **Ukraine** | **GDPrLGiniH** | 0.113 | 0.109 | 0.101 | 0.022 | 0.009 |
| **Ukraine** | **GDPrLGiniHv** | 0.16 | 0.154 | 0.144 | 0.034 | 0.014 |
| **Ukraine** | **GDPrLGiniLow** | 0.068 | 0.065 | 0.06 | 0.011 | 0.004 |
| **Ukraine** | **NoCOVIDginiB** | 0.064 | 0.054 | 0.046 | 0.008 | 0.004 |
| **United Arab Emirates** | **GDPrGiniBase** | 0.127 | 0.128 | 0.14 | 0.071 | 0.024 |
| **United Arab Emirates** | **GDPrHGiniH** | 0.163 | 0.164 | 0.17 | 0.084 | 0.028 |
| **United Arab Emirates** | **GDPrHGiniHv** | 0.229 | 0.231 | 0.24 | 0.123 | 0.044 |
| **United Arab Emirates** | **GDPrHGiniLow** | 0.098 | 0.099 | 0.103 | 0.048 | 0.014 |
| **United Arab Emirates** | **GDPrLGiniH** | 0.163 | 0.164 | 0.187 | 0.103 | 0.039 |
| **United Arab Emirates** | **GDPrLGiniHv** | 0.229 | 0.231 | 0.262 | 0.15 | 0.061 |
| **United Arab Emirates** | **GDPrLGiniLow** | 0.098 | 0.099 | 0.114 | 0.06 | 0.021 |
| **United Arab Emirates** | **NoCOVIDginiB** | 0.093 | 0.09 | 0.087 | 0.056 | 0.02 |
| **United Kingdom** | **GDPrGiniBase** | 0.264 | 0.237 | 0.227 | 0.239 | 0.089 |
| **United Kingdom** | **GDPrHGiniH** | 0.33 | 0.297 | 0.276 | 0.272 | 0.101 |
| **United Kingdom** | **GDPrHGiniHv** | 0.449 | 0.407 | 0.38 | 0.375 | 0.148 |
| **United Kingdom** | **GDPrHGiniLow** | 0.209 | 0.187 | 0.172 | 0.17 | 0.057 |
| **United Kingdom** | **GDPrLGiniH** | 0.33 | 0.297 | 0.295 | 0.329 | 0.134 |
| **United Kingdom** | **GDPrLGiniHv** | 0.449 | 0.407 | 0.404 | 0.448 | 0.193 |
| **United Kingdom** | **GDPrLGiniLow** | 0.209 | 0.187 | 0.185 | 0.209 | 0.078 |
| **United Kingdom** | **NoCOVIDginiB** | 0.201 | 0.201 | 0.2 | 0.236 | 0.081 |
| **United States of America** | **GDPrGiniBase** | 1.019 | 0.959 | 0.924 | 0.829 | 0.482 |
| **United States of America** | **GDPrHGiniH** | 1.216 | 1.147 | 1.059 | 0.928 | 0.538 |
| **United States of America** | **GDPrHGiniHv** | 1.558 | 1.475 | 1.369 | 1.21 | 0.727 |
| **United States of America** | **GDPrHGiniLow** | 0.845 | 0.793 | 0.727 | 0.628 | 0.345 |
| **United States of America** | **GDPrLGiniH** | 1.216 | 1.147 | 1.148 | 1.076 | 0.663 |
| **United States of America** | **GDPrLGiniHv** | 1.558 | 1.475 | 1.477 | 1.39 | 0.884 |
| **United States of America** | **GDPrLGiniLow** | 0.845 | 0.793 | 0.794 | 0.739 | 0.434 |
| **United States of America** | **NoCOVIDginiB** | 0.914 | 0.917 | 0.929 | 0.865 | 0.505 |
| **Uruguay** | **GDPrGiniBase** | 0.169 | 0.168 | 0.172 | 0.084 | 0.008 |
| **Uruguay** | **GDPrHGiniH** | 0.217 | 0.217 | 0.213 | 0.1 | 0.01 |
| **Uruguay** | **GDPrHGiniHv** | 0.309 | 0.308 | 0.304 | 0.15 | 0.018 |
| **Uruguay** | **GDPrHGiniLow** | 0.13 | 0.129 | 0.127 | 0.055 | 0.005 |
| **Uruguay** | **GDPrLGiniH** | 0.217 | 0.217 | 0.229 | 0.125 | 0.014 |
| **Uruguay** | **GDPrLGiniHv** | 0.309 | 0.308 | 0.324 | 0.185 | 0.023 |
| **Uruguay** | **GDPrLGiniLow** | 0.13 | 0.129 | 0.137 | 0.071 | 0.006 |
| **Uruguay** | **NoCOVIDginiB** | 0.137 | 0.134 | 0.135 | 0.066 | 0.006 |
| **Uzbekistan** | **GDPrGiniBase** | 10.23 | 9.467 | 8.849 | 3.104 | 0.5 |
| **Uzbekistan** | **GDPrHGiniH** | 10.97 | 10.18 | 9.173 | 3.205 | 0.532 |
| **Uzbekistan** | **GDPrHGiniHv** | 12.11 | 11.29 | 10.23 | 3.807 | 0.705 |
| **Uzbekistan** | **GDPrHGiniLow** | 9.506 | 8.767 | 7.826 | 2.49 | 0.351 |
| **Uzbekistan** | **GDPrLGiniH** | 10.97 | 10.18 | 9.794 | 3.797 | 0.695 |
| **Uzbekistan** | **GDPrLGiniHv** | 12.11 | 11.29 | 10.88 | 4.465 | 0.907 |
| **Uzbekistan** | **GDPrLGiniLow** | 9.506 | 8.767 | 8.402 | 2.996 | 0.471 |
| **Uzbekistan** | **NoCOVIDginiB** | 9.225 | 8.448 | 7.727 | 2.584 | 0.401 |
| **Vanuatu** | **GDPrGiniBase** | 17.43 | 19.64 | 20.81 | 15.26 | 7.284 |
| **Vanuatu** | **GDPrHGiniH** | 18.34 | 20.58 | 21.2 | 14.44 | 7.076 |
| **Vanuatu** | **GDPrHGiniHv** | 19.71 | 21.98 | 22.59 | 15.72 | 8.077 |
| **Vanuatu** | **GDPrHGiniLow** | 16.52 | 18.71 | 19.34 | 12.76 | 5.831 |
| **Vanuatu** | **GDPrLGiniH** | 18.34 | 20.58 | 22.29 | 17.99 | 9.091 |
| **Vanuatu** | **GDPrLGiniHv** | 19.71 | 21.98 | 23.69 | 19.34 | 10.22 |
| **Vanuatu** | **GDPrLGiniLow** | 16.52 | 18.71 | 20.42 | 16.2 | 7.656 |
| **Vanuatu** | **NoCOVIDginiB** | 13.77 | 14.84 | 16.14 | 11.3 | 4.644 |
| **Venezuela, Bolivarian Republic** | **GDPrGiniBase** | 47.81 | 44.73 | 47.51 | 56.61 | 27.37 |
| **Venezuela, Bolivarian Republic** | **GDPrHGiniH** | 48.66 | 45.64 | 47.67 | 55 | 27.07 |
| **Venezuela, Bolivarian Republic** | **GDPrHGiniHv** | 49.91 | 46.99 | 48.96 | 56.05 | 28.83 |
| **Venezuela, Bolivarian Republic** | **GDPrHGiniLow** | 46.95 | 43.8 | 45.92 | 53.55 | 24.72 |
| **Venezuela, Bolivarian Republic** | **GDPrLGiniH** | 48.66 | 45.64 | 49.07 | 59.52 | 30.07 |
| **Venezuela, Bolivarian Republic** | **GDPrLGiniHv** | 49.91 | 46.99 | 50.31 | 60.41 | 31.81 |
| **Venezuela, Bolivarian Republic** | **GDPrLGiniLow** | 46.95 | 43.8 | 47.38 | 58.32 | 27.73 |
| **Venezuela, Bolivarian Republic** | **NoCOVIDginiB** | 41.32 | 38.61 | 34.56 | 42.86 | 15.29 |
| **Viet Nam** | **GDPrGiniBase** | 1.098 | 1.077 | 0.93 | 0.205 | 0.005 |
| **Viet Nam** | **GDPrHGiniH** | 1.291 | 1.268 | 1.04 | 0.23 | 0.007 |
| **Viet Nam** | **GDPrHGiniHv** | 1.622 | 1.595 | 1.323 | 0.321 | 0.012 |
| **Viet Nam** | **GDPrHGiniLow** | 0.925 | 0.907 | 0.731 | 0.14 | 0.003 |
| **Viet Nam** | **GDPrLGiniH** | 1.291 | 1.268 | 1.158 | 0.29 | 0.009 |
| **Viet Nam** | **GDPrLGiniHv** | 1.622 | 1.595 | 1.463 | 0.398 | 0.015 |
| **Viet Nam** | **GDPrLGiniLow** | 0.925 | 0.907 | 0.821 | 0.181 | 0.004 |
| **Viet Nam** | **NoCOVIDginiB** | 0.952 | 0.838 | 0.758 | 0.15 | 0.004 |
| **Yemen** | **GDPrGiniBase** | 56.27 | 59 | 59.48 | 41.96 | 12.64 |
| **Yemen** | **GDPrHGiniH** | 56.71 | 59.38 | 59.02 | 40.97 | 12.29 |
| **Yemen** | **GDPrHGiniHv** | 57.35 | 59.93 | 59.58 | 42.1 | 13.55 |
| **Yemen** | **GDPrHGiniLow** | 55.83 | 58.62 | 58.26 | 39.41 | 10.66 |
| **Yemen** | **GDPrLGiniH** | 56.71 | 59.38 | 60.55 | 44.25 | 14.78 |
| **Yemen** | **GDPrLGiniHv** | 57.35 | 59.93 | 61.06 | 45.3 | 16.11 |
| **Yemen** | **GDPrLGiniLow** | 55.83 | 58.62 | 59.86 | 42.81 | 13.04 |
| **Yemen** | **NoCOVIDginiB** | 49.63 | 43.61 | 40.44 | 24.91 | 4.543 |
| **Zambia** | **GDPrGiniBase** | 59.45 | 60.09 | 60.19 | 54.28 | 41.27 |
| **Zambia** | **GDPrHGiniH** | 60.29 | 60.91 | 60.63 | 54.24 | 41.6 |
| **Zambia** | **GDPrHGiniHv** | 61.54 | 62.14 | 61.87 | 55.76 | 43.65 |
| **Zambia** | **GDPrHGiniLow** | 58.6 | 59.26 | 58.97 | 52.18 | 38.85 |
| **Zambia** | **GDPrLGiniH** | 60.29 | 60.91 | 61.39 | 56.33 | 43.67 |
| **Zambia** | **GDPrLGiniHv** | 61.54 | 62.14 | 62.59 | 57.76 | 45.66 |
| **Zambia** | **GDPrLGiniLow** | 58.6 | 59.26 | 59.78 | 54.39 | 40.97 |
| **Zambia** | **NoCOVIDginiB** | 58.27 | 58.48 | 58.16 | 51.47 | 39.25 |
| **Zimbabwe** | **GDPrGiniBase** | 38.92 | 37.19 | 36.16 | 27.03 | 8.284 |
| **Zimbabwe** | **GDPrHGiniH** | 39.87 | 38.17 | 36.42 | 27.22 | 8.453 |
| **Zimbabwe** | **GDPrHGiniHv** | 41.27 | 39.62 | 37.9 | 28.84 | 9.709 |
| **Zimbabwe** | **GDPrHGiniLow** | 37.95 | 36.2 | 34.41 | 25.06 | 6.906 |
| **Zimbabwe** | **GDPrLGiniH** | 39.87 | 38.17 | 37.79 | 28.97 | 9.739 |
| **Zimbabwe** | **GDPrLGiniHv** | 41.27 | 39.62 | 39.24 | 30.56 | 11.06 |
| **Zimbabwe** | **GDPrLGiniLow** | 37.95 | 36.2 | 35.81 | 26.82 | 8.082 |
| **Zimbabwe** | **NoCOVIDginiB** | 34.17 | 33.99 | 33.6 | 25.21 | 7.325 |
