# Supplementary material for: How many people is the COVID-19 pandemic pushing into poverty? A long-term forecast to 2050 with alternative scenarios
Source: PLoS One. 2022 Jul 8;17(7):e0270846. doi: 10.1371/journal.pone.0270846 (PMC9269768; doi:10.1371/journal.pone.0270846)
Supplement: S1 File — (PDF) [file pone.0270846.s002.pdf]

Result replication instructions: The impact of COVID-19 on global extreme poverty: a long-term projection

The printable version is no longer supported and may have rendering errors. Please update your browser bookmarks and please use the default browser print function instead.

International Futures (IFs) is a tool for thinking about long-term country-specific, regional, and global futures. IFs integrates forecasts across different sub-models, including: population, economy, agriculture, education, energy, sociopolitical, international political, environment, technology, infrastructure, and health. The interconnection of these sub-models allows IFs to simulate how changes in one system lead to changes across all other systems.

These instructions will help you download and run scenarios that reflect work done in this study. If you would like additional information on specific result replication please direct requests to pardee.center@du.edu.

☐

Contents

Data Availability
General instructions
Necessary files
Specific instruction
- How to install IFs
- How to add scenario files
- How to batch run scenarios
- Visualizing results

Relevant Documentation

Data Availability

All historical data used in this analysis can be found in the IFs historical database included in the software (see below). After installing the software, users can view or download historical data from within the 'Data Analysis' options accessible through the main menu. More information regarding how to access and work with historical data can be found here. Results presented in this study are available by running the scenarios (instructions and necessary files are listed below).

General instructions

1. Download model and all supplementary files (see below)
2. Install IFs
3. Add supplementary files to their specified locations (see below)
4. Run the scenarios (see below)

Necessary files

IFs Model: Link to download IFs (version 7.82)

Scenario files: Link to download SCE files

Specific instruction

How to install IFs

Requirements for installing and running IFs on your computer can be found on our website here. Requirements and instructions on installing IFs on a Mac can be found here.

After downloading IFs (as a zipped folder), you will need to extract all the files from the zipped folder. Then double-click 'IFsSetupStarter". This should initiate the installation. After installation, go to 'Start' and select 'IFs' from the list of programs to run IFs.

How to add scenario files

Scenario files can be manually added to IFs once they have been downloaded. To manually add scenario files (files with the .sce extension) to IFs, you will need to copy the downloaded .sce file to the Scenario folder within IFs. The scenario folder can be accessed using the following path:

C:/Users/Public/IFs/Scenario

Create a new folder and copy over all scenario files that are necessary for the reproduction of desired results. For more information on the file structure in IFs please click here.

How to batch run scenarios

The batch run feature in IFs runs multiple scenarios in succession and saves each resulting

run files. To do this, select the ‘Run’ sub-option from ‘Scenario Analysis’ in the main menu bar and then select the option to perform a ‘Batch Run’. From this point all folders contained within the ‘IFs/Scenario’ folder should be visible. Navigate to the desired folder, choose a run horizon, and then press ‘Select.’ IFs will then run each scenario contained within the selected folder save them as a sperate run file.

Visualizing results

There are many ways to visualize Base Case and scenario results in IFs. The Flexible Displays allows a user to display a wide variety of projected variables (sometimes in combination with historical data series), create graphs and view tables of these displays. This display also allows the user to compare results across countries, groups, and scenarios. Not all of the variables available in IFs can be found in Flexible Displays; its purpose is to provide users with access to commonly used variables. If you wish to access the full range of variables and parameters, you can do so in Self-Managed Displays. For more information on using Flexible Displays, click here and for more information on self managed displays, please click here.

Relevant Documentation

IFs training manual: Link to IFs Training Manual

IFs scenario guide: Link to IFs Scenario Guide

Model documentation: Link to all documentation

Relevant code: Link to all code

Retrieved from
"https://pardeewiki.du.edu//index.php?title=Result_replication_instructions:_The_impact_of_COVID-19_on_global_extreme_poverty:_a_long-term_projection&oldid=9441"

This page was last edited on 1 April 2022, at 20:20.
